# Beyond benchmarking: an expert-guided consensus approach to spatially aware clustering

**DOI:** 10.1038/s41592-026-03194-8

**Published:** 2026-08-24

**Authors:** Jieran Sun, Kirti Biharie, Peiying Cai, Niklas Müller-Bötticher, Paul Kiessling, Meghan A. Turner, Søren Helweg Dam, Florian Heyl, Sarusan Kathirchelvan, Martin Emons, Samuel Gunz, Sven Twardziok, Amin El-Heliebi, Martin Zacharias, SpaceHack 2.0 participants, Jieran Sun, Kirti Biharie, Peiying Cai, Niklas Müller-Bötticher, Paul Kiessling, Meghan A. Turner, Florian Heyl, Sarusan Kathirchelvan, Sven Twardziok, Søren Helweg Dam, Fadhl Alakwaa, Shahul Alam, María Calleja, Yuzhou Chang, Thomas Chartrand, Nigel S. Chou, Estella Y. Dong, Michael Fletcher, George Gavriilidis, Alexander Kanitz, Sameesh Kher, Louis Kümmerle, Francesca A. Luongo, Qirong Mao, Giorgia Moranzoni, Mar M. Moreno, Anastasiia Okhtienko, Lena Perry, Lucie Pfeiferova, Daryna Pikulska, Shyam Prabhakar, Rasool Saghaleyni, Zaira Seferbekova, Vipul Singhal, Divya Sitani, Charlotte Soneson, Sebastian Tiesmeyer, Marco Varrone, Siao-Han Wong, Liya Zaygerman, Teresa Zulueta-Coarasa, Marcel Reinders, Raphael Gottardo, Christoph Kuppe, Brian Long, Ahmed Mahfouz, Mark D. Robinson, Naveed Ishaque, Roland Eils, Marcel Reinders, Raphael Gottardo, Christoph Kuppe, Brian Long, Ahmed Mahfouz, Mark D. Robinson, Naveed Ishaque

**Affiliations:** 1 Biomedical Data Science Center, Centre Hospitalier Universitaire Vaudois, Lausanne, Switzerland; 2 Department of Human Genetics, Leiden University Medical Center, Leiden, the Netherlands; 3 Delft Bioinformatics Lab, Delft University of Technology, Delft, the Netherlands; 4 Department of Molecular Life Sciences and SIB Swiss Institute of Bioinformatics, University of Zurich, Zurich, Switzerland; 5 Berlin Institute of Health at Charité - Universitätsmedizin Berlin, Center of Digital, Berlin, Germany; 6 Department of Nephrology, Rheumatology, and Clinical Immunology, University Hospital RWTH Aachen, Aachen, Germany; 7 Allen Institute for Brain Science, Seattle, WA USA; 8 DTU Health Tech, Technical University of Denmark, Ørsteds Plads, Kongens Lyngby, Denmark; 9 LEO Foundation Skin Immunology Research Center, Department of Immunology and Microbiology, University of Copenhagen, Copenhagen, Denmark; 10 German Cancer Research Center (DKFZ), Division of Computational Genomics and Systems Genetics, Heidelberg, Germany; 11 The German Human Genome-Phenome Archive, Heidelberg, Germany; 12 Division of Cell Biology, Histology and Embryology, Gottfried Schatz Research Center, Medical University of Graz, Graz, Austria; 13 Diagnostic and Research Institute of Pathology, Medical University of Graz, Graz, Austria; 14 Department of Internal Medicine, Division of Nephrology, University of Michigan, Ann Arbor, MI USA; 15 Computational Biology Department, Carnegie Mellon University, Pittsburgh, PA USA; 16 CIMA Universidad de Navarra, Pamplona, Spain; 17 Department of Biomedical Informatics, College of Medicine, Ohio State University, Columbus, OH USA; 18 Allen Institute for Neural Dynamics, Seattle, WA USA; 19 Genome Institute of Singapore, Agency for Science, Technology and Research (A*STAR), Singapore, Singapore; 20 Nature Genetics, Berlin, Germany; 21 Institute of Applied Biosciences, Centre for Research and Technology Hellas, Thessaloniki, Greece; 22 Biozentrum, University of Basel, Basel, Switzerland; 23 SIB Swiss Institute of Bioinformatics, Quartier Sorge Batiment Amphipole, Lausanne, Switzerland; 24 Helmholtz Zentrum München, Institute of Computational Biology, Neuherberg, Germany; 25 CSEM, Allschwil, Switzerland; 26 ETHZ, BMIC - Biomedical Image Computing lab, Zurich, Switzerland; 27 OMAPiX GmbH Raiffeisenstraße 3, Langenfeld, Germany; 28 Technical University of Munich, School of Medicine, Institute of Virology, Munich, Germany; 29 University of Michigan, Undergraduate Research Opportunity Program, Ann Arbor, MI USA; 30 Laboratory of Genomics and Bioinformatics, Institute of Molecular Genetics of the Czech Academy of Sciences, Prague, Czech Republic; 31 Department of Informatics and Chemistry, Faculty of Chemical Technology, University of Chemistry and Technology, Prague, Czech Republic; 32 Department of Biology and Biological Engineering, Chalmers University of Technology, Gothenburg, Sweden; 33 National Bioinformatics Infrastructure of Sweden, Scilifelab, Sweden; 34 Division of Artificial Intelligence in Oncology (B450), German Cancer Research Center (DKFZ), Heidelberg, Germany; 35 Data Science Lab, Hertie School, Berlin, Germany; 36 Friedrich Miescher Institute for Biomedical Research, Basel, Switzerland; 37 Department of Computational Biology, University of Lausanne, Lausanne, Switzerland; 38 Swiss Cancer Center Léman, Lausanne, Switzerland; 39 DKFZ Hector Cancer Institute at the University Medical Center Mannheim, Mannheim, Germany; 40 Division of Personalized Medical Oncology (A420), German Cancer Research Center (DKFZ), Heidelberg, Germany; 41 German Center for Lung Research (DZL), Giessen, Germany; 42 Department of Personalized Oncology, University Hospital Mannheim, Medical Faculty Mannheim, University of Heidelberg, Mannheim, Germany; 43 Division Applied Bioinformatics (B330), German Cancer Research Center (DKFZ), Heidelberg, Germany; 44 European Molecular Biology Laboratory, European Bioinformatics Institute, Hinxton, UK

**Keywords:** Statistical methods, Computational biology and bioinformatics, Transcriptomics

## Abstract

Spatial omics technologies have revolutionized the study of tissue architecture and cellular heterogeneity by integrating molecular profiles with spatial localization. In spatially resolved transcriptomics, delineating higher-order anatomical structures is critical for understanding how cellular organization affects function. However, the reliability of current benchmarks of spatially aware clustering (SAC) methods is undermined by their narrow focus on Visium and brain tissue datasets and the incorrect interpretation of manual annotation as ground truth. Here we present SACCELERATOR, a community-driven, extensible framework that standardizes data formatting, method integration and metric evaluation, enabling rapid inclusion of new methods and datasets. Our analysis revealed substantial limitations in the generalizability and reproducibility of SAC methods and shows that anatomical labels commonly used as ground truths are often biased, error prone and unsuitable for benchmarking. Rather than ranking methods, we propose a consensus-guided workflow where descriptive spatial metrics highlight high-entropy regions of method disagreement, enabling targeted feedback for tissue experts. Applied to brain and cancer datasets, this approach uncovered biologically meaningful patterns overlooked by individual SAC methods and manual annotations, highlighting the need for iterative, expert-in-the-loop evaluation.

## Abstract (web summary)

Benchmarking spatially aware clustering is challenging. SACCELERATOR is an open-source, extensible framework that formalizes expert-guided spatial clustering analysis.

## Main

Spatially resolved transcriptomics (SRT) technologies map gene expression while preserving the spatial architecture of tissues, offering insights into how cells are organized into higher-order anatomical structures, such as tissues and organs^1,2^. Delineating higher-order anatomical structures is pivotal for advancing our understanding of biological systems. These structures reveal how cells communicate within and between tissues, offering new perspectives on the mechanisms underlying both healthy and diseased states.

Since 2020, more than 50 spatially aware clustering (SAC) methods have been developed to delineate structures in SRT data into spatial regions. However, terminology describing these regions remains inconsistent across studies, with ‘spatial domains’ or ‘spatial clusters’ loosely defined as tissue regions exhibiting spatially coherent gene expression patterns^3^. Several terms are in use: ‘spatially homogeneous regions’^4^, ‘cellular neighborhoods’^5^, ‘schematics’^6^, ‘niches’^7,8^ and ‘milieu’^9^, which should also be distinguished from terms around anatomical structures, such as ‘functional tissue units’^10^ or ‘parcellations’^11^. This lack of standardized terminology reflects the diversity of biological questions and spatial scales addressed by SRT, ranging from cells^12^ to organs^13^. It also suggests that spatial clusters may not always align with biological structures of interest, highlighting the need for clarity in definitions and expectations.

Robust benchmarking is required to guide method selection and parameterization. Previous studies have compared SAC methods across various settings, including SRT technologies, tissues, simulated datasets, preprocessing and random seed initializations^3,14–16^. However, these studies are not easily extensible to new methods or datasets and are limited in scope, focusing primarily on brain datasets generated by early technologies such as Visium.

The most common approach to benchmark SAC methods is to compare their output with ground truth (GT) annotations. Traditionally, these GT annotations are manually annotated on the basis of apparent structures and regions using histological stains such as hematoxylin and eosin (H&E), often augmented with tissue-specific labels to visualize cytoarchitecture, pathology or other features of interest^17^. As GT annotations are commonly determined manually by experts, they are prone to human error and knowledge biases. The lack of well-defined ontologies describing anatomical structures hinders accurate and standardized definition of GTs, although efforts are underway to define cell-type ontologies using single-cell and SRT technologies^18–21^.

The challenges of benchmarking SAC methods became apparent during the BioHackathon Germany SpaceHack 2.0 project^22^. Our initial goal was to implement an extensible, community-driven benchmark to quantitatively evaluate the performance of SAC methods across different SRT technologies and tissues, but we encountered three main obstacles. First, most SAC methods were not sufficiently demonstrated on modern SRT technologies. Second, the conceptual complexity in defining GTs rendered standard performance metrics difficult to interpret. Third, in most real-world scenarios, SAC methods are utilized interactively and iteratively, giving more importance to being able to investigate the output of different methods and parameters.

To address these challenges, we propose a consensus-guided, expert-in-the-loop framework to systematically evaluate commonalities and differences between methods and parameter choices. This framework enhances tissue annotations, facilitates method recommendations and reflects the iterative, collaborative nature of spatial omics research. We present evidence of inconsistencies in previously reported method performances and demonstrate the value of leveraging consensus among SAC results. We also highlight the need for improved computational efficiency to accommodate modern large-scale SRT datasets. Finally, we present two case studies to illustrate the utility of method consensus and entropy in expert-guided analysis. Our extensible framework, SACCELERATOR, is available on GitHub with clear contribution guidelines and templates to enable reuse and extension.

## Results

### Reported performance of SAC methods is inconsistent across studies

We observed inconsistencies in reported method performances across existing SAC benchmarks. To evaluate this systematically, we conducted a meta-analysis of 13 studies^7,12,14,23–32^, using the widely used Visium dataset of the human brain dorsolateral prefrontal cortex (LIBD DLPFC) region^33^.

**Fig. 1:**
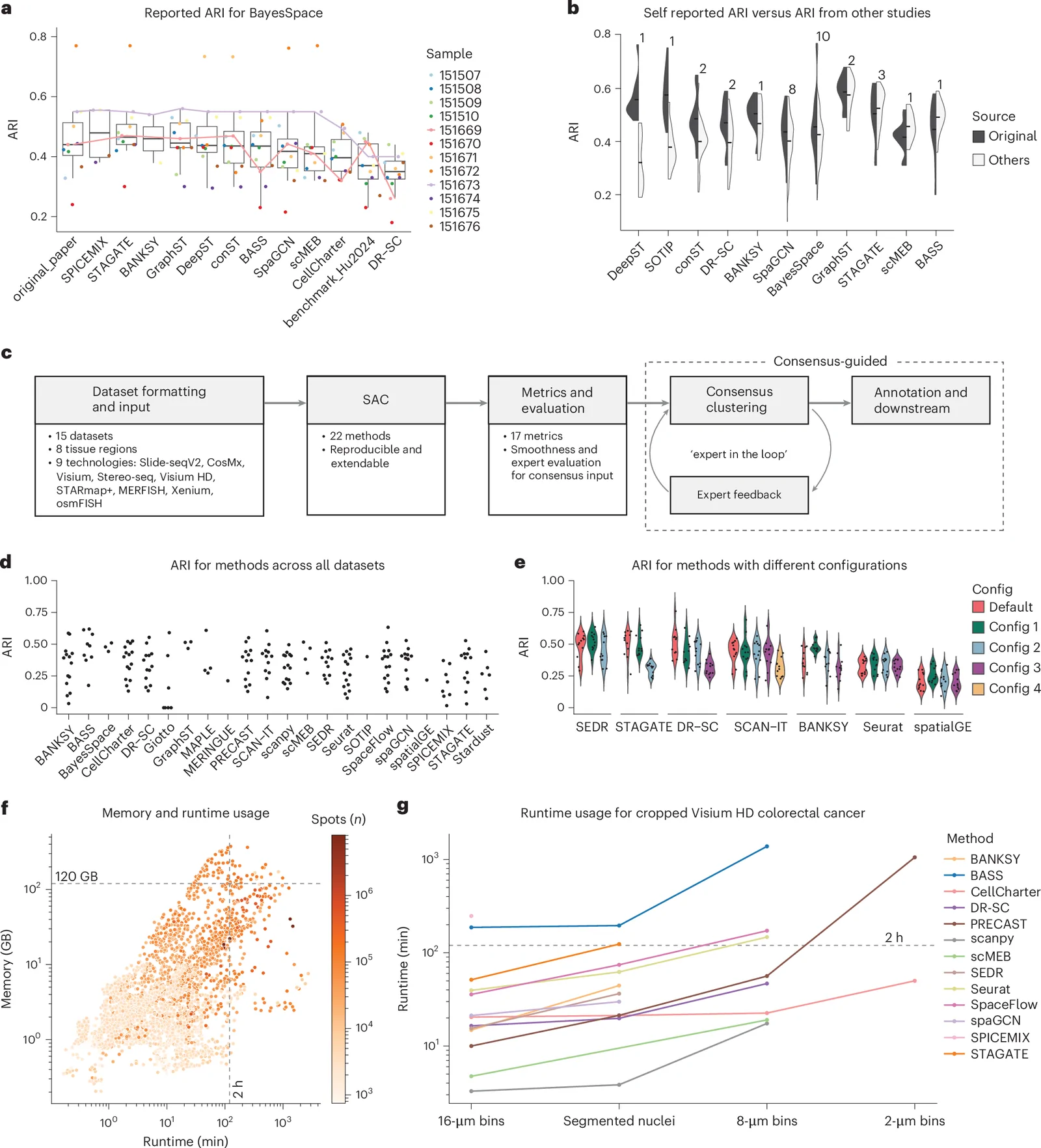
Addressing inconsistent reporting of method performance. **a**, Reported ARI of BayesSpace on the LIBD DLPFC, in the original BayesSpace study and other studies. The reported ARI for each sample in the dataset is depicted in a different color, with those for slices 151669 and 151673 connected with a line. Studies after BayesSpace are ordered by decreasing median ARI. Box plots show medians (central line), 25th–75th percentiles (box limits) and whiskers extending to the most extreme data point within 1.5× the interquartile range from the box. Data points beyond whiskers are plotted individually (*n* = 12). **b**, The original self-reported ARI (dark gray) versus the ARI reported in other studies (light gray) for method performance on the LIBD DLPFC dataset. Methods are arranged by the difference of the mean of self-reported ARI versus those in other studies. **c**, Schematic of the SACCELERATOR framework. **d**, ARI for methods across different datasets. **e**, ARI for selected methods with multiple configurations applied to the LIBD DLPFC dataset. **f**, Memory usage versus CPU runtime across SRT technologies, colored by the number of spots/cells in those datasets. **g**, CPU runtime of methods on 25% of the Visium HD colorectal cancer dataset across various bin sizes.

We focused on BayesSpace, a foundational SAC method, as a benchmark control. We expected relatively consistent BayesSpace results across studies; however, collected adjusted Rand indices (ARIs) revealed substantial variability (Fig. 1a). For instance, slice 151673 showed strong and consistent performance, whereas the results of slice 151669 fluctuated dramatically, highlighting the lack of robustness of reported method performances across previous studies.

As expected, we observed that performances reported in method papers are frequently higher than those reported by subsequent competitors (Fig. 1b). This discrepancy could arise from original studies optimizing their methods to demonstrate superior performance or suboptimal parameter choices in subsequent comparisons. While neutral benchmarking studies have aimed to systematically address such biases, we still encountered inconsistencies. For example, BayesSpace was ranked as 4th of 14 methods by Yuan et al.^3^ but 13th of 16 methods by Hu et al.^14^ on the basis of the same LIBD DLPFC dataset. Together, this meta-analysis questions the utility of conventional SAC benchmarks.

### An extensible framework for running and evaluating SAC methods

Given the limitations of existing benchmarking efforts, we investigated the utility of SAC methods across various real-world scenarios. To achieve this, we developed SACCELERATOR, a modular and reproducible workflow built on Snakemake^34^, specifically designed for extension with new datasets, methods and evaluation metrics (Fig. 1c). Within this framework, each dataset, method and metric is encapsulated as an independent module, with standardized command-line interfaces and outputs. This modular structure enables seamless integration of additional methods or datasets and method-specific configuration files to facilitate systematic exploration of parameter spaces.

Recognizing that SRT technologies produce data in diverse formats, we defined a set of necessary and optional metadata for each dataset. This ensures that downstream analyses are agnostic to the underlying technology and that data can be easily accessed across programming languages.

The SACCELERATOR framework enables performance evaluation via both spatially agnostic clustering metrics, such as ARI or normalized mutual information (NMI), as well as spatially aware metrics, such as spatial chaos score (CHAOS)^35^, percentage of abnormal spots (PAS) score^35^ or spot entropy^36^. These spatially aware metrics consider the spatial location of the predicted labels and/or evaluate the spatial continuity of the clusters^35–37^. Moreover, the clustering results of different parameter combinations or multiple methods can be aggregated using consensus clustering approaches.

SACCELERATOR currently includes data from over 170 tissue samples from 15 datasets, generated using the sequencing-based SRT technologies Visium^38^, Visium HD^39^, Slide-seqV2^40^ and Stereo-seq as well as the imaging-based technologies Xenium, CosMx, STARmap PLUS^41^ and MERFISH^42^ (Supplementary Table 1). The framework applied the 22 implemented methods (Supplementary Table 2) to these datasets, each with one to five parameter configurations (61 total combinations; Supplementary Table 3). Accuracy-based metrics, such as ARI and NMI, and spatial continuity-based metrics (Supplementary Table 4), such as smoothness entropy (SE) and CHAOS, show generally concordant correlation and ranking (Supplementary Figs. 1 and 2).

To assess the impact of parameterization on performance variability, we evaluated each method using both default settings and parameters recommended in tutorials or dataset-specific analyses after quality control and filtering steps. In general, we observed substantial variation in SAC method performance as measured by ARI (Fig. 1d). Surprisingly, we observed that differences between parameter configurations can exceed those between different methods (Fig. 1e and Supplementary Fig. 3). Some methods exhibited stable performance across configurations (for example, SEDR), while others (for example, STAGATE) achieved results ranging from top- to bottom-tier performance, probably reflecting sensitivity to technology-specific parameters (Fig. 1e). This pronounced sensitivity underscores the challenge for users in selecting optimal settings and highlights the need for clear guidance (Supplementary File 1) and robust defaults.

### Few current methods scale to modern datasets

Newer SRT technologies have improved spatial resolution and/or gene plexity, leading to a substantial increase in data volume. The smallest dataset we evaluated contained fewer than 3,500 spots (Visium LIBD DLPFC), while the largest dataset contained more than 460,000 cells (CosMx liver cancer). To assess whether SAC methods scale to large datasets, we applied them to large Visium HD, Stereo-seq, Xenium and CosMx datasets. We measured the central processing unit (CPU) runtime and memory usage with respect to the number of spatial observations (that is, cells, bins and spots) and features (genes). We found that both runtime and memory usage increased exponentially with the number of spatial observations (Fig. 1f), whereas the number of features had little impact, probably owing to the widespread use of feature selection and/or dimensionality reduction steps in most SAC methods (Supplementary Fig. 4a). Similarly, the specific SRT technology had less impact than the number of spatial observations (Supplementary Fig. 5). Most methods scaled well until 200,000 spatial observations (Fig. 1f), but only 5 of 22 methods were able to process the largest dataset (CosMx liver cancer) on a compute node with 500-GB RAM and ten cores within 2 days.

To further explore method scalability, we examined resource usage for the Visium HD colorectal cancer dataset across multiple bin sizes (2, 8 and 16 μm, resulting in 8, 0.5 and 0.125 million bins, respectively; Supplementary Fig. 4b). While 11 of 22 completed for the 16-μm bins, only 5 could process the 8-μm bins and just CellCharter managed to process the 2-μm bins within the memory and runtime constraints; all other methods exceeded the 500-GB memory limit. As only one method completed for all bin sizes, we repeated the experiment using a cropped 25% subset of the dataset (Fig. 1g). This increased the number of methods able to process the 16-μm bins to 13 (of 22), with 8 successfully finishing for the 8-μm bins. For the smallest bin size, only PRECAST could now complete alongside CellCharter; however, results were dissimilar (ARI, 0.10), with CellCharter more consistent with GT labels (ARIs, 0.31 versus 0.07; Supplementary Fig. 6).

These results demonstrate that most SAC methods do not scale to state-of-the-art SRT technologies and may not be usable for large-scale analyses.

### Over-reliance on GT annotations complicates method evaluations

**Fig. 2:**
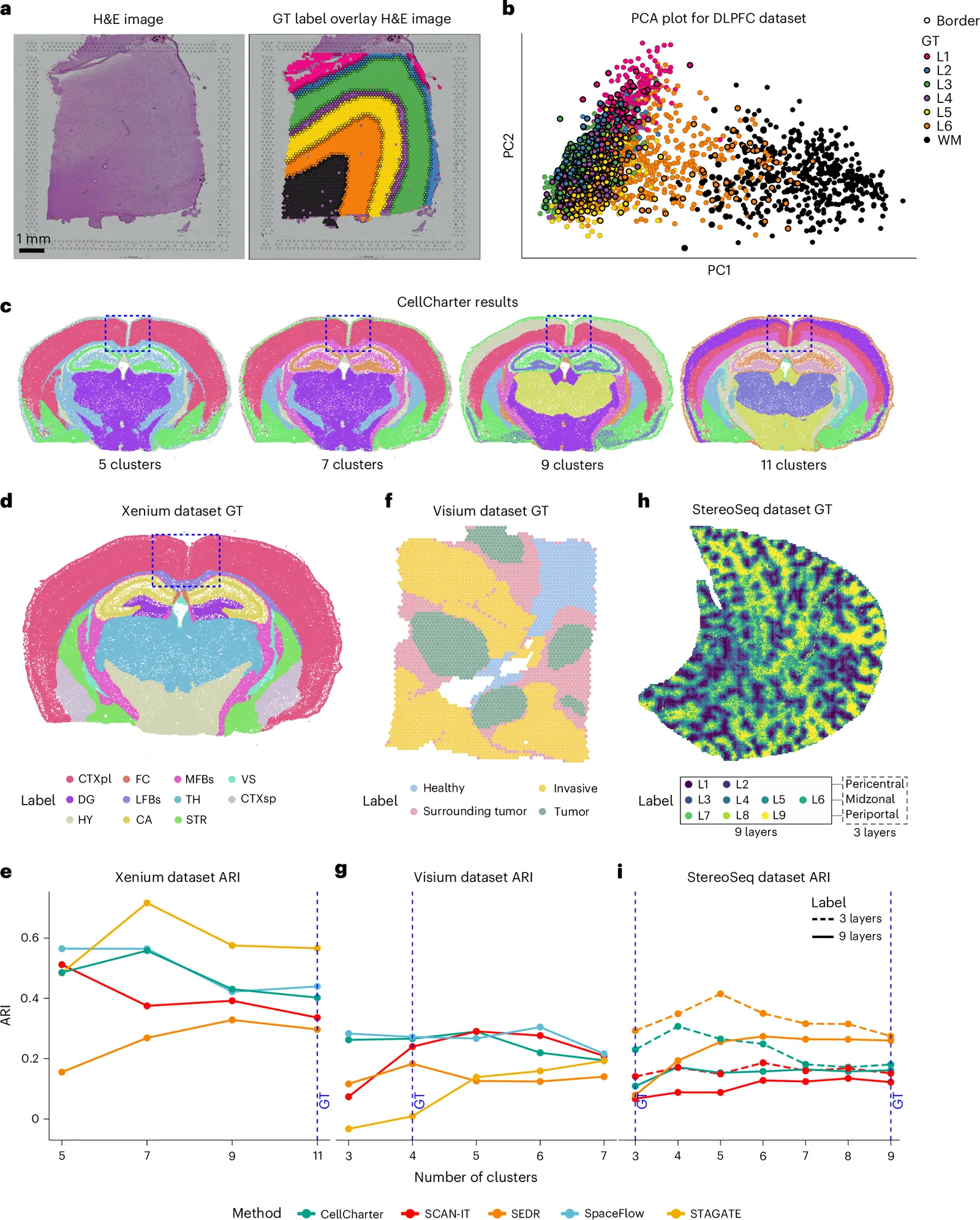
Challenges in utilizing GTs effectively for benchmarking. **a**, H&E images (left) and GT annotation (right) of dorsolateral prefrontal cortex from the Visium LIBD DLPFC Br8100_151673 slice. **b**, LIBD DLPFC Br8100_151673 slice, projected on the first and second PC, colored by GT labels. Spots spatially located at cluster borders are marked with black outlines. **c**, CellCharter results on Xenium mouse brain dataset with 5, 7, 9 and 11 clusters. **d**,**f**,**h**, Spatial plots of GT labels of Xenium mouse brain dataset (**d**), Visium breast cancer dataset (**f**) and Stereo-seq mouse liver dataset (**h**). **e**,**g**,**i**, ARI against the GT labels for methods with different cluster numbers on the Xenium (**e**), Visium (**g**) and Stereo-seq (**i**) datasets. The cluster number of the GT labels is marked by dashed blue lines. Two sets of GT labels are shown in the Stereo-seq dataset (**i**), marked by different line types.

One key limitation in benchmarking studies of SAC methods is the reliance on GT labels. GT labels are often derived from multimodal information not fully reflected in the transcriptome alone. For example, in the Visium LIBD DLPFC dataset^33^, GT annotations were defined by cross-referencing histological H&E staining (Fig. 2a) with marker gene expression. However, using transcriptional data alone, most isocortical layers cluster closely in the first two principal components (PCs), except for white matter and partially layer 6 (Fig. 2b). Similar intermixed patterns are observed across datasets (Supplementary Fig. 7).

Gene expression differences across the isocortical layers were subtle and could be further obscured by technical limitations of sequencing-based SRT technologies, including coarse resolution (multiple cells per spot), and transcript-capture biases (for example, lateral diffusion)^43^. Beyond these technical constraints, conceptual misalignments may exist between spatial clusters and the biological reality of tissue organization. Many biological phenomena, including transcriptomic gradients and transitional cell states, are continuous and diffusive processes, whereas SAC methods impose discrete boundaries.

GT label assignment is also inherently subjective, reflecting the annotator’s biological interests and scale of analysis. For instance, in the Xenium mouse brain datasets (Fig. 2c), the entire isocortex is labeled as a single region in the GT (Fig. 2d). Yet, when SAC methods were configured to identify the same number of clusters as the GT, some detected finer layered structures within the isocortex (Fig. 2c; 11 clusters, and Supplementary Fig. 8). Although biologically meaningful, these layers deviate from the GT annotations and yield lower ARI scores. Conversely, using fewer clusters (Fig. 2c; 7 and 9 clusters) produces a coarser isocortical structure that better matches the GT labels with higher ARI scores (Fig. 2e, green). However, this reduced granularity can obscure other anatomical distinctions, such as between the thalamus and hypothalamus, which emerge at higher cluster numbers (Supplementary Fig. 9 and Supplementary File 2). Subjective GT definitions panelize alternative clustering structures and induce granularity bias, where methods often achieve peak ARI at cluster numbers different from the GT labels (Fig. 2e,g,i). This pattern is pervasive across datasets and methods.

The limitations of GT-based evaluation are further compounded by divergent annotation objectives. For example, the GT annotation for the Visium breast cancer dataset^44^ (Fig. 2f) defines four manually annotated regions: tumor, tumor-surrounding, invasive and healthy tissue. While these labels provide a biologically motivated scaffold, their coarse granularity introduces evaluation biases: methods such as STAGATE and SCAN-IT achieve peak ARIs only when configured to identify more clusters than in the GT (Fig. 2g). More fundamentally, there is an intrinsic mismatch: GT labels capture broad pathological zones on the basis of histological features, whereas most SAC methods are optimized to identify transcriptionally coherent spatial patterns. This conceptual disconnect leads to uniformly low ARI scores across all methods (mean ARI, 0.20; Fig. 2g), even when the detected clusters correspond to meaningful tissue structures (Supplementary Fig. 10).

In the Stereo-seq mouse liver dataset^44^ (Fig. 2h), the GT labels were annotated to reflect hepatocyte zonation patterns between central and portal veins in liver lobules^45^. Most SAC methods failed to capture the pattern and yielded low ARI scores (Fig. 2i and Supplementary Fig. 11), which can be attributed to several factors, including the use of selected markers for GT annotation and the granularity of the zonation definition.

To assess the impact of expert-supervised gene selection, we identified 925 zonation-associated genes from three studies^46–48^ that were not used in the original GT annotation. Limiting to those genes produced mixed effects: SpaceFlow achieved a higher ARI, whereas SEDR and CellCharter showed a reduced ARI compared with analyses using all genes (Supplementary Fig. 12). These results suggest that some SAC methods may benefit from leveraging curated gene sets rather than relying solely on unsupervised gene selection.

We further investigated the effect of annotation granularity. The original GT divided the zonation gradient into nine equal parts, although these can also be grouped into three biologically established layers: pericentral, midzonal and periportal. Using this three-layer label substantially increased ARI scores compared with the original nine-layer GT (Fig. 2h,i), highlighting the importance of aligning GT granularity with the biological structures of interest.

As manual GT annotation is labor-intensive, some datasets have used computationally generated references (Supplementary Table 1). For example, the GT for the Stereo-seq mouse embryo dataset was produced using spatially constrained clustering, a method built on the Leiden algorithm^13^. This methodological overlap probably explains why Leiden clustering implementations in Seurat and Scanpy achieved the highest mean ARI scores (0.428 and 0.421, respectively; Supplementary Fig. 13). Similar circularity is also found in SAC benchmarking studies of the STARmap mouse cortex dataset: BASS, the method used to generate GT labels, consistently achieved top-tier performance^3,14^.

Collectively, these examples highlight systematic challenges in GT-based SAC benchmarking. While careful curation may mitigate some issues, the inherent limitations of GT labels still undermine their reliability and the validity of current benchmarking practices.

### Methods are often more similar to each other than to the GT

Given the challenges in effectively utilizing GT references, we explored the similarity of clustering results produced by different SAC methods. We compared ‘base clusterings’ (that is, clustering results from multiple methods and configurations) with each other and also computed SE as an intrinsic, GT-independent performance measure, as some level of spatial continuity is expected.

**Fig. 3:**
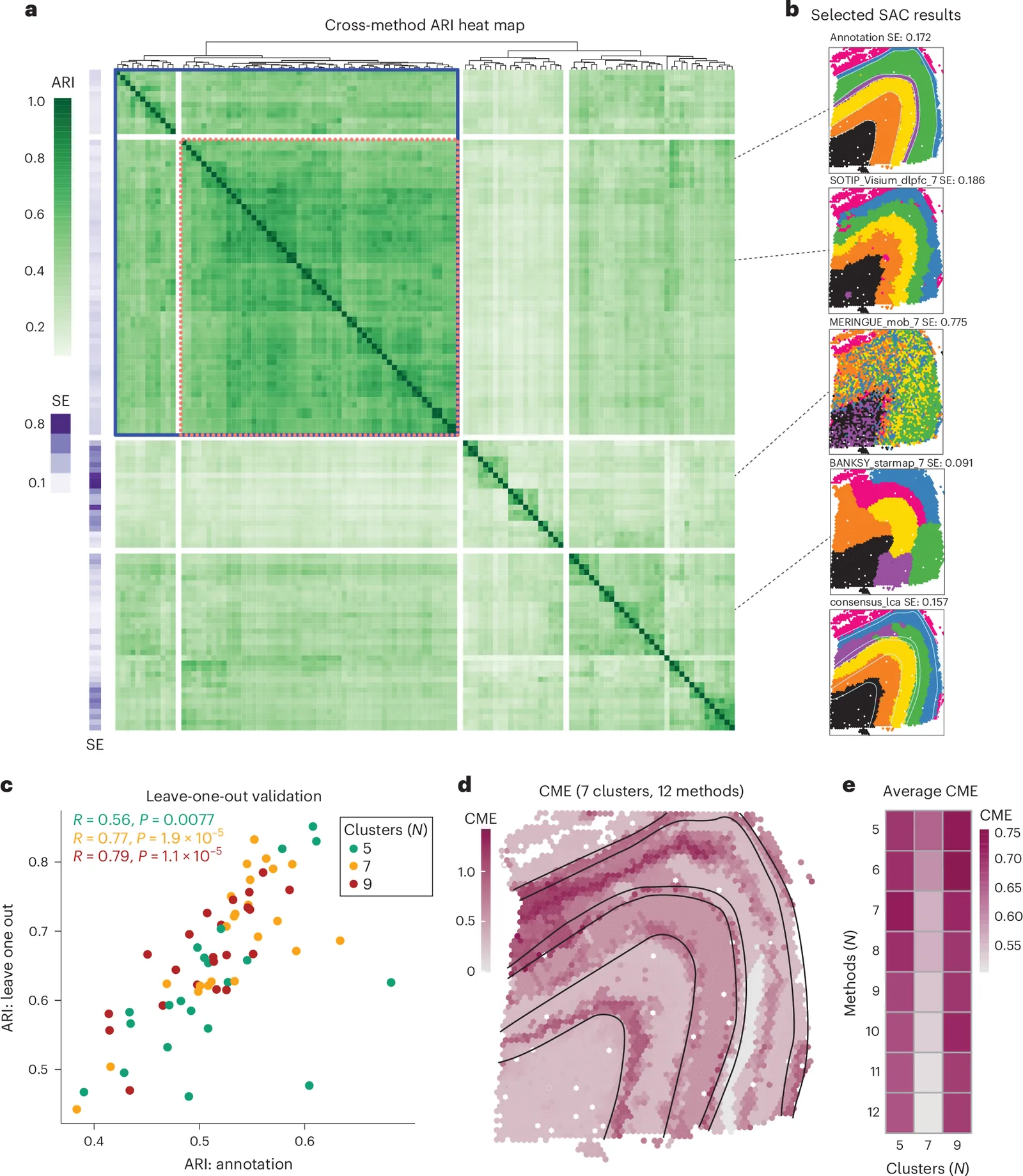
Consensus and entropy of SAC. **a**, Method-versus-method ARIs for 119 base clusterings (including the manual annotation). The row annotations show the SE (lower SE means ‘smoother’ domains or, specifically, fewer total boundaries between domains). Blue box denotes similar clustering results with an average ARI of 0.56 and pink box with 0.62. **b**, Selected base clusterings. consensus_lca denotes a consensus clustering built from 12 methods at 7 clusters (**d** and **e**; Methods). **c**, The ARI of SAC methods calculated against a leave-one-out consensus versus the ARI against the original GT annotations. Pearson’s correlation coefficients (*R*) were calculated using the default two-sided test. No multiple comparison correction was applied. **d**, CME (spot-wise similarity of cluster membership across methods) for 7 clusters and the 12 smoothest nonduplicated SAC methods. **e**, CME (averaged over all spots) for combinations of the number of smoothest methods and number of clusters.

For the Visium LIBD DLPFC dataset, base clusterings revealed a wide range of smoothness levels (Fig. 3a,b and Supplementary Fig. 14). SAC methods showed higher concordance with each other than the GT (Fig. 3b). Notably, for slice 151673 of the LIBD DLPFC dataset, we identified a block of base clusterings with method-versus-method ARIs averaging around 0.56 (Fig. 3a, blue box), and another exceeding 0.62 (Fig. 3a, pink box with dashed lines). This method concordance held across algorithms and all slices of the LIBD DLPFC dataset (Supplementary Fig. 15).

To determine whether combining outputs of high-concordance (high pairwise ARI) and spatially continuous (low SE) methods could yield biologically relevant groupings, we selected SAC methods and formed a consensus clustering using latent class analysis (LCA)^49^. LCA was chosen for its scalability to large datasets (Supplementary Fig. 16), although K-modes and weight-based consensus approaches^50–52^ were also implemented. We applied a leave-one-out strategy among preselected continuous and concordant methods (Fig. 3a, blue box). For each method, we built consensus clusterings from the remaining methods, serving as a pseudo-GT to assess whether that method’s clustering differed from others. While this is not a bona fide performance metric, clusterings matching pseudo-GTs also matched manual annotation (Fig. 3c).

Consensus clustering allows us to spatially map commonalities and uncertainties in base clusterings using cross-method entropy (CME; lower values highlight concordance across methods). We selected the 12 lowest-SE concordant-block base clusterings with seven clusters (Supplementary Fig. 17) and generated a consensus clustering for slice 151673 of the LIBD DLPFC dataset (Fig. 3b, consensus_lca). While this dataset is known to exhibit layered neocortex structures, we included base clusterings without perfect layering (Supplementary Fig. 17), but the consensus exhibited a smooth (SE, 0.17) and mostly layered cluster organization (Fig. 3b). To identify areas of disagreement, we visualized the CME across the set of base clusterings (Fig. 3d). Heterogeneous regions aligned with boundaries of both consensus clusters and anatomical labels. While none of the methods could identify cortical layer 4 (L4) (Fig. 3b), the CME plot showed that the L4 layer regions exhibited high entropy, indicating the difficulty of correctly annotating L4 in this dataset.

Furthermore, by sweeping across the number of clusters and the base clusterings, we identified that 7 clusters induce the highest correspondence between methods, and low CME remains stable even when selecting up to 12 base clusterings for the consensus (Fig. 3e). This approach can serve as a practical guide for an initial clustering of spatial data without requiring a GT (Supplementary Figs. 18–22).

### Consensus with expert in the loop

In practice, researchers iteratively test multiple SAC methods and parameters alongside expert collaboration. SACCELERATOR’s consensus clustering and parameter sweeps systematize this workflow. We demonstrate this with domain experts evaluating mouse brain thalamus^53^ and colorectal cancer^54^ datasets.

#### Consensus SAC identifies anatomical boundaries in the mouse thalamus (case study 1)

**Fig. 4:**
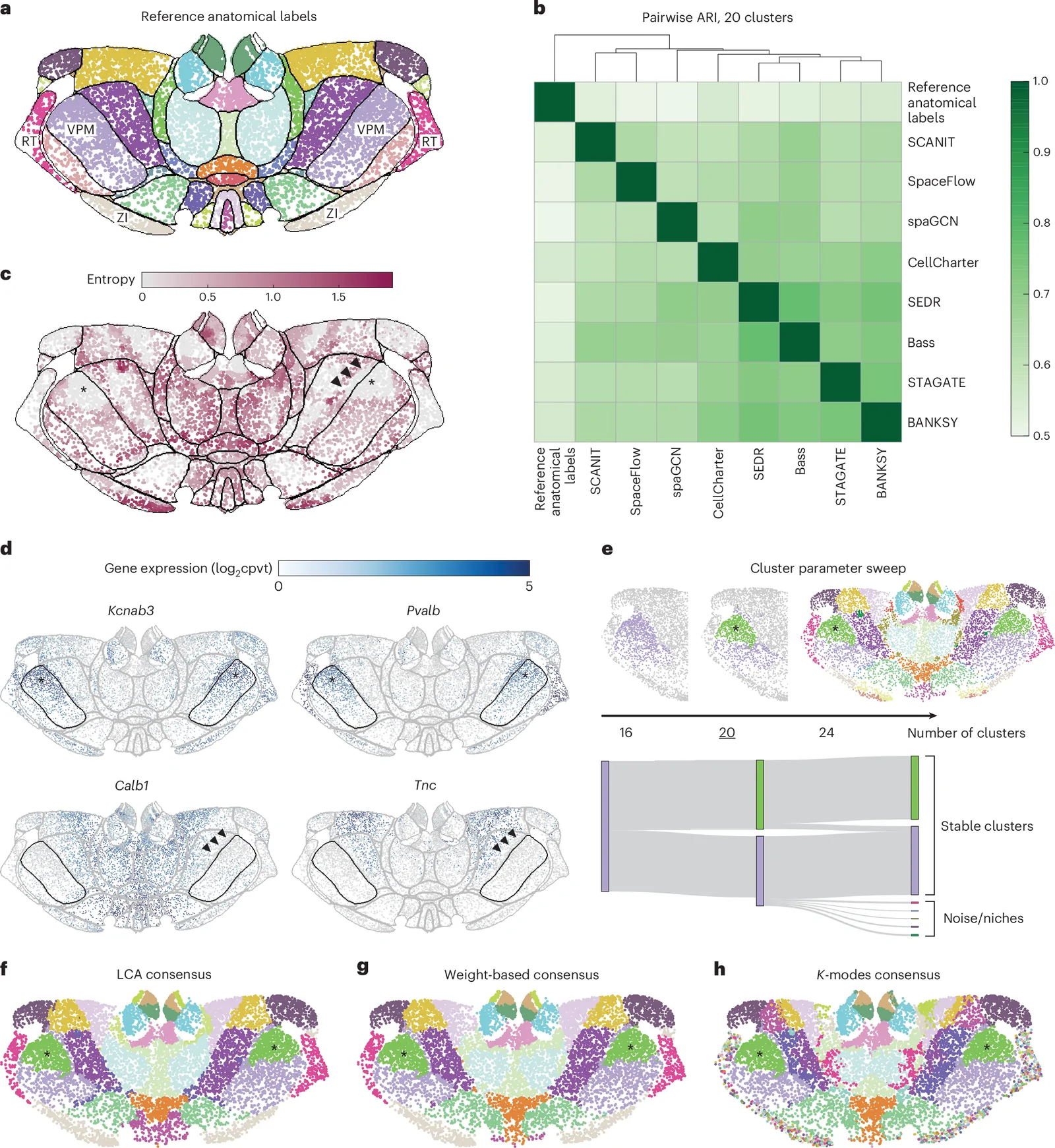
MERFISH dataset of the adult mouse brain thalamus region. **a**, Reference anatomical labels with borders indicated in black. **b**, Pairwise method-versus-method ARI comparison heat map of the eight base SAC methods and the reference anatomical labels. **c**, CME of the eight base SAC results selected to build the consensus clusters. Reference anatomical label borders are indicated in black. Asterisks mark a low-entropy subdivision of the VPM. Arrowheads mark a discrepancy between the reference anatomical borders and the SAC consensus in the right hemisphere. **d**, Gene expression of four genes (*Kcnab3*, *Pvalb*, *Calb1* and *Tnc*) that have spatially variable expression within or at the boundaries of the VPM subregion of the thalamus. The borders of all reference anatomical labels are outlined in gray; the VPM is highlighted in black. **e**, Weight-based consensus of the datasets for 16, 20 and 24 clusters, with the left VPM structure highlighted for the 16 and 20 cluster cases (top). We used 20 clusters (underlined) to generate the consensus clusterings in **f** to **h**. Sankey diagram of the VPM region with the increase in cluster number (bottom). **f**, Consensus clustering computed with LCA and the clustering parameter set to 20 clusters. **g**, Consensus clustering computed using a weight-based framework and clustering parameter set to 20 clusters. **h**, Consensus clustering computed using K-modes and the clustering parameter set to 20 clusters. Data from Yao et al.^49^, MERFISH-C57BL6J-638850 dataset.

The adult mouse brain thalamus has been parcellated into approximately 40 anatomical labels, called thalamic nuclei (Fig. 4a), on the basis of cytoarchitecture (cell shape, size and density) and histochemical stains. However, this level of granularity insufficiently captures the finer topographical patterns of neuronal connectivity and function within the thalamus^55^.

SAC methods can identify novel, functionally relevant parcellations. However, the results of eight SAC methods on the mouse thalamus MERFISH dataset were highly discordant (Supplementary Figs. 23 and 24). Without further refinement, this lack of agreement would only introduce confusion, rather than improve our understanding of molecular thalamic organization. Therefore, we leveraged consensus clustering and entropy analyses to interpret the outputs of SAC methods on this dataset.

We compared SAC results with each other and to reference anatomical labels from the Allen Mouse Brain Common Coordinate Framework (CCFv3)^56^ using pairwise ARIs (Fig. 4b). Again, SAC methods agreed more with each other than with the anatomical reference labels (Fig. 4a,b), reiterating the limitations of using anatomical annotations as GT for SAC evaluation.

Expert reviewers compared clusterings with reference anatomical labels and their thalamic biology expertise. The CME (Fig. 4c) revealed regions of high entropy (low cross-method agreement) near the boundaries between anatomical labels. Two key insights emerged. First, boundaries between thalamic nuclei often correspond to transitions in gene expression, and the entropy plot visually highlights how small differences in method or parameterization lead to variation in SAC calls at these transitions. Second, the entropy-indicated border identifies regions where the reference anatomical labels could be refined. While reference anatomical labels were derived from spatial registration to a whole-brain coordinate space (CCFv3), this process imperfectly results in misalignments, most notably at borders between anatomical regions. The ventral posteromedial nucleus (VPM) shows key marker gene expression aligning better with entropy-indicated borders than reference labels (Fig. 4c,d, arrowheads).

Next, we generated consensus clusterings for the thalamus (Fig. 4e–h). By sweeping the number of clusters from 16 to 24 (Fig. 4e), size and continuity stabilized at around 20 clusters, while higher cluster numbers yielded small and noisy clusters. We then generated consensus clusterings using LCA, weight-based and K-modes methods, each set to 20 clusters (Fig. 4f–h). Expert evaluation revealed distinct behaviors across aggregation approaches. K-modes consensus subdivided many anatomical labels and introduced numerous salt-and-pepper (noncontiguous) clusters across the zona incerta (ZI) and reticular thalamic nucleus (RT). This is consistent with transcriptional diversity and spatial structure within these areas^57,58^. By contrast, weight-based and LCA consensus found one cluster each for ZI and RT.

Interestingly, some anatomical regions were consistently subdivided by all three consensus methods. For example, the VPM split into two clusters (Fig. 4f–h, asterisks), reflecting the multigene expression gradients in the SRT data (Fig. 4d, asterisks). The genes shown were chosen on the basis of differential expression between the cells in the two VPM clusters and may correspond to the spatial organization of neurons on the basis of their axonal projections^59^. Given the high interest in identifying novel molecular parcellations of the thalamus, this robust consensus approach, combined with reassurance that we can find a stable number of clusters, is a promising target for experimental investigation with additional molecular and multimodal measurements.

#### Detecting neoplastic progression stages in colorectal cancer (case study 2)

To evaluate our approach in a setting lacking established anatomical reference labels, we analyzed a previously unannotated SRT dataset of a pretreatment human colon adenocarcinoma profiled using Visium HD^54^. We engaged a histologist and a pathologist specializing in colorectal cancer, who provided iterative feedback throughout the analysis process. This use case reveals another aspect of cluster annotation: a pathologist may not be equally interested in all regions, instead focusing their attention on pathophysiologically distinct areas.

**Fig. 5:**
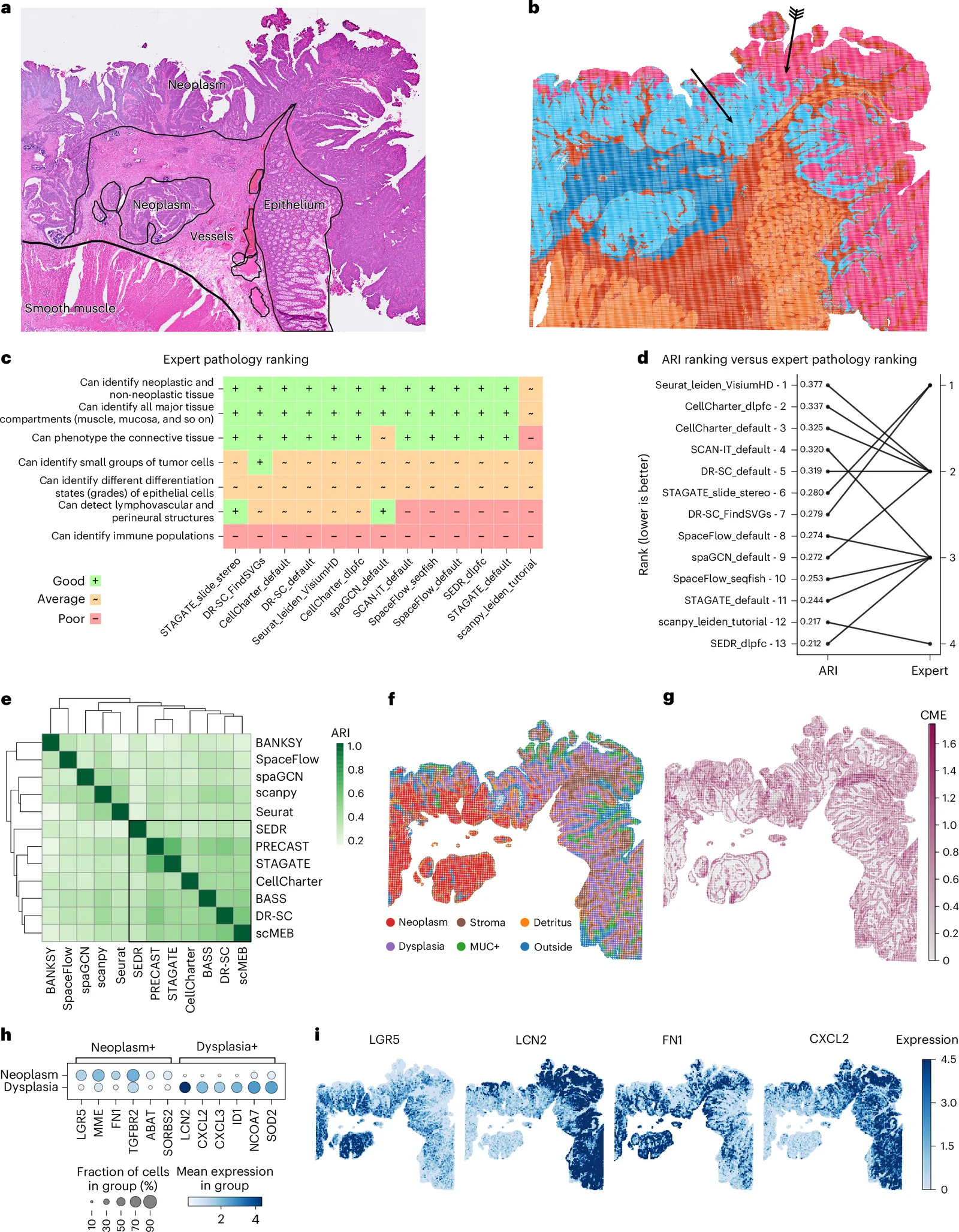
Colorectal cancer Visium HD consensus with expert in the loop. **a**, Initial annotation of tissue regions on the basis of the H&E image by an expert histologist. **b**, SEDR clustering (six clusters) identifies two distinct clusters in the dysplastic epithelium (feathered and smooth arrow). **c**, Expert ranking of SAC method performance for spatial feature identification relevant for pathological assessment (nine clusters; full result shown in Supplementary Fig. 26). **d**, Comparison of the ARI calculated on the basis of annotations (**a**) versus the expert ranking (**c**) for each SAC method. **e**, Method-versus-method ARI comparison heat map for the neoplasm area. The boxed methods were identified as a concordant cluster on the basis of Euclidean distance. **f**, Consensus clustering computed using K-modes for the seven methods highlighted in **e**. **g**, CME map of the consensus clustering. **h**, Top differentially expressed genes between neoplasm and dysplasia. Color-coded by log1p-normalized gene expression and size-coded by the percentage of bins expressing the gene in the clusters. **i**, Spatial expression patterns of selected genes. Expression in log1p-normalized counts.

Initially, the histologist annotated the tissue sections on the basis of a high-resolution H&E image. The neoplasm (tumor tissue) boundary was refined using *EPCAM* expression (Supplementary Fig. 25), consistent with established FISH workflows (Fig. 5a). These annotations served as a baseline for evaluating SAC methods. We observed a clear correspondence between expert annotation and SAC results (Supplementary Fig. 26), with a consistent difference being the split neoplasm into two distinct clusters (Fig. 5b). Before analyzing ARI scores, the two experts independently ranked each algorithm on the basis of the detection of relevant tissue features: (1) distinguishing cancer from noncancer tissue, (2) identifying neoplastic differentiation, (3) differentiating between all colon tissue compartments, (4) identifying lymphovascular and perineural structures, (5) detecting small groups of neoplastic cells, (6) identifying immune cells and (7) phenotyping of connective tissue (Fig. 5c). Interestingly, this expert ranking differed substantially from the ARI scores calculated against the GT labels defined by the experts themselves (Fig. 5d). DR-SC and STAGATE received the highest expert score while performing average in ARI, whereas the best-performing methods in ARI, Seurat and CellCharter, did not reach the top expert rank (Fig. 5d). This lack of concordance between expert versus metric evaluation was also evident when evaluating the NMI, SE and CHAOS scores (Supplementary Fig. 27). This is consistent with previous reports highlighting the limitations of global metrics, such as ARI, in evaluating performance for small, biologically relevant structures^60^.

Further investigation of nuclear morphology in the two distinct neoplasm clusters (Fig. 5b) indicated a possible transition between neoplastic and dysplastic tissues, characterized by an increased nuclear-to-cytoplasmic ratio and nuclear crowding^61^. Annotation of these areas is usually carried out on the basis of morphology^62^, while more fine-grained molecular assessment of neoplastic cells has only recently emerged^63^. Thus, this subclustering of the neoplasm represents different scales of biologically relevant features that can be missed when rigidly evaluating SAC methods against manually annotated GTs.

To assess whether SAC methods could assist with this task, we re-applied available methods (using default configurations) to the region originally annotated as neoplasm by the experts. We formed a consensus from methods showing a high concordance in terms of pairwise ARIs (Fig. 5e), reasoning that this could pinpoint pathological structures of interest (Supplementary Fig. 28). The agreement between methods was generally high, as indicated by low entropy, except at the boundary between the tumor stroma and healthy epithelium (Fig. 5g). This might be caused by transitional zones, similar to observations in case study 1. The six distinct regions identified included (1) separate tumor and (2) dysplastic zones, (3) mucin signaling hot spots (glycoprotein-rich areas linked to specific colorectal cancer phenotypes^64^), (4) tumor stroma, (5) cellular detritus split off from the tumor and (6) an external region (probably diffusion artifacts) outside the tissue (Fig. 5f).

We found well-studied differentially expressed genes between neoplastic and dysplastic areas (Fig. 5h,i). The neoplasm showed high expression of *LGR5* (stem cell marker, investigated as a therapeutic target in CRC)^65^, *FN1* (extracellular matrix remodeling)^66^ and *TGFBR2* (conditional tumor suppressor/activator as part of TGF-β signaling)^67^. Conversely, the suspected dysplasia expressed *CXCL2/3* (inflammatory markers)^68^, *LCN2* (a secretory protein potentially acting as a tumor suppressor in colorectal cancer)^69^ and *SOD2*^70^ and *NCOA7*^71^ (stress response genes). Collectively, the gene expression patterns support the transition from a premalignant, inflammatory and stressed dysplasia to a proliferative neoplasm with pro-invasive traits.

Our findings illustrate the value of an expert-in-the-loop approach where data-driven clusters combined with domain knowledge guides method selection and analysis direction. Notably, the experts did not initially set out to investigate the dysplasia-to-neoplasia transition, underscoring the iterative and exploratory nature of investigating tissue architecture. This experience suggests that defining a single GT for spatial regions is often impractical, especially in complex tissues where biological heterogeneity and functional gradients are expected.

## Discussion

Existing SAC benchmarks focused narrowly on older SRT technologies and brain datasets, with questionable GT annotations. Alarmingly, most SAC methods fail on large, modern datasets, while meta-analysis revealed considerable ARI inconsistencies between studies, driven by parameter sensitivity, preprocessing differences, unreliable GTs and technology-specific effects.

Therefore, we developed SACCELERATOR, a consensus clustering framework that better reflects realistic research workflows. By integrating method agreement with spatial entropy maps, SACCELERATOR enables targeted feedback for tissue (Supplementary Discussion, Supplementary File 1 and Supplementary Fig. 29).

GT-based metrics fail in complex tissues. Consensus clustering instead identifies reproducible domains and disagreement hot spots. Our case studies showed high entropy highlighting biologically meaningful gradients, such as dysplastic-to-neoplastic transitions in colorectal cancer, and robust subdivisions within anatomical regions, such as the VPM nucleus in the thalamus^59^. Framework-wide granularity sweeps in the Xenium mouse brain dataset confirmed anatomical correspondence at intermediate cluster numbers, with extremes either under- or overpartitioning.

Unlike prior SAC consensus methods (STCC and EnSDD)^50,72^, optimized for GT alignment that failed to scale beyond Visium datasets, SACCELERATOR uses consensus as an exploratory guide. Divergence from consensus provides method developers feedback on unique strengths and weaknesses. Users should balance algorithmic and methodological base-clustering diversity with computational feasibility for exploratory analyses lacking GT priors.

In our work, we rely on several metrics, such as ARI and NMI; however, this is only to provide familiar references, rather than primary validation. Metric correlation analyses showed similarity and complementarity, with general-purpose metrics (ARI and NMI) clustering separately from application-specific spatial metrics (CHAOS and PAS) and internal validation metrics (Davies–Bouldin index and Calinski–Harabasz index). Overall, ARI and SE were found to be generally representative of the other metrics in most scenarios. Furthermore, expert evaluation remains essential, though subjective, for biological interpretation, and is best used alongside quantitative metrics (Supplementary Discussion).

While focused on SAC, SACCELERATOR provides a generic framework for reproducible evaluation of genomics tools, bridging computational analysis with collaborative, iterative biological interpretation.

## Methods

### Datasets

We evaluated SAC methods across 15 published datasets representing both newer and older SRT technologies. Datasets were categorized on the basis of their spatial resolution: mini-bulk, single-cell and subcellular (Supplementary Table 1). Only datasets with available GT annotations were considered for inclusion. To ensure biological relevance and technical diversity, we prioritized nonbrain tissue datasets with subcellular spatial resolution.

The spatially resolved gene expression data, GT annotations and further metadata were downloaded from original sources and converted to a standardized TSV/JSON format using our Snakemake pipeline. As the datasets used in this study originate from publicly available sources that have already undergone careful curation by their respective authors, we applied minimal filtering. Specifically, we removed cells and bins with no gene expression, removed genes that were not expressed in any cell and bins and excluded cells in which mitochondrial transcripts accounted for more than 30% of total transcripts. For datasets generated using mini-bulk technologies, we filtered out bins containing fewer than ten cells when this information was available. Furthermore, if the metadata contained bin-wise information of whether the bins overlapped with the tissue region, we excluded those outside the tissue area. No additional preprocessing was applied. Quality-filtered raw data were directly input into each method, following the method implementation or vignette without modification. Supplementary Table 1 presents an overview of the analyzed datasets.

#### CosMx human liver dataset (cosmx_liver)

The CosMx human liver dataset was obtained from the NanoString website (https://nanostring.com/products/cosmx-spatial-molecular-imager/ffpe-dataset/human-liver-rna-ffpe-dataset). The dataset consists of two formalin-fixed paraffin-embedded (FFPE) samples from two patients, one being normal liver and the other from a patient with grade G3 hepatocellular carcinoma. Data were generated on the CosMx platform using the Human Universal Cell Characterization Panel 1000 plex. The GT annotations were computationally identified using Mclust clustering on the frequency of each cell type among its 200 nearest neighbors.

#### CosMx human non-small-cell lung cancer dataset (cosmx_lung)

The CosMx human non-small-cell lung cancer dataset was obtained from the NanoString website (https://staging.nanostring.com/products/cosmx-spatial-molecular-imager/ffpe-dataset/nsclc-ffpe-dataset). The dataset consists of eight FFPE samples from five patients presenting with non-small-cell lung cancer grade G1–G3. Data were generated on a CosMx prototype instrument using a 960-gene panel^73^. The GT annotations were computationally identified using Mclust clustering on the frequency of each cell type among its 200 nearest neighbors.

#### MERFISH mouse brain thalamus (abc_atlas_wmb_thalamus)

The MERFISH mouse brain thalamus dataset^53^ was obtained from the Brain Knowledge Platform (https://portal.brain-map.org/atlases-and-data/bkp/abc-atlas; https://alleninstitute.github.io/abc_atlas_access/descriptions/MERFISH-C57BL6J-638850.html). The dataset consists of 59 fresh frozen (FF) serial full coronal sections at 200-μm intervals spanning one entire mouse brain. Data were generated on a Vizgen MERSCOPE instrument using a custom gene panel of 500 genes. The GT annotations were identified by aligning the MERFISH data to the CCFv3 coordinate space and labeling cells with the corresponding CCFv3 anatomical parcellation term^56^. Only the thalamus (CCFv3 structure ID 549) and hypothalamic ZI (CCFv3 structure ID 797) were analyzed in this study. Spatially variable genes in the thalamus were identified by differential gene expression analysis on neighboring consensus clusters.

#### MERFISH human developmental heart dataset (merfish_devheart)

The MERFISH human developmental heart dataset^74^ was obtained from Dryad^75^. The dataset consists of four FF samples from two donors at 13 and 15 post-conception weeks. Data were generated using MERFISH with a custom 238-gene panel. The GT annotations (referred to as cellular communities in the original study) were computationally identified using *k*-means clustering of relative cell-type composition within 150 μm of each cell.

#### STARmap PLUS mouse brain dataset (STARmap_plus)

The STARmap PLUS mouse brain dataset^76^ was obtained from Zenodo^77^. The dataset consists of 20 FF samples from three mice. Data were generated using STARmap PLUS using a custom 1,022-gene panel. The GT annotations were manually identified by aligning the data to the CCFv3.

#### Xenium human breast cancer dataset (xenium-ffpe-bc-idc)

The Xenium breast cancer dataset was obtained from the 10x Genomics website (https://www.10xgenomics.com/datasets/xenium-ffpe-human-breast-with-custom-add-on-panel-1-standard). The dataset consists of one FFPE sample from a patient with infiltrating ductal carcinoma breast cancer. Data were generated on a Xenium Analyzer using the Xenium human breast gene expression panel v1 (280 genes) with 100 additional custom genes. The GT annotation was manually identified using the matched histopathology image, annotating for eight region types: ductal carcinoma in situ, invasive tumor, normal ducts, immune cells, cysts, blood vessels, adipose tissue and stroma^78^.

#### Xenium mouse brain dataset (xenium-mouse-brain-SergioSalas)

The Xenium mouse brain dataset was obtained from the 10x Genomics website (https://www.10xgenomics.com/datasets/fresh-frozen-mouse-brain-replicates-1-standard). The dataset consists of one FF sample of a full coronal section. Data were generated on a Xenium Analyzer using the v1 mouse brain gene expression panel (247 genes). The GT annotation was manually identified using the mouse coronal P56 sample from Allen Brain Atlas^56^ to specify anatomical regions^79^.

#### Slide-seqV2 mouse brain olfactory bulb dataset (slideseq2_olfactory_bulb)

The Slide-seqV2 mouse brain olfactory bulb dataset^80^ was obtained from the STOmicsDB website (https://db.cngb.org/stomics/datasets/STDS0000172). The dataset consists of 20 samples of a mouse olfactory bulb evenly spaced along the anterior–posterior axis. Data were generated using Slide-seqV2 and sequenced using paired-end reads on an Illumina NovaSeq 6000 instrument, targeting 200 million reads per sample. The GT annotations were manually identified on the basis of the expression of marker genes.

#### Stereo-seq mouse liver dataset (stereoseq_liver)

The Stereo-seq mouse liver dataset^44^ was obtained from the STOmicsDB website (https://db.cngb.org/stomics/datasets/STDS0000059). The dataset consists of six FF samples. Data were generated on Stereo-seq chips and sequenced using paired-end reads on a DIPSEQ T1 instrument. The GT annotations were computationally identified where zonation layers were annotated on the basis of the differences between the scores of pericentral and periportal hepatocyte landmark genes.

#### Stereo-seq mouse embryo dataset (stereoseq_mouse_embryo)

The Stereo-seq mouse embryo dataset^13^ was obtained from the STOmicsDB website (https://db.cngb.org/stomics/datasets/STDS0000058). The dataset consists of 53 FF samples from mouse embryos spanning E9.5–E16.5 with 1-day intervals. Data were generated on Stereo-seq chips and sequenced using paired-end reads on a MGI DNBSEQ-Tx sequencer. The GT annotations were computationally identified using spatially constrained clustering, which is built on top of the Leiden clustering algorithm^13^.

#### Visium human brain LIBD DLPFC dataset 1 (libd_dlpfc)

The Visium human brain LIBD DLPFC dataset 1 (ref. ^33^) was obtained from the spatialLIBD Bioconductor package (https://research.libd.org/spatialLIBD). The dataset consists of 12 FF samples from three donors. The data were generated on Visium chips and sequenced using paired-end reads on an Illumina NovaSeq 6000 instrument. The GT annotations were manually identified on the basis of cytoarchitecture and selected gene markers.

#### osmFISH mouse brain somatosensory cortex dataset (osmfish_Ssp)

The osmFISH mouse brain somatosensory cortex dataset^81^ was obtained from the Linnarsson Lab website (https://linnarssonlab.org/osmFISH). The dataset consists of a single FF sample from the mouse brain somatosensory cortex. Data were generated using osmFISH using a custom 33-gene panel. The GT annotation was computationally identified using an iterative graph-based algorithm.

#### Visium human breast cancer (visium_breast_cancer_SEDR)

The Visium human breast cancer dataset, originally from 10x Genomics (https://www.10xgenomics.com/resources/datasets/human-breast-cancer-block-a-section-1-1-standard-1-1-0), was obtained from GitHub (https://github.com/JinmiaoChenLab/SEDR_analyses)^82^. The dataset consists of a single FF sample of invasive ductal carcinoma breast tissue. The data were generated on a Visium chip and sequenced using paired-end reads on an Illumina NovaSeq 6000 instrument. The GT annotation was manually identified on the basis of the H&E image.

#### Visium chicken heart (visium_chicken_heart)

The Visium chicken heart dataset^83^ was obtained from GEO (https://www.ncbi.nlm.nih.gov/geo/query/acc.cgi?acc=GSE149457). The dataset consists of 11 FF samples from four hearts at different stages of ventricular development. The data were generated on a Visium chip and sequenced using paired-end reads on an Illumina NextSeq 500/550 instrument. The GT annotations were computationally identified using Louvain clustering as implemented in Seurat v3.

#### Visium HD human colorectal cancer dataset (visium_hd_cancer_colon)

The Visium HD human colorectal cancer dataset^59^ was obtained from the 10x Genomics website (https://www.10xgenomics.com/datasets/visium-hd-cytassist-gene-expression-libraries-of-human-crc). The dataset consists of one FFPE sample of colorectal cancer obtained from the sigmoid colon of a patient. Data were generated on a Visium HD chip and sequenced using paired-end reads on an Illumina NovaSeq 6000 instrument.

### GT annotation of the Visium HD human colorectal cancer dataset

At the time of analysis, no GT annotation was available for the Visium HD human colorectal cancer dataset. After data download, annotation of the Visium HD colorectal cancer histology sections was performed on H&E-stained whole-slide images by a trained histologist. Regions of smooth muscle, vessels, normal epithelium and neoplastic epithelium were manually annotated, followed by review by a pathologist. Smooth muscle was identified by its characteristic pink eosinophilic staining in organized bundles, with elongated, centrally located nuclei. Blood vessel regions were annotated on the basis of the presence of luminal structures, filled with erythrocytes and lined by flat cells (endothelium). Normal epithelium was identified by recognizing well-organized crypt architecture, composed of columnar epithelial cells with basally oriented nuclei, low nuclear-to-cytoplasmic ratios and regular cell polarity. Neoplastic epithelium was annotated by identifying regions with loss of normal crypt architecture, increased nuclear pleomorphism, hyperchromasia, irregular nuclear contours, aberrant nuclear-to-cytoplasmic ratios and loss of polarity. Stroma or connective tissue was identified by its bright eosinophilic staining, low cellular density, presence of spindle-shaped fibroblast-like cells and visible connective tissue fibers (for example, collagen). Rare interspersed immune cells, such as neutrophils, lymphocytes and others, could occasionally be observed. Stroma was typically located adjacent to epithelial or neoplastic areas. Dense accumulation of lymphoid cells, including lymphocytes and histiocytes, would represent lymphoid structures. However, such structures were not observed in the present colorectal cancer case. Dissociated cell material indicative of detritus or necrosis was identified by the presence of cellular debris, often accompanied by infiltration of neutrophilic granulocytes. Annotations were further refined by the expression of the characteristic marker genes *EPCAM* (epithelial neoplasm), *VWF* (vessels) and *MYL9* (smooth muscle). The annotations were attached to individual Visium HD bins by annotation in SpatialData^84^ with the help of the Napari interface^85^.

### SAC methods

We implemented all 22 SAC methods according to their original publications and official documentation. To ensure fidelity to method designs, preprocessing steps (for example, normalization and feature selection) were replicated exactly as described in method vignettes or GitHub repositories. For parameter configurations, we included a ‘default’ configuration, where we used author-predefined parameter values, with undefined parameters set via prominent tutorials or GitHub READMEs. Additional configurations were limited to those available in the method documentation, where parameters were set as specified in case-specific vignettes. For added provenance tracking, the configuration files include a reference to their sources.

Graph-based SAC methods often use resolution parameters to control cluster granularity that ultimately dictates the number of resultant clusters. To enable fair comparison across metrics requiring fixed cluster counts, we performed binary searches for the resolution values yielding the number of clusters defined in the GT annotation. This approach ensured compatibility with spatially agnostic metrics such as ARI while maintaining method-specific spatial constraints.

In addition, we included the most prominent single-cell transcriptomics clustering algorithm, Leiden, as implemented in Seurat and Scanpy. See Supplementary Table 2 for a complete overview of the implemented methods.

### Metrics

We implemented 17 metrics in the framework, categorized based on their use of GT labels or spatial information. See Supplementary Table 4 for a complete overview of the implemented metrics. In addition to the conventional clustering metrics, we developed two entropy-based metrics. Both metrics require the calculation of Shannon entropy. In our implementation, if we have a vector of cells from different classes (that is, clusters), the entropy can subsequently be calculated as

$$H({\bf{X}})=-\sum _{i={1}}^{m}\left(\displaystyle\frac{{n}_{i}}{{\sum }_{j={1}}^{m}{n}_{j}}\times {\mathrm{log}}\left(\displaystyle\frac{{n}_{i}}{{\sum }_{j={1}}^{m}{n}_{j}}\right)\right)$$

where *m* is the number of classes, *n*_*i*_ is the occurrence of class *i* in vector *X* and ${n}_{i}/\mathop{\sum }\nolimits_{j = 1}^{m}{n}_{j}$ is the empirical probability of class *i*.

#### SE

SE quantifies the spatial coherence of clustering results by measuring label consistency within local neighborhoods. The SE is calculated by identifying the *k*-nearest neighbors for each spot or cell in the dataset. Subsequently, the entropy is calculated spot-wise across the labels of the spot and its *k* neighbors. Spot-wise entropy is then averaged to provide the SE for the sample on a particular label. For SE shown in Fig. 3, *k* is set to 6.

#### CME

CME identifies regions of uncertainty by measuring label discordance across multiple clustering results. To generate the CME for a dataset, a set of labelings with the same number of clusters is aligned by finding the optimal matches across different labelings using the Hungarian method implemented in the R package clue (version 0.3_66)^86^. Once the labelings are aligned, spot-wise entropy is calculated across the labelings from different methods. The labeling alignment requires a reference labeling that other labelings are aligned against. To avoid stochasticity from arbitrary selection of reference labeling, the entropy is calculated once for each instance where a labeling is selected as the reference. The final spot-wise CME is calculated by averaging the entropies from different reference labeling cases.

### Computational framework

SACCELERATOR is orchestrated by a modular Snakemake^87^ workflow, which coordinates all computational steps for standardized and reproducible analysis. The framework is organized into distinct modules for datasets, SAC methods, metrics and consensus clustering. Each dataset module consisted of a script to download the dataset, GT annotations, images and other metadata and store them in a standardized format. Likewise, each method and metric module consisted of a separate script, ensuring interoperability with the standardized data structure produced by other modules. Consensus clustering within SACCELERATOR is implemented as a three-step process: results aggregation, base clustering selection and consensus generation. Each of these steps is documented with a minimally executable example, clear instructions to add new instances and explanatory notes in its respective folder in the GitHub repository and the documentation (https://spatialhackathon.github.io/SACCELERATOR). To guarantee reproducibility and prevent dependency conflicts, each script is linked to a version-pinned conda environment file. A centralized configuration file allows the Snakemake workflow to specify parameters for each module, such as which datasets to download and the number of clusters to identify.

### Consensus clustering algorithms

Three consensus clustering approaches were implemented in the framework and applied in the paper: K-modes, LCA and weight-based consensus. While many consensus algorithms exist, these three were selected as representative examples to demonstrate the flexibility of the consensus workflow.

K-modes was selected as the categorical analog of *k*-means. In the context of consensus clustering, each base clustering contributes to the consensus clustering equally, and their labels serve merely as nominal categories without ordering or magnitude, which aligns with the assumption of the K-modes method. As an algorithmic approach to consensus clustering, K-modes aims to find clustering partitions that minimize the overall hamming distance to cluster-specific mode vector^88^. This approach returns interpretable results in a scalable manner, especially for big datasets (Supplementary Fig. 16). In the SACCELERATOR framework, K-modes is implemented via the R package diceR^89^.

LCA was chosen as a representative model-based consensus clustering approach. LCA assumes that there exists an unobserved categorical latent class (the consensus clustering) that explains the observed base clusterings and that individual base clustering results are conditionally independent given the latent class. Unlike the distance-based K-modes, LCA is a probabilistic approach that enables flexible handling of base clustering disagreement by method-specific conditional probabilities^90^. In the SACCELERATOR framework, LCA is implemented via the R package diceR^89^.

In addition, we also implemented a weighted-based consensus on the basis of a Bregmannian divergence framework^50,52^ utilizing the weighted Jensen–Shannon divergence as the loss function^91^

$$\mathop{\min }\limits_{S,\{{\omega }_{m}\}}\sum _{m=1}^{M}{\omega }_{m}\,\mathrm{JSD}(S\parallel {S}^{(m)})+\lambda \sum _{m=1}^{M}{\omega }_{m}\mathrm{log}{\omega }_{m},$$

subject to

$$\sum _{m=1}^{M}{\omega }_{m}=1,\quad {\omega }_{m}\ge 0.$$

where *M* is the number of base clusterings, *S* is the binary similarity matrix for consensus results, *S*^(*m*)^ is the binary similarity matrix of individual base clusterings and *ω*_*m*_ are the respective weights of each base clustering. The second term is an entropy regularization of the weights to prevent the inclination of the binary similarity matrix to any single base clusterings. The resulting binary similarity matrix *S* was clustered using the Leiden algorithm to generate the final consensus clusters. Unlike LCA and K-modes, the weighted consensus also returns the weight terms assigned to each base clustering, providing an interpretable understanding of the relative contributions of different methods. However, the incorporation of large binary matrices limits the scalability of this method (Supplementary Fig. 16).

Although the three approaches (K-modes, LCA and weighted consensus) are already integrated into the workflow, we encourage users to explore additional consensus algorithms as needed, such as majority voting or linkage clustering ensemble^92^. The choice of method should be guided by both computational considerations (for example, scalability; Supplementary Fig. 16) and the specific biological questions of interest, ideally in consultation with domain experts (see Supplementary Fig. 29 for an overview of the expert-guided workflow instruction).

The cluster scalability results were generated using the benchmark feature of Snakemake. The consensus part of the pipeline was applied to all existing clustering results of datasets with the following names: xenium-mouse-brain-SergioSalas, abc_atlas_wmb_thalamus, libd_dlpfc, visium_hd_cancer_colon, cosmx_lung, STARmap_plus and visium_breast_cancer_SEDR. Results of those consensus results can be found on Zenodo (see ‘Data availability’ and ‘Code availability’ sections).

### Base clustering selection

Base clustering selection is an essential process for generating consensus clusterings. As implemented in SACCELERATOR, this process is designed to be flexible and context dependent, reflecting the expert-in-the-loop workflow. There is no universally optimal criterion for selecting the ‘best’ base clusterings for consensus clustering; rather, selection should be tailored to the specific technological platform and biological context under study. In the analysis-specific sections below, we describe in detail how base clusterings are selected on the basis of different biological settings.

### Meta-benchmark analysis

We collated ARI values for the Visium LIBD DLPFC dataset from original method papers and benchmarking studies, sourcing data from the main text and supplementary material of published articles, GitHub repositories and Zenodo repositories. In total, we gathered results for 22 SAC methods that we implemented and three benchmarking studies^3,14,15^. Among these, 17 method papers and one benchmarking study^14^ reported ARI values for the DLPFC dataset. BayesSpace, one of the earliest SAC methods, was the most frequently benchmarked, with ARI reported in 16 studies. We excluded studies that only reported summary statistics (for example, mean or median ARI) or results from a single tissue slice. For comparisons of self-reported ARI with those from other studies, we excluded methods that had not been independently applied by at least one subsequent study.

### Computational resource management

All SACCELERATOR computational jobs were orchestrated using Snakemake (v8.14.0) for workflow management and SLURM (v21.08.5) for workload management on the de.NBI cloud infrastructure. Each method was executed using a dedicated allocation of ten CPU threads and 500 GB of RAM per job. For each sample, the maximum allowed runtime was set to 48 h. If a job exceeded this limit, it was automatically terminated and recorded as a failure for that sample. Resource utilization, including wall-clock runtime and peak memory usage, was monitored and recorded using Snakemake’s built-in benchmarking functionality. Benchmarking data were parsed and summarized using custom Python scripts.

### Configuration benchmarking

For the Visium DLPFC dataset, we calculated the ARI values for all methods using the same number of clusters and method-specific configurations. Methods without additional configurations were excluded. This resulted in ARI values for 18 methods, each with two to six configurations. For details on the configuration settings, see the ‘SAC methods’ section.

### GT granularity analysis

To generate the principle component analysis (PCA) plots for the LIBD DLPFC dataset shown in Fig. 2a,b, we performed quality control on slice 151673 by filtering out spots with less than 600 total mRNA counts or more than 28% mitochondrial transcript content. Spots with fewer than ten cells detected were also excluded. The 10% most highly variable genes were detected for the sample via getTopHVGs() function in R package scran (version 1.34.0)^93^. PCA was run on the highly variable genes via function runPCA() in the R package scater (version 1.34.1)^94^. To identify border spots, we constructed a *k*-nearest neighbor graph on the basis of the spatial coordinates of the spots using the function kNN() from the R package dbscan (version 1.2.0)^95^ with *k* = 4. We filtered out neighbors with a distance larger than 1.5. A spot was deemed a border spot if any of its neighbors, including itself, did not belong to the same cluster as the GT labels.

For the Xenium mouse brain dataset and the Visium breast cancer dataset shown in Fig. 2, SAC results were generated using the default configurations of five methods (Supplementary Table 3). To examine the relationship between ARI and cluster granularity, all methods were configured to identify various numbers of clusters for the datasets (Xenium: 5, 7, 9 and 11; Visium: 3–7). The ARI against the GT annotation was then calculated using the implemented workflow.

The granularity scanning for the Xenium mouse brain dataset (Supplementary Fig. 9) is conducted using the default configuration of CellCharter in the workflow while setting cluster numbers from 5 to 49. Results of the cluster sweeping have been uploaded to Zenodo (see the ‘Data availability’ and Code availability’ sections), and additional visualization of the sweeping results can be found in Supplementary File 2.

For the Stereo-seq mouse liver dataset, we subsetted the datasets to the 925 unique zonation-related genes reported in three studies^46–48^. Five methods were then run on both the full and subsetted liver dataset with a memory and runtime cap of 500 GB and 48 h. All methods except STAGATE managed to generate clustering results. The four remaining methods were configured to generate between three and nine clusters. In addition to the default nine-layer GT, we aggregated the layers into three regions according to the original study. Specifically, layers 1 and 2 were aggregated as the pericentral cluster, layers 4–6 were merged into a midzonal cluster and the rest into the periportal cluster. ARI values were calculated for the SAC results against both the original nine-layer and aggregated three-region GTs.

### LIBD DLPFC Visium consensus analysis

All base clusterings generated from the workflow for slice 151673 (with 5, 7 or 9 clusters; Supplementary Table 3) were aggregated to compute pairwise ARIs (Fig. 3a). To identify a concordant block of SAC methods, we applied cutoffs to the hierarchical clustering tree (Euclidean distance and complete linkage) of the pairwise ARIs, which revealed two groupings, one of which represented a highly concordant set of 14 distinct clustering methods (Fig. 3a; blue box). From this set of concordant methods, base clusterings were selected as input to LCA consensus clustering^49^, separately for each number of clusters (5, 7 and 9), according to their SE, with priority given to smoother base clusterings. For example, Fig. 3d (‘7 clusters, 12 methods’) shows a consensus clustering of the 12 smoothest (that is, lowest SE) seven-cluster base clusterings. In some cases, we have multiple clusterings from the same algorithm (different parameter configurations) with the same number of clusters, to build the consensus (as described above); however, we enforce algorithm diversity by only keeping one base clustering (the smoothest) per algorithm. For the leave-one-out validation, a consensus clustering (LCA) was generated for each method, separately for each number of clusters. Each consensus comprised the six smoothest base clusterings of the starting set of methods (Fig. 3a, blue box), excluding the method itself.

### Thalamus consensus analysis

Thalamus sample C57BL6J-638850_6800 was selected to showcase the consensus workflow. We first generated SAC results of the sample from existing method configurations in the workflow (Supplementary Table 3). Then, we filtered out any base clusterings with a prominent class imbalance, where one cluster comprised more than 90% of the cells. Next, the eight methods with the lowest SE were selected as base clusterings for the consensus clustering for all three consensus algorithms. A consensus was calculated separately for base clusterings with 16, 20 and 24 clusters. The cluster numbers were selected on the basis of expert suggestions.

### Visium HD consensus analysis

The dataset was prepared as described in the ‘Datasets’ section above. Multiple configurations were used for the tested SAC methods (Supplementary Table 3). For the full-section analysis, the pipeline was configured to identify 5, 6, 7, 9, 11 and 14 clusters. For the consensus calling, the sample was subset to only the neoplasm region and the pipeline was configured to identify six clusters. The base clustering selection was done similarly to the LIBD DLPFC example (see above), via selecting a core set of ‘concordant’ methods according to pairwise ARIs and setting cutoffs on the hierarchical clustering tree (Fig. 5e, black box). As with the LIBD DLPFC consensus approach, the consensus clustering was generated using the LCA algorithm^49^. Marker genes for the neoplastic region and dysplasia were calculated with the tie-corrected Wilcoxon rank-sum test implemented in Scanpy^96^.

### Reporting summary

Further information on research design is available in the Nature Portfolio Reporting Summary linked to this article.

## Online content

Any methods, additional references, Nature Portfolio reporting summaries, source data, extended data, supplementary information, acknowledgements, peer review information; details of author contributions and competing interests; and statements of data and code availability are available at https://doi.org/10.1038/s41592-026-03194-8.

## Supplementary information

Supplementary Information Supplementary Discussion, Figs. 1–29, Tables 1–4 and Files 1 and 2.

Reporting Summary

Peer Review File

Supplementary Table 3

## Data Availability

Links to raw data are provided in the ‘Datasets’ section (Supplementary Table 1). All filtered data, intermediate results and final results are available via Zenodo at https://zenodo.org/records/15487519 (ref. ^97^).

## Code availability

The SACCELERATOR workflow and all relevant code to run the analysis are available as open-source software via GitHub at https://github.com/SpatialHackathon/SACCELERATOR. All code for reproducing results presented in this study, including figures and collected ARIs for the meta benchmark, is available via GitHub at https://github.com/SpatialHackathon/SpaceHack2023_study.
