# Supplementary material for: Beyond benchmarking: an expert-guided consensus approach to spatially aware clustering
Source: Nat Methods. 2026 Aug 24;23(9):1814–26. doi: 10.1038/s41592-026-03194-8 (PMC13541621; doi:10.1038/s41592-026-03194-8)
Supplement: Supplementary file 1 [file 41592_2026_3194_MOESM1_ESM.pdf]

nature methods

Supplementary information

https://doi.org/10.1038/s41592-026-03194-8

Beyond benchmarking: an expert-guided consensus approach to spatially aware clustering

In the format provided by the authors and unedited

Supplementary Information

Table of contents

Supplementary Discussion.

Supplementary Figures S1-S29.

Supplementary Tables S1, S2 and S4.

Supplementary File 1.

Supplementary File 2.

Supplementary Discussion

Practical guidelines for an expert-in-the-loop workflow

Bioinformatics analyses are inherently iterative and question-driven, and spatially aware clustering analyses are no exception. Their strong dependence on biological context is what challenges the idea of a one-size-fits-all benchmarking framework and motivates the development of the expert-in-the-loop workflow. While our workflow has demonstrated its utility across several case studies, its broader application inevitably depends on case-specific conditions and expert interpretation. To summarize this complexity, we compiled a concise guideline outlining key considerations and checkpoints for the workflow, including approaches for expert involvement, parameter selection, and ensuring reproducibility (Supplementary File 1). Rather than prescribing a standardized best practice, this guideline serves as a reference manual to promote thoughtful and transparent use of the workflow across diverse analysis settings.

Whether studies are hypothesis-driven or exploratory, expert knowledge offers critical guidance that helps prevent misinterpretations and analytical pitfalls. The scope and nature of involvement of tissue experts inevitably vary across projects. In the case studies, we demonstrate how experts can guide clustering granularity (Figure 4E), assess base clustering performance (Figure 5C), and interpret consensus-level biological features (Figure 4F-H). Beyond these specific instances, the guideline outlines additional scenarios for expert participation and the reasoning behind them. We also illustrate how they translate into actionable steps by mapping analysis decisions to guideline checkpoints, exemplified by a detailed breakdown of analysis logic for Case study 1 (Supplementary Figure S29), which offers practical insights into the workflow's implementation.

Base clustering (BC) and the selection of consensus algorithms are pivotal steps in the workflow. Strategies for BC selection can generally be classified into three categories: concordance-based, ranking-based, and manual selection. Manual selection relies on expert feedback or contextual biological criteria, while the other two approaches can be flexibly tailored to the research question at hand. In our case studies, base clusterings were chosen based on concordance blocks identified within the cross-method ARI heatmap (Figure 3B), as well as rankings of application-specific metrics that evaluate the spatial smoothness of clusters, such as SE (Supplementary Figure S24). Given the diversity of metrics included in the workflow, we encourage users to adapt these selection strategies to whichever metrics most effectively capture the biological phenomena of interest. Since consensus clustering aims to synthesize outputs from methods based on different modeling frameworks, a valuable sanity check during BC selection is to ensure a mix of clustering methods.

The choice of consensus algorithm should be tailored to the specific research question, taking into account tissue type, spatial technology, and the underlying biological hypothesis. In this study, our recommendations are primarily guided by scalability considerations (Supplementary Figure S16). We assessed runtime and memory usage for the three implemented consensus algorithms (K-modes, LCA, and weighted consensus) across a range of dataset sizes (i.e. number of cells or bins) and the number of input base clusterings. Overall, resource requirements increase with dataset size for all three methods. K-modes scaled best to large datasets, followed by LCA and weighted consensus. When varying the number of base clusterings, no consistent trend in runtime was observed; however, LCA showed a tendency toward higher memory consumption with increasing numbers

of base clusterings. Across both variables, K-modes was the most resource-efficient, Weighted consensus was the most resource-intensive, and LCA showed intermediate performance.

A critical, yet often underexplored, aspect of spatial clustering is the investigation of the granularity of tissue features, analogous to the granularity considerations commonly addressed in cell typing for single-cell transcriptomics. We further examined the clustering granularity in the Xenium mouse brain dataset (Supplementary Figure S9). Although a broad range of cluster numbers corresponded well with established anatomical segmentations, clusterings with very low numbers were insufficient to resolve larger, coarse anatomical structures and very high cluster numbers tended to capture cellular heterogeneity rather than spatially contiguous anatomical domains. This finding highlights the importance of selecting an appropriate clustering resolution that balances anatomical interpretability with cellular detail when analyzing spatial transcriptomic data.

Although only two case studies were presented, the selected datasets represent two major classes of spatial transcriptomic technologies: imaging-based (MERFISH) and sequencing-based (Visium HD). They also cover tissue types that particularly benefit from spatial context, including brain and solid tumors. We expect that the practical guideline, comprehensive documentation, and aforementioned considerations will encourage the community to further evaluate and adapt the workflow across a wide range of settings. As a first step to facilitate those adaptations, we have included unsupervised (i.e., no expert involvement) consensus results from a selection of representative datasets covering different recent technologies (Supplementary Figure S18 to S22, Data Availability) as an initial resource to spur further investigation.

Supplementary Figures

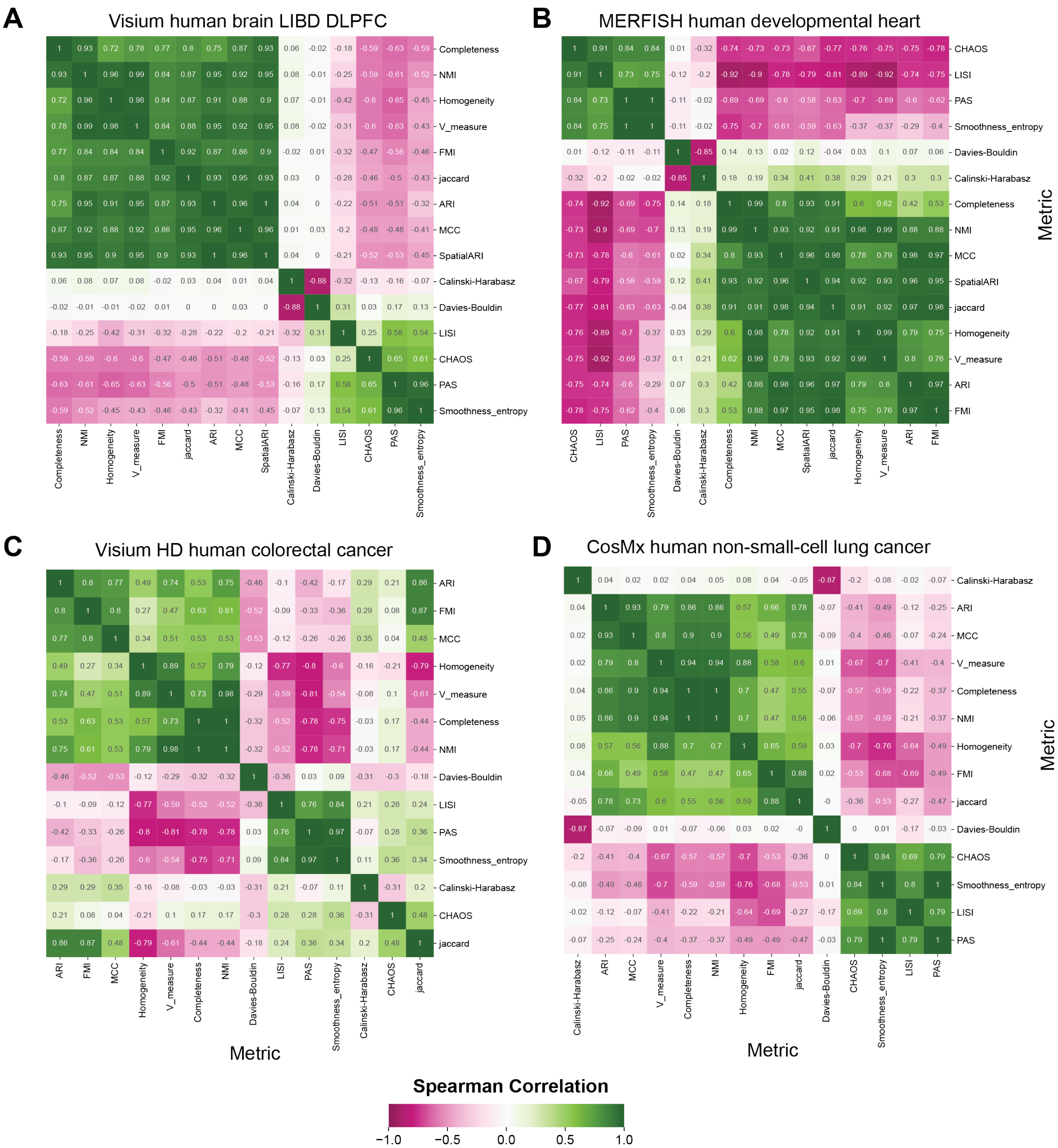

Figure S1: Spearman correlation matrix of different metrics across all methods and samples from **A**. LIBD DLPFC, **B**. MERFISH human developmental heart, **C**. Visium HD human colorectal cancer, **D**. CosMx lung.

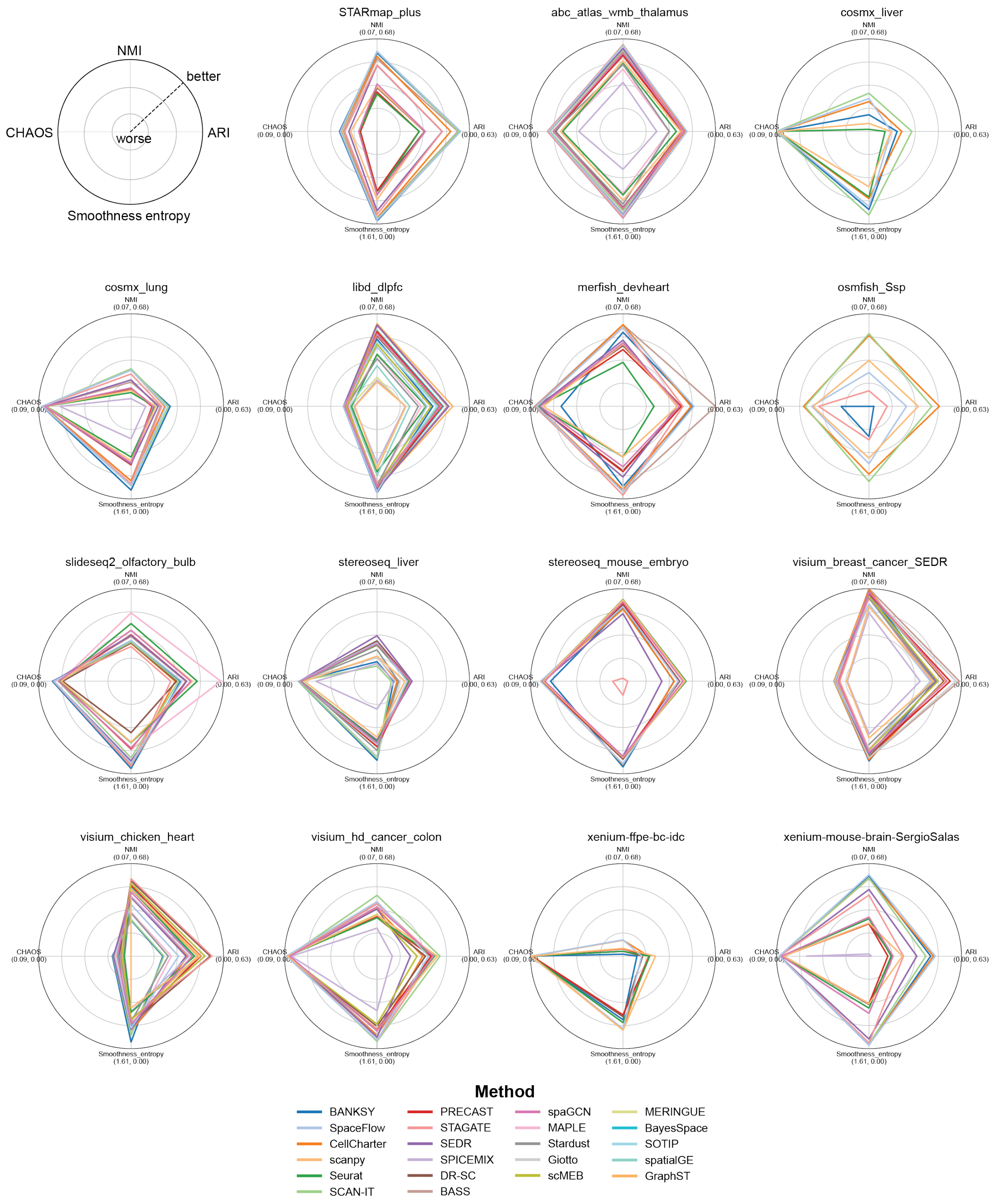

Figure S2: Radar plot for all datasets and each method across 4 metrics: ARI, NMI, Smoothness Entropy, and CHAOS. Smoothness Entropy and Chaos scores are inverted so that the outer range of the radar plot corresponds to better scores.

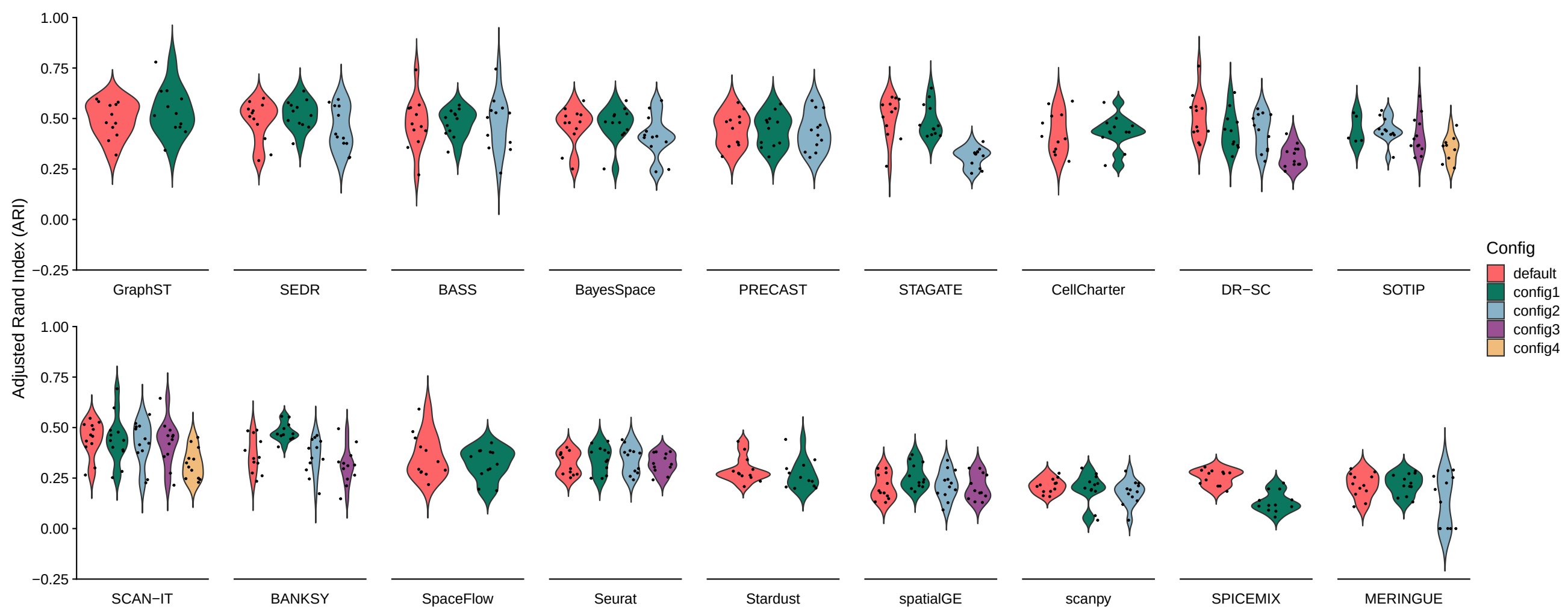

Figure S3: ARI for methods with multiple configurations applied to the LIBD DLPFC dataset. Methods are ordered by mean ARI.

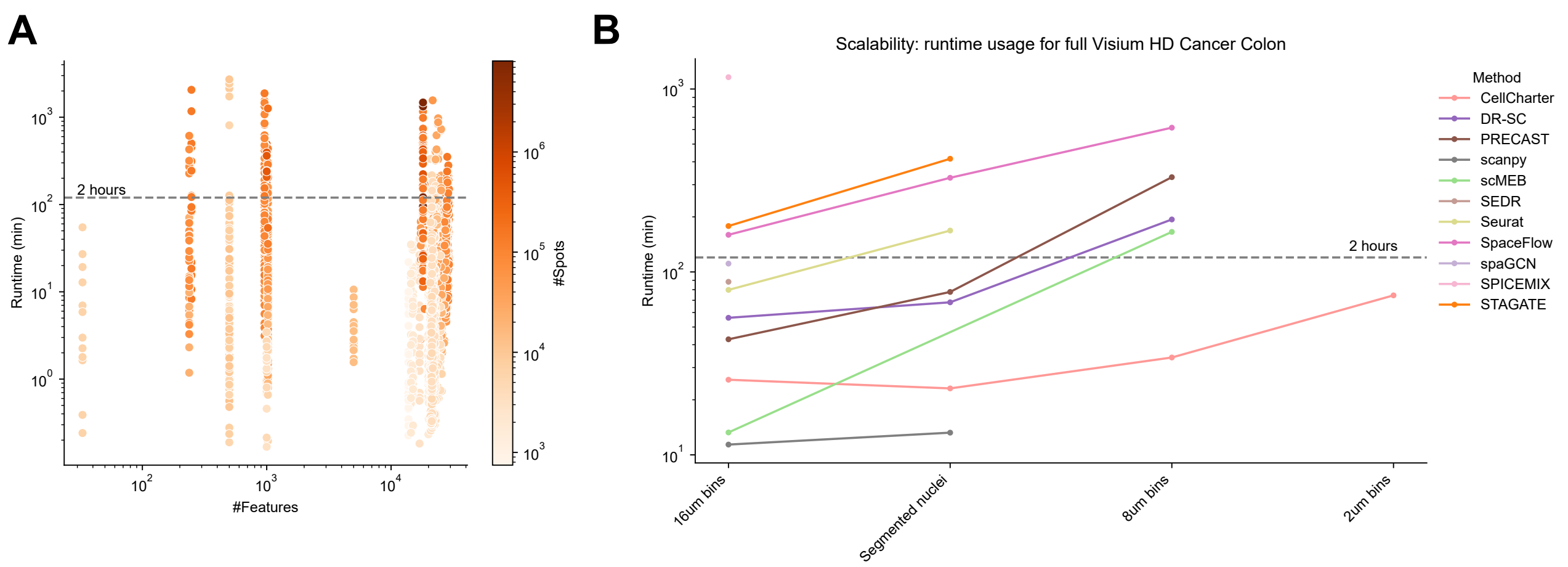

Figure S4: **A**. CPU runtime vs the number of features, colored by the number of spots/cells in the dataset. **B**. CPU runtime of methods on the full Visium HD colorectal cancer dataset across various bin sizes.

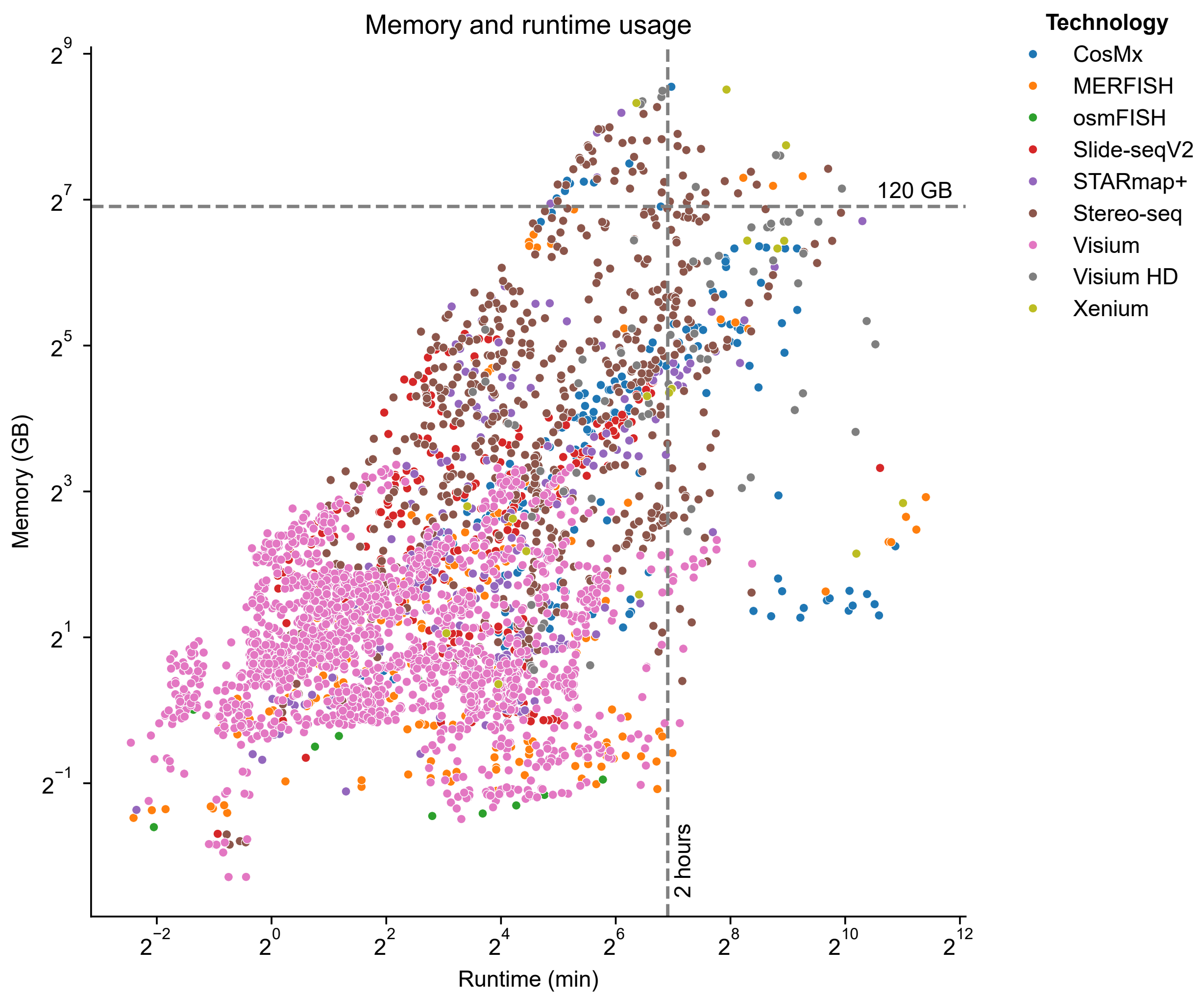

Figure S5: Memory usage vs CPU runtime, colored by the technology of the dataset.

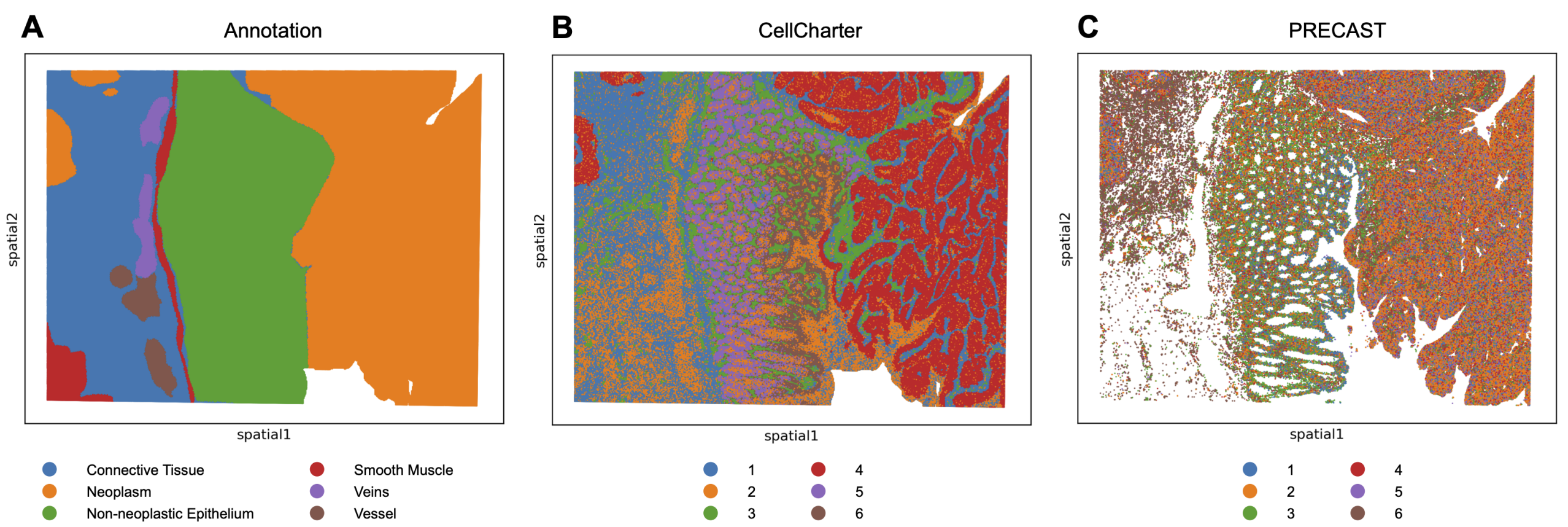

Figure S6: Spatial plots of the cropped Visium HD colon cancer dataset with 2 µm bins, colored by **A**. ground truth, **B**. CellCharter clusters, and **C**. PRECAST clusters.

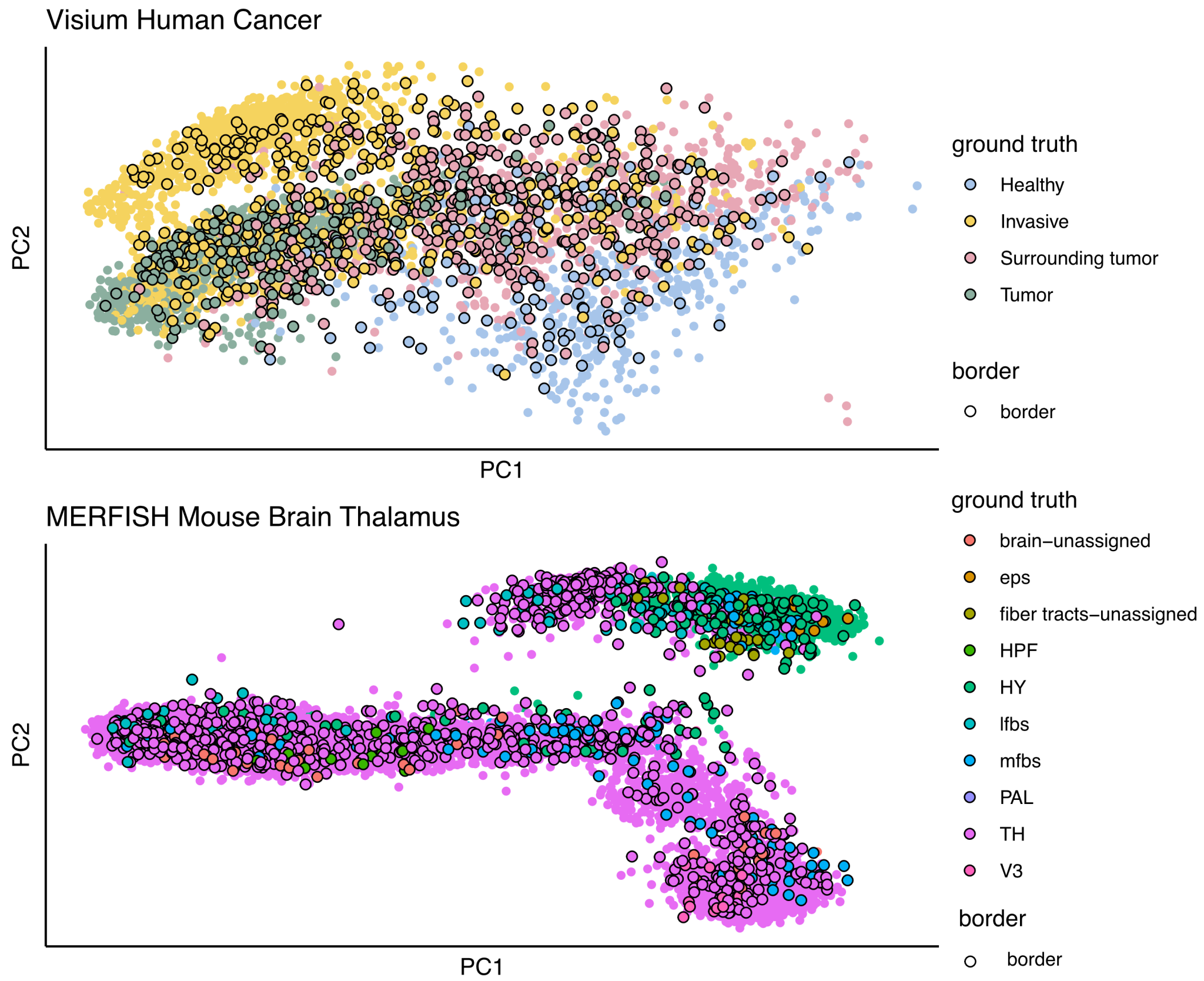

Figure S7: Visium human cancer, and MERFISH mouse brain thalamus datasets, projected on the 1st and 2nd principal component (PC), colored by GT labels. Spots that are spatially located at the border between GT clusters are marked with black outlines.

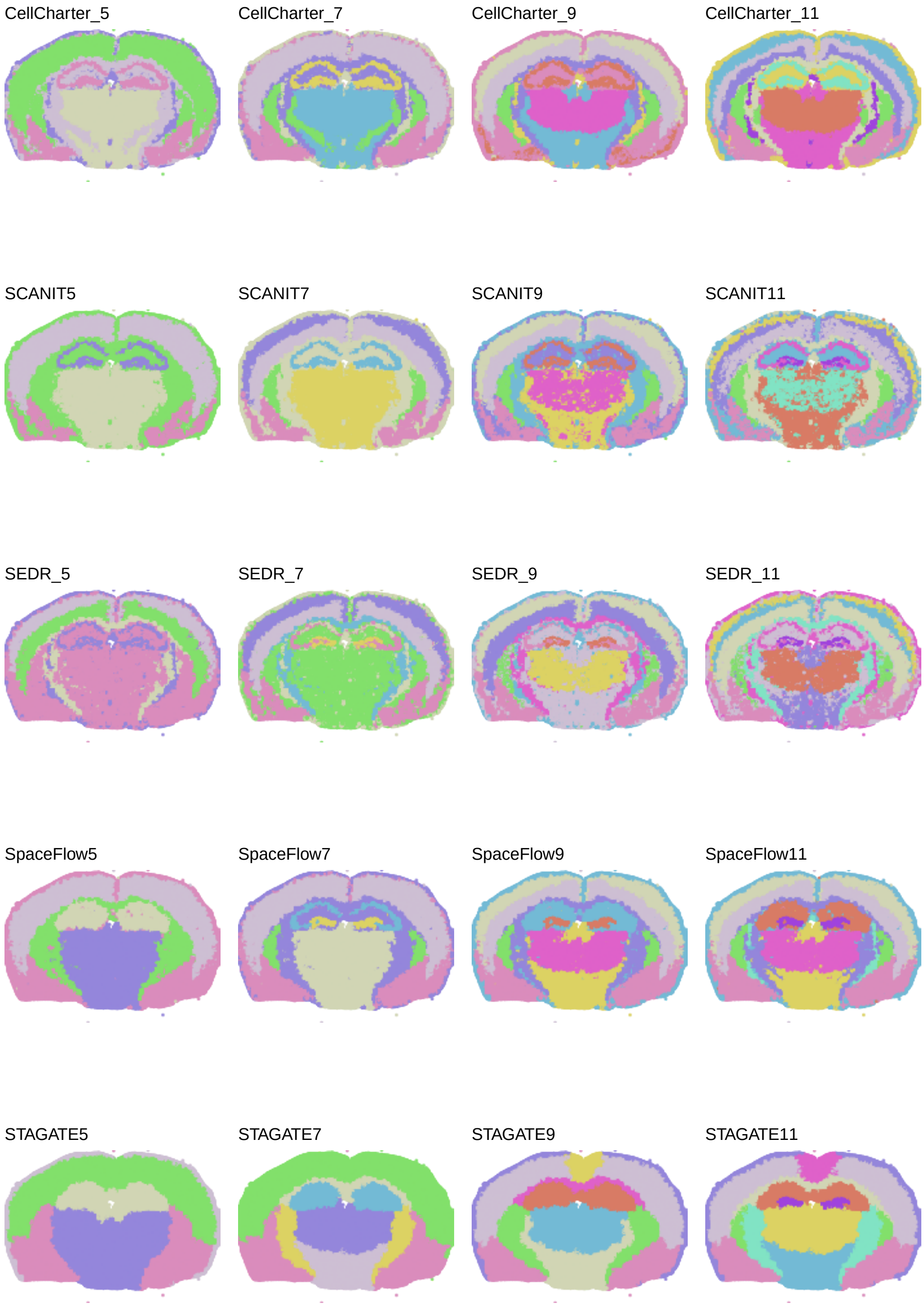

Figure S8: Spatial plots of Xenium mouse brain dataset cluster sweep results.

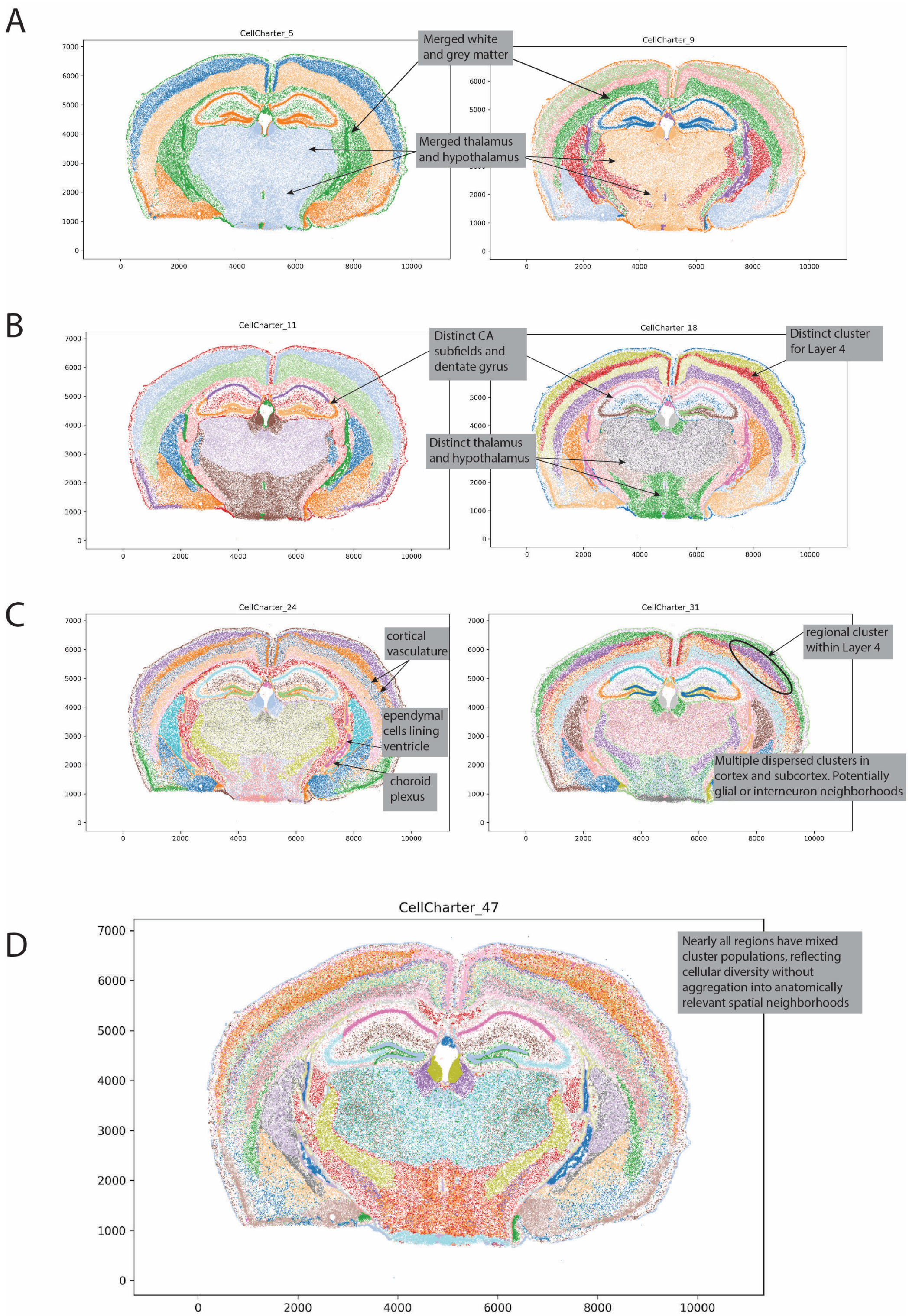

Figure S9: SAC cluster sweep encompasses high- and low-resolution clustering results. In this annotated example using CellCharter on the Xenium mouse brain data, we covered a wide range of cluster parameters to illustrate how spatial resolution is related to cluster number. Subplot titles indicate the number of clusters. A. Low numbers of clusters fail to resolve even coarse anatomical regions. B. Moderate cluster numbers include highly resolved anatomical structures, including cortical layers, structures within the hippocampus and distinct domains for thalamus and hypothalamus. C. High cluster numbers showing further resolution, which may be desirable for some studies, but some anatomical regions begin to include multiple clusters built on distinct cellular populations. D. Very high cluster number showing salt-and-pepper distributions of clusters across the brain, reflecting cellular diversity rather than contiguous spatial domains.

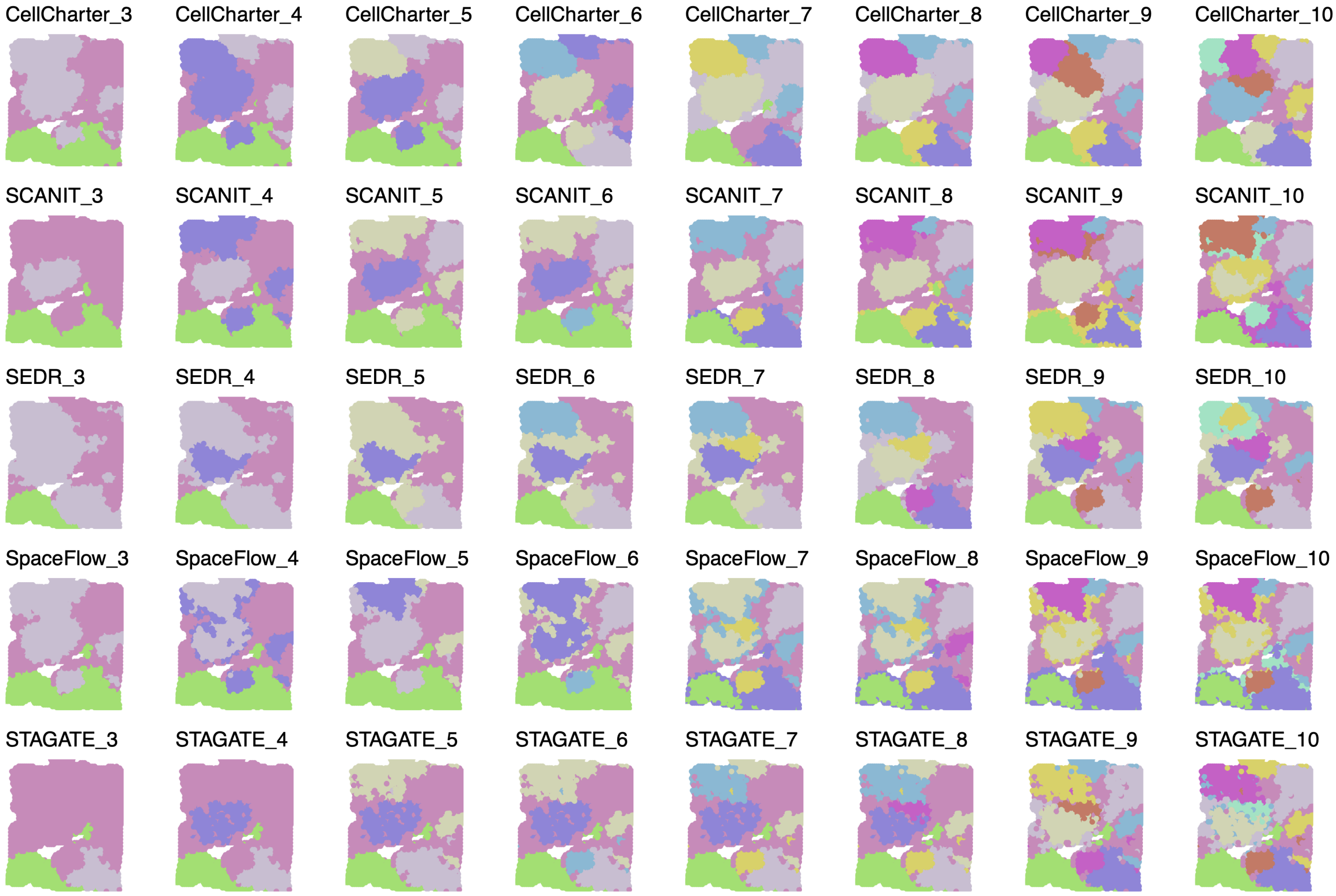

Figure S10: Spatial plots of Visium breast cancer dataset cluster sweep results.

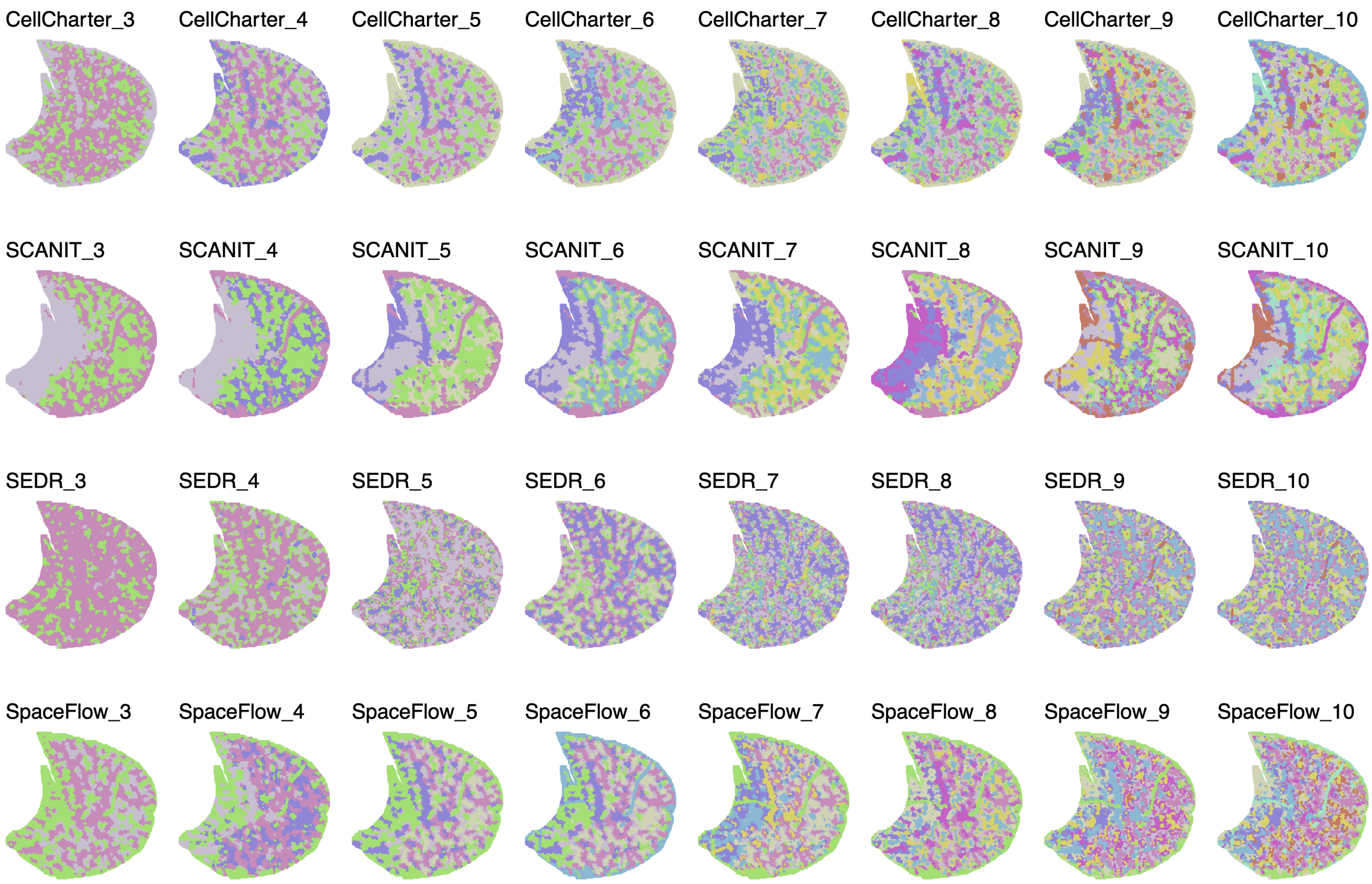

Figure S11: Spatial plots of Stereo-seq mouse liver dataset cluster sweep results with all genes.

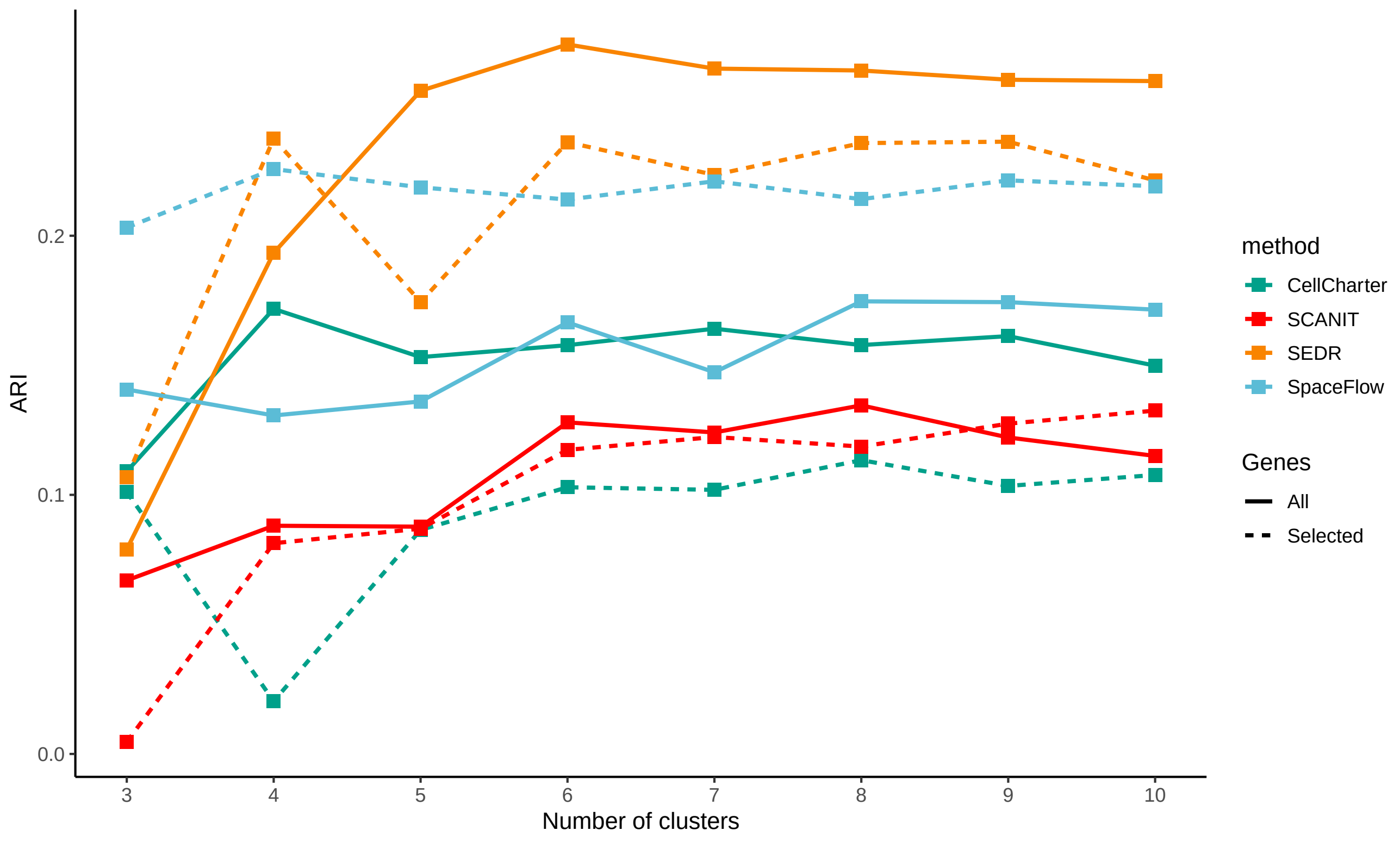

Figure S12: ARI results of Stereo-seq mouse liver datasets with all genes or selected marker genes.

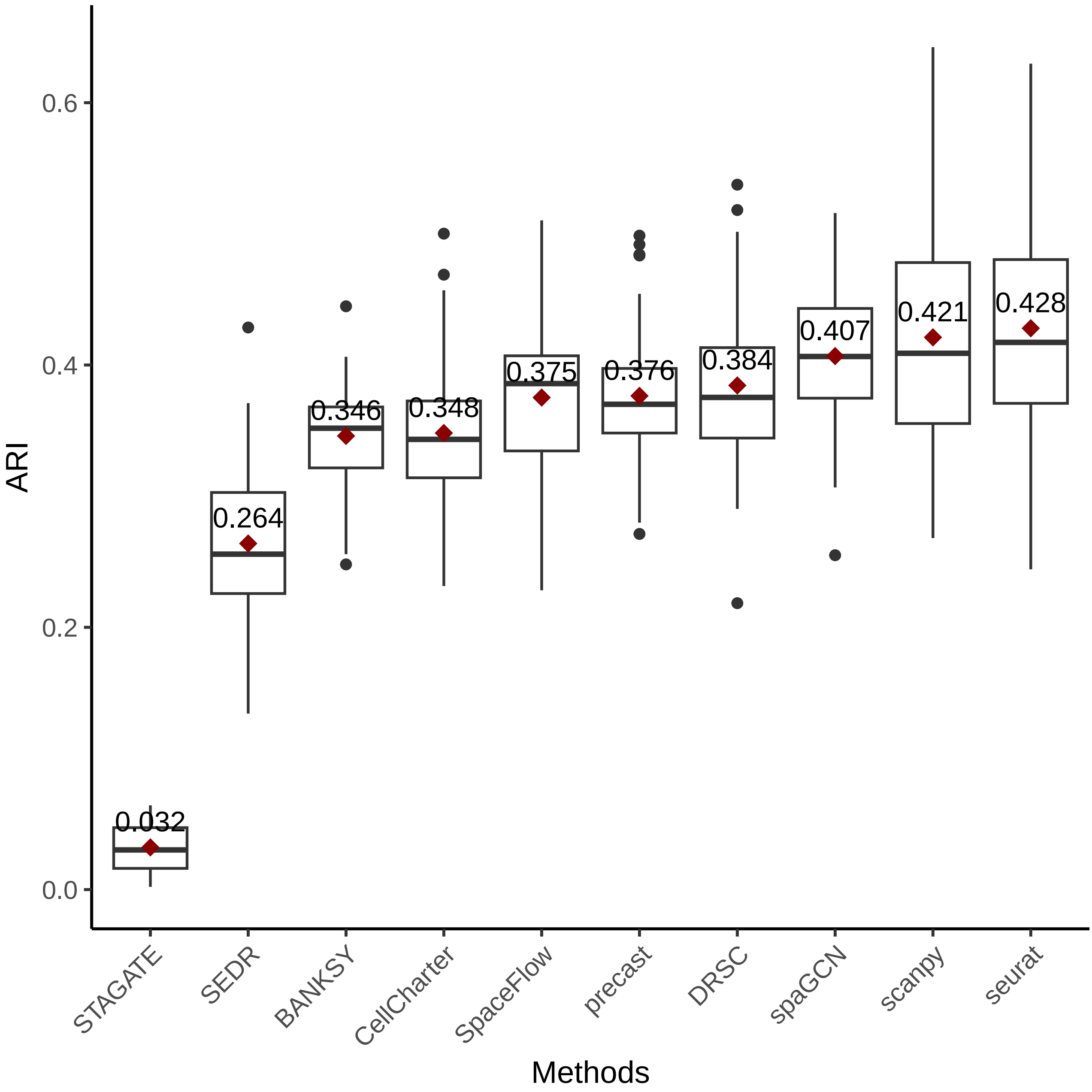

Figure S13: ARI results for Stereo-seq mouse embryo dataset, with mean value indicated in red square and corresponding values.

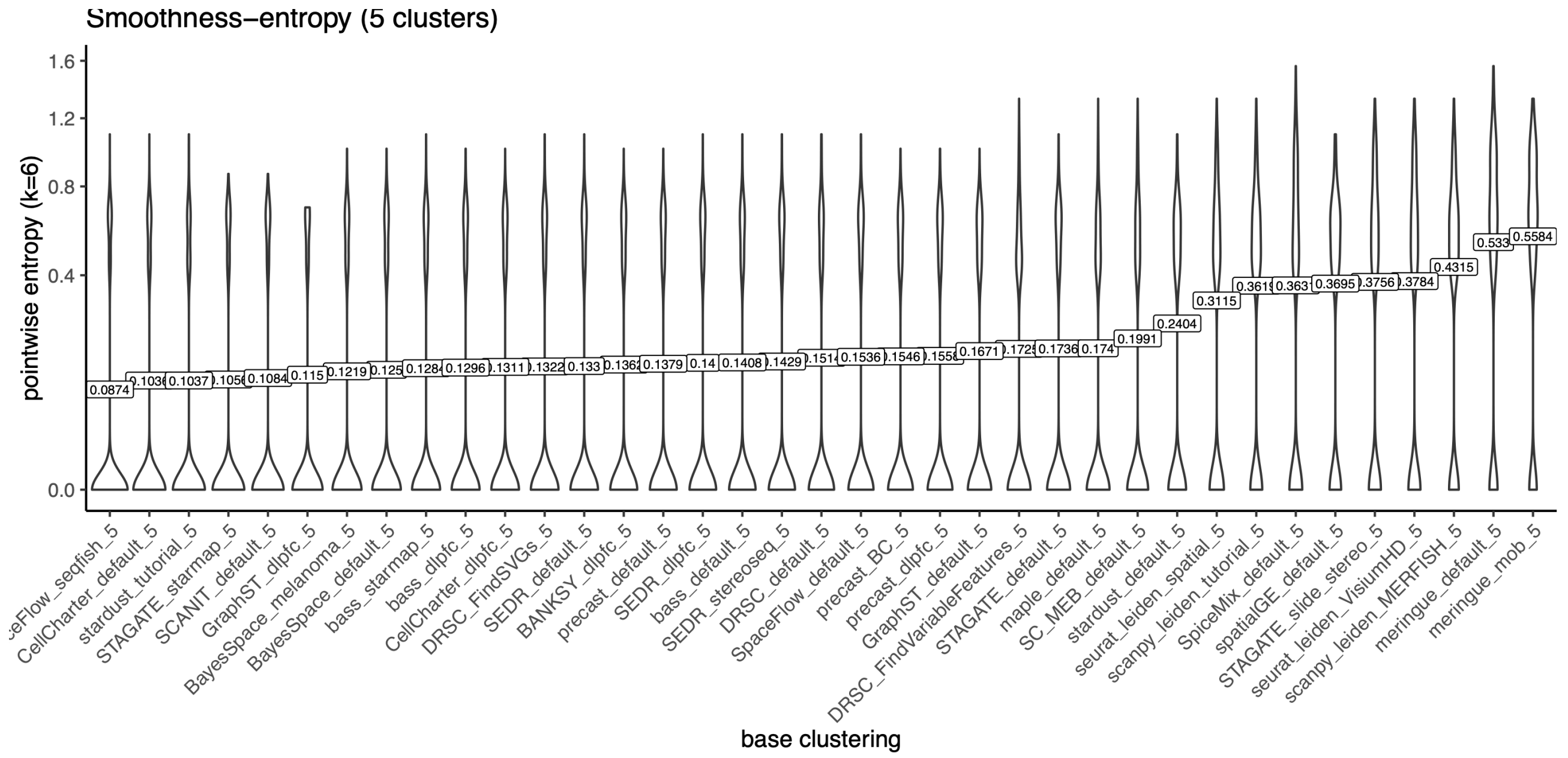

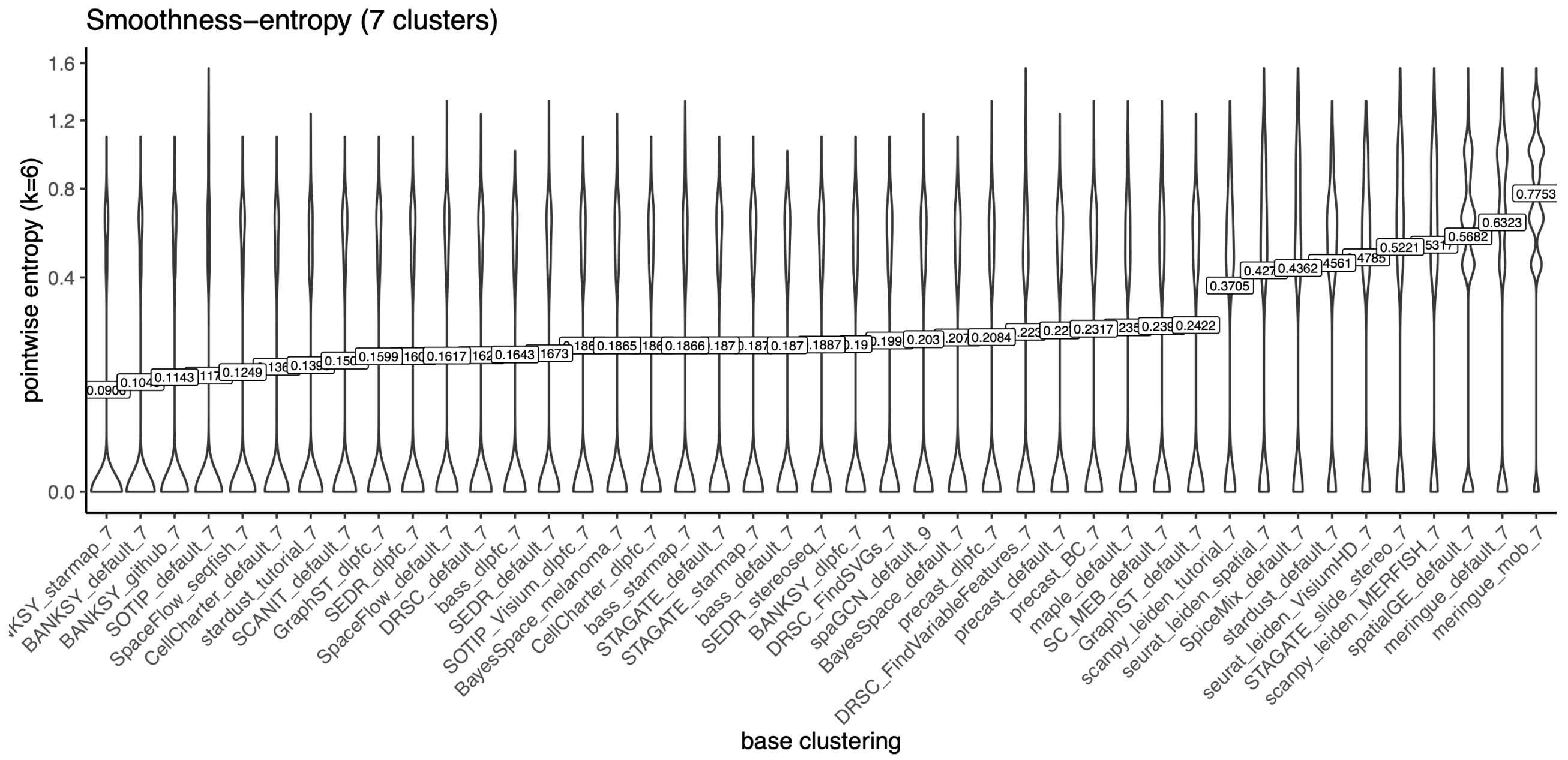

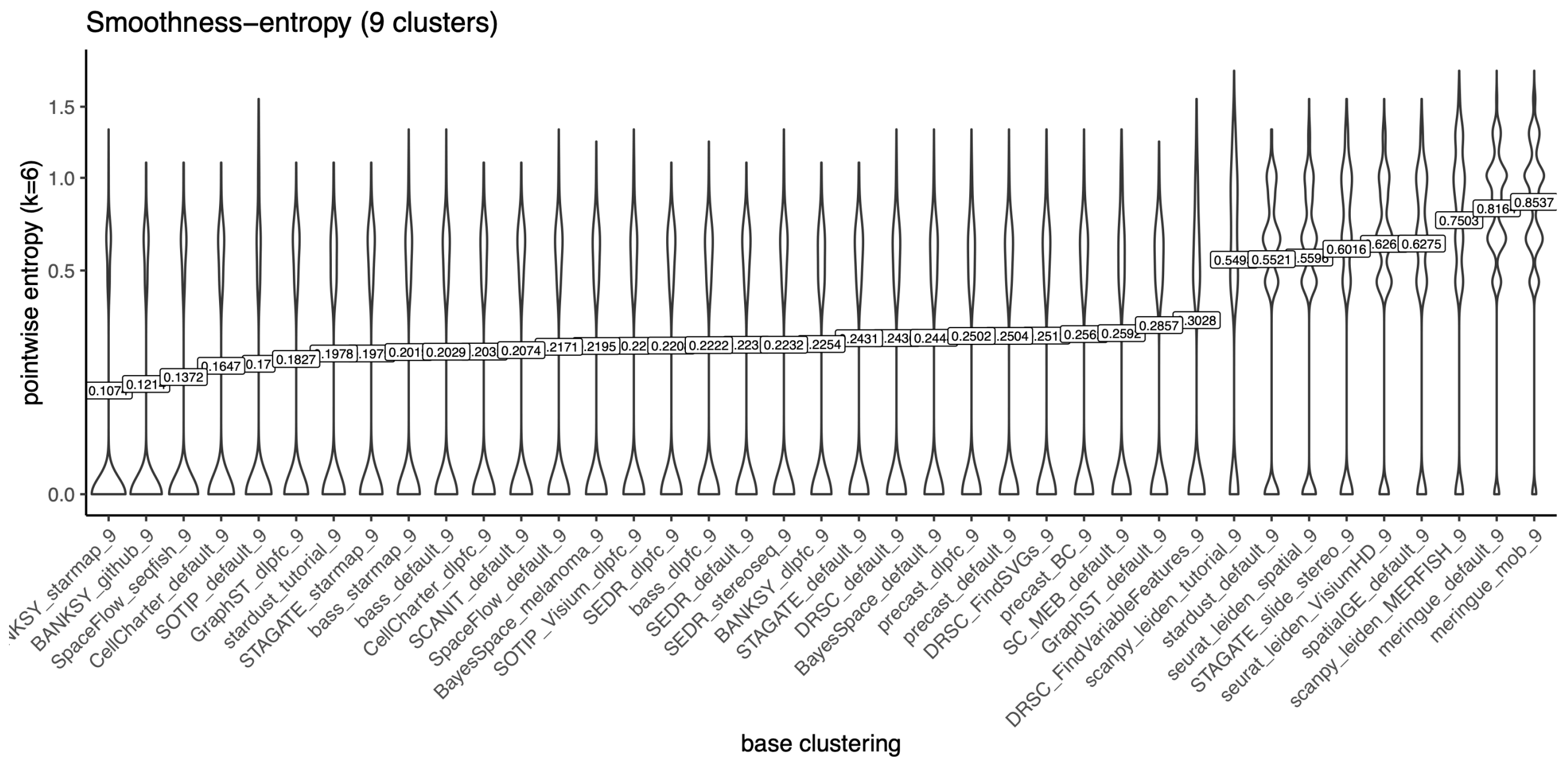

Figure S14: Distributions of SE scores for all base clusterings of LIBD DLPFC slice 151673, with mean value indicated in the text boxes.

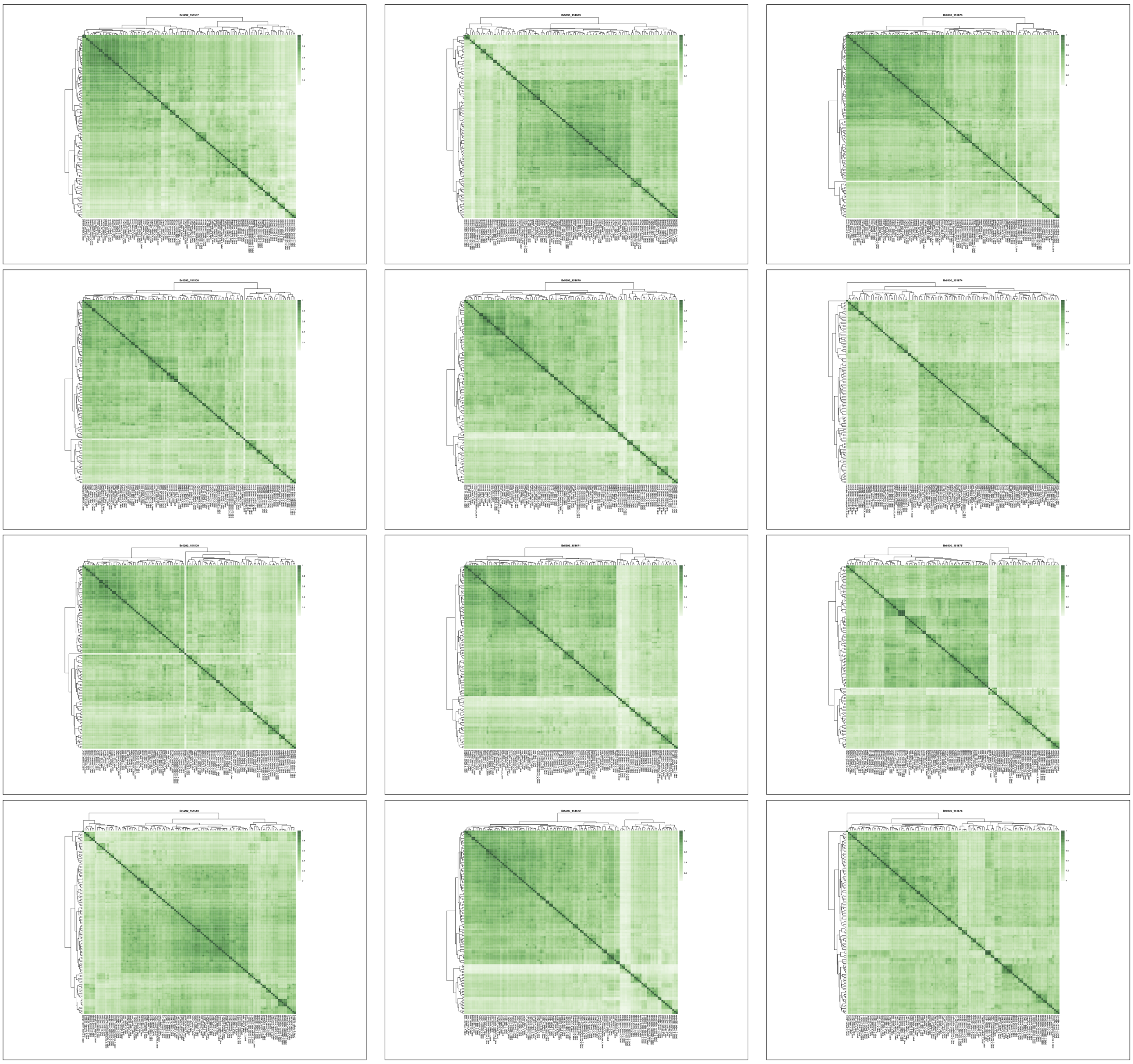

Figure S15: Heatmaps of pairwise ARIs between all base clusterings for each LIBD DLPFC slice. In all cases, we observed a block of concordant methods.

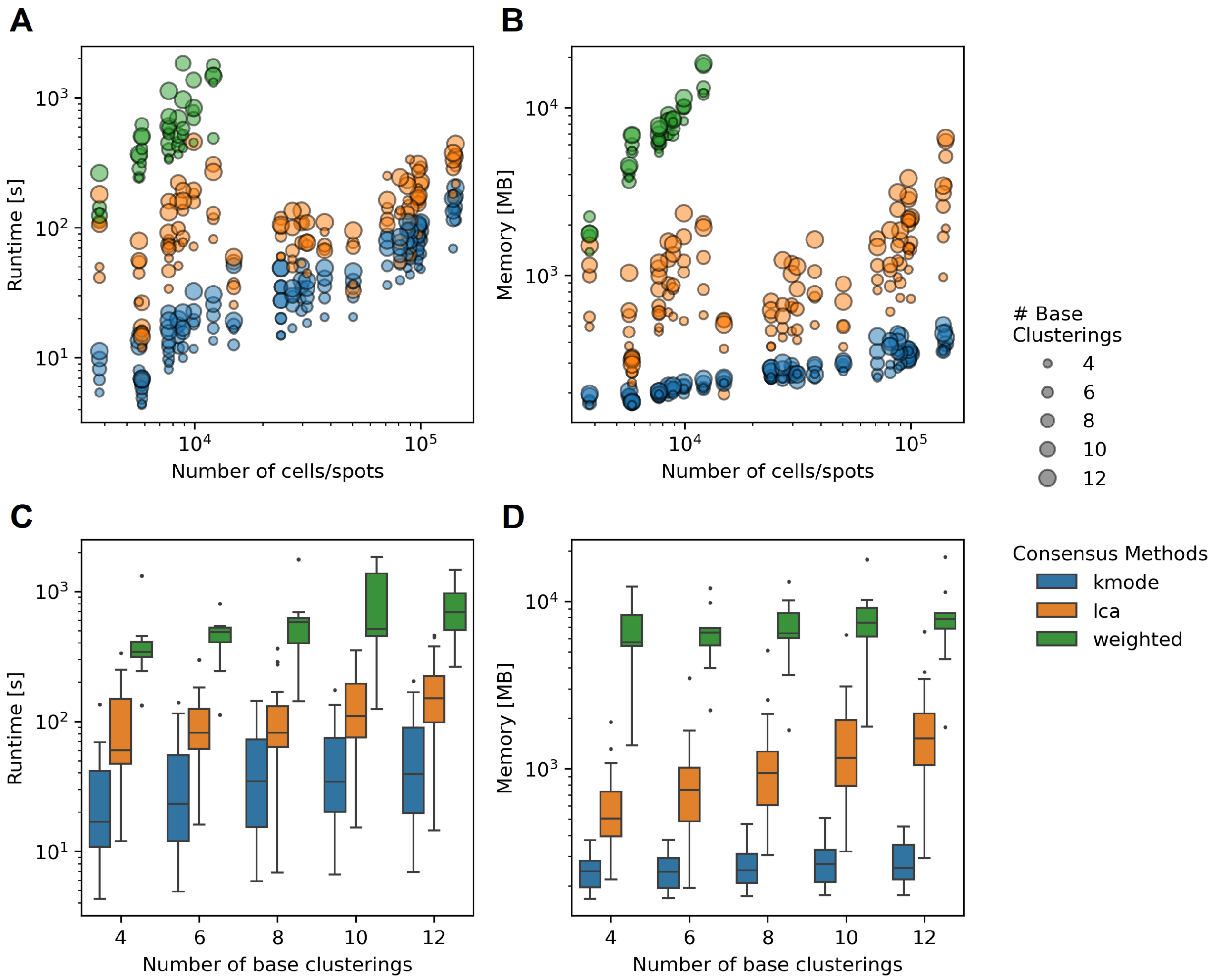

Figure S16: Scalability analysis of consensus algorithms. (A, B) Consensus algorithm runtime (A) and memory (B) w.r.t. number of cells and spots in the dataset. **(C, D)** Consensus algorithm runtime (C) and memory (B) w.r.t. number of base clustering input.

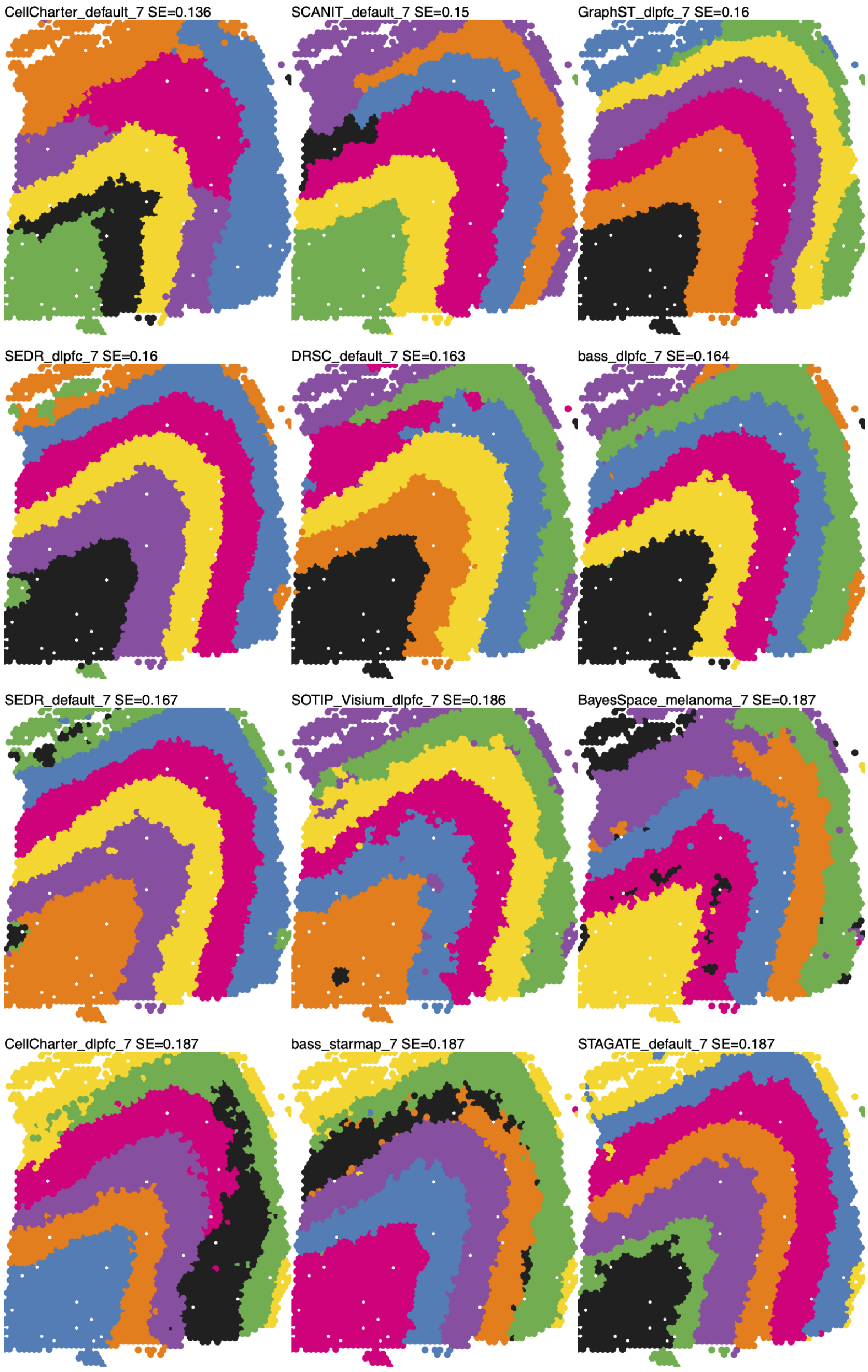

Figure S17: 12 selected base clusterings at 7 clusters to form consensus used for Figure 3B ("consensus_lca", and represented in Figure 3D) for LIBD DLPFC slice 151673.

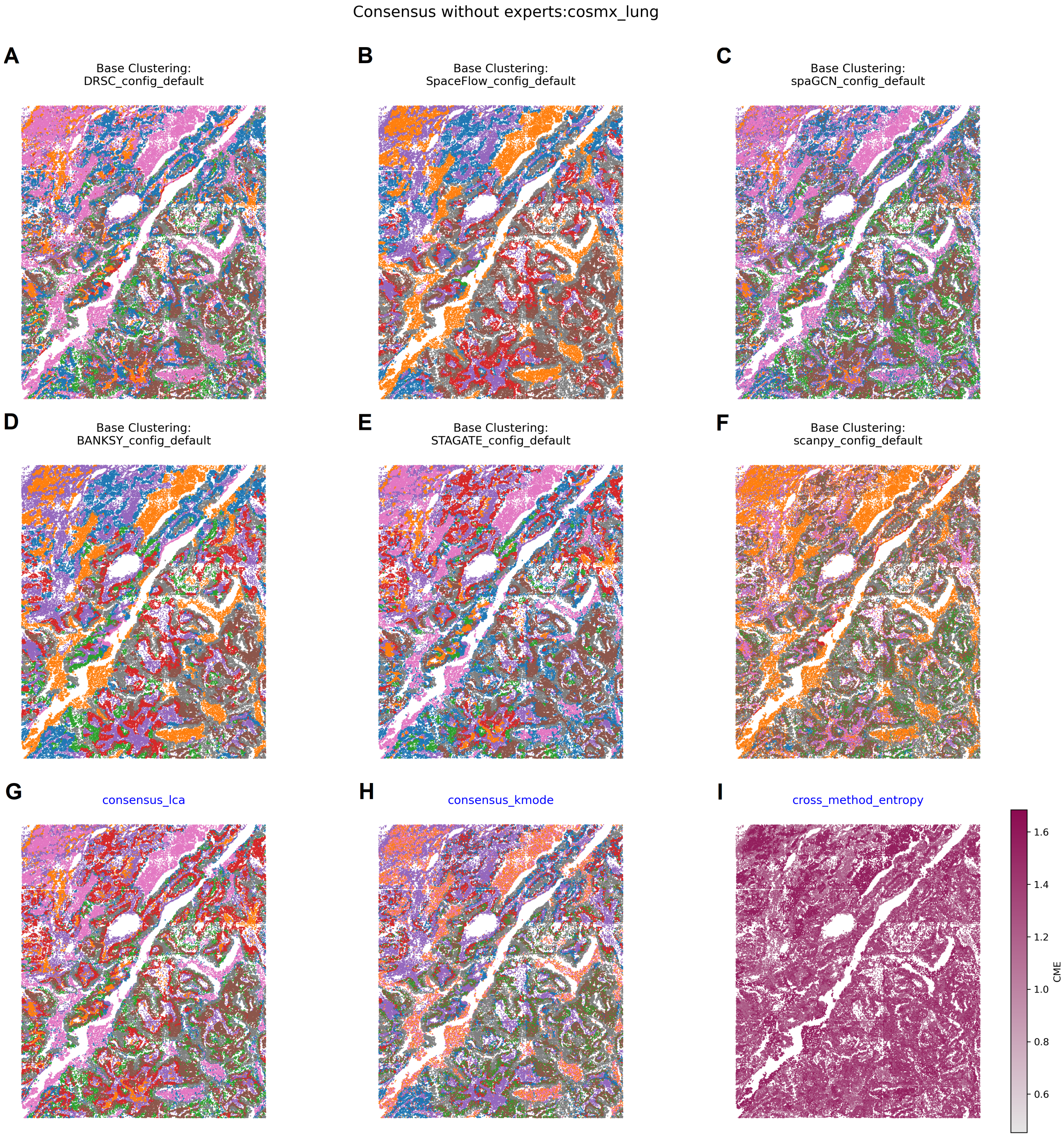

Figure S18: Base and consensus clusterings for the CosMX lung dataset. **(A-F)** Base clustering from methods using default configurations set to 8 clusters. **(G-H)** Consensus clustering using LCA and K-modes consensus building. Results for weighted are not shown as the computation did not complete. **(I)** Cross method entropy heatmap.

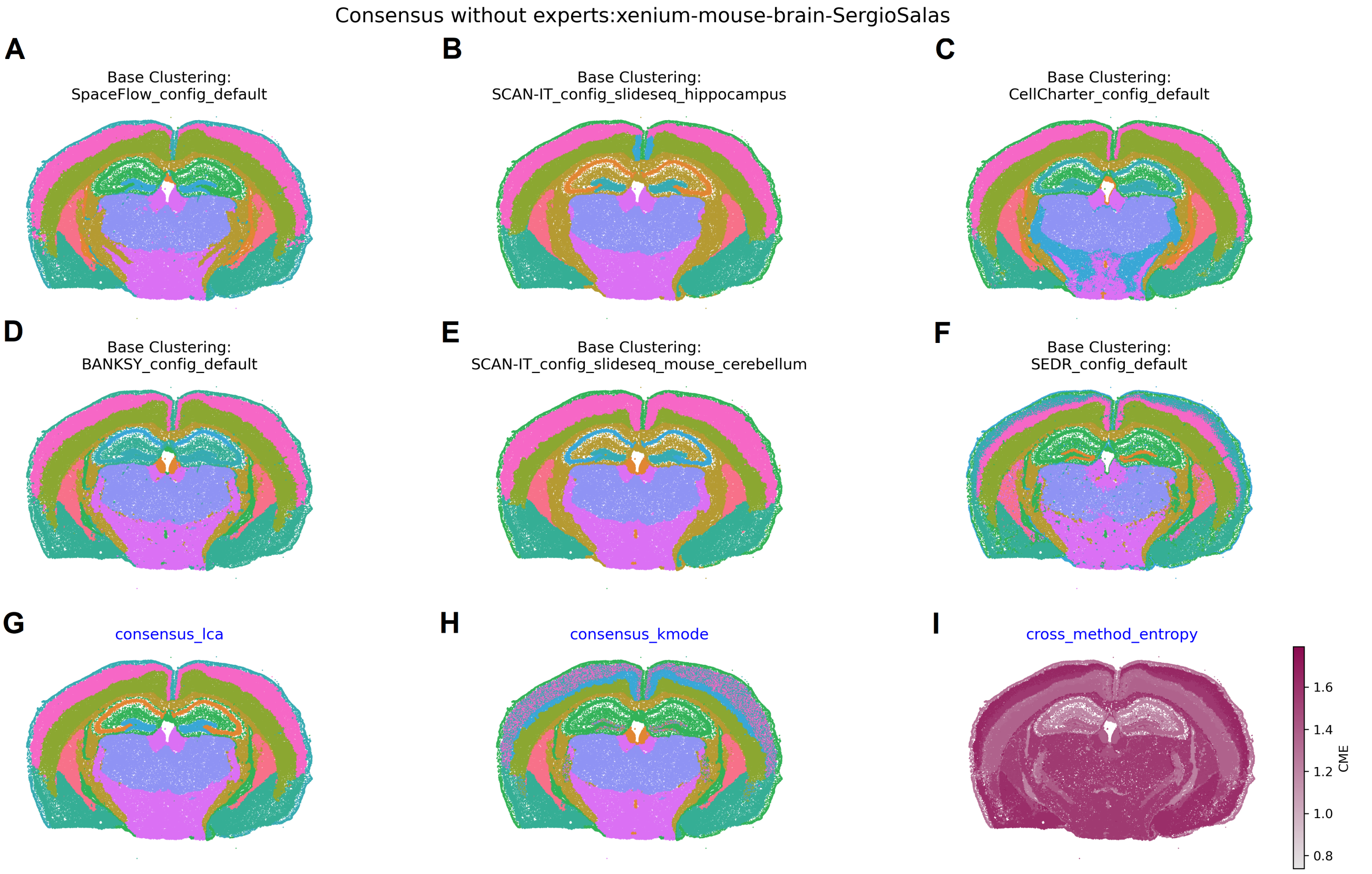

Figure S19: Base and consensus clusterings for the Xenium mouse brain dataset. **(A-F)** Base clustering from methods using default configurations set to 11 clusters. **(G-H)** Consensus clustering using LCA and K-modes consensus building. Results for weighted are not shown as the computation did not complete. **(I)** Cross method entropy heatmap.

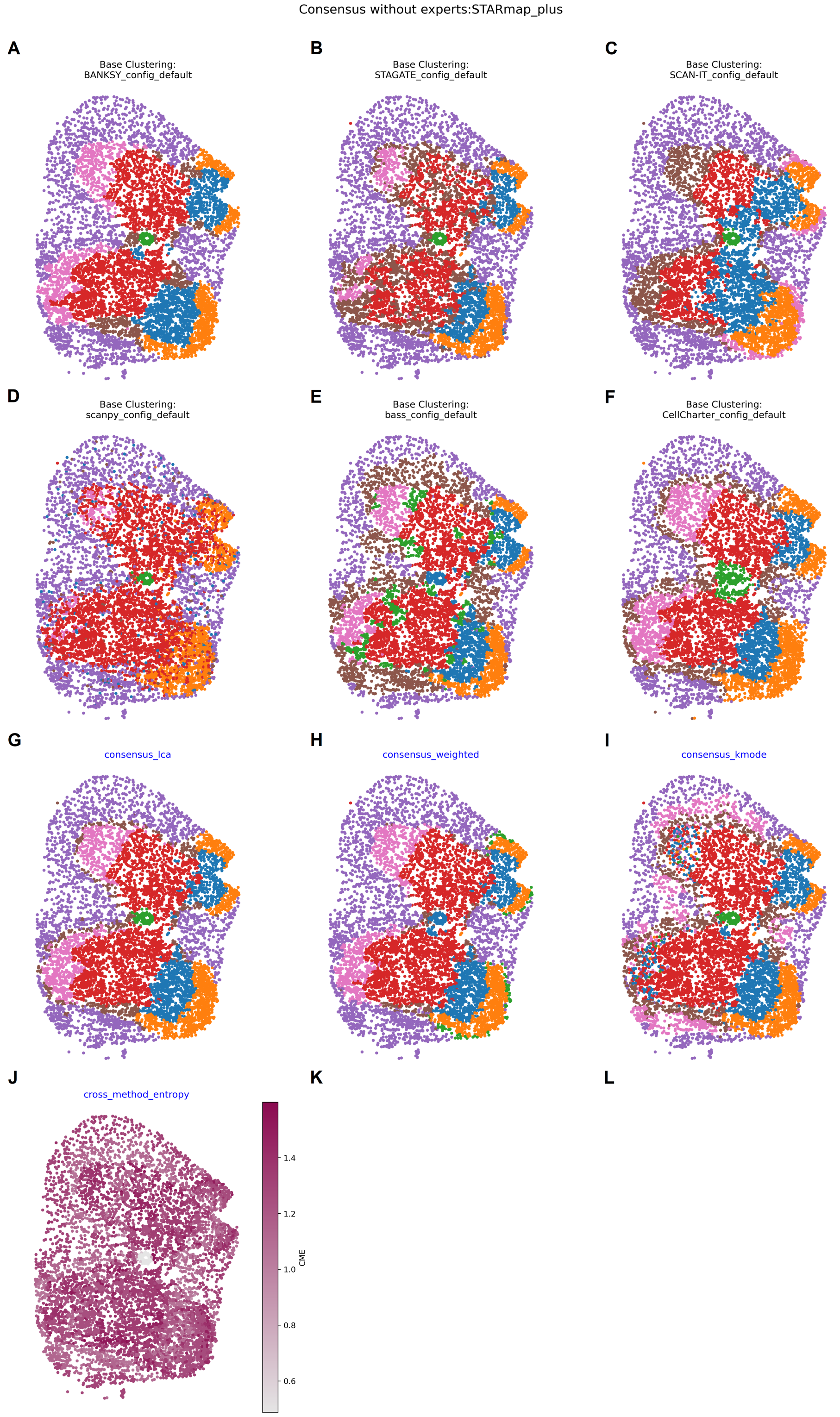

Figure S20: Base and consensus clusterings for the STARmap plus mouse brain dataset. **(A-F)** Base clustering from methods using default configurations set to 7 clusters. **(G-I)** Consensus clustering using LCA, K-modes and weighted consensus building. **(J)** Cross method entropy heatmap.

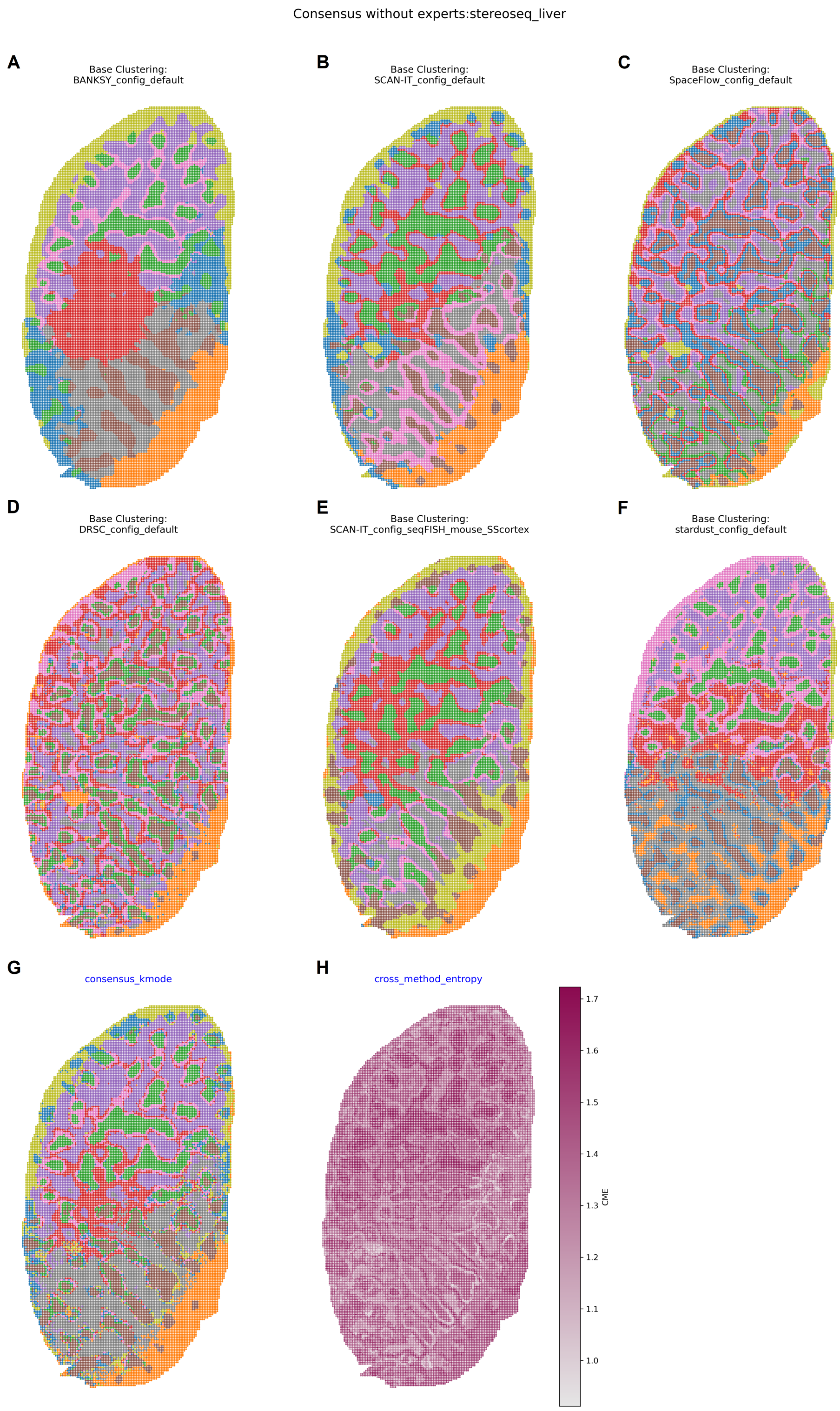

Figure S21: Base and consensus clusterings for the Stero-seq liver dataset. **(A-F)** Base clustering from methods using default configurations set to 9 clusters. **(G)** Consensus clustering using K-modes. Results for LCA and weighted are not shown as the computation did not complete. **(H)** Cross method entropy heatmap.

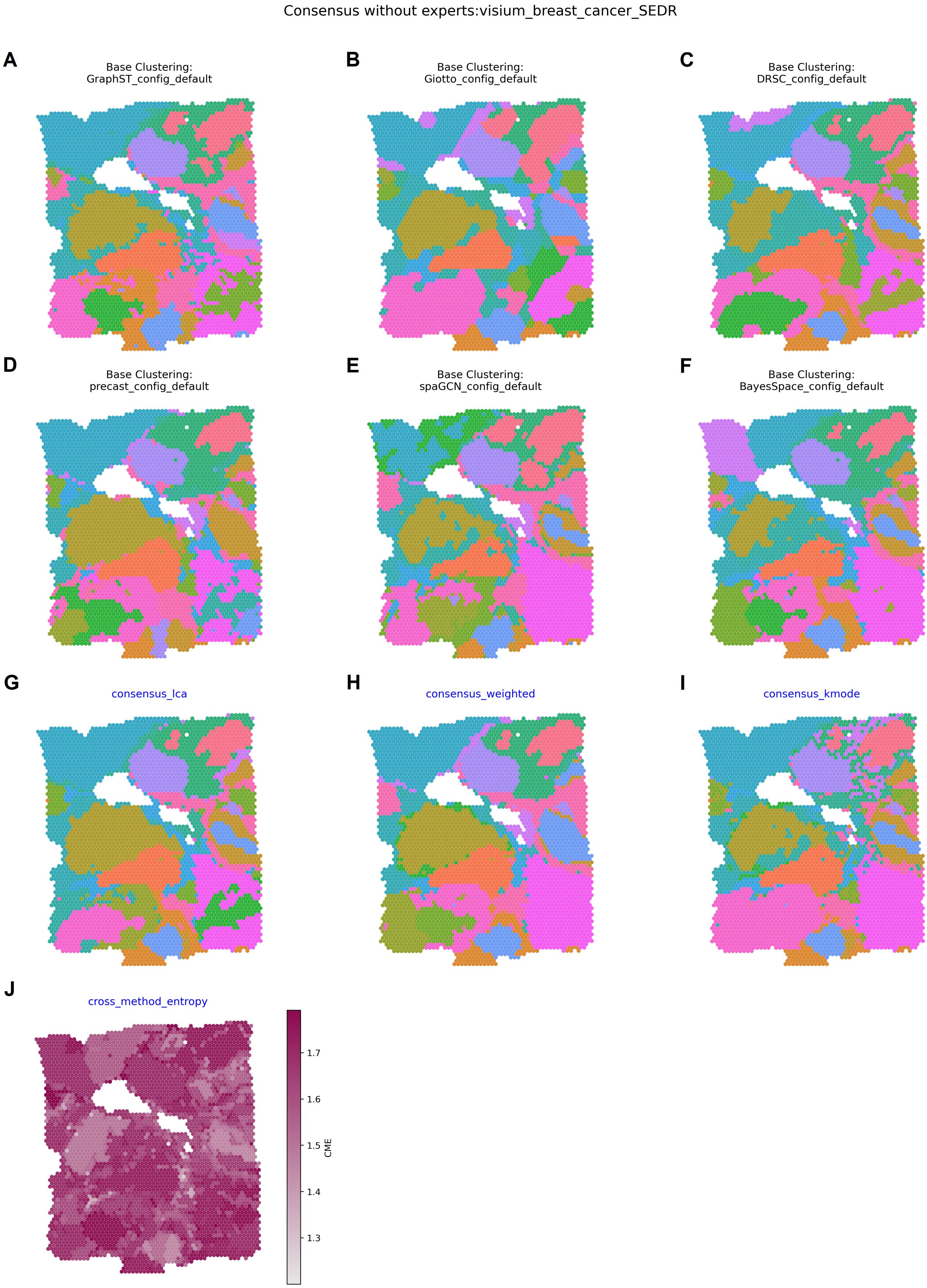

Figure S22: Base and consensus clusterings for the Visum breast cancer dataset. **(A-F)** Base clustering from methods using default configurations set to 20 clusters. **(G-I)** Consensus clustering using LCA, K-modes and weighted consensus building. **(J)** Cross method entropy heatmap.

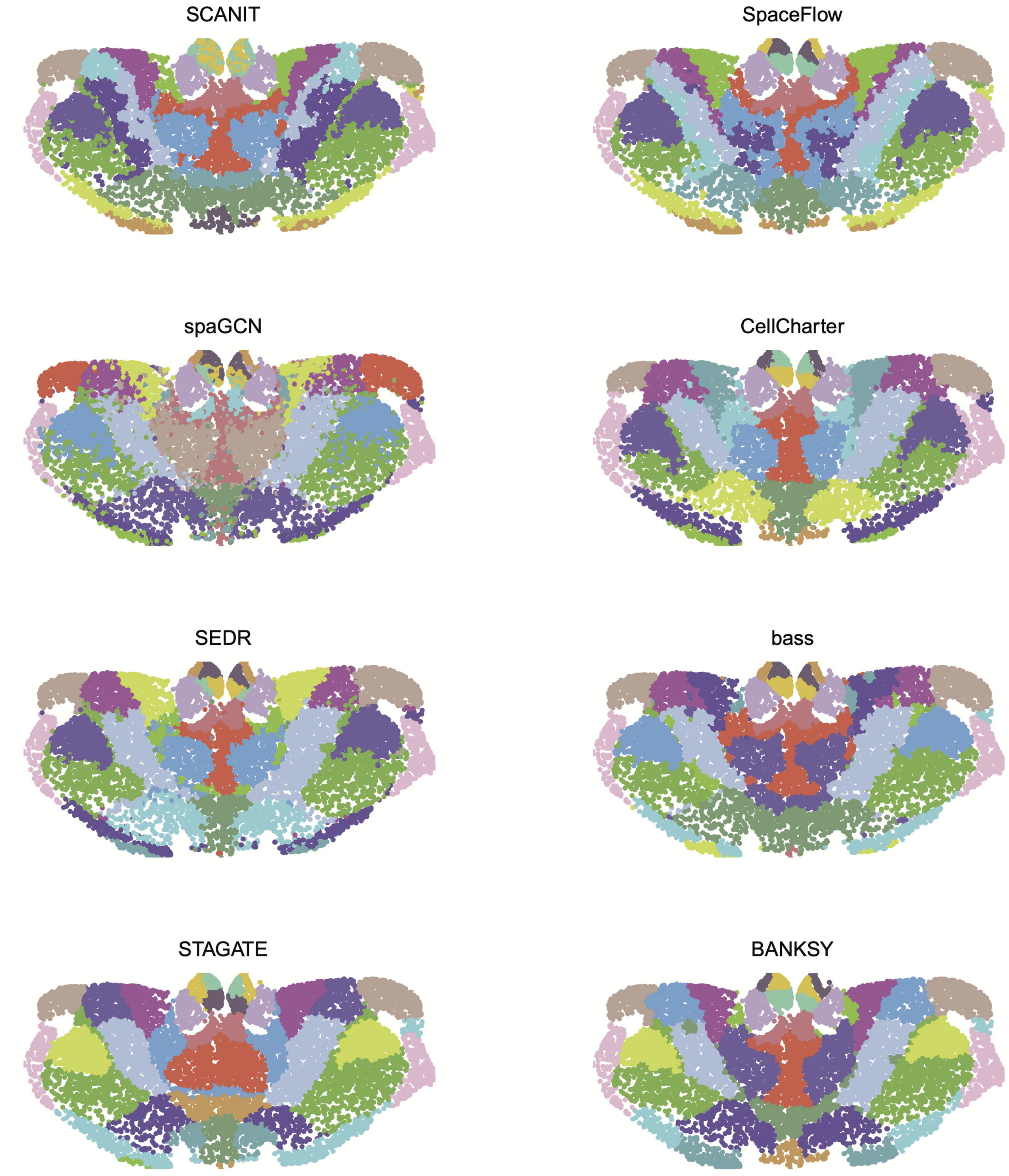

Figure S23: Clusters in the mouse thalamus generated from running 8 SAC methods with the cluster parameter set to 20.

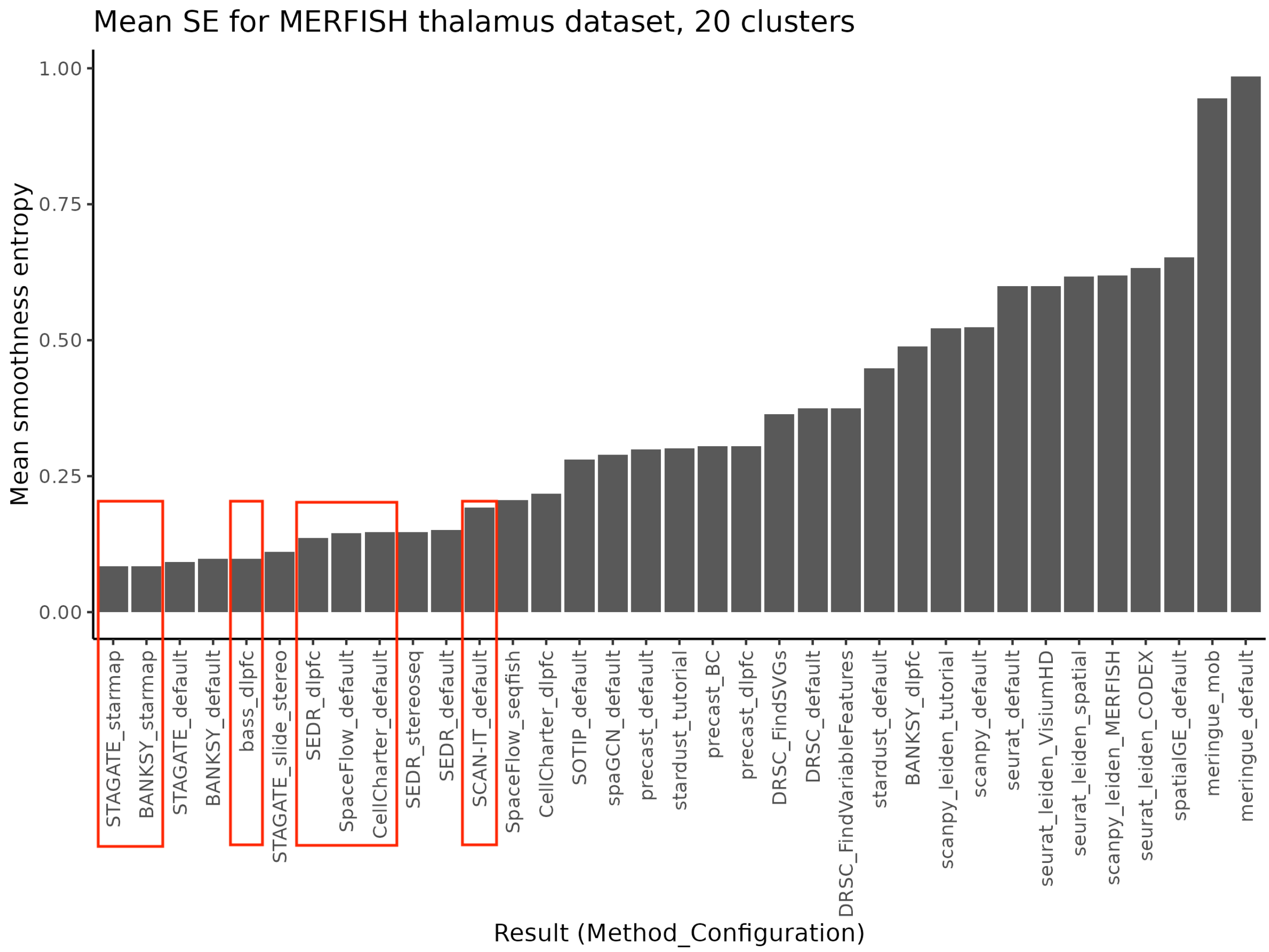

Figure S24: Smoothness entropy (SE) of MERFISH thalamus dataset with 20 clusters. Selected instances of base clusterings are marked by red boxes. Only the smoothness configuration of each method is selected.

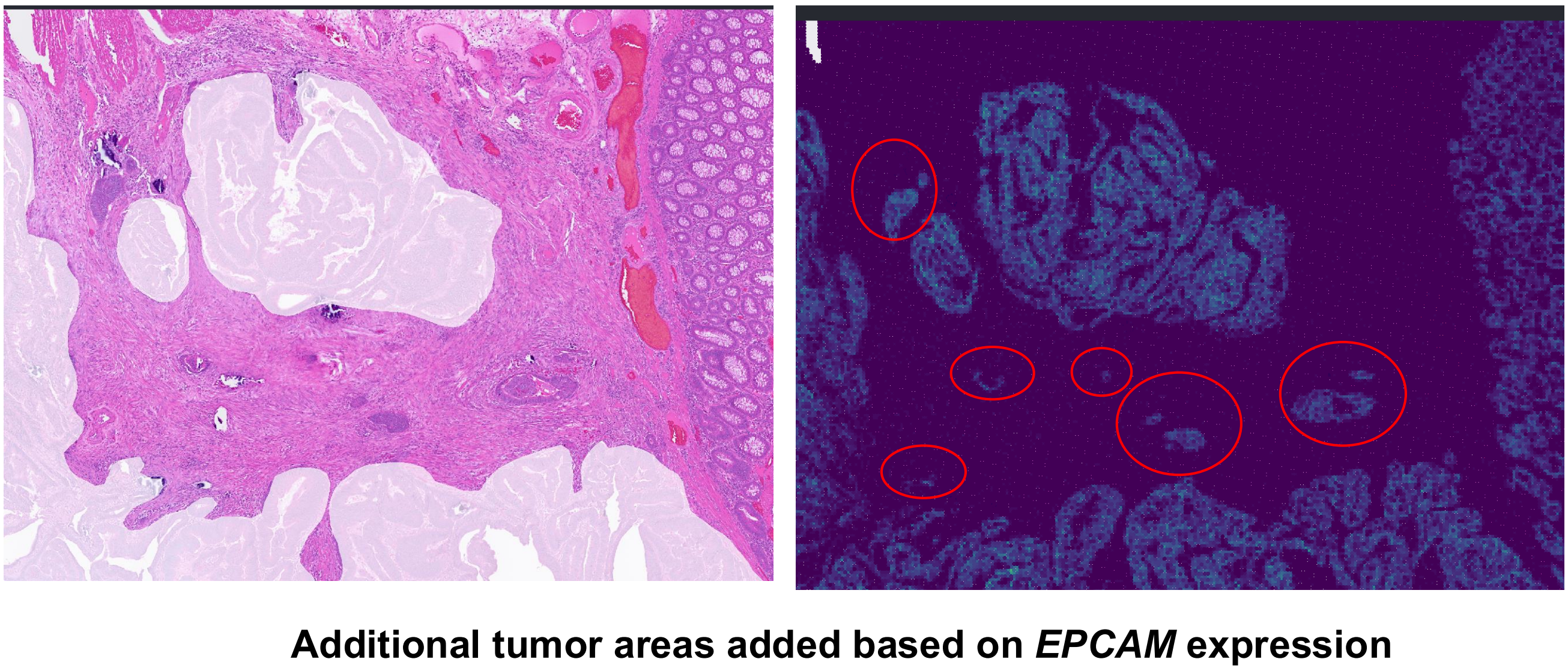

Figure S25: *EPCAM* expression used for fine annotation of the neoplasm. Circles in red were initially missed based solely on the HE

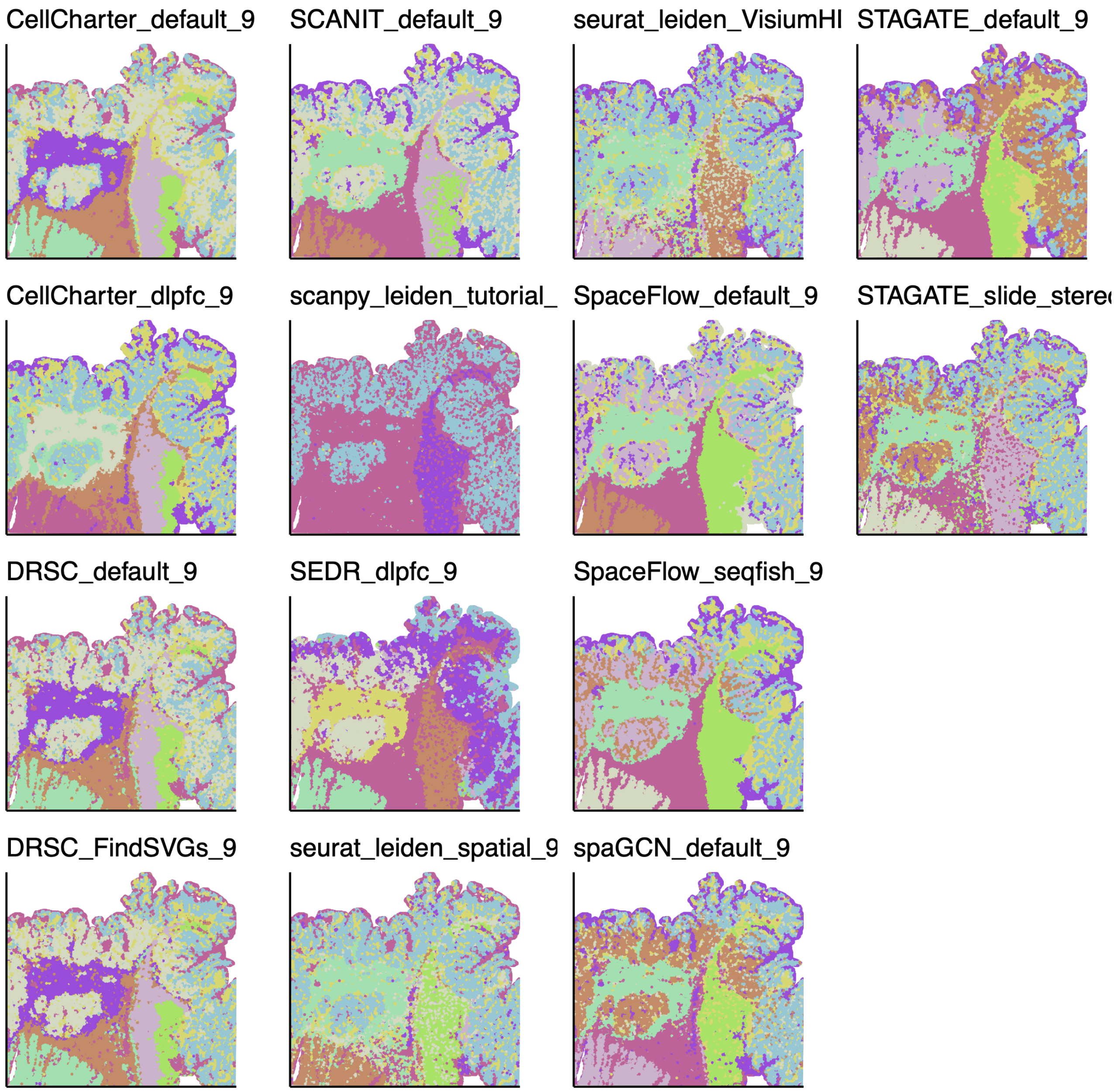

Figure S26: Base clusterings of full Visium HD cancer dataset with 9 clusters for pathologist evaluation.

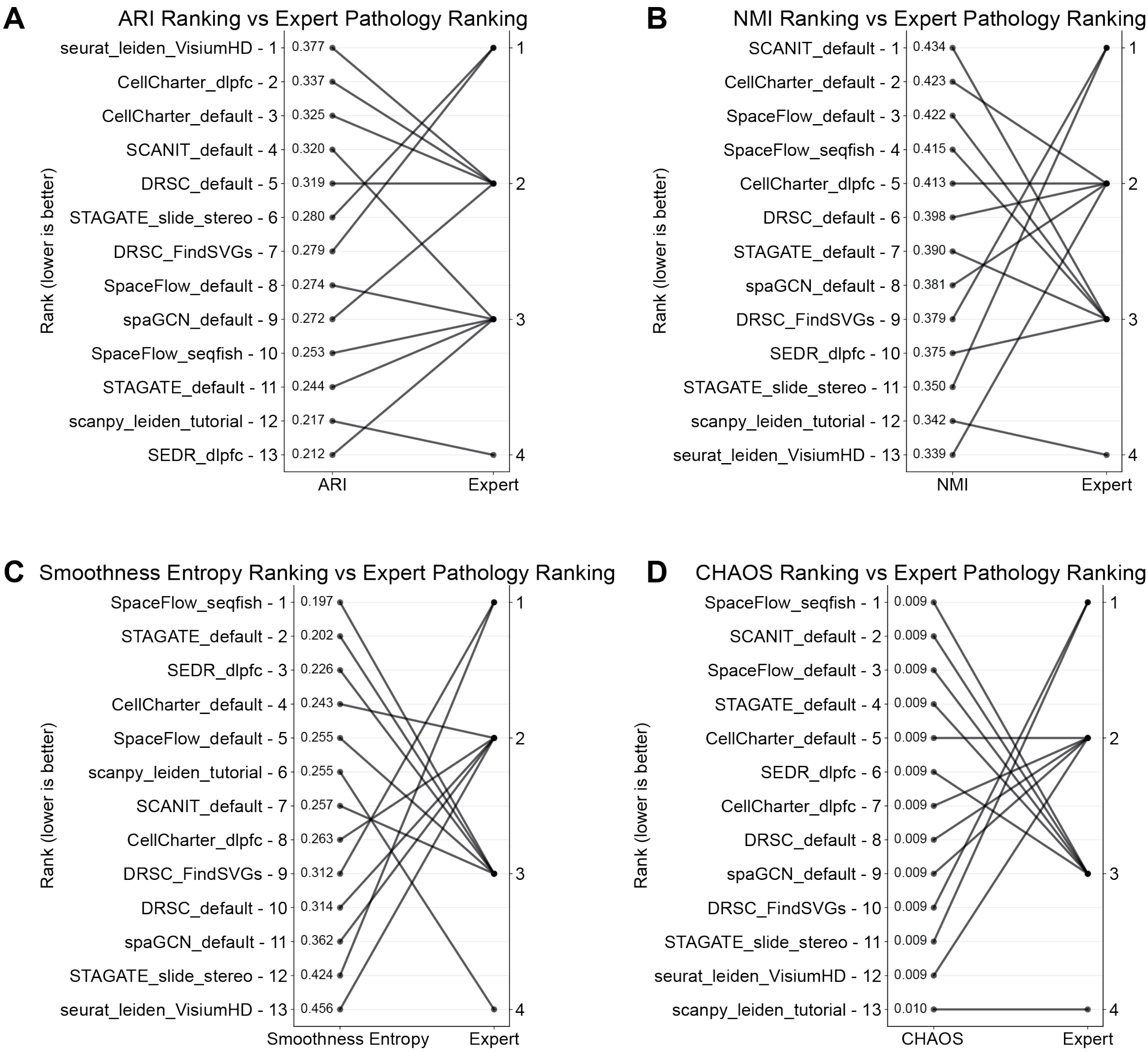

Figure S27: Comparison of expert rankings against evaluation metrics for the Visium HD colorectal cancer sample. Rankings according to **A**. ARI, **B**. NMI, **C**. Smoothness entropy, and **D**. CHAOS vs the expert rankings. The lowest rank is assigned to the best score per metric i.e., highest value for ARI and NMI, and lowest value for Smoothness entropy and CHAOS.

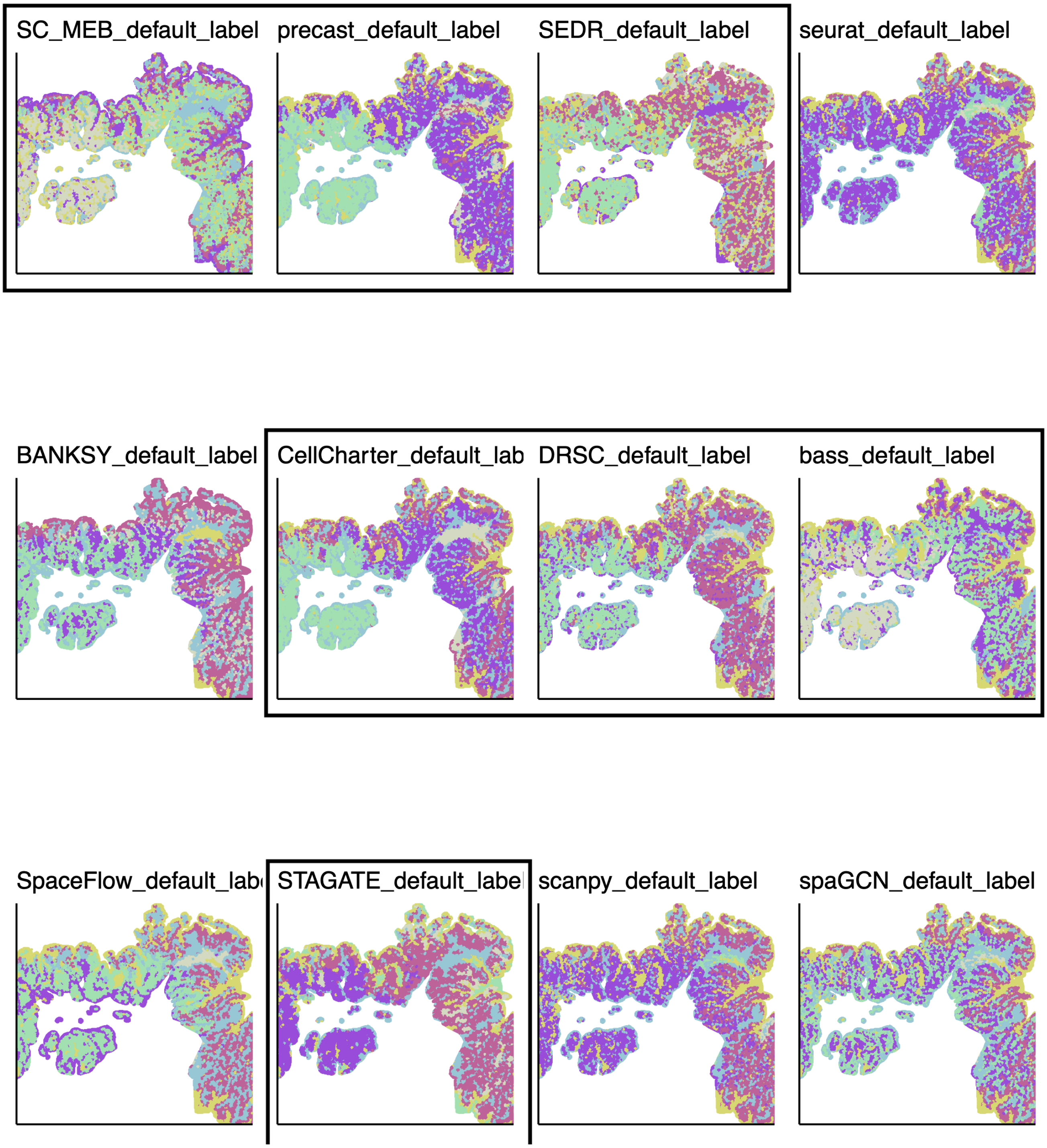

Figure S28: Base clusterings generated for subsetted Visium HD dataset, the instances selected for consensus are enclosed with black boxes.

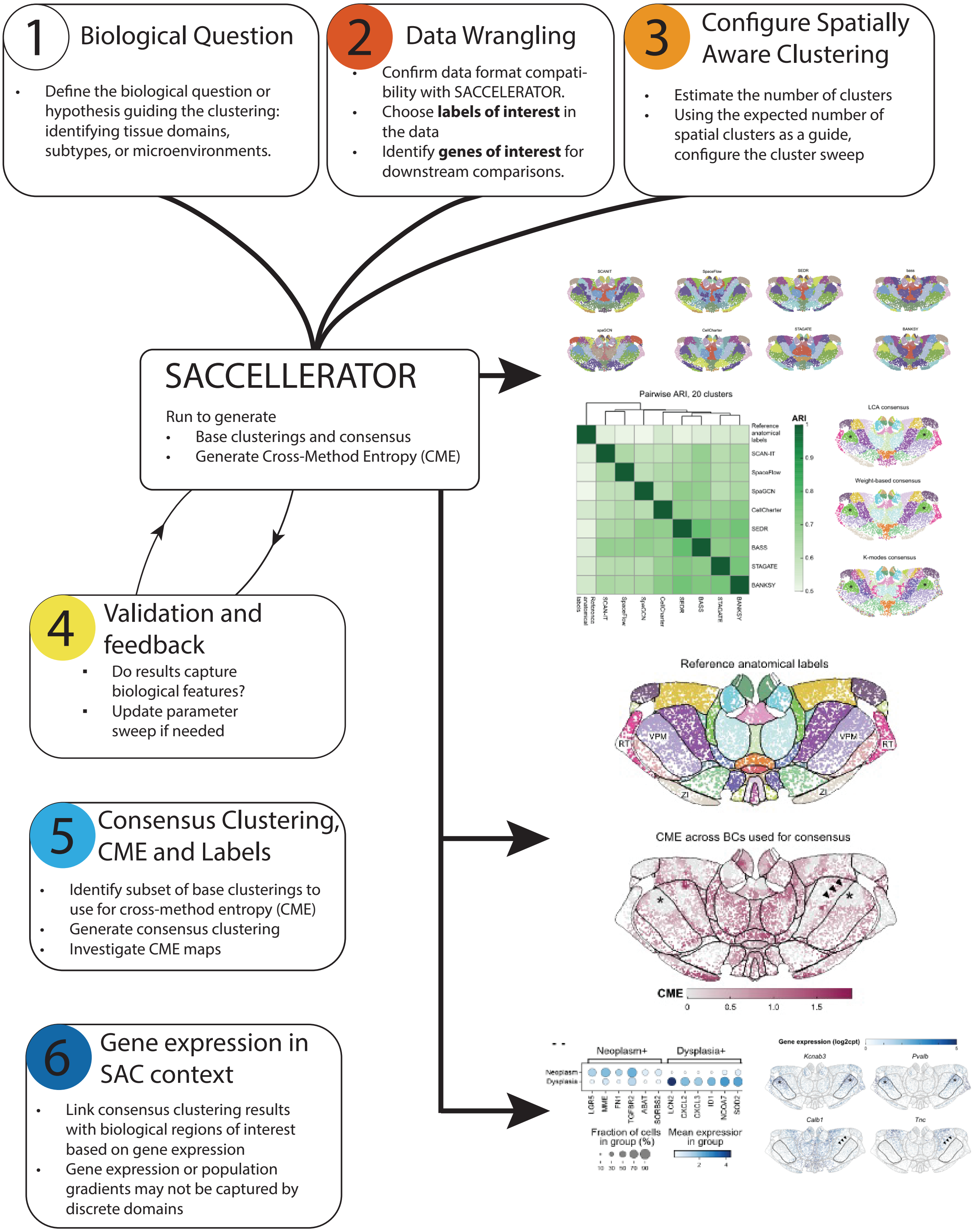

Figure S29: Schematic of the SACCELERATOR consensus guided expert in the loop framework as applied to analysis of the MERFISH mouse brain thalamus dataset (Case study 1). To address issues in identifying biologically relevant regions in spatial transcriptomics data, we adopt a flexible expert-in-the-loop consensus-driven approach. This goes beyond traditional ensemble/consensus methods, and allows researchers to interact with intermediate results to determine which tools should be used to generate a consensus (steps 1-3). Inclusion of an expert-in-the-loop is critical to ensure that the computational analysis matches the biological question at hand, and we believe that when the focus of the analysis is to uncover novel biological discoveries, tissue experts are accessible more often than not (steps 4-6). The figure can also be found in SACCELEATOR GitHub landing page (Code availability).

Supplementary Tables

Table S1 An overview of the datasets used in this study. FF = fresh frozen. FFPE = formalin-fixed paraffin-embedded.

Name	Species	Tissue	Type	Slices	Date	Citation	Technology	Readout	Panel	Resolution	Ground Truth
cosmx_liver	Human	Liver (Normal and HCC)	FFPE	2	2023	Nanostring	CosMx	Imaging	1000	sub-cellular	Computational
cosmx_lung	Human	Lung (NSCLC)	FFPE	5	2022	[73]	CosMx	Imaging	960	sub-cellular	Computational
abc_atlas_wmb_thalamus	Mouse	Brain (thalamus)	FF	59	2023	[53]	MERFISH	Imaging	500	sub-cellular	Manual
merfish_devheart	Human	Heart	FF	4	2024	[74]	MERFISH	Imaging	238	sub-cellular	Computational
STARmap_plus	Mouse	Brain	FF	20	2023	[75]	STARmap PLUS	Imaging	1022	sub-cellular	Manual
xenium-ffpe-bc-idc	Human	Breast	FFPE	1	2023	10X Genomics	Xenium	Imaging	380	sub-cellular	Manual
xenium-mouse-brain-SergioSalas	Mouse	Brain (coronal section)	FF	1	2023	10X Genomics	Xenium	Imaging	247	sub-cellular	Manual
slideseq2_olfactory_bulb	Mouse	Brain (olfactory bulb)	FF	20	2022	[78]	Slide-seqV2	Sequencing	N/A	single-cell	Manual
stereoseq_liver	Mouse	Liver	FF	6	2024	[44]	Stereo-seq	Sequencing	N/A	single-cell / sub-cellular	Computational
stereoseq_mouse_embryo	Mouse	Embryo	FF	53	2022	[13]	Stereo-seq	Sequencing	N/A	single-cell / sub-cellular	Computational
libd_dlpfc	Human	Brain (dorosolateral prefrontal cortex)	FF	12	2021	[33]	Visium	Sequencing	N/A	mini-bulk	Manual
osmfish_Ssp	Mouse	Brain (SSp)	FF	1	2018	[79]	osmFISH	Imaging	33	sub-cellular	Computational
visium_breast_cancer_SEDR	Human	Breast (invasive ductal carcinoma)	FF	1	2020	[94]	Visium	Sequencing	N/A	mini-bulk	Manual
visium_chicken_heart	Chicken	Heart	FF	11	2021	[80]	Visium	Sequencing	N/A	mini-bulk	Manual
Visium_hd_cancer_colon	Human	Colon (CRC)	FFPE	1	2024	[54]	Visium HD	Sequencing	N/A	single-cell / sub-cellular	Manual

Table S2 Overview of methods. A GitHub commit is only included for methods installed from GitHub without releases.

Name	Version (commit)	Year	Language	Citation
MAPLE	0.99.1 (b173e89)	2024	R	Jeon et al. (2024)
BANKSY	0.99.7 (dbda6fd)	2023	R (+ Python)	Singhal et al. (2023)
PRECAST	1.6.3	2023	R	Liu et al. (2023)
GraphST	1.1.1	2023	Python	Long et al. (2023)
SPICEMIX	0.0.71	2023	Python	Chidester et al. (2023)
CellCharter	0.2.0	2023	Python	Varrone et al. (2023)
STAGATE	1.0.1 (48ce7f8)	2022	Python	Dong & Zhang (2022)
scMEB	1.1	2022	R	Yang et al. (2022)
SpaceFlow	1.0.4	2022	Python	Ren et al. (2022)
DR-SC	3.3	2022	R	Liu et al. (2022)
SCAN-IT	0.1 (ebf3894)	2022	Python	Cang et al. (2022)
SOTIP	1.0 (d3b762c)	2022	Python	Yuan et al. (2022)
Stardust	2.0 (f1b5417)	2022	R	Avesani et al. (2022)
BASS	1.1.0.016 (37980c9)	2022	R	Li & Zhou (2022)
spatialGE	1.2.0.0000	2022	R	Ospina et al. (2022)
SpaGCN	1.2.7	2021	Python	Hu et al. (2021)
BayesSpace	1.10.1	2021	R	Zhao et al. (2021)
SEDR	1.0.0 (8c273af)	2021	Python	Fu et al. (2021)
MERINGUE	1.0 (ca9e2cc)	2021	R	Miller et al. (2021)
Giotto	4.0.0	2021	R (+ Python)	Dries et al. (2021)
Scanpy	1.10.0	2018	Python	Wolf et al. (2018)
Seurat	5.0.1	2018	R	Butler et al. (2018)

Table S3 Overview of method configurations.

Table S4 Overview of metrics.

Name	Requires GT labels	Uses spatial information	Language	Citation
Adjusted Rand Index (ARI)	yes	no	Python	Hubert & Arabie (1985)
Spatial chaos score (CHAOS)	no	yes	R	Shang & Zhou (2022)
Calinski–Harabasz index	yes	no	Python	Calinski & Harabasz (1974)
Completeness	yes	no	Python	Rosenberg & Hirschberg (2007)
Davies–Bouldin index	no	no	Python	Davies & Bouldin (1979)
Entropy	yes	no	Python	Shannon (1948)
Fowlkes–Mallows Index (FMI)	yes	no	Python	Fowlkes & Mallows (1983)
Homogeneity	yes	no	Python	Rosenberg & Hirschberg (2007)
Local Inverse Simpson's Index (LISI)	yes	no	R	Korsunsky et al. (2019)
Mathew's Correlation Coefficient (MCC)	yes	no	Python	Chicco & Jurman (2020)
Normalized Mutual Information (NMI)	yes	no	R	Strehl & Ghosh (2002)
Percentage of Abnormal Spots (PAS)	no	yes	R	Shang & Zhou (2022)
SpatialARI	yes	yes	R	Luo et al. (2025)
V measure	yes	no	Python	Rosenberg & Hirschberg (2007)
Cluster specific silhouette	no	no	R	Rousseeuw (1987)
Domain specific F1	yes	no	R	Rijsbergen (1979)
Jaccard index	yes	no	Python	Jaccard (1912)

Supplementary Files

Supplementary File 1: SACCELERATOR Checklist

**Biological Question**	• **Define the biological question** or hypothesis guiding the clustering: identifying tissue domains, subtypes, or microenvironments. For example:<br>○ Does spatial transcriptomics support subdividing anatomical regions of the thalamus?<br>○ Can spatial transcriptomics identify neoplastic progression stages in colorectal cancer?
**Data Wrangling**	• Confirm **data format compatibility** with SACCELERATOR.<br>• Identify **labels of interest** already in the data: manual annotations and cell type labels can be used for comparison and validation.<br>• Identify **genes of interest** for downstream comparisons.
**Configure Spatially Aware Clustering**	• **Estimate** how many spatial clusters are present in the tissue<br>• Using the **expected number of spatial clusters** as a guide, configure the cluster sweep to span at least ±5 numbers of clusters around the expected number.<br>• **Run** all, or selected methods suitable for the data. By default the downstream consensus workflow will select the 8 most concordant methods based on cross-method ARI. This will generate the base clusterings
**Validation and Feedback**	• **Review SACCELERATOR outputs**: and compare to biologically relevant structures.<br>○ Validate scale and number of clusters in the context of the biological topic.<br>○ Does one base clustering result address biological questions?<br>○ For example, in the brain: do some outputs include laminar organization of cortex? In cancer cases, do some outputs correctly identify tumor tissue?<br>• If biological features are not included, **review SACCELLERATOR configuration** and broaden the parameter sweep.
**Consensus Clustering, CME and Reference Labels**	• If needed, identify the **subset of base clusterings** to use for cross-method entropy (CME), e.g., filtering base clusterings based on spatial continuity measures (such as smoothness entropy).<br>• Generate **visual summaries** (spatial consensus plot, cross-method entropy map, weight distribution plot, pairwise ARI heatmaps with clustering method class) to accompany expert interpretation.<br>• Using CME heatmap and consensus, check if any single base **clustering method/class dominates the consensus**.<br>• Verify that spatial resolution and granularity of the **final consensus** includes the biological scales of interest.<br>• **Interpretation of CME maps:**<br>○ Identify low entropy areas, where labels are consistent across methods.<br>○ Areas of high entropy often correspond to boundaries between regions where multiple methods give slightly different results.
**Gene Expression in SAC context**	• **Review gene expression** in the context of consensus clustering.<br>○ Link **consensus clustering results with biological regions of interest** based on gene expression.<br>○ Gene expression or population **gradients may not be captured by discrete domains**.<br>○ Do genes of interest show differential expression between consensus clusters?
**Important Notes**	• SACCELLERATOR assumes cellular **quality control** has already been performed.<br>• Even within a single tissue sample, genes of interest, relevant length scales, and expected numbers of clusters can **vary based on biological questions**. Independent runs of SACCELERATOR may be required to address different biological questions.<br>• We recommend a discussion to **review interpretations** of the consensus clusterings, including, for example: choice of a single consensus output or application of CME to identify domain boundaries.

Supplementary File 2: CellCharter Xenium Mouse Brain Cluster Granularity Scan (k=5-49)

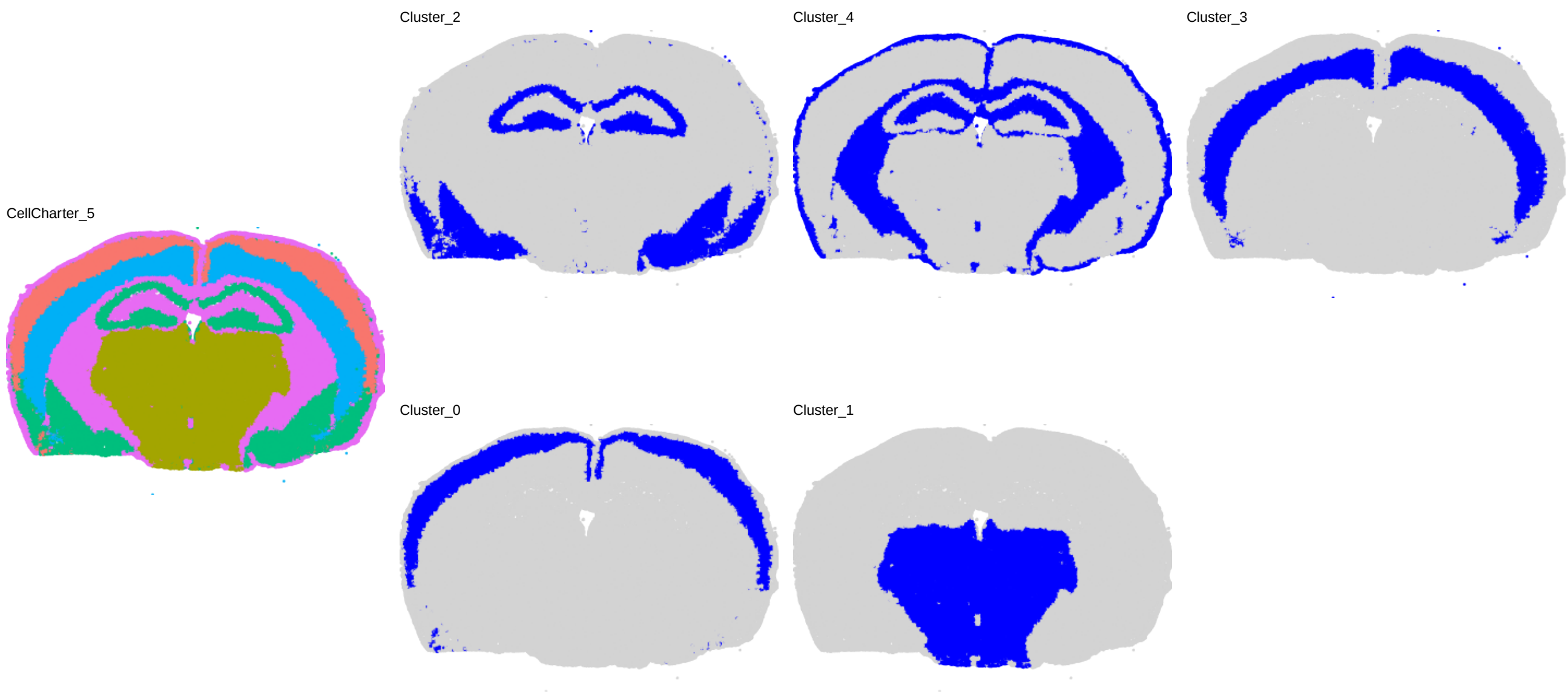

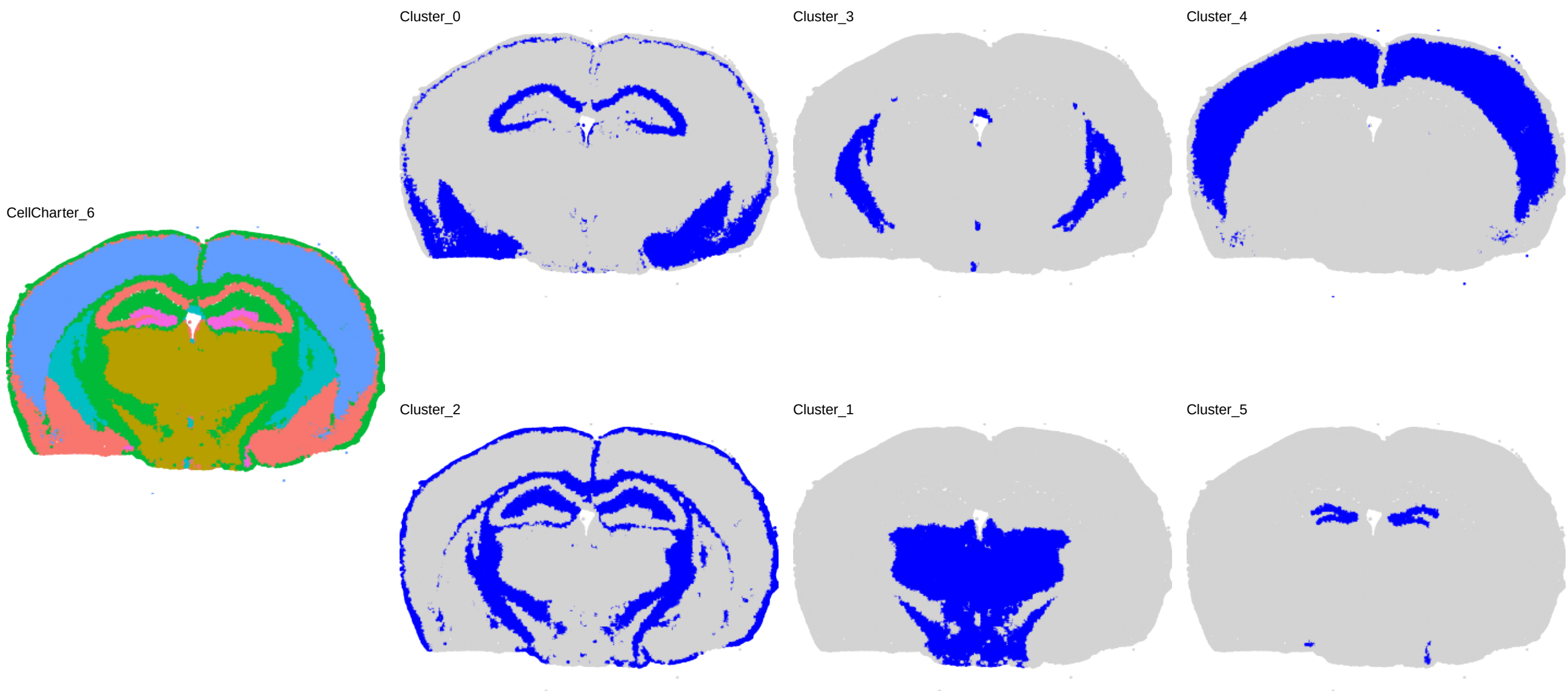

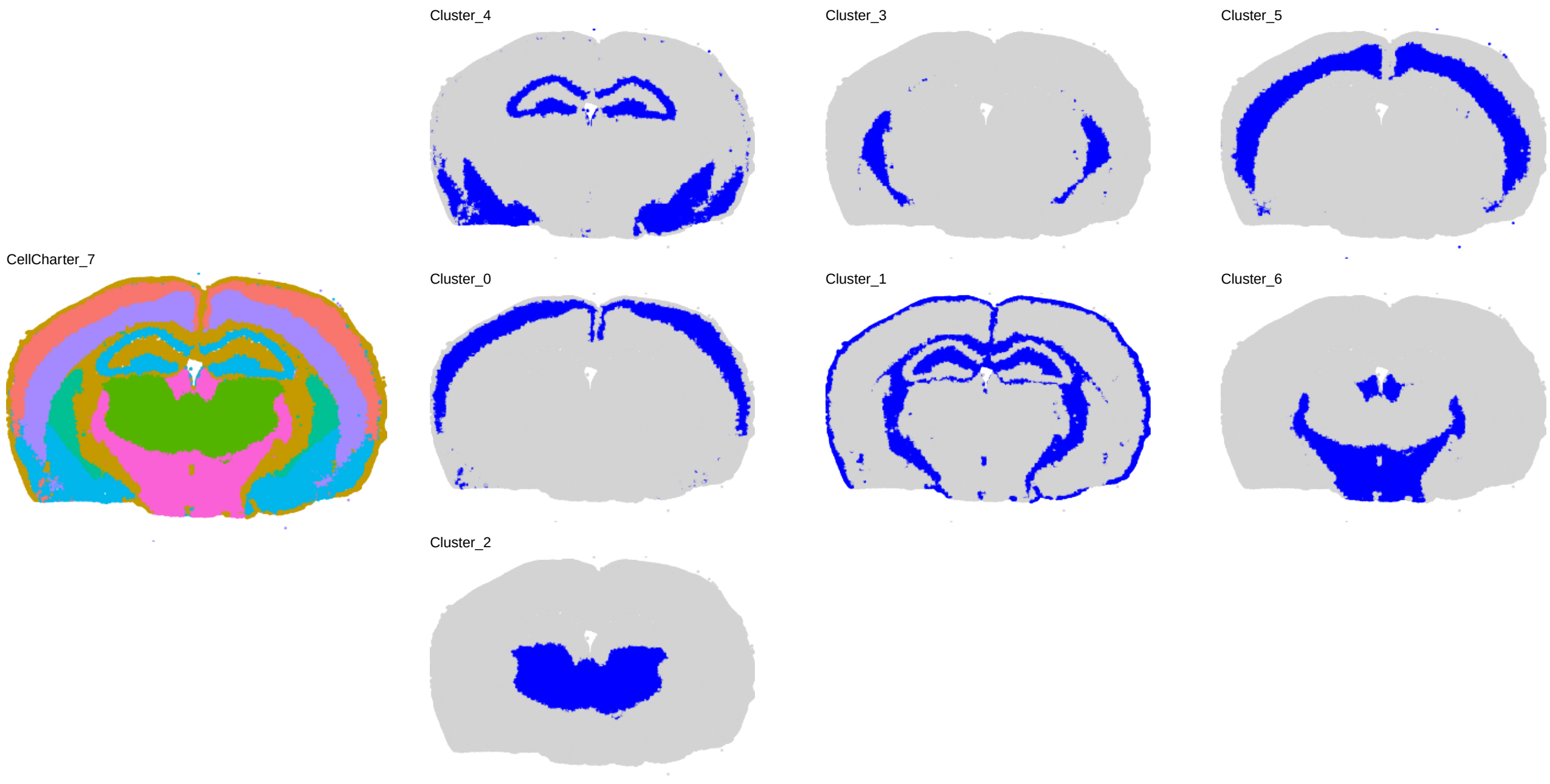
CellCharter_7
Cluster_4
Cluster_3
Cluster_5
Cluster_0
Cluster_1
Cluster_6
Cluster_2

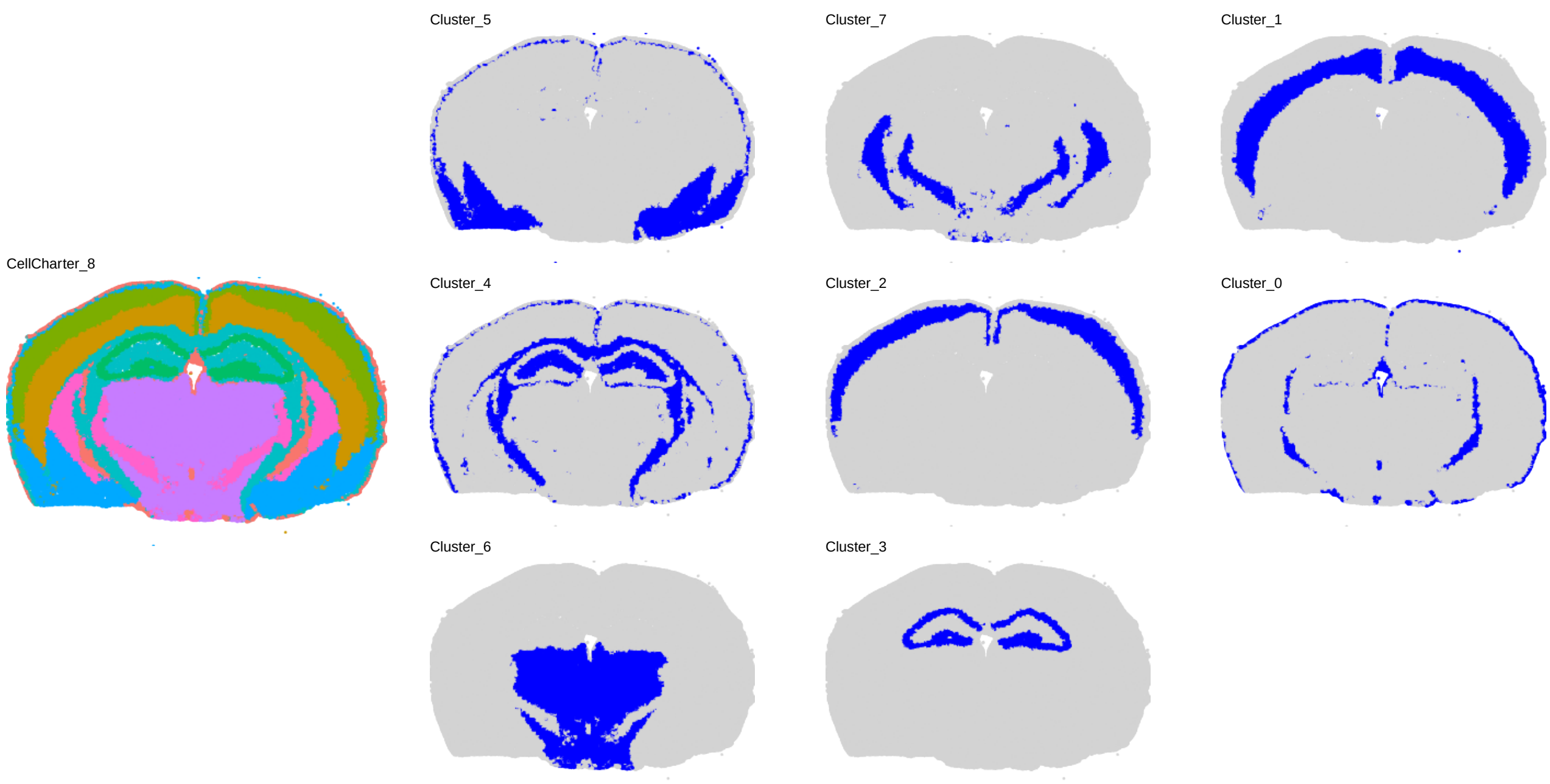
CellCharter_8
Cluster_5
Cluster_7
Cluster_1
Cluster_4
Cluster_2
Cluster_0
Cluster_6
Cluster_3

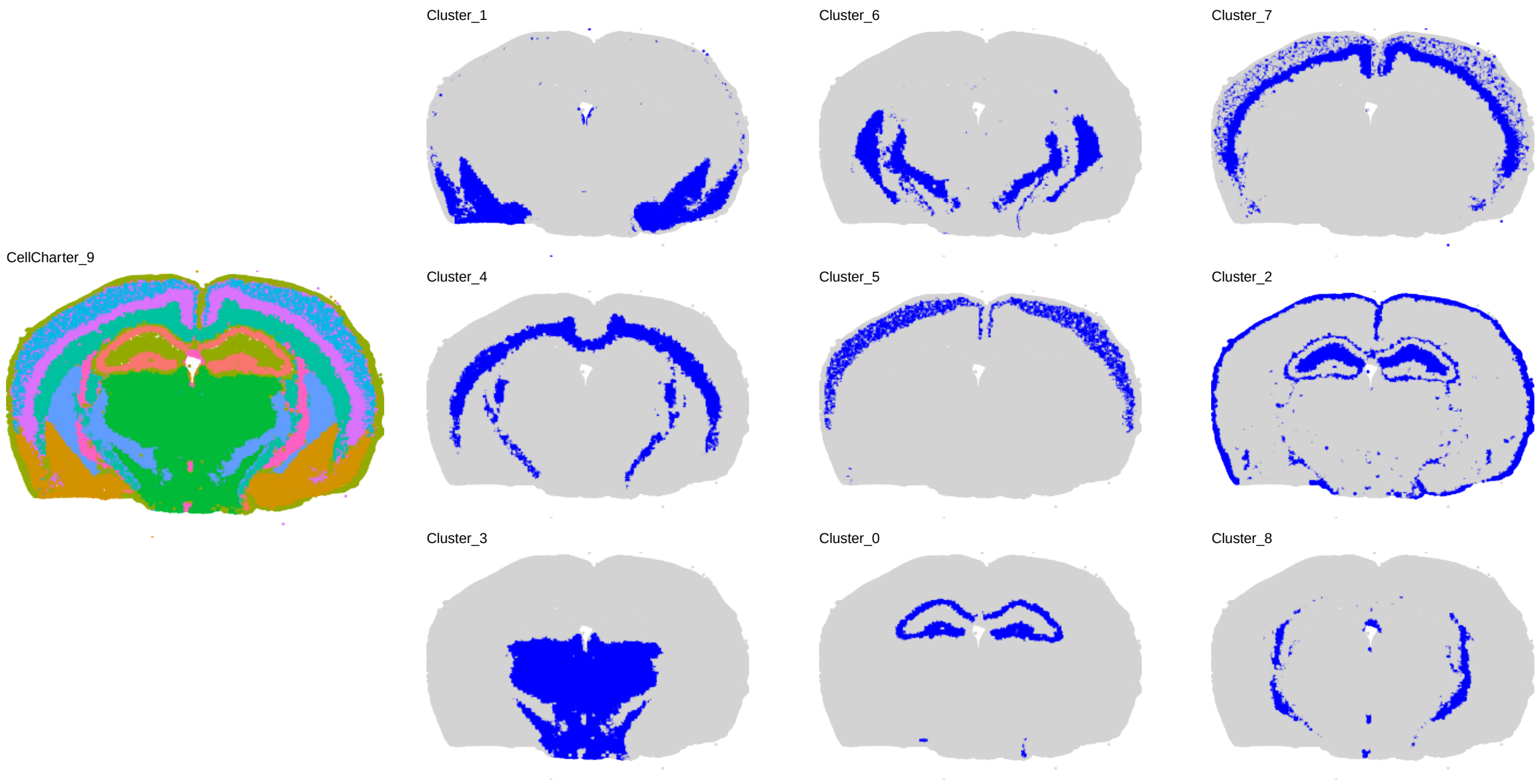
CellCharter_9
Cluster_1
Cluster_6
Cluster_7
Cluster_4
Cluster_5
Cluster_2
Cluster_3
Cluster_0
Cluster_8

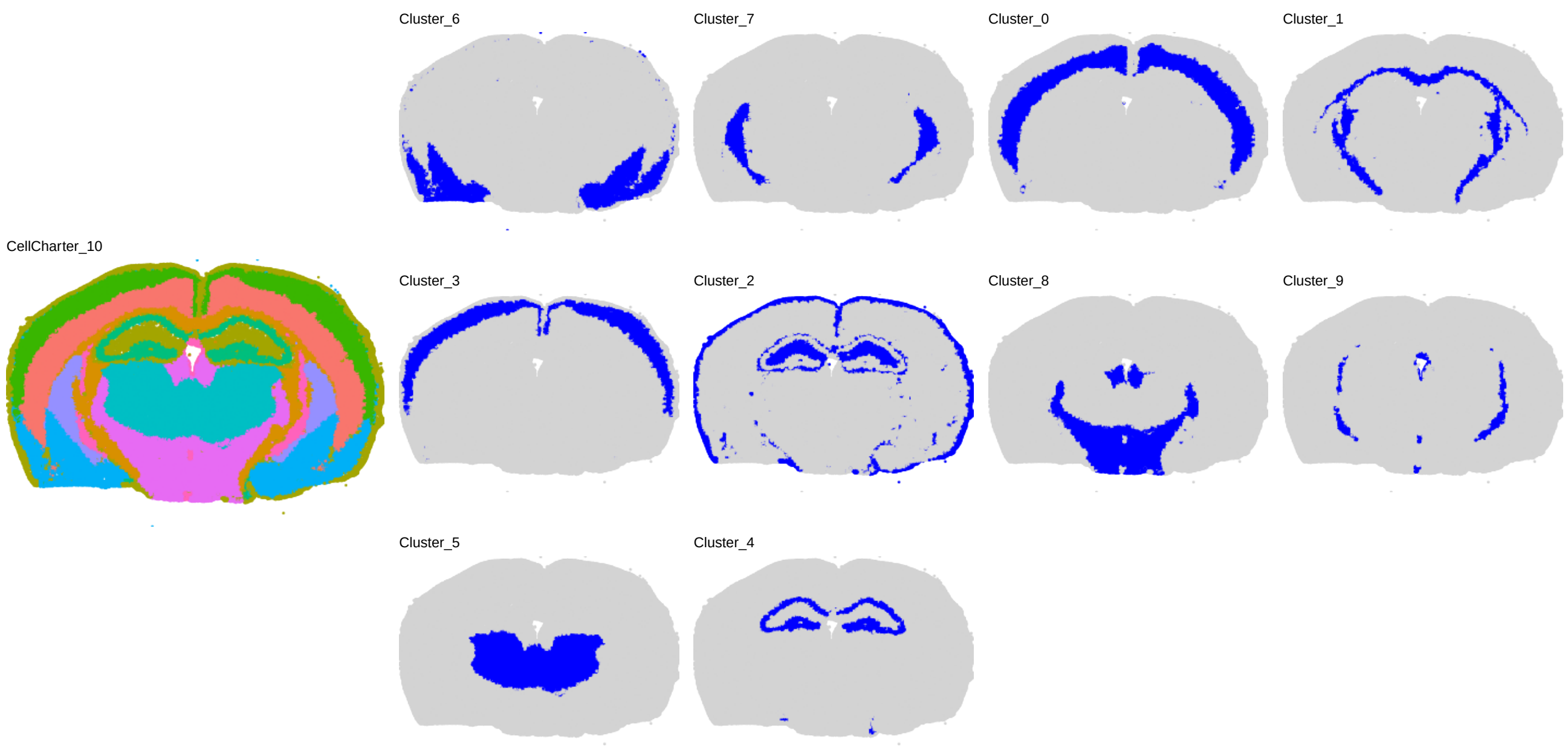
CellCharter_10
Cluster_6
Cluster_7
Cluster_0
Cluster_1
Cluster_3
Cluster_2
Cluster_8
Cluster_9
Cluster_5
Cluster_4

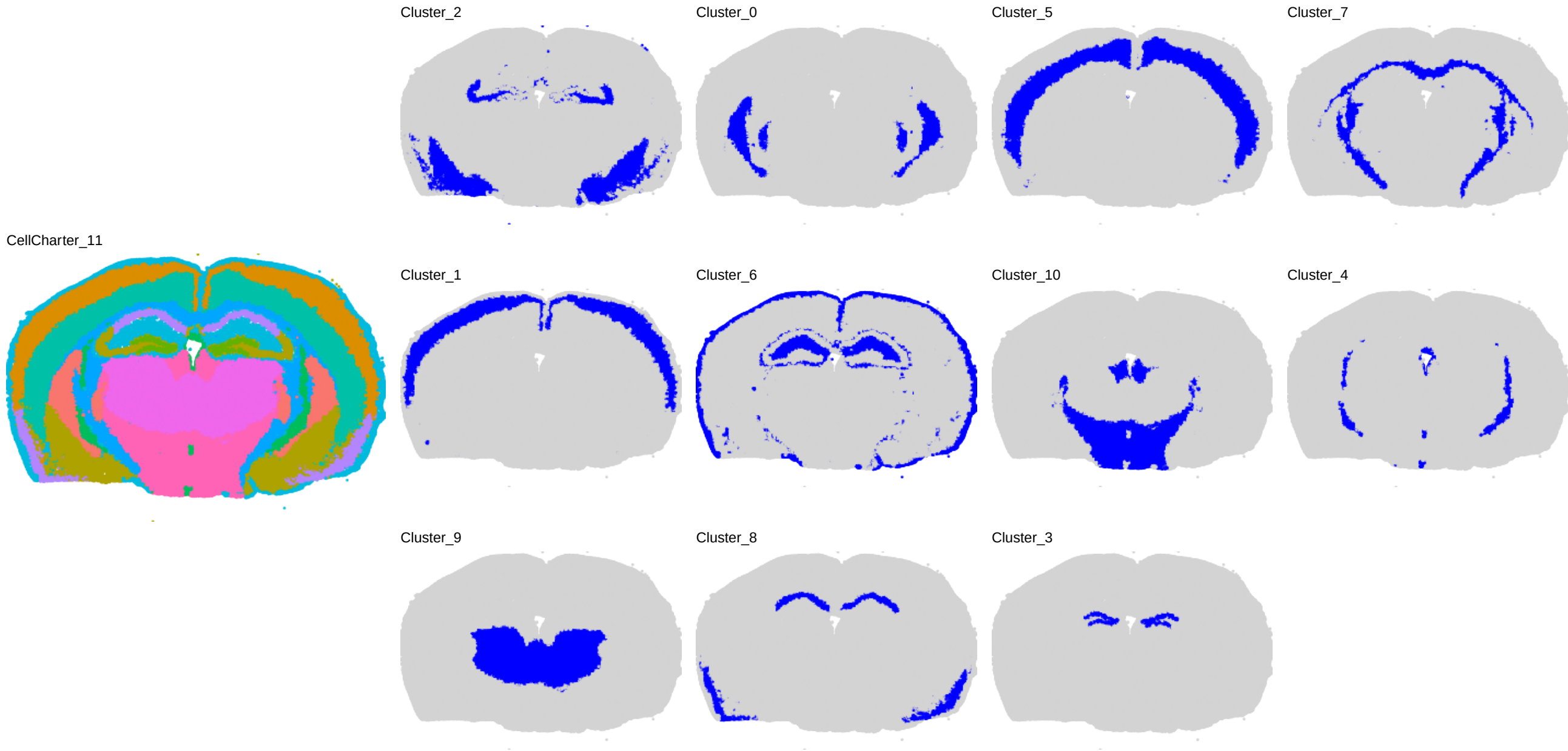
CellCharter_11
Cluster_2
Cluster_0
Cluster_5
Cluster_7
Cluster_1
Cluster_6
Cluster_10
Cluster_4
Cluster_9
Cluster_8
Cluster_3

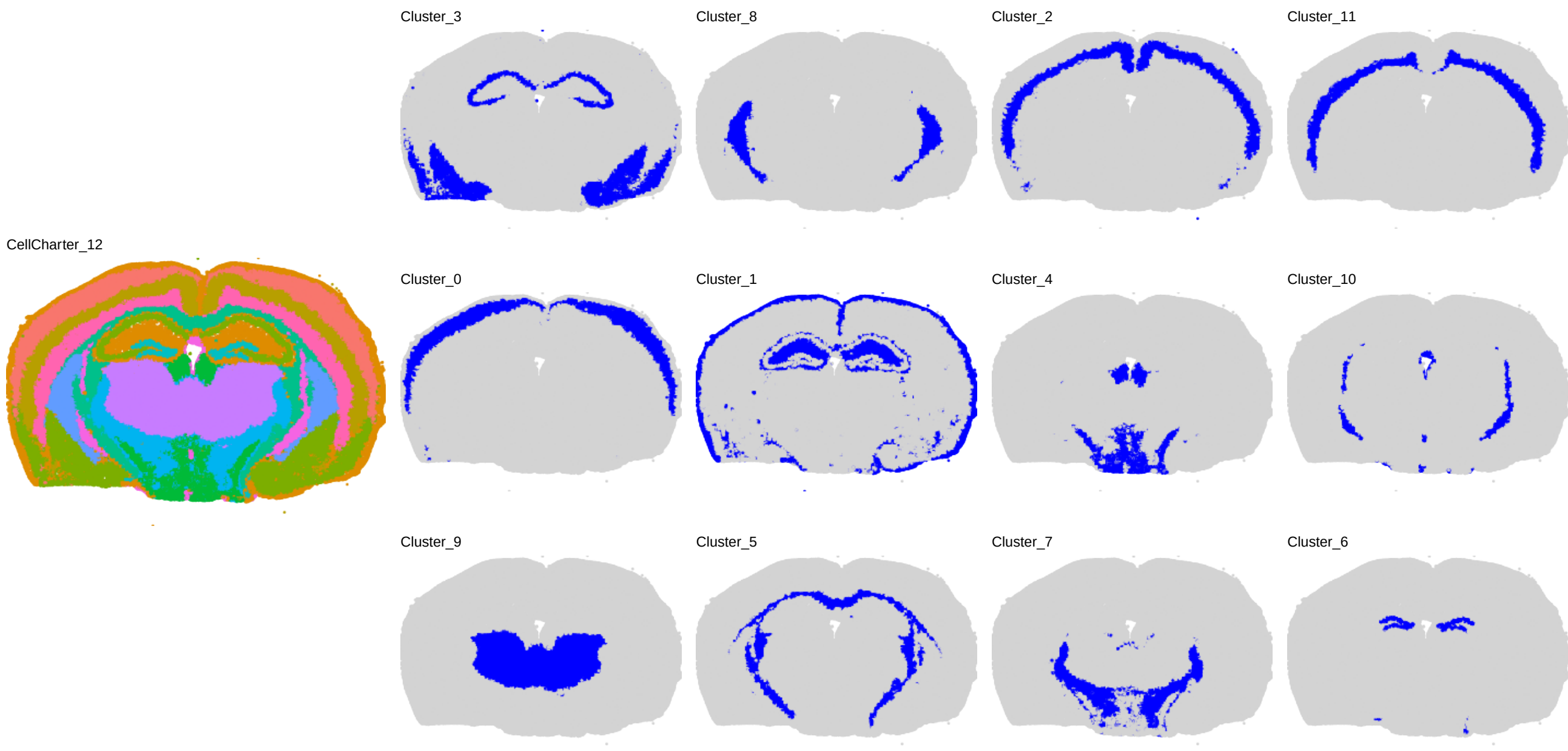
CellCharter_12
Cluster_3
Cluster_8
Cluster_2
Cluster_11
Cluster_0
Cluster_1
Cluster_4
Cluster_10
Cluster_9
Cluster_5
Cluster_7
Cluster_6

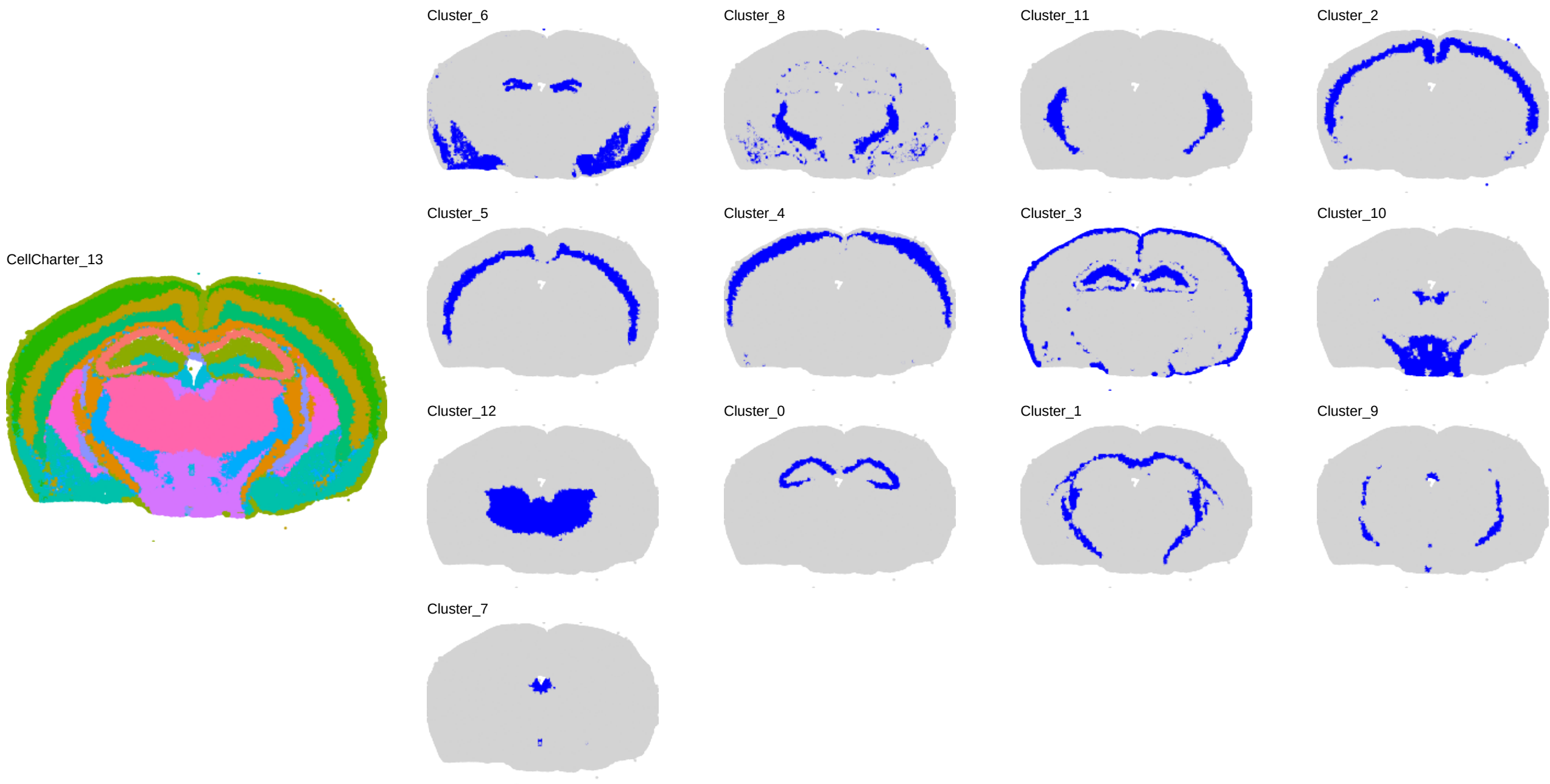
CellCharter_13
Cluster_6
Cluster_8
Cluster_11
Cluster_2
Cluster_5
Cluster_4
Cluster_3
Cluster_10
Cluster_12
Cluster_0
Cluster_1
Cluster_9
Cluster_7

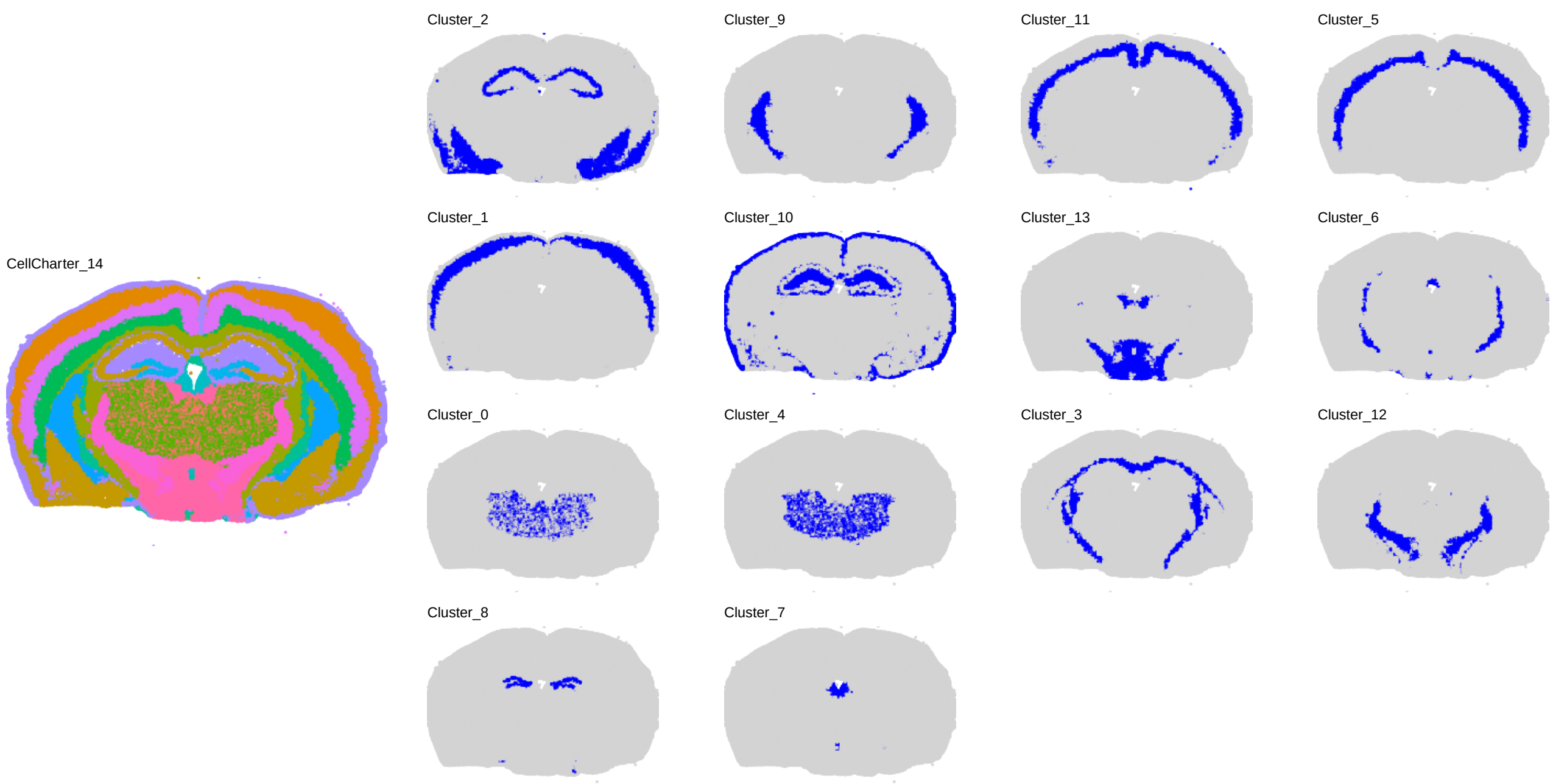
CellCharter_14
Cluster_2
Cluster_9
Cluster_11
Cluster_5
Cluster_1
Cluster_10
Cluster_13
Cluster_6
Cluster_0
Cluster_4
Cluster_3
Cluster_12
Cluster_8
Cluster_7

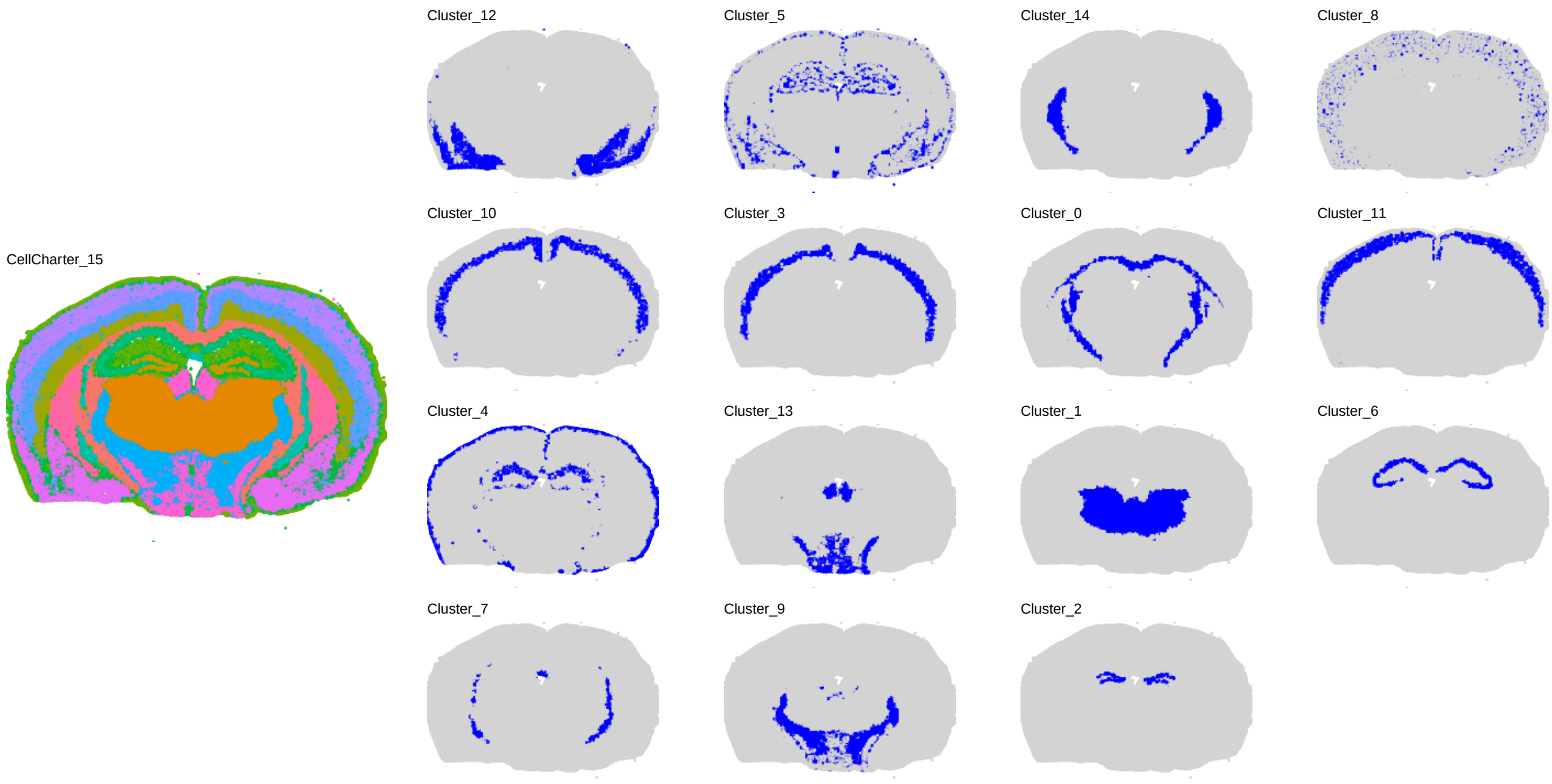
CellCharter_15
Cluster_12
Cluster_5
Cluster_14
Cluster_8
Cluster_10
Cluster_3
Cluster_0
Cluster_11
Cluster_4
Cluster_13
Cluster_1
Cluster_6
Cluster_7
Cluster_9
Cluster_2

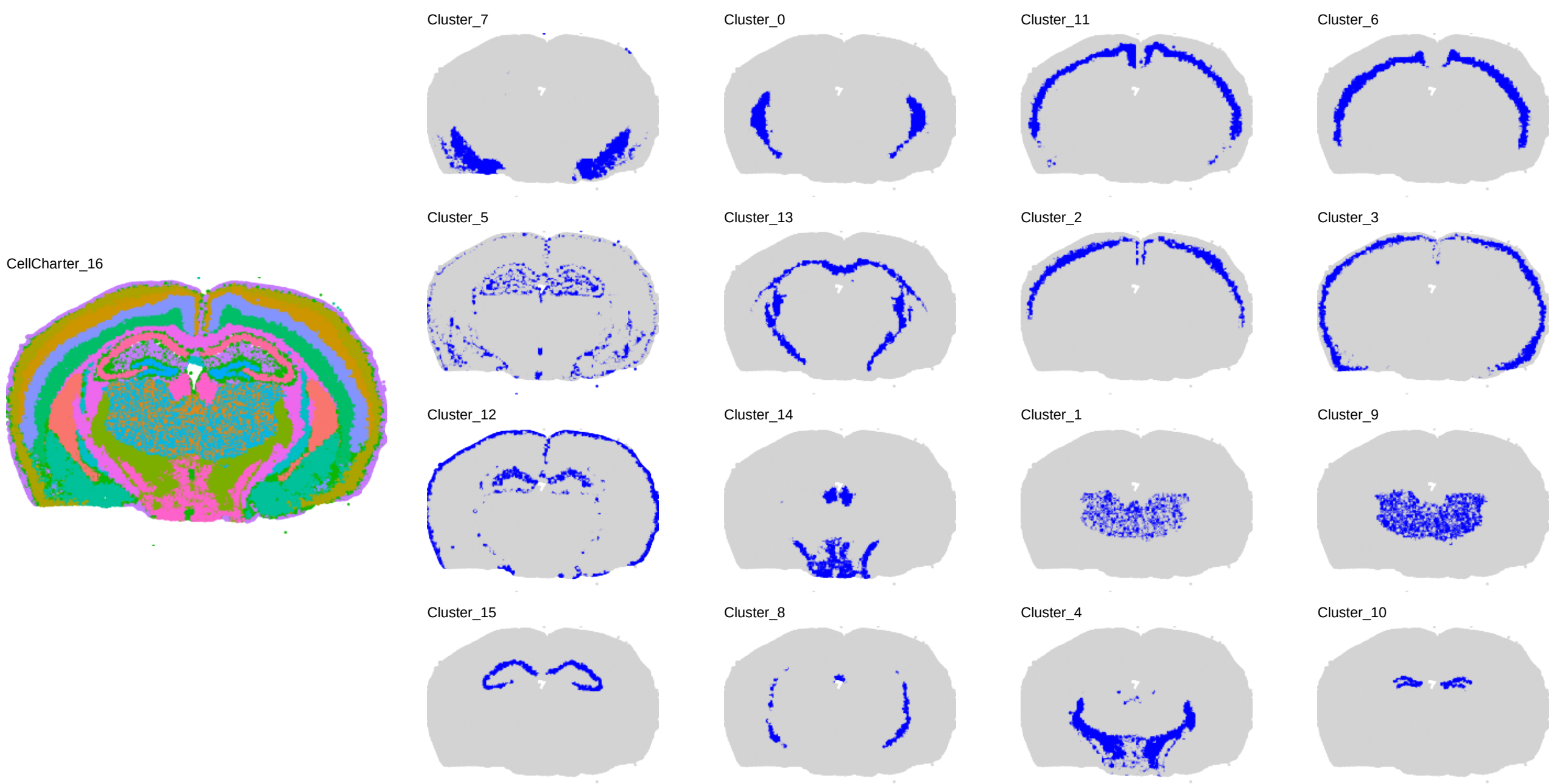
CellCharter_16
Cluster_7
Cluster_0
Cluster_11
Cluster_6
Cluster_5
Cluster_13
Cluster_2
Cluster_3
Cluster_12
Cluster_14
Cluster_1
Cluster_9
Cluster_15
Cluster_8
Cluster_4
Cluster_10

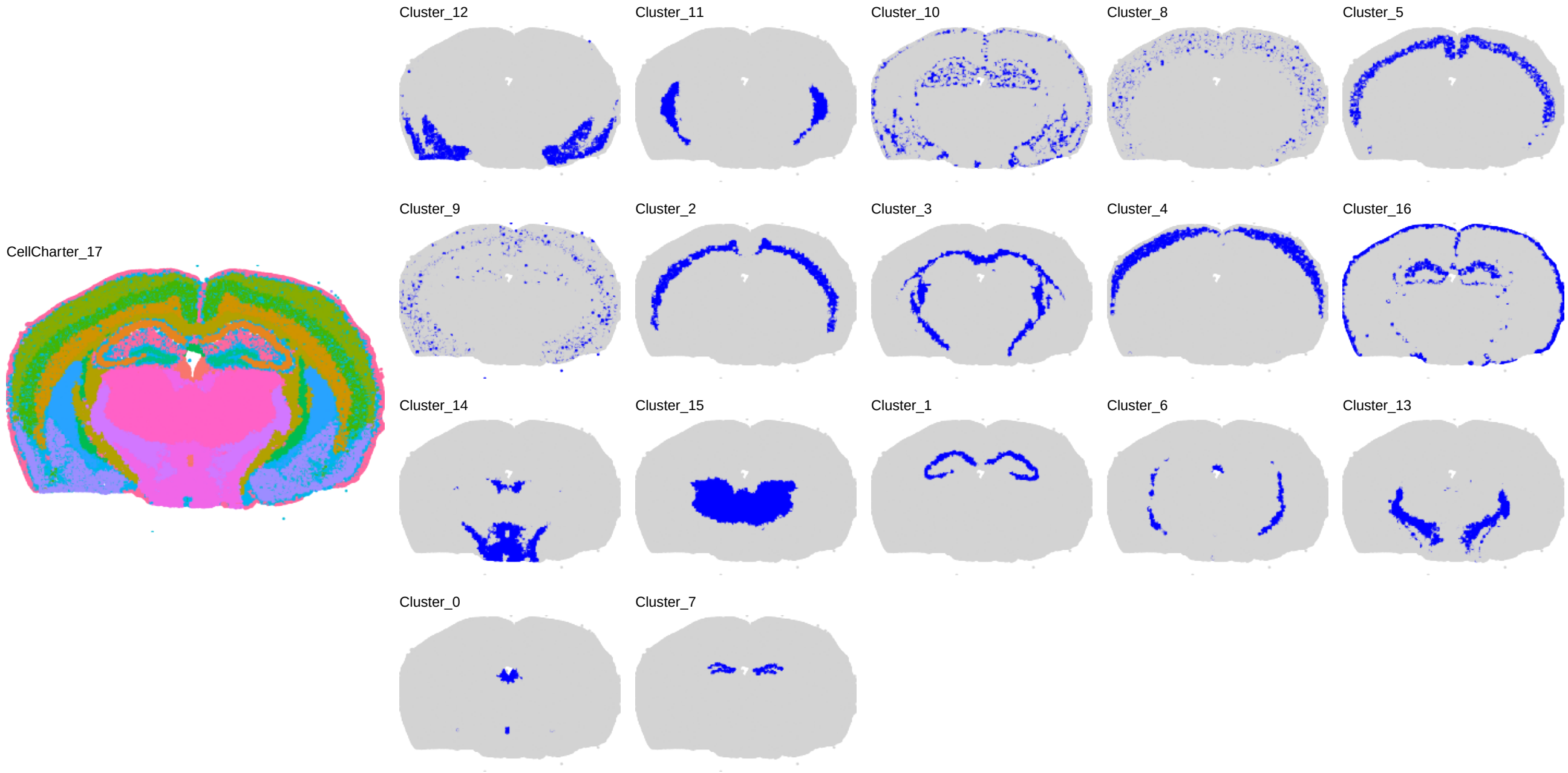
CellCharter_17
Cluster_12
Cluster_11
Cluster_10
Cluster_8
Cluster_5
Cluster_9
Cluster_2
Cluster_3
Cluster_4
Cluster_16
Cluster_14
Cluster_15
Cluster_1
Cluster_6
Cluster_13
Cluster_0
Cluster_7

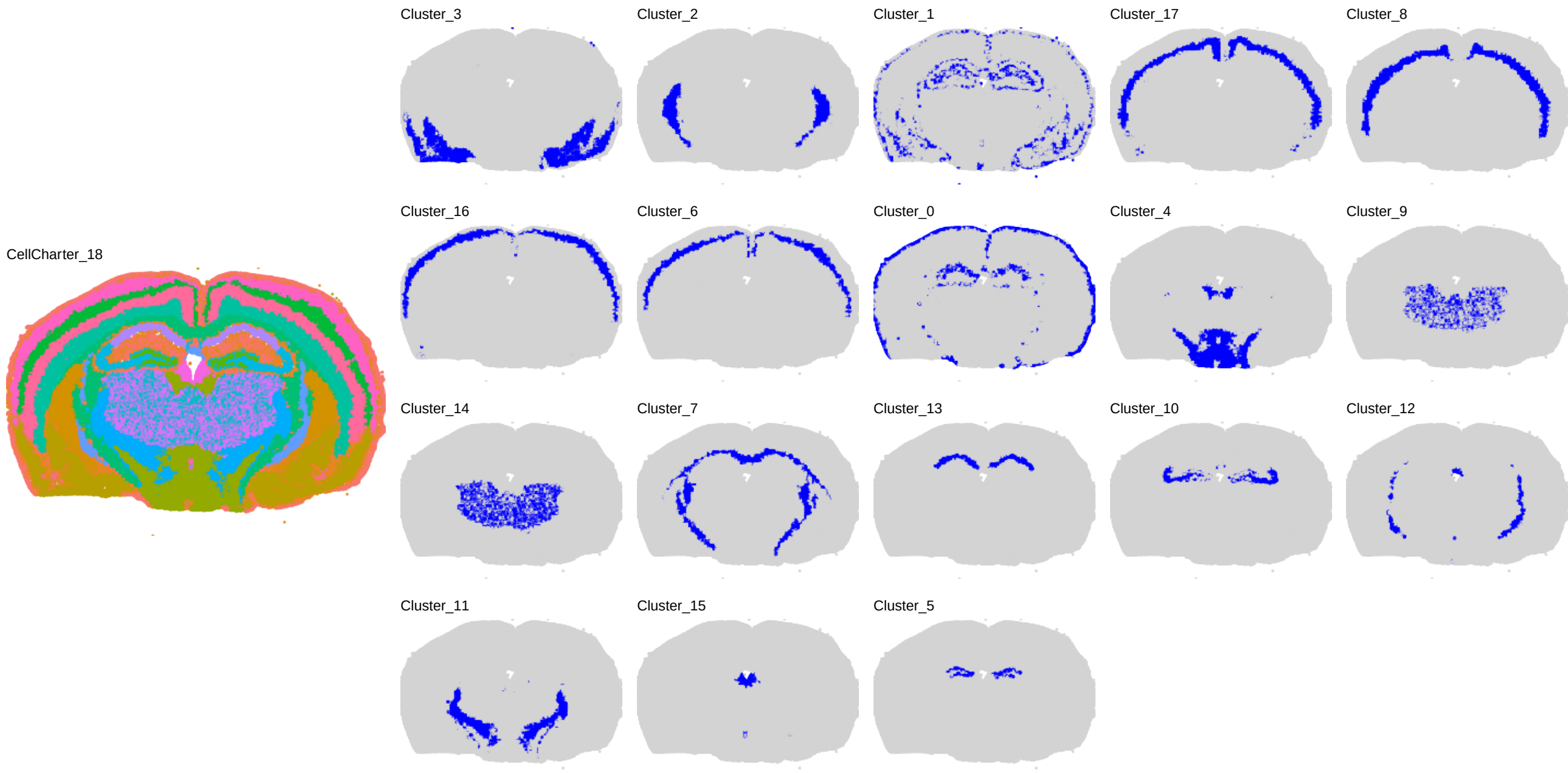
CellCharter_18
Cluster_3
Cluster_2
Cluster_1
Cluster_17
Cluster_8
Cluster_16
Cluster_6
Cluster_0
Cluster_4
Cluster_9
Cluster_14
Cluster_7
Cluster_13
Cluster_10
Cluster_12
Cluster_11
Cluster_15
Cluster_5

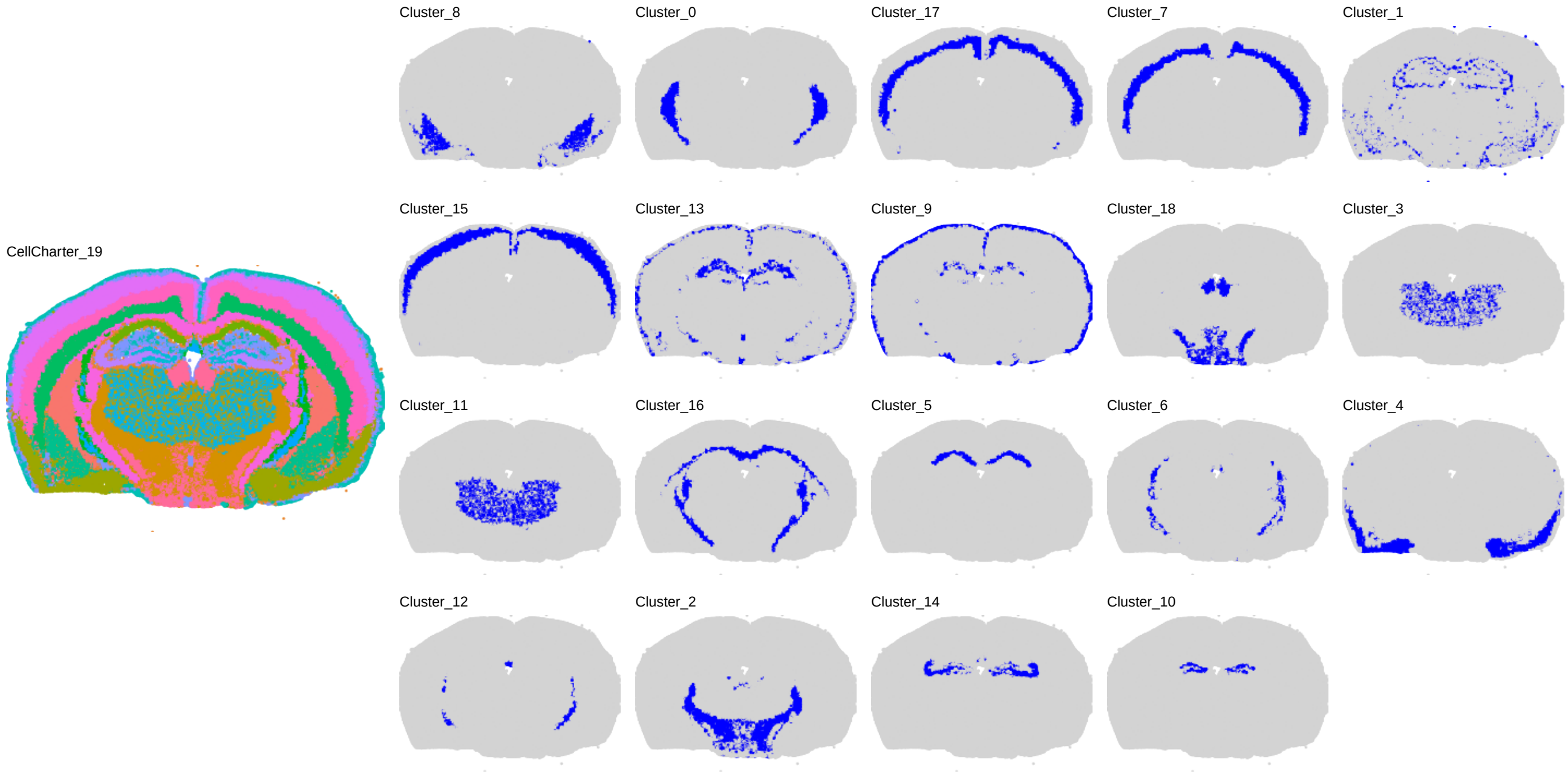
CellCharter_19
Cluster_8
Cluster_0
Cluster_17
Cluster_7
Cluster_1
Cluster_15
Cluster_13
Cluster_9
Cluster_18
Cluster_3
Cluster_11
Cluster_16
Cluster_5
Cluster_6
Cluster_4
Cluster_12
Cluster_2
Cluster_14
Cluster_10

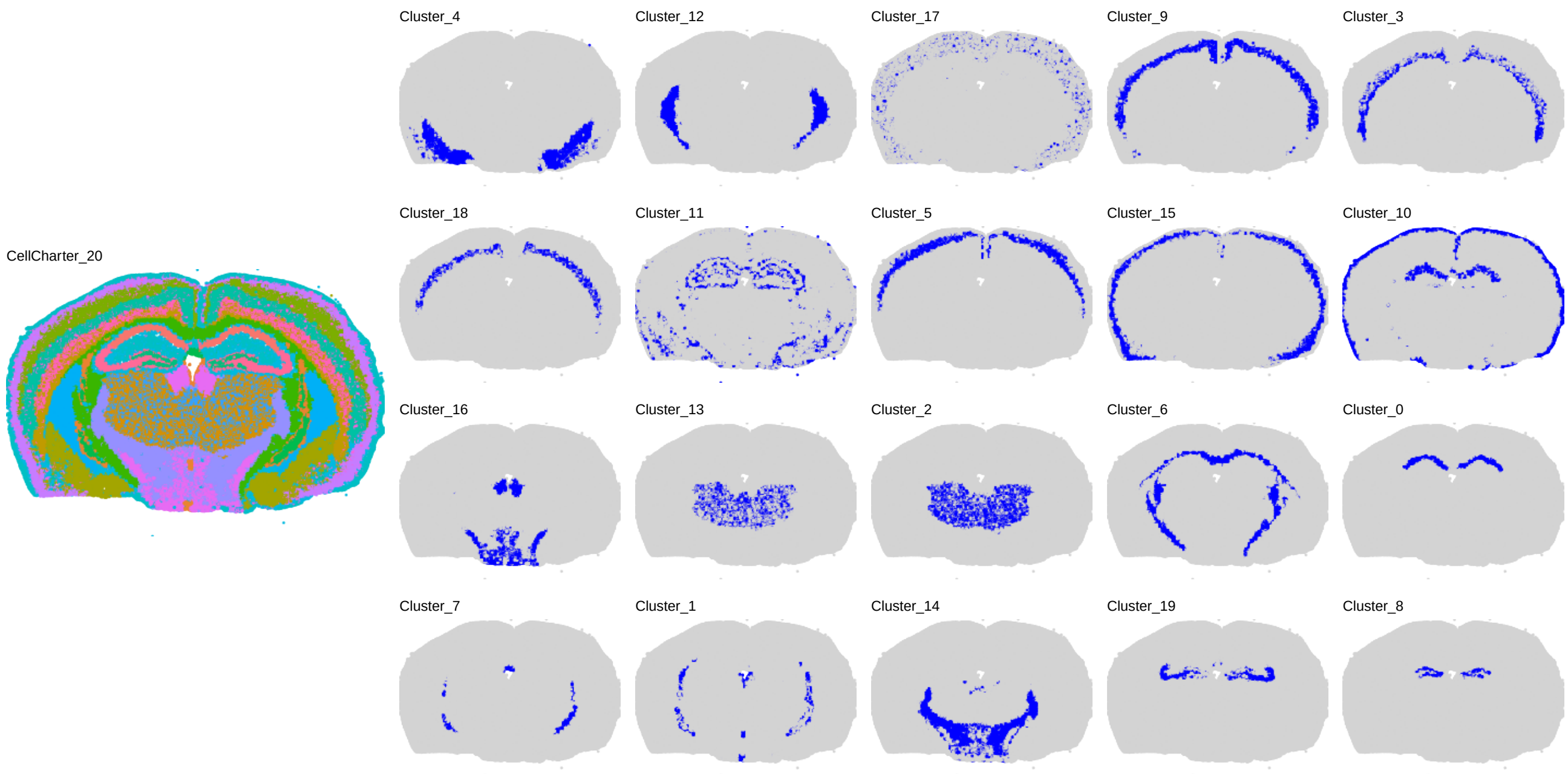
CellCharter_20
Cluster_4
Cluster_12
Cluster_17
Cluster_9
Cluster_3
Cluster_18
Cluster_11
Cluster_5
Cluster_15
Cluster_10
Cluster_16
Cluster_13
Cluster_2
Cluster_6
Cluster_0
Cluster_7
Cluster_1
Cluster_14
Cluster_19
Cluster_8

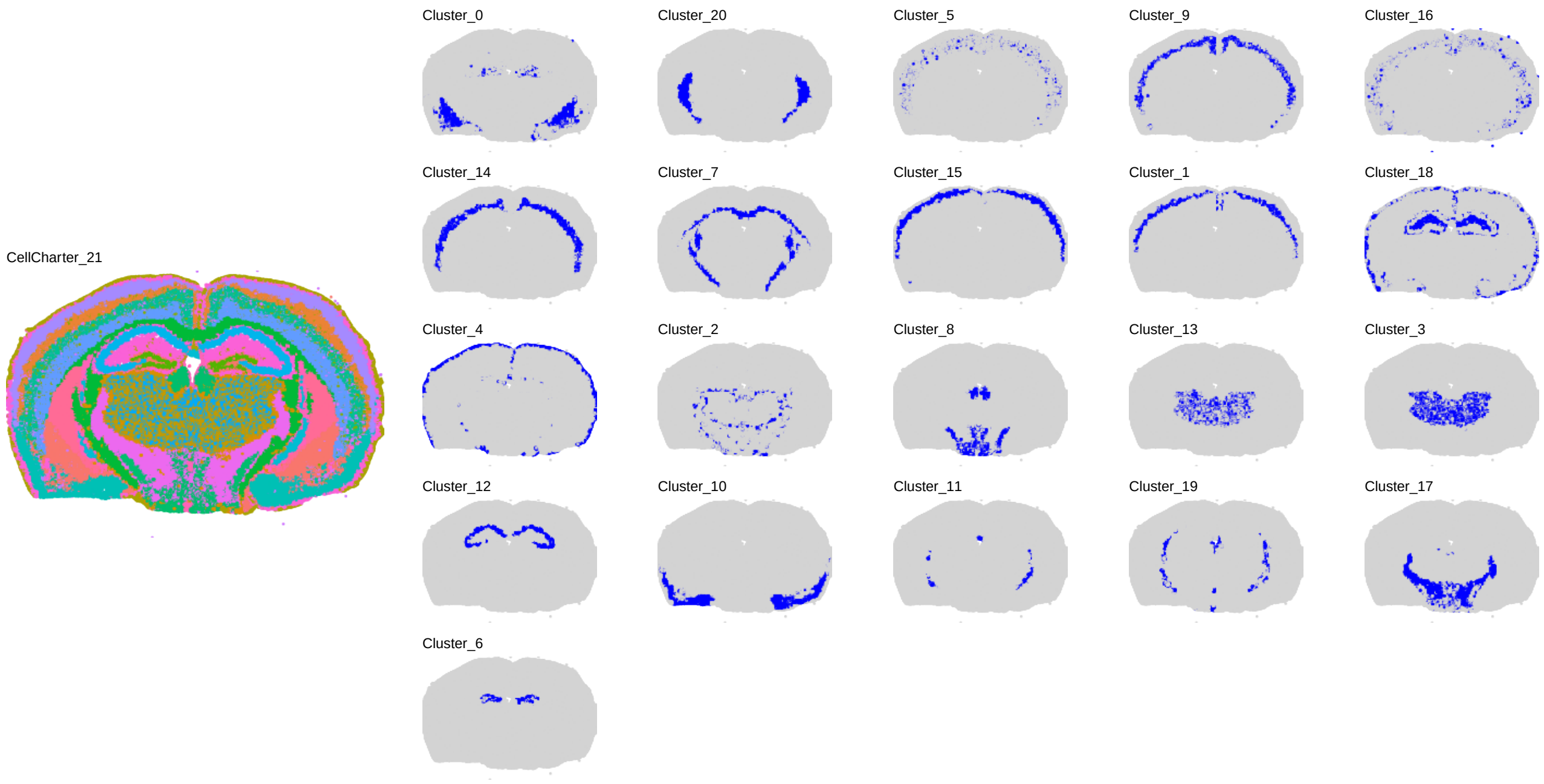
CellCharter_21
Cluster_0
Cluster_20
Cluster_5
Cluster_9
Cluster_16
Cluster_14
Cluster_7
Cluster_15
Cluster_1
Cluster_18
Cluster_4
Cluster_2
Cluster_8
Cluster_13
Cluster_3
Cluster_12
Cluster_10
Cluster_11
Cluster_19
Cluster_17
Cluster_6

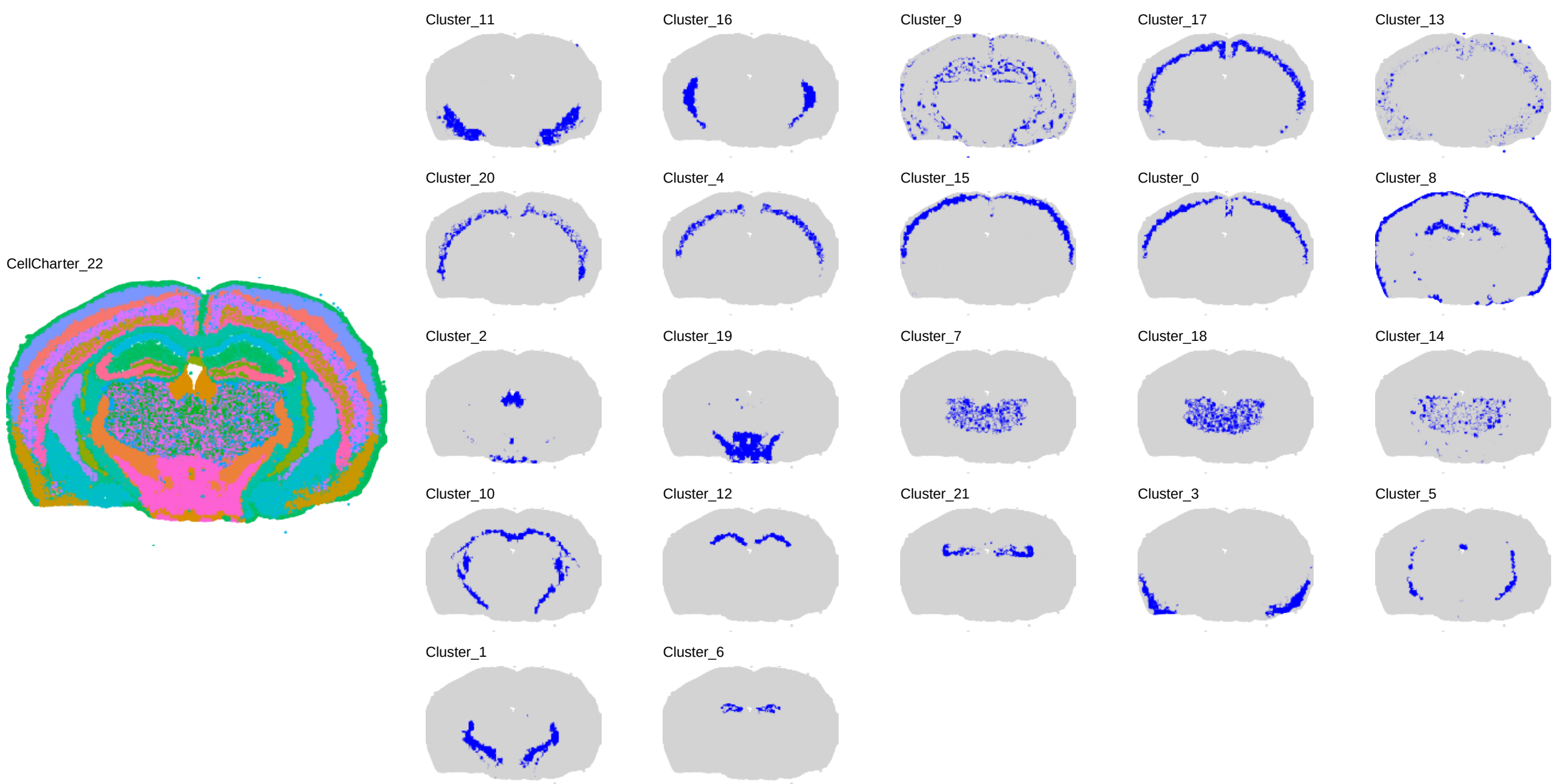
CellCharter_22
Cluster_11
Cluster_16
Cluster_9
Cluster_17
Cluster_13
Cluster_20
Cluster_4
Cluster_15
Cluster_0
Cluster_8
Cluster_2
Cluster_19
Cluster_7
Cluster_18
Cluster_14
Cluster_10
Cluster_12
Cluster_21
Cluster_3
Cluster_5
Cluster_1
Cluster_6

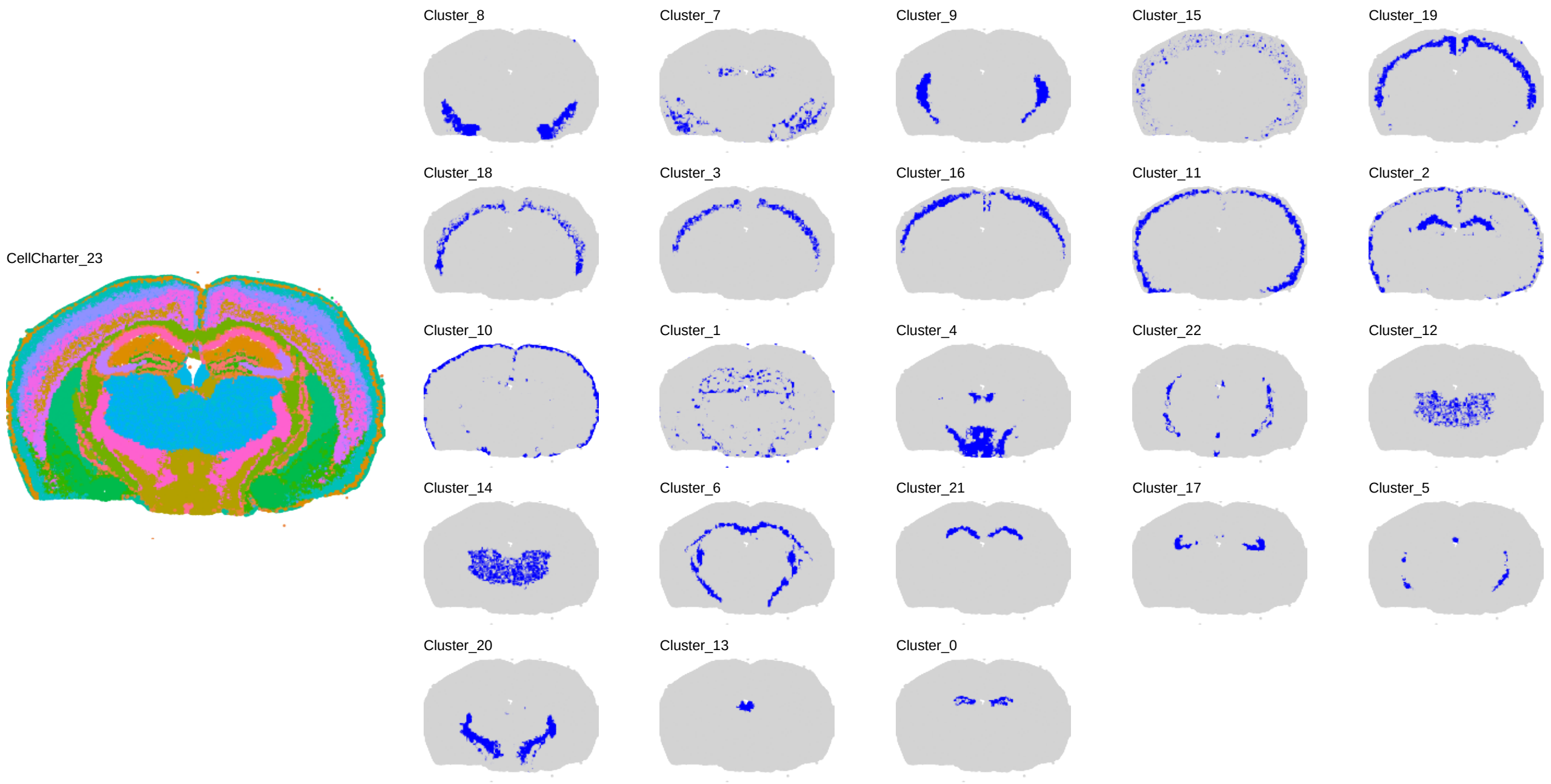
CellCharter_23
Cluster_8
Cluster_7
Cluster_9
Cluster_15
Cluster_19
Cluster_18
Cluster_3
Cluster_16
Cluster_11
Cluster_2
Cluster_10
Cluster_1
Cluster_4
Cluster_22
Cluster_12
Cluster_14
Cluster_6
Cluster_21
Cluster_17
Cluster_5
Cluster_20
Cluster_13
Cluster_0

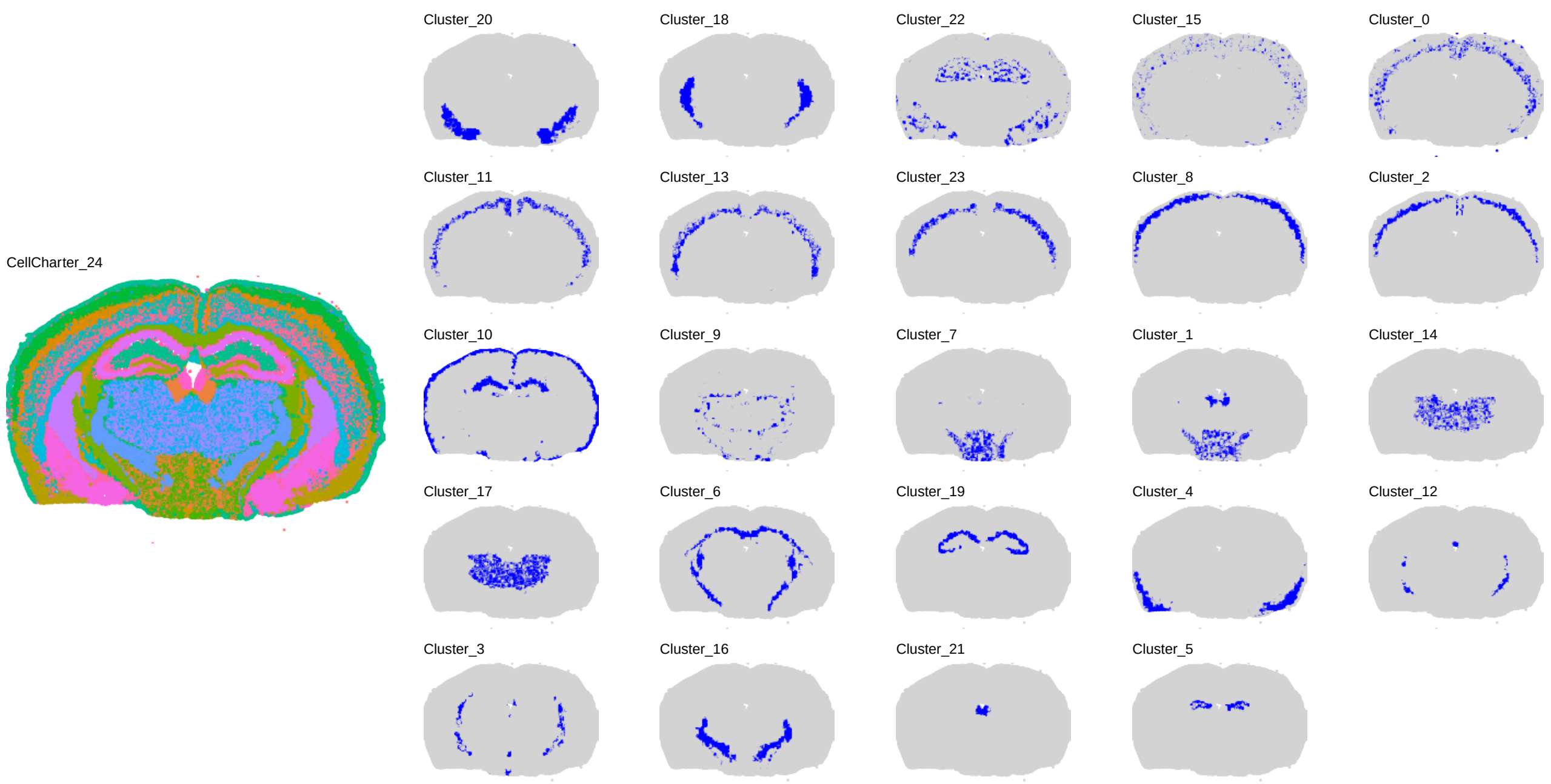
CellCharter_24
Cluster_20
Cluster_18
Cluster_22
Cluster_15
Cluster_0
Cluster_11
Cluster_13
Cluster_23
Cluster_8
Cluster_2
Cluster_10
Cluster_9
Cluster_7
Cluster_1
Cluster_14
Cluster_17
Cluster_6
Cluster_19
Cluster_4
Cluster_12
Cluster_3
Cluster_16
Cluster_21
Cluster_5

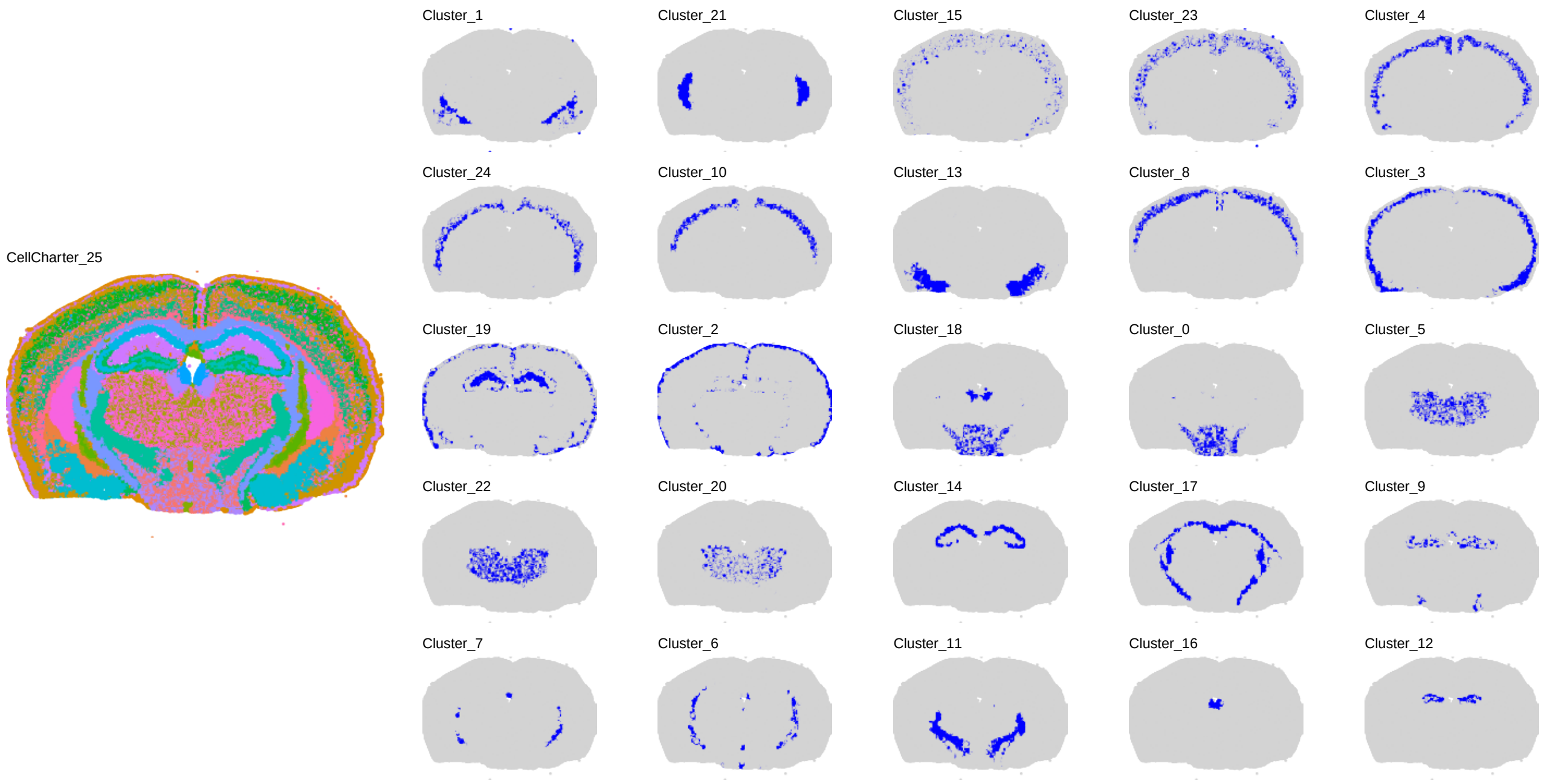
CellCharter_25
Cluster_1
Cluster_21
Cluster_15
Cluster_23
Cluster_4
Cluster_24
Cluster_10
Cluster_13
Cluster_8
Cluster_3
Cluster_19
Cluster_2
Cluster_18
Cluster_0
Cluster_5
Cluster_22
Cluster_20
Cluster_14
Cluster_17
Cluster_9
Cluster_7
Cluster_6
Cluster_11
Cluster_16
Cluster_12

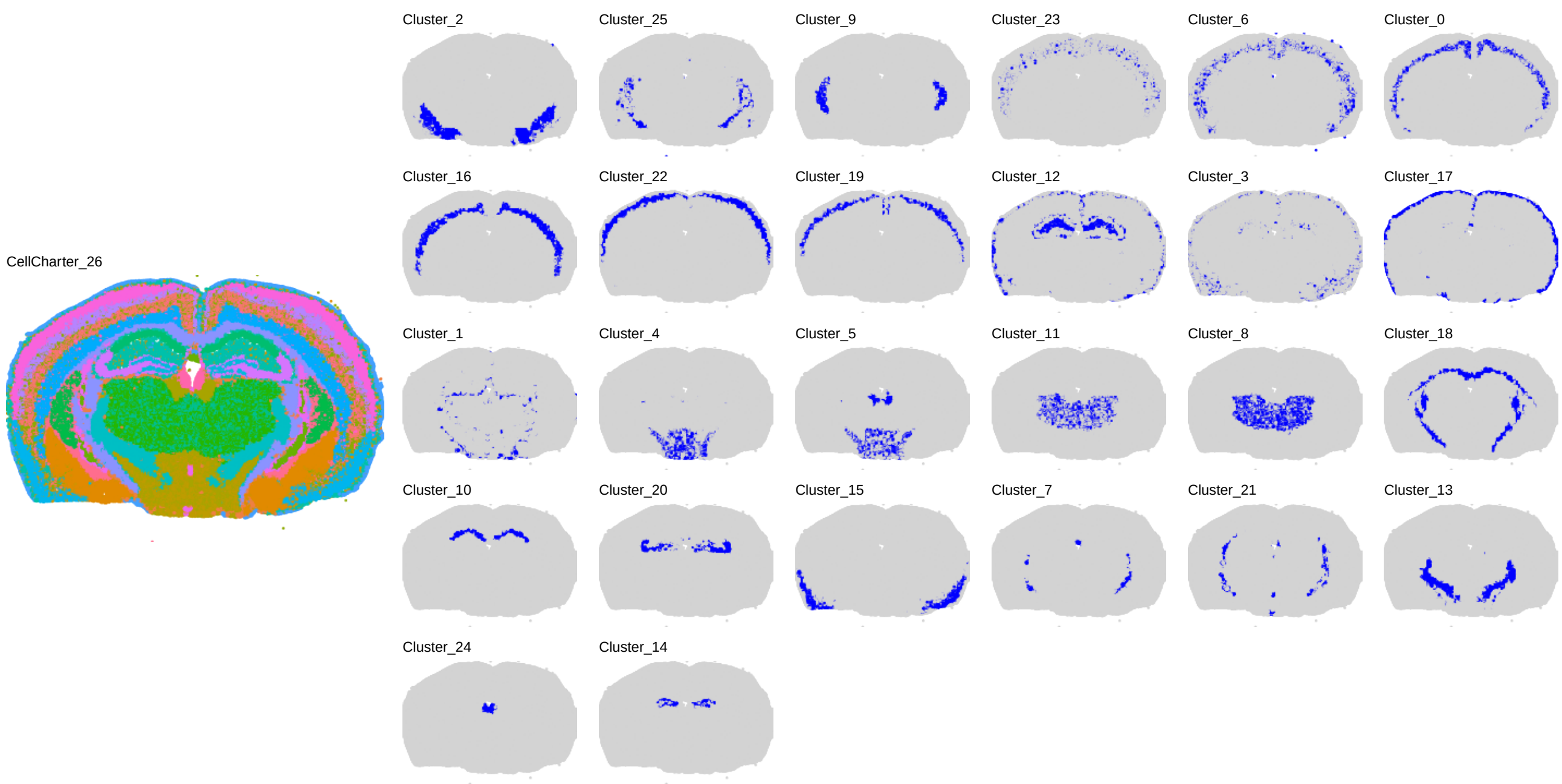
CellCharter_26
Cluster_2
Cluster_25
Cluster_9
Cluster_23
Cluster_6
Cluster_0
Cluster_16
Cluster_22
Cluster_19
Cluster_12
Cluster_3
Cluster_17
Cluster_1
Cluster_4
Cluster_5
Cluster_11
Cluster_8
Cluster_18
Cluster_10
Cluster_20
Cluster_15
Cluster_7
Cluster_21
Cluster_13
Cluster_24
Cluster_14

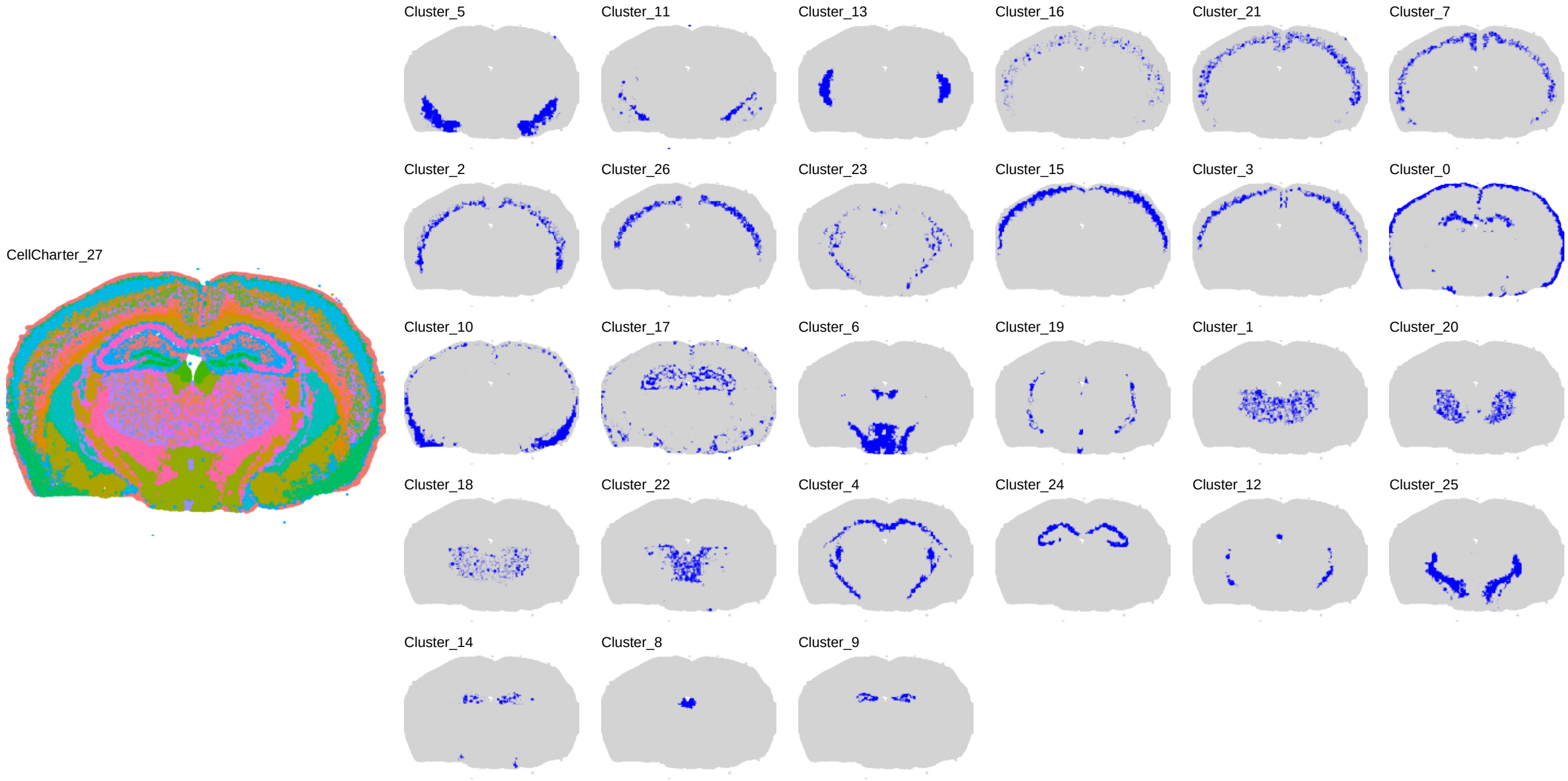
CellCharter_27
Cluster_5
Cluster_11
Cluster_13
Cluster_16
Cluster_21
Cluster_7
Cluster_2
Cluster_26
Cluster_23
Cluster_15
Cluster_3
Cluster_0
Cluster_10
Cluster_17
Cluster_6
Cluster_19
Cluster_1
Cluster_20
Cluster_18
Cluster_22
Cluster_4
Cluster_24
Cluster_12
Cluster_25
Cluster_14
Cluster_8
Cluster_9

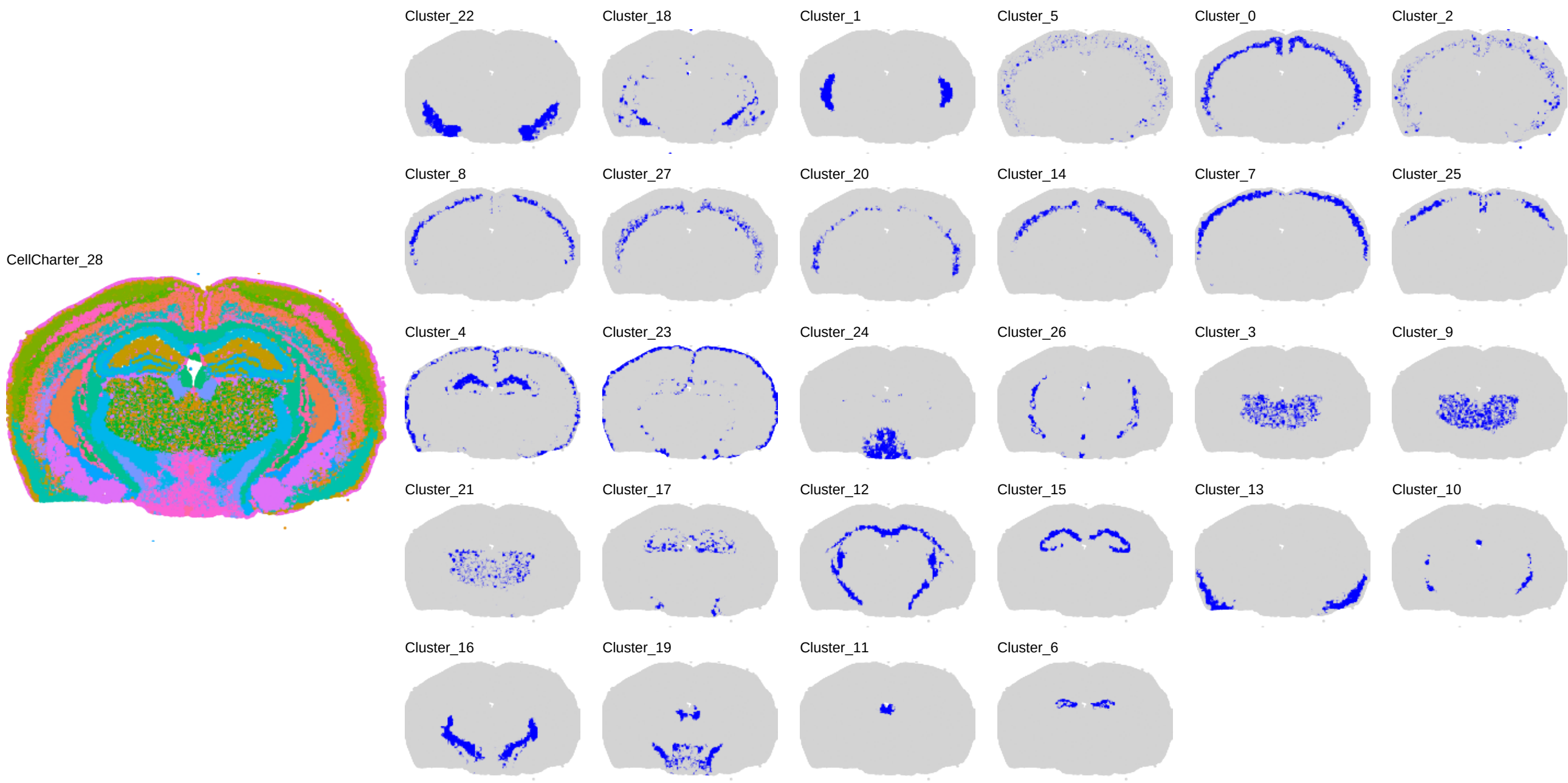
CellCharter_28
Cluster_22
Cluster_18
Cluster_1
Cluster_5
Cluster_0
Cluster_2
Cluster_8
Cluster_27
Cluster_20
Cluster_14
Cluster_7
Cluster_25
Cluster_4
Cluster_23
Cluster_24
Cluster_26
Cluster_3
Cluster_9
Cluster_21
Cluster_17
Cluster_12
Cluster_15
Cluster_13
Cluster_10
Cluster_16
Cluster_19
Cluster_11
Cluster_6

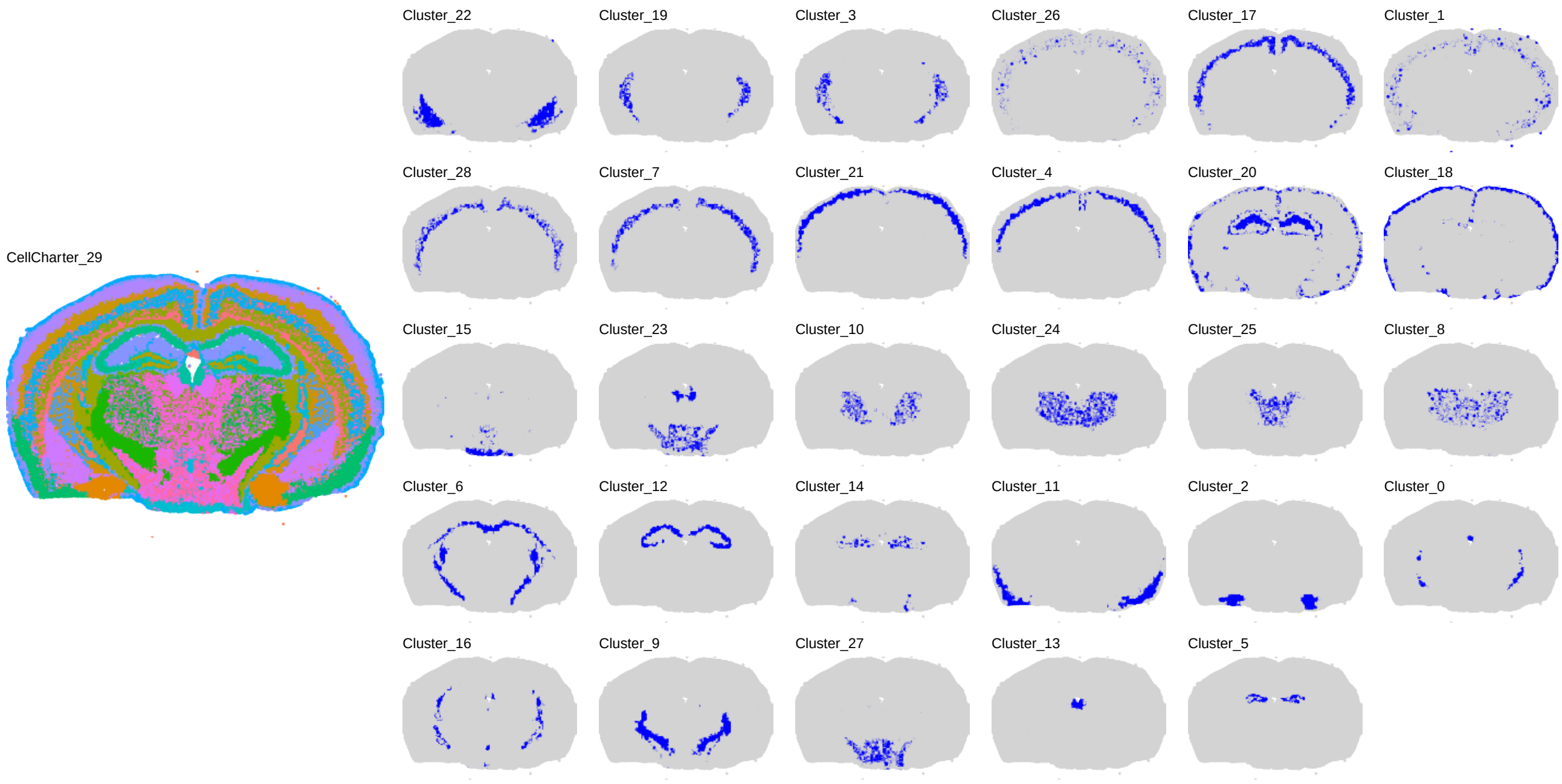
CellCharter_29
Cluster_22
Cluster_19
Cluster_3
Cluster_26
Cluster_17
Cluster_1
Cluster_28
Cluster_7
Cluster_21
Cluster_4
Cluster_20
Cluster_18
Cluster_15
Cluster_23
Cluster_10
Cluster_24
Cluster_25
Cluster_8
Cluster_6
Cluster_12
Cluster_14
Cluster_11
Cluster_2
Cluster_0
Cluster_16
Cluster_9
Cluster_27
Cluster_13
Cluster_5

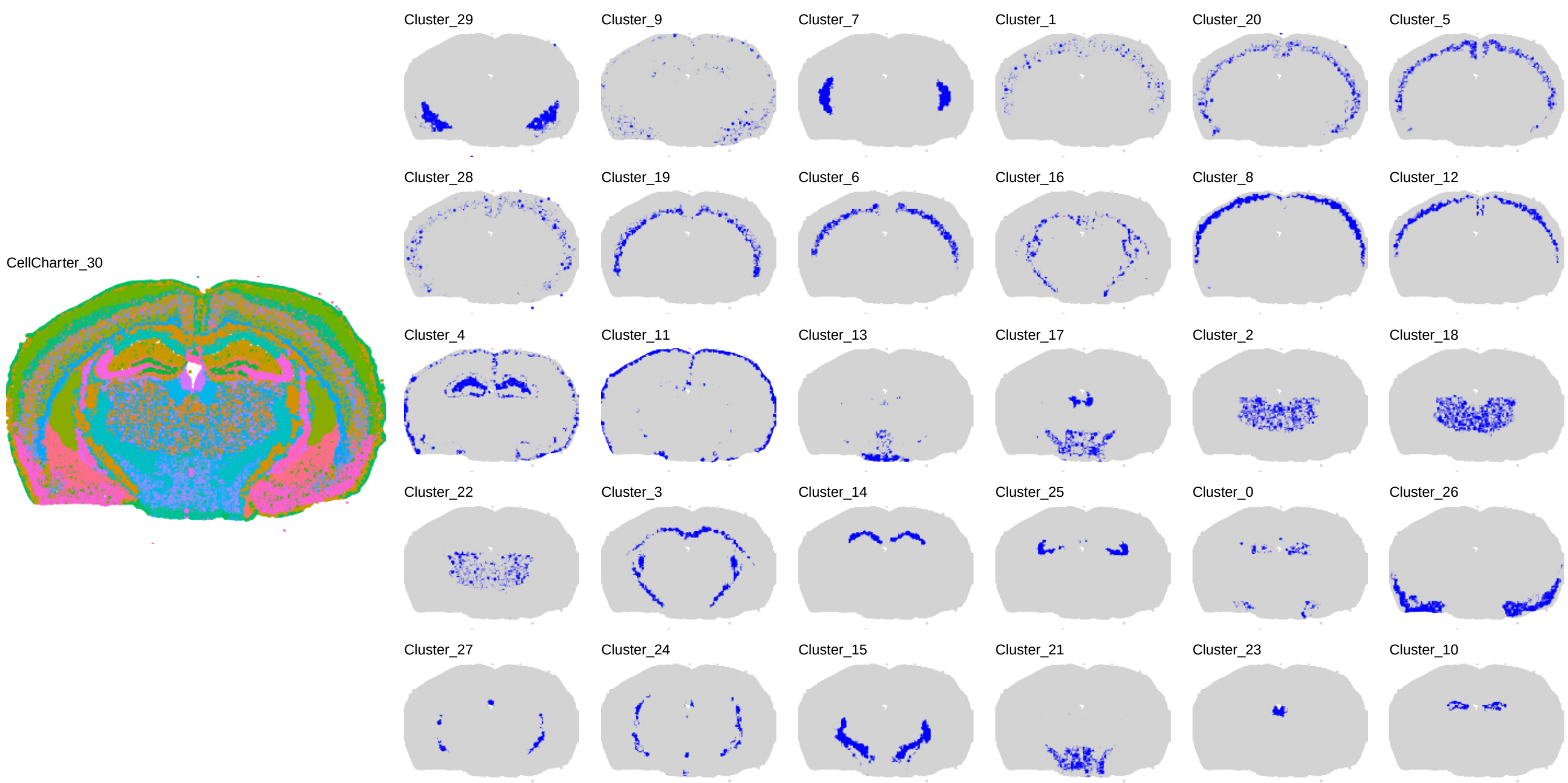
CellCharter_30
Cluster_29
Cluster_9
Cluster_7
Cluster_1
Cluster_20
Cluster_5
Cluster_28
Cluster_19
Cluster_6
Cluster_16
Cluster_8
Cluster_12
Cluster_4
Cluster_11
Cluster_13
Cluster_17
Cluster_2
Cluster_18
Cluster_22
Cluster_3
Cluster_14
Cluster_25
Cluster_0
Cluster_26
Cluster_27
Cluster_24
Cluster_15
Cluster_21
Cluster_23
Cluster_10

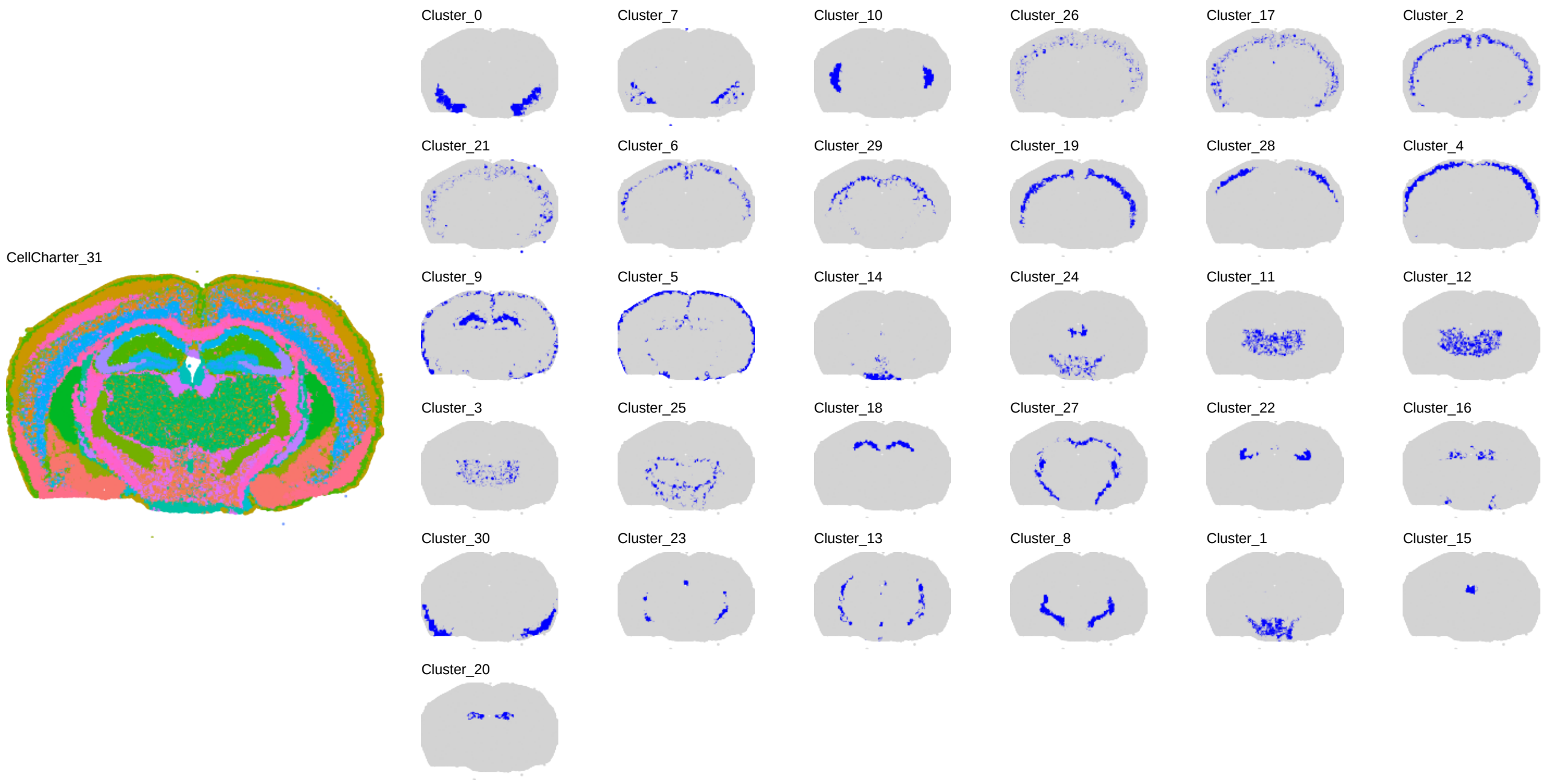
CellCharter_31
Cluster_0
Cluster_7
Cluster_10
Cluster_26
Cluster_17
Cluster_2
Cluster_21
Cluster_6
Cluster_29
Cluster_19
Cluster_28
Cluster_4
Cluster_9
Cluster_5
Cluster_14
Cluster_24
Cluster_11
Cluster_12
Cluster_3
Cluster_25
Cluster_18
Cluster_27
Cluster_22
Cluster_16
Cluster_30
Cluster_23
Cluster_13
Cluster_8
Cluster_1
Cluster_15
Cluster_20

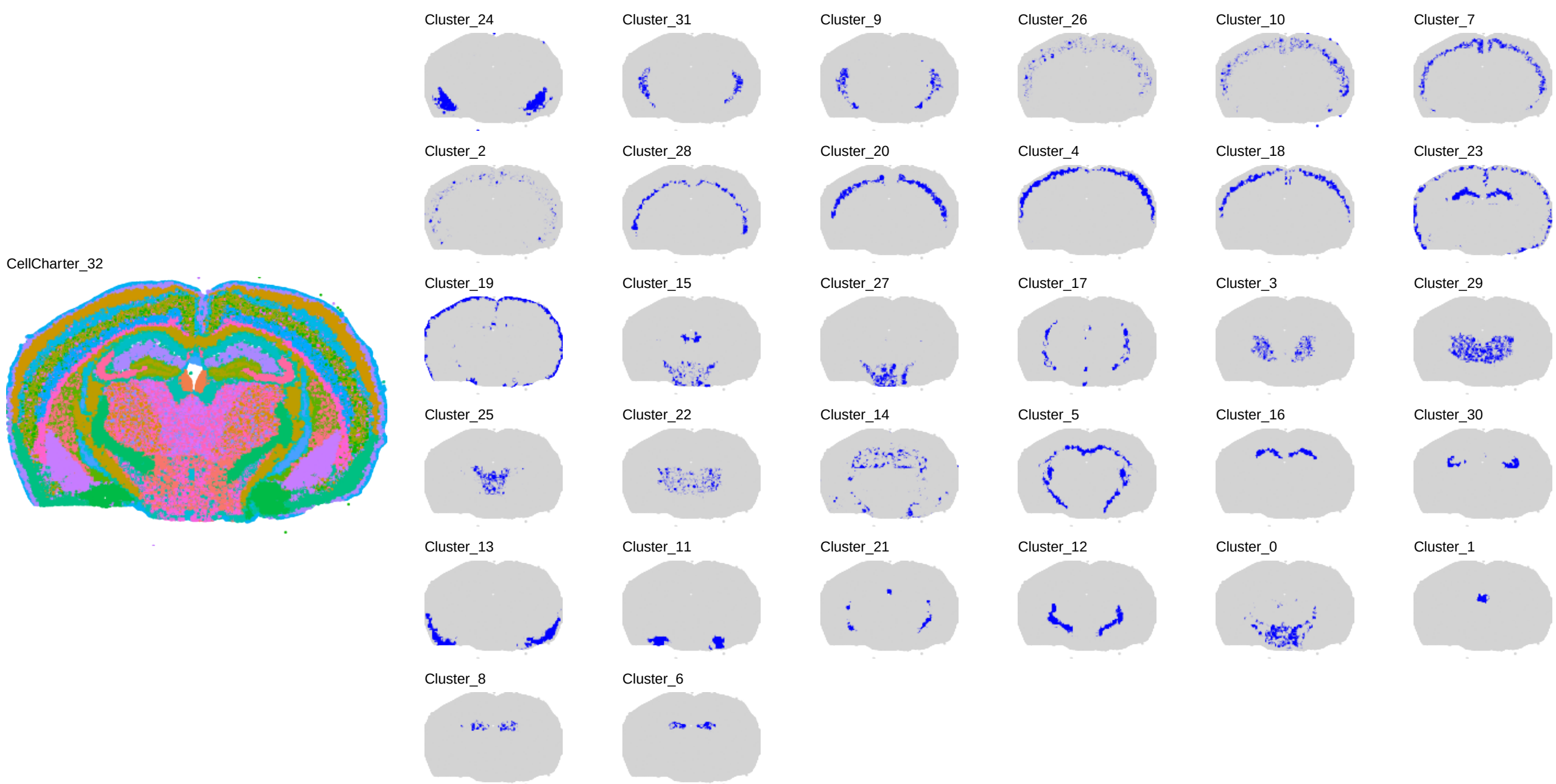
CellCharter_32
Cluster_24
Cluster_31
Cluster_9
Cluster_26
Cluster_10
Cluster_7
Cluster_2
Cluster_28
Cluster_20
Cluster_4
Cluster_18
Cluster_23
Cluster_19
Cluster_15
Cluster_27
Cluster_17
Cluster_3
Cluster_29
Cluster_25
Cluster_22
Cluster_14
Cluster_5
Cluster_16
Cluster_30
Cluster_13
Cluster_11
Cluster_21
Cluster_12
Cluster_0
Cluster_1
Cluster_8
Cluster_6

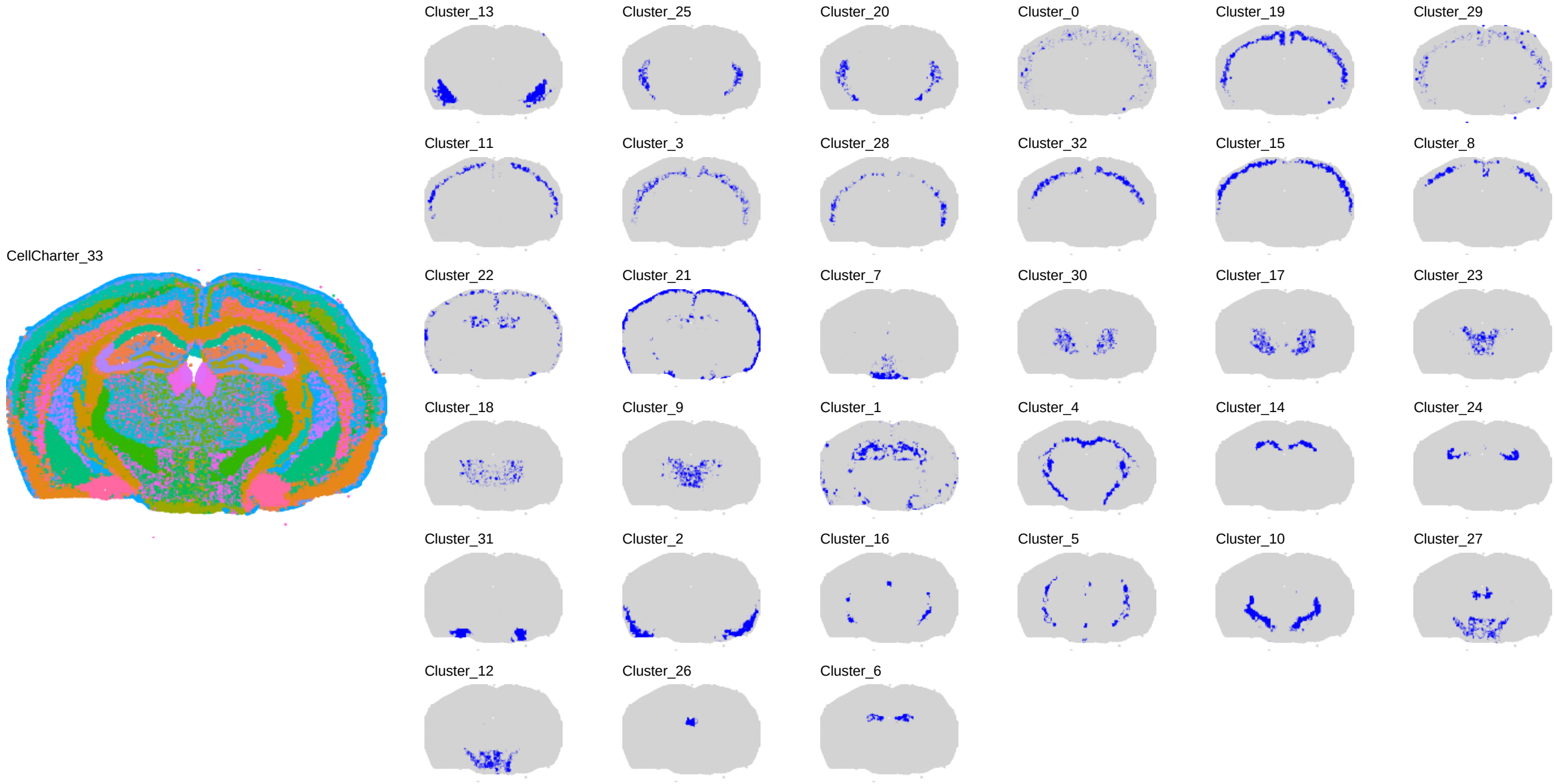
CellCharter_33
Cluster_13
Cluster_25
Cluster_20
Cluster_0
Cluster_19
Cluster_29
Cluster_11
Cluster_3
Cluster_28
Cluster_32
Cluster_15
Cluster_8
Cluster_22
Cluster_21
Cluster_7
Cluster_30
Cluster_17
Cluster_23
Cluster_18
Cluster_9
Cluster_1
Cluster_4
Cluster_14
Cluster_24
Cluster_31
Cluster_2
Cluster_16
Cluster_5
Cluster_10
Cluster_27
Cluster_12
Cluster_26
Cluster_6

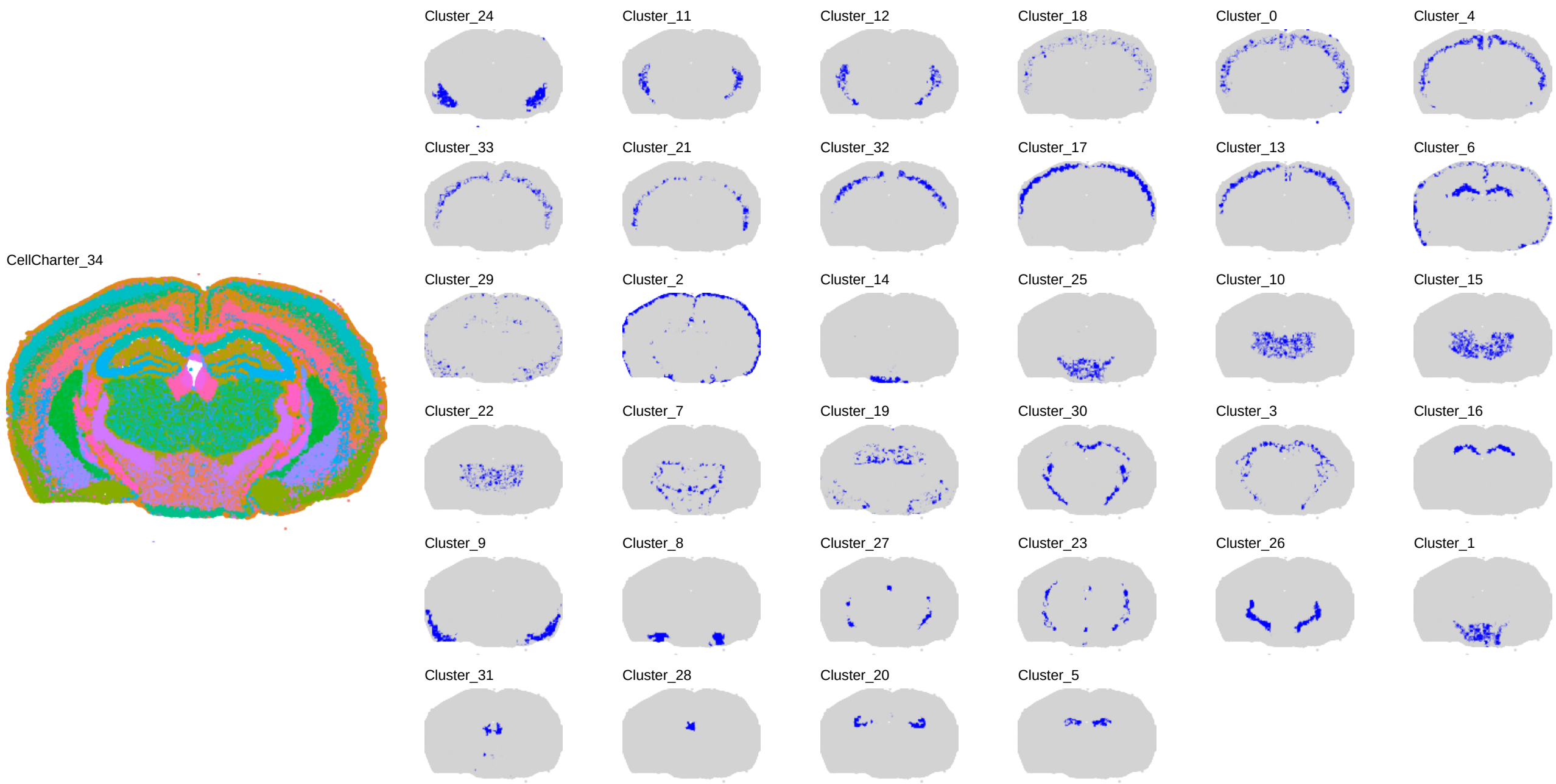
CellCharter_34
Cluster_24
Cluster_11
Cluster_12
Cluster_18
Cluster_0
Cluster_4
Cluster_33
Cluster_21
Cluster_32
Cluster_17
Cluster_13
Cluster_6
Cluster_29
Cluster_2
Cluster_14
Cluster_25
Cluster_10
Cluster_15
Cluster_22
Cluster_7
Cluster_19
Cluster_30
Cluster_3
Cluster_16
Cluster_9
Cluster_8
Cluster_27
Cluster_23
Cluster_26
Cluster_1
Cluster_31
Cluster_28
Cluster_20
Cluster_5

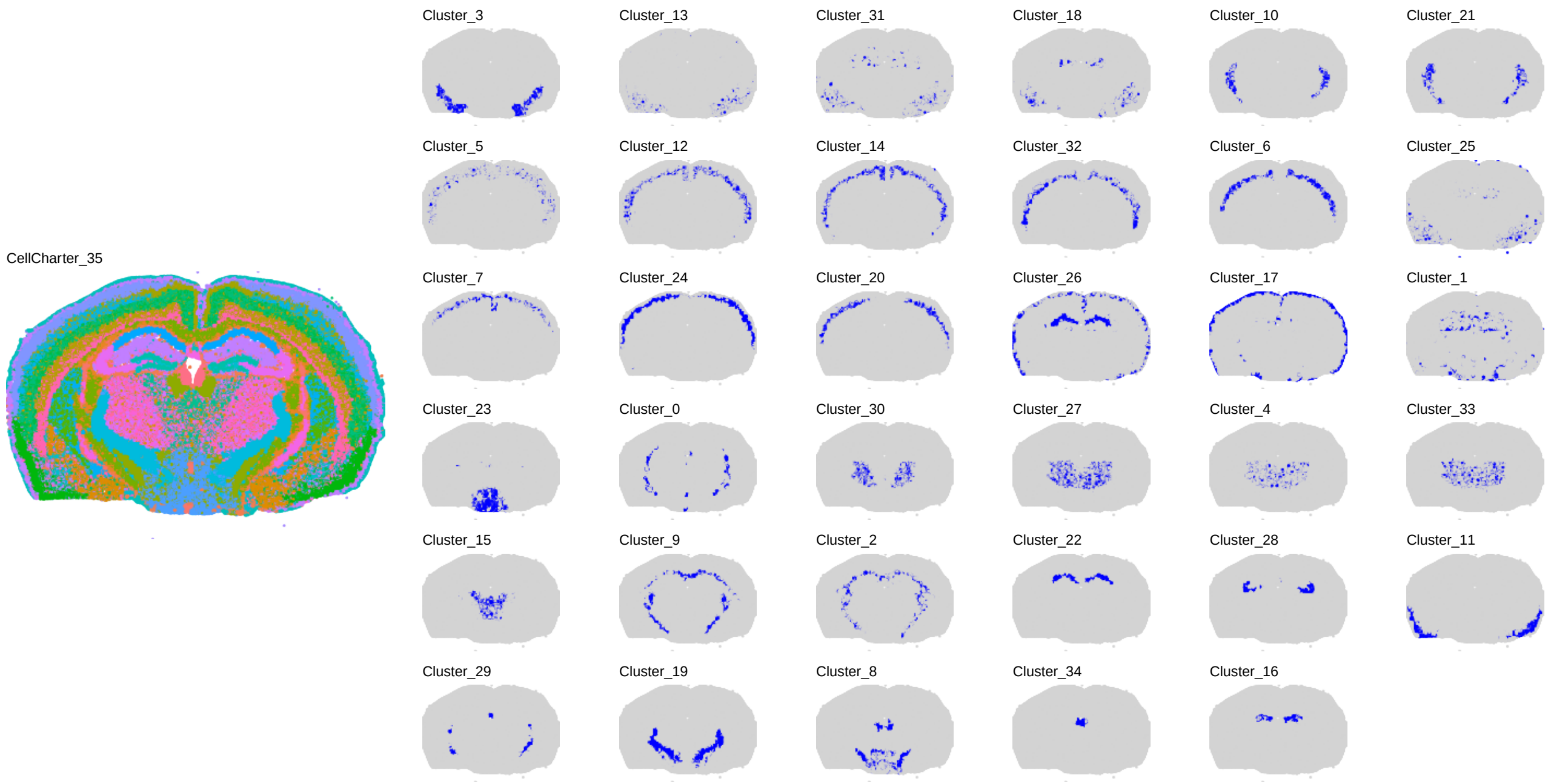
CellCharter_35
Cluster_3
Cluster_13
Cluster_31
Cluster_18
Cluster_10
Cluster_21
Cluster_5
Cluster_12
Cluster_14
Cluster_32
Cluster_6
Cluster_25
Cluster_7
Cluster_24
Cluster_20
Cluster_26
Cluster_17
Cluster_1
Cluster_23
Cluster_0
Cluster_30
Cluster_27
Cluster_4
Cluster_33
Cluster_15
Cluster_9
Cluster_2
Cluster_22
Cluster_28
Cluster_11
Cluster_29
Cluster_19
Cluster_8
Cluster_34
Cluster_16

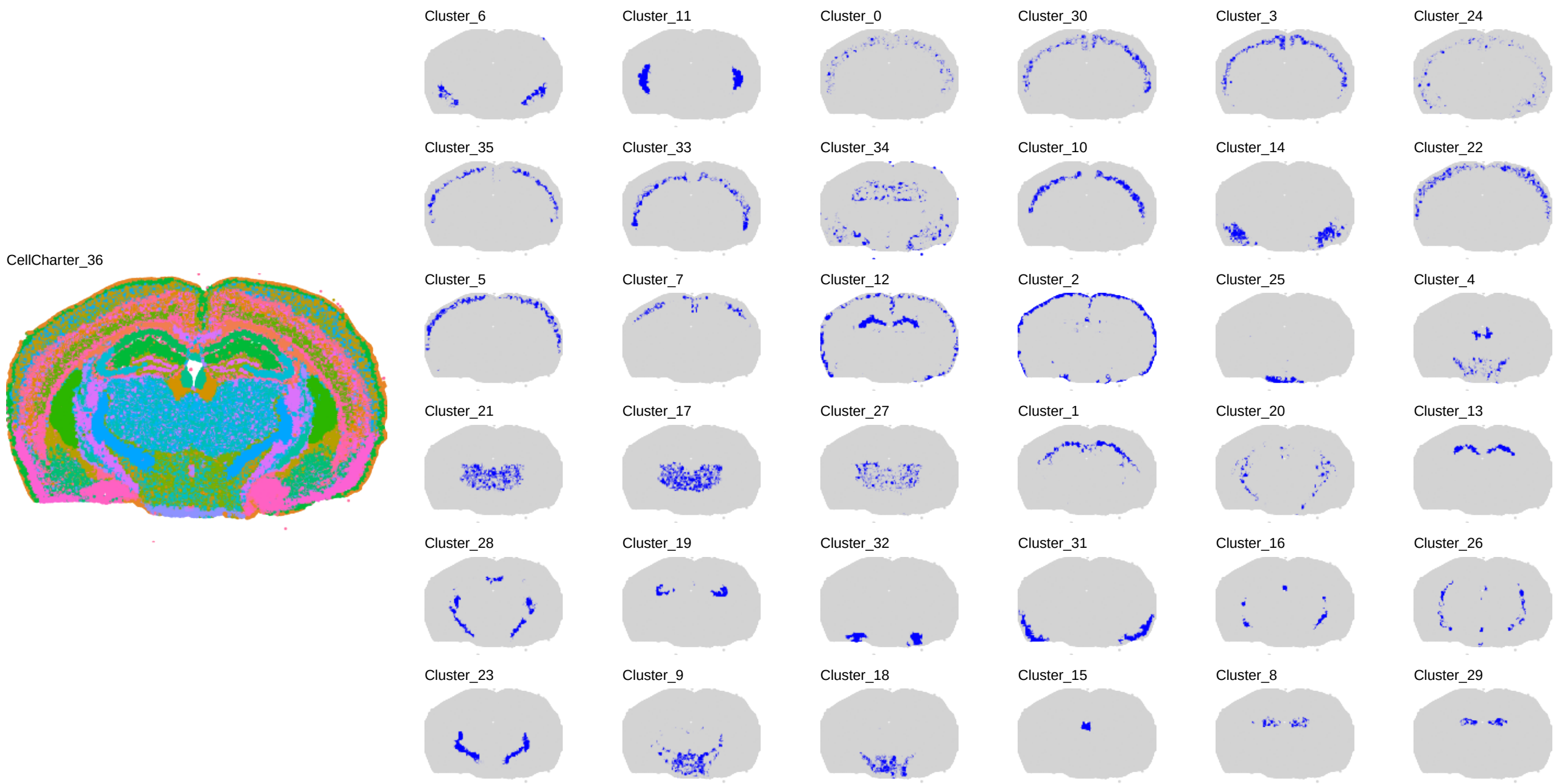
CellCharter_36
Cluster_6
Cluster_11
Cluster_0
Cluster_30
Cluster_3
Cluster_24
Cluster_35
Cluster_33
Cluster_34
Cluster_10
Cluster_14
Cluster_22
Cluster_5
Cluster_7
Cluster_12
Cluster_2
Cluster_25
Cluster_4
Cluster_21
Cluster_17
Cluster_27
Cluster_1
Cluster_20
Cluster_13
Cluster_28
Cluster_19
Cluster_32
Cluster_31
Cluster_16
Cluster_26
Cluster_23
Cluster_9
Cluster_18
Cluster_15
Cluster_8
Cluster_29

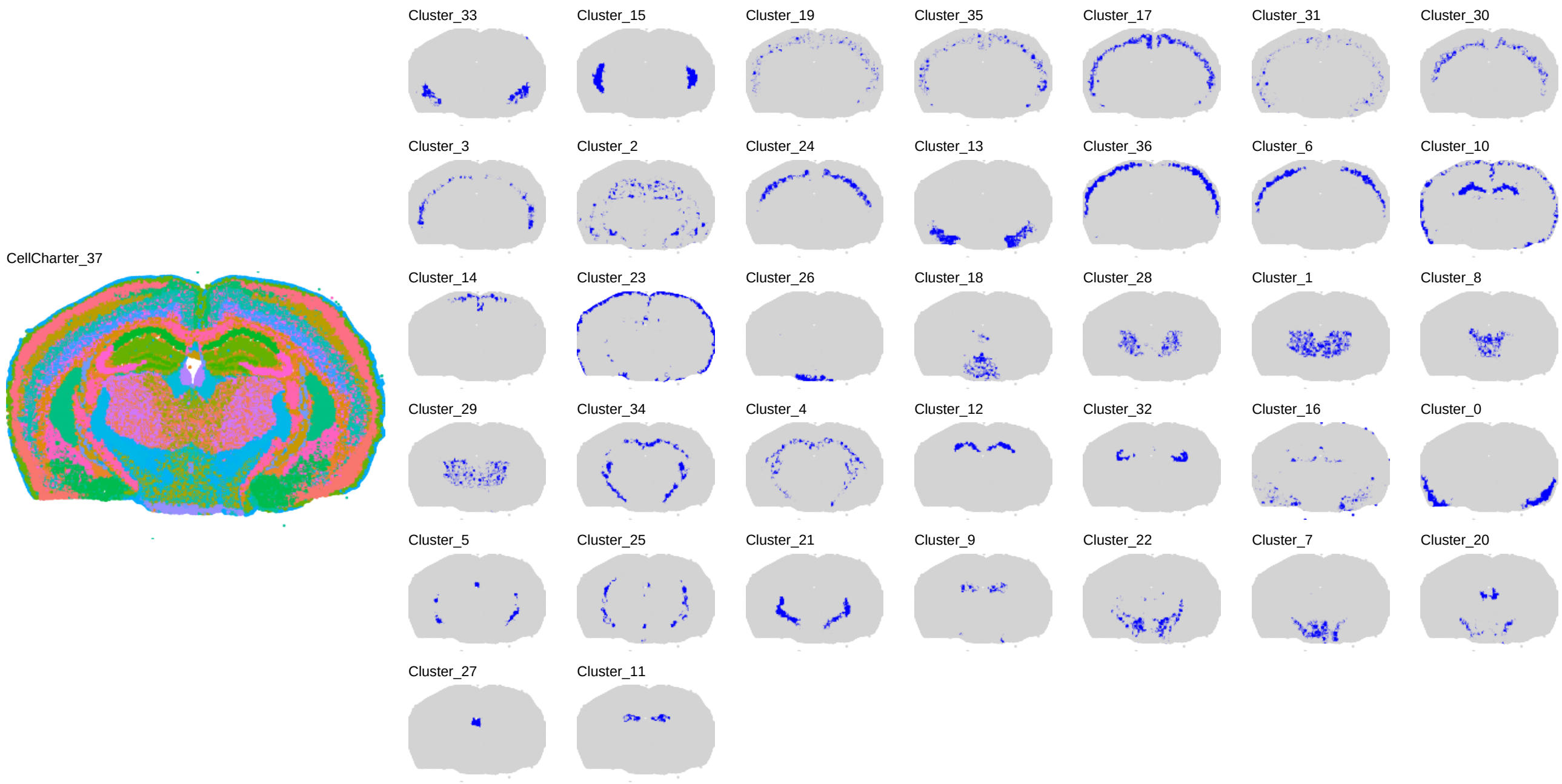
CellCharter_37
Cluster_33
Cluster_15
Cluster_19
Cluster_35
Cluster_17
Cluster_31
Cluster_30
Cluster_3
Cluster_2
Cluster_24
Cluster_13
Cluster_36
Cluster_6
Cluster_10
Cluster_14
Cluster_23
Cluster_26
Cluster_18
Cluster_28
Cluster_1
Cluster_8
Cluster_29
Cluster_34
Cluster_4
Cluster_12
Cluster_32
Cluster_16
Cluster_0
Cluster_5
Cluster_25
Cluster_21
Cluster_9
Cluster_22
Cluster_7
Cluster_20
Cluster_27
Cluster_11

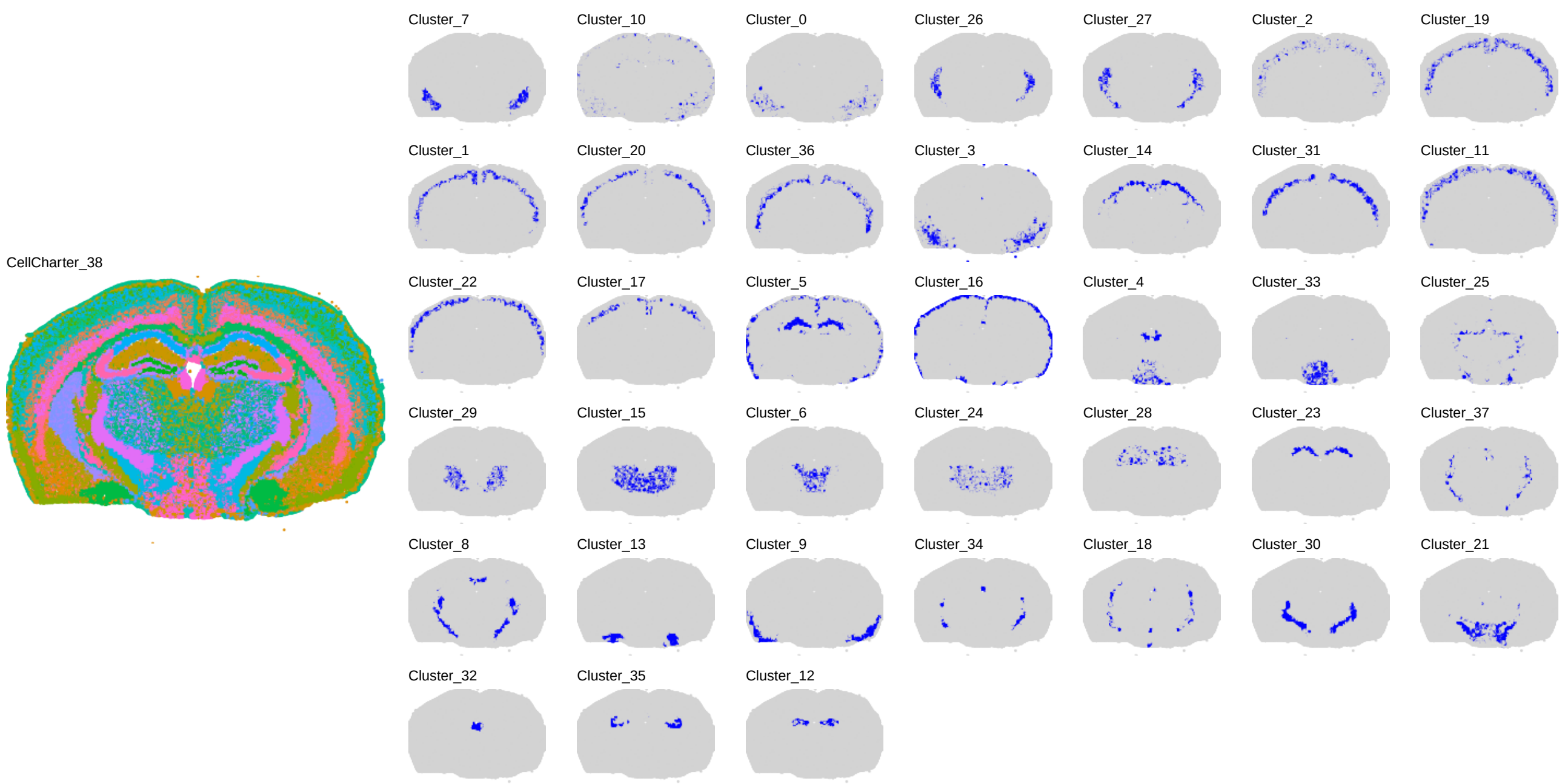
CellCharter_38
Cluster_7
Cluster_10
Cluster_0
Cluster_26
Cluster_27
Cluster_2
Cluster_19
Cluster_1
Cluster_20
Cluster_36
Cluster_3
Cluster_14
Cluster_31
Cluster_11
Cluster_22
Cluster_17
Cluster_5
Cluster_16
Cluster_4
Cluster_33
Cluster_25
Cluster_29
Cluster_15
Cluster_6
Cluster_24
Cluster_28
Cluster_23
Cluster_37
Cluster_8
Cluster_13
Cluster_9
Cluster_34
Cluster_18
Cluster_30
Cluster_21
Cluster_32
Cluster_35
Cluster_12

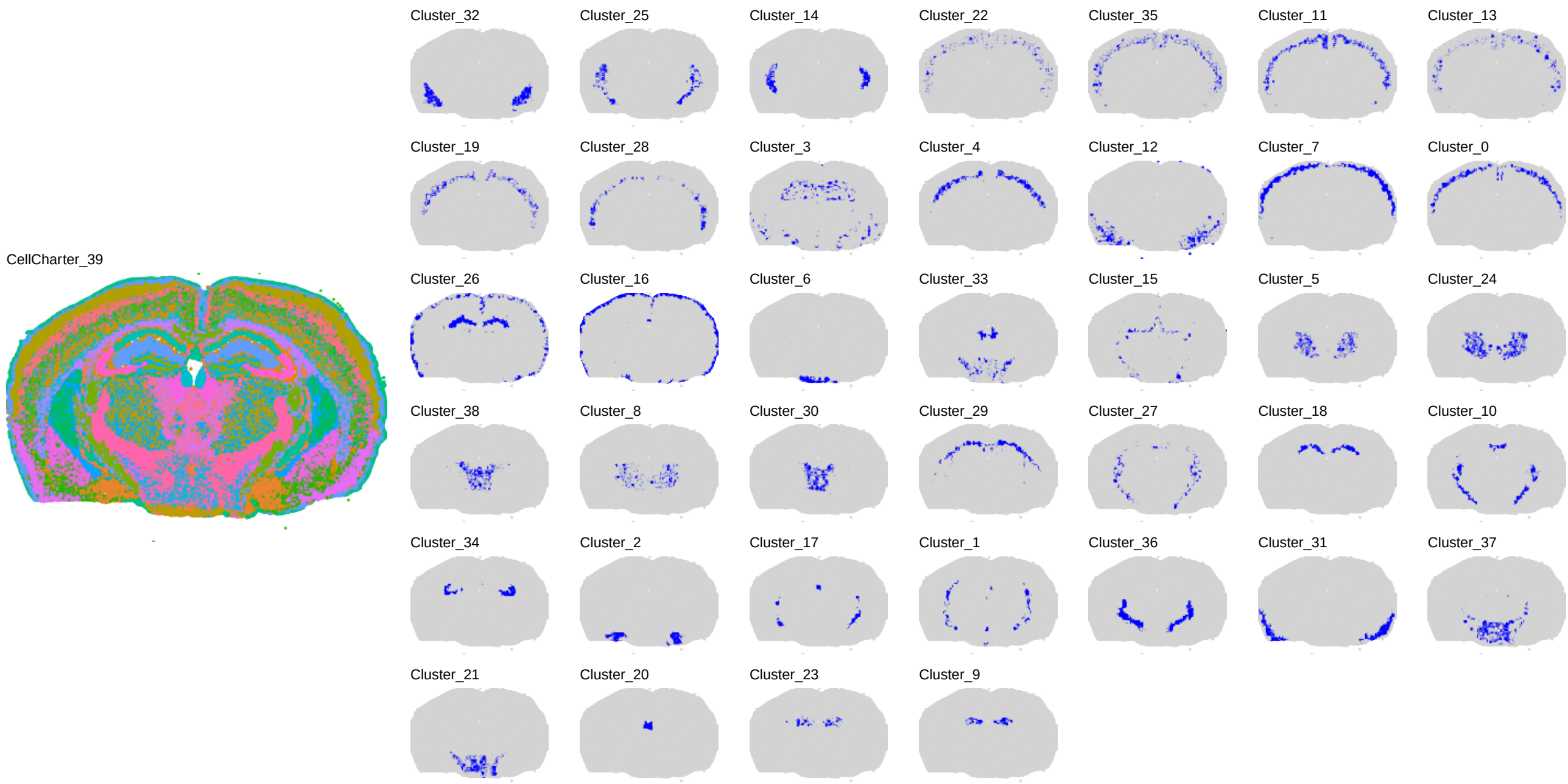
CellCharter_39
Cluster_32
Cluster_25
Cluster_14
Cluster_22
Cluster_35
Cluster_11
Cluster_13
Cluster_19
Cluster_28
Cluster_3
Cluster_4
Cluster_12
Cluster_7
Cluster_0
Cluster_26
Cluster_16
Cluster_6
Cluster_33
Cluster_15
Cluster_5
Cluster_24
Cluster_38
Cluster_8
Cluster_30
Cluster_29
Cluster_27
Cluster_18
Cluster_10
Cluster_34
Cluster_2
Cluster_17
Cluster_1
Cluster_36
Cluster_31
Cluster_37
Cluster_21
Cluster_20
Cluster_23
Cluster_9

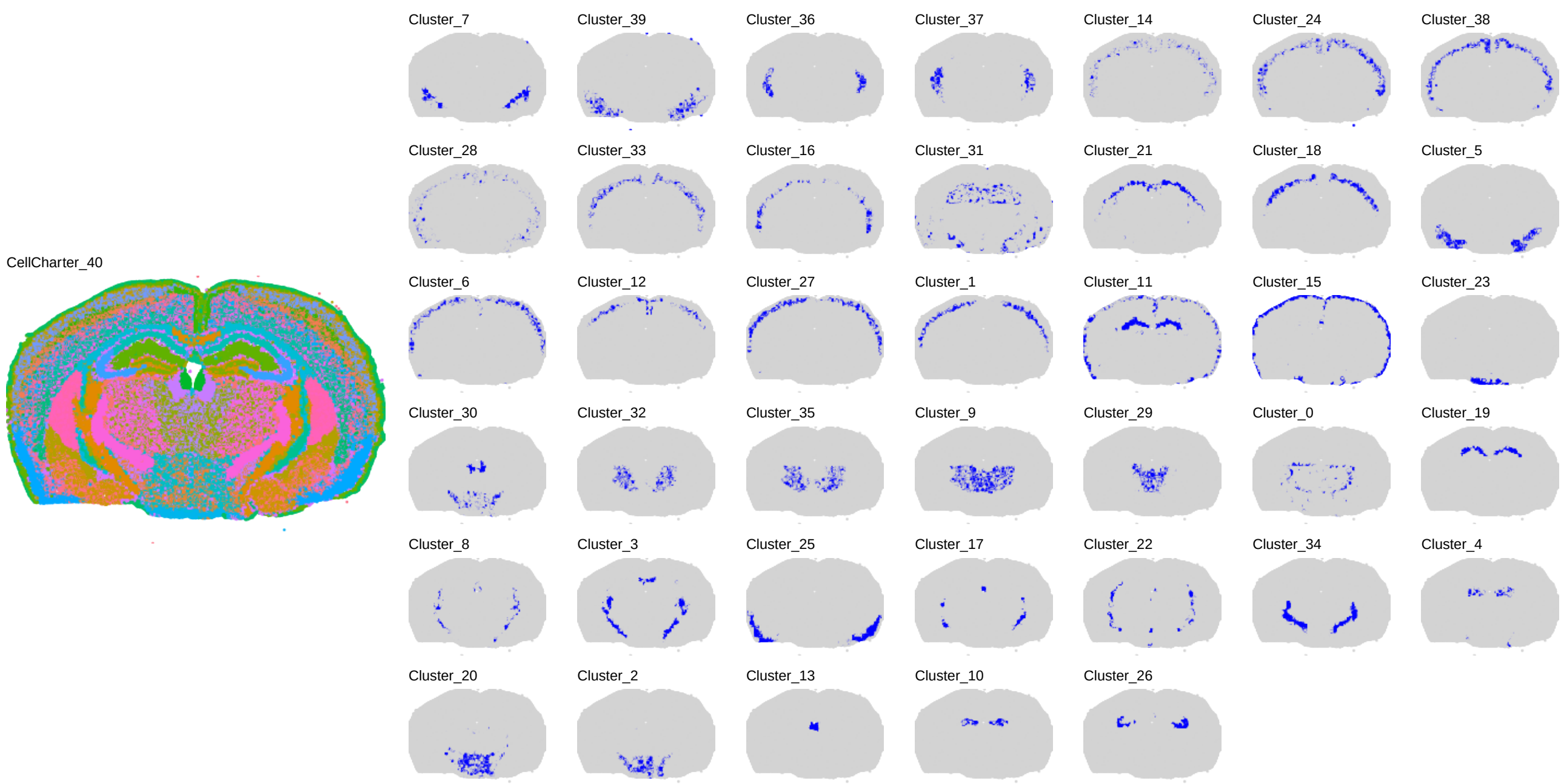
CellCharter_40
Cluster_7
Cluster_39
Cluster_36
Cluster_37
Cluster_14
Cluster_24
Cluster_38
Cluster_28
Cluster_33
Cluster_16
Cluster_31
Cluster_21
Cluster_18
Cluster_5
Cluster_6
Cluster_12
Cluster_27
Cluster_1
Cluster_11
Cluster_15
Cluster_23
Cluster_30
Cluster_32
Cluster_35
Cluster_9
Cluster_29
Cluster_0
Cluster_19
Cluster_8
Cluster_3
Cluster_25
Cluster_17
Cluster_22
Cluster_34
Cluster_4
Cluster_20
Cluster_2
Cluster_13
Cluster_10
Cluster_26

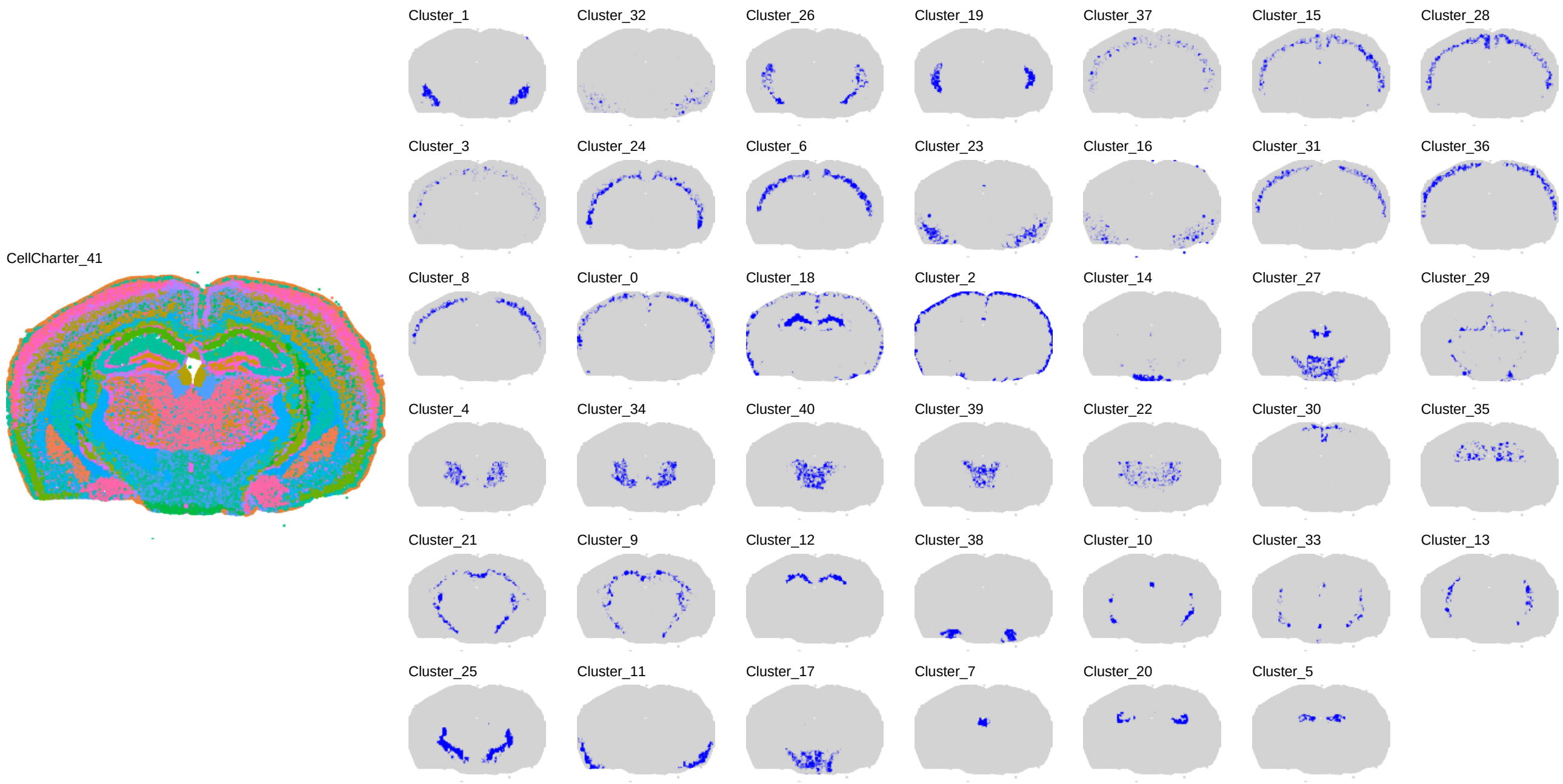
CellCharter_41
Cluster_1
Cluster_32
Cluster_26
Cluster_19
Cluster_37
Cluster_15
Cluster_28
Cluster_3
Cluster_24
Cluster_6
Cluster_23
Cluster_16
Cluster_31
Cluster_36
Cluster_8
Cluster_0
Cluster_18
Cluster_2
Cluster_14
Cluster_27
Cluster_29
Cluster_4
Cluster_34
Cluster_40
Cluster_39
Cluster_22
Cluster_30
Cluster_35
Cluster_21
Cluster_9
Cluster_12
Cluster_38
Cluster_10
Cluster_33
Cluster_13
Cluster_25
Cluster_11
Cluster_17
Cluster_7
Cluster_20
Cluster_5

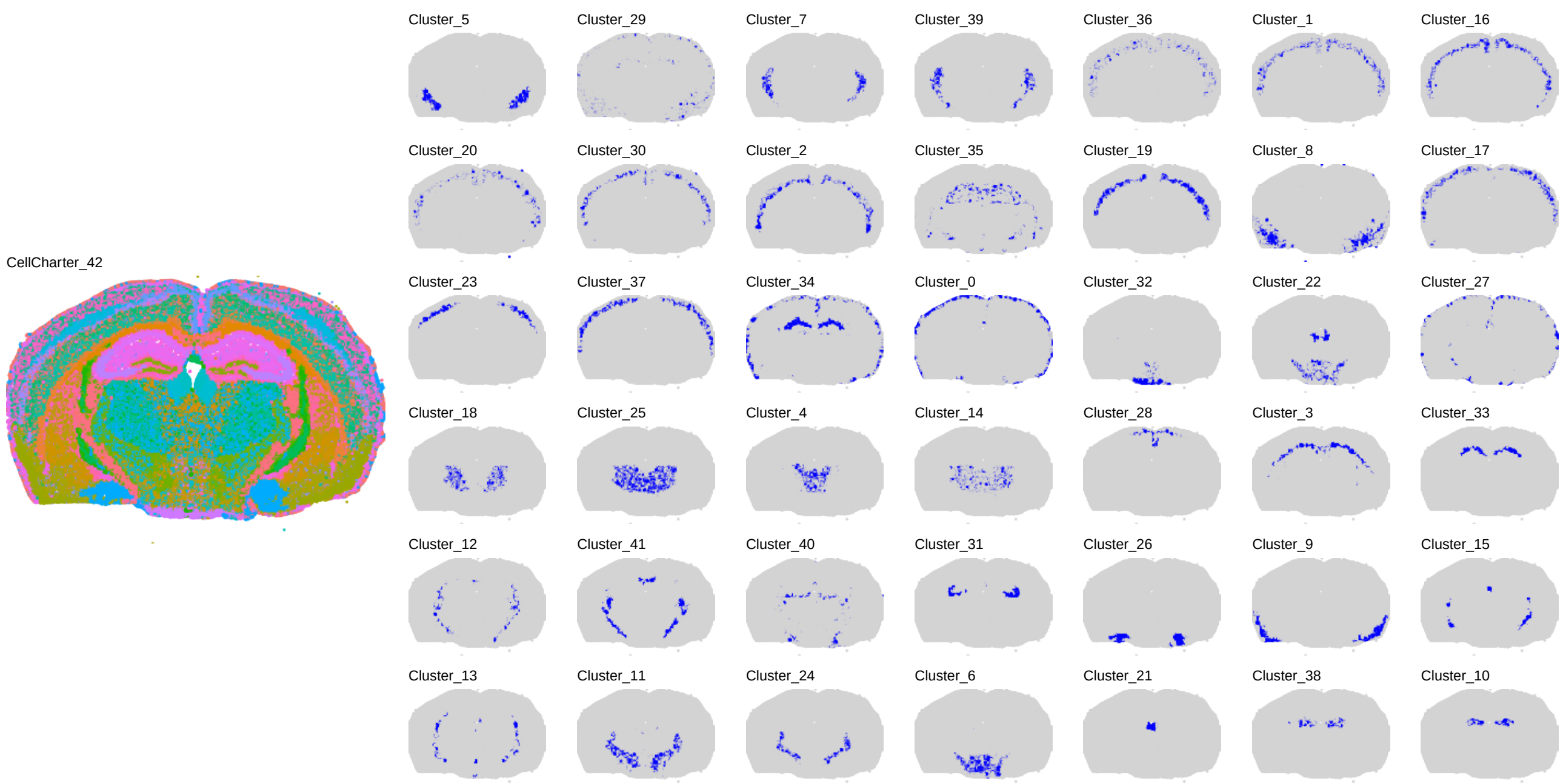
CellCharter_42
Cluster_5
Cluster_29
Cluster_7
Cluster_39
Cluster_36
Cluster_1
Cluster_16
Cluster_20
Cluster_30
Cluster_2
Cluster_35
Cluster_19
Cluster_8
Cluster_17
Cluster_23
Cluster_37
Cluster_34
Cluster_0
Cluster_32
Cluster_22
Cluster_27
Cluster_18
Cluster_25
Cluster_4
Cluster_14
Cluster_28
Cluster_3
Cluster_33
Cluster_12
Cluster_41
Cluster_40
Cluster_31
Cluster_26
Cluster_9
Cluster_15
Cluster_13
Cluster_11
Cluster_24
Cluster_6
Cluster_21
Cluster_38
Cluster_10

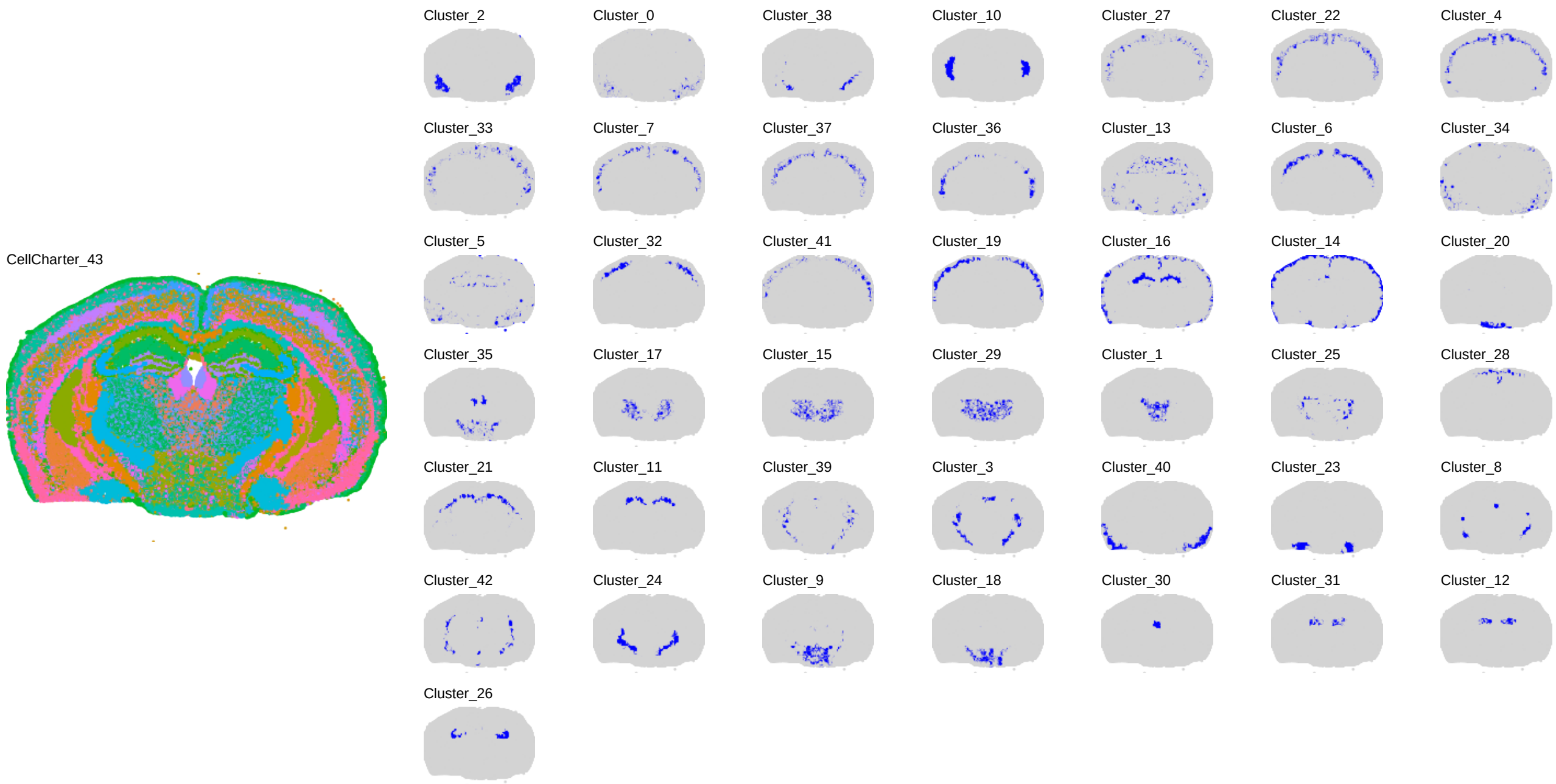
CellCharter_43
Cluster_2
Cluster_0
Cluster_38
Cluster_10
Cluster_27
Cluster_22
Cluster_4
Cluster_33
Cluster_7
Cluster_37
Cluster_36
Cluster_13
Cluster_6
Cluster_34
Cluster_5
Cluster_32
Cluster_41
Cluster_19
Cluster_16
Cluster_14
Cluster_20
Cluster_35
Cluster_17
Cluster_15
Cluster_29
Cluster_1
Cluster_25
Cluster_28
Cluster_21
Cluster_11
Cluster_39
Cluster_3
Cluster_40
Cluster_23
Cluster_8
Cluster_42
Cluster_24
Cluster_9
Cluster_18
Cluster_30
Cluster_31
Cluster_12
Cluster_26

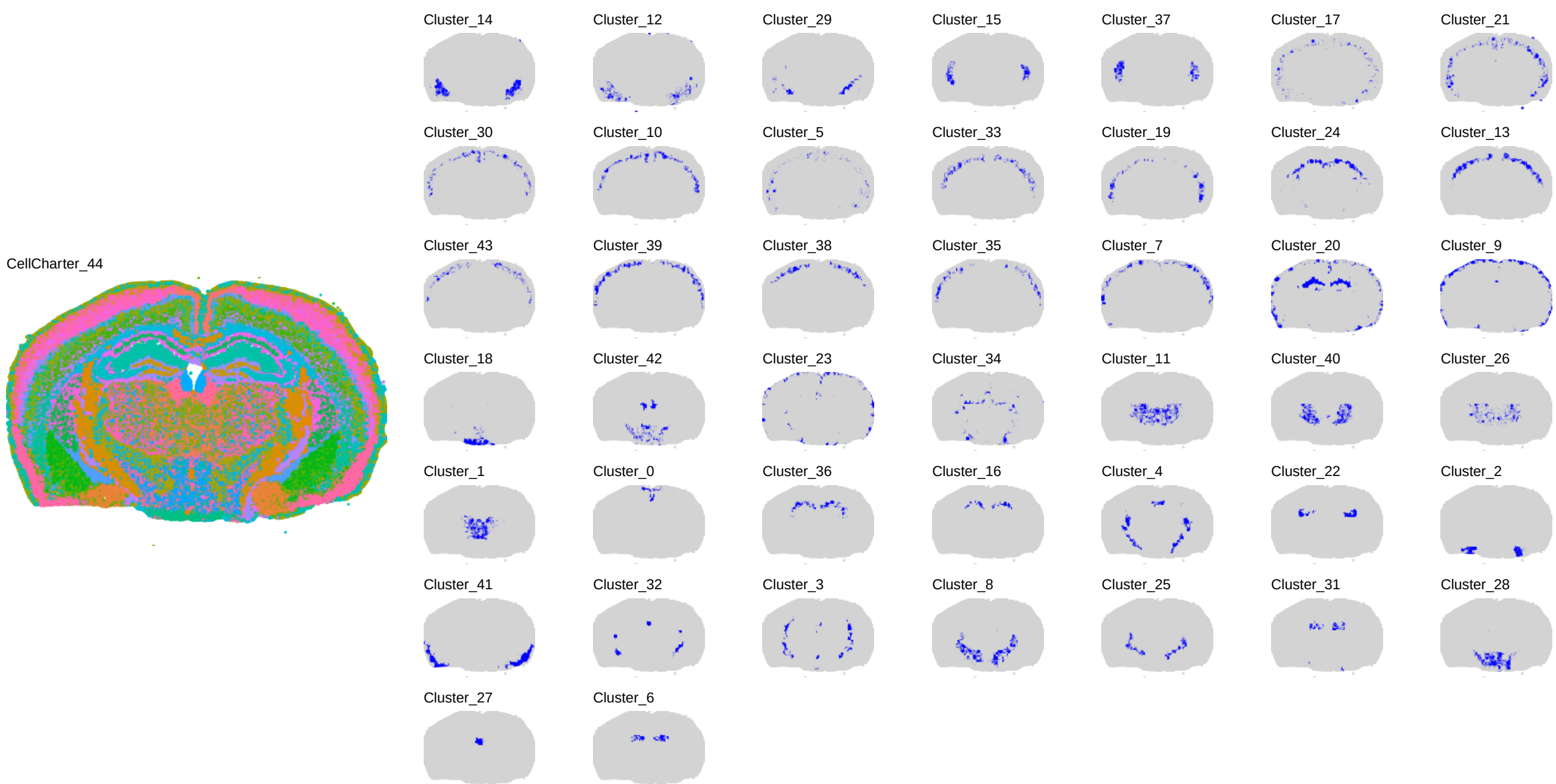
CellCharter_44
Cluster_14
Cluster_12
Cluster_29
Cluster_15
Cluster_37
Cluster_17
Cluster_21
Cluster_30
Cluster_10
Cluster_5
Cluster_33
Cluster_19
Cluster_24
Cluster_13
Cluster_43
Cluster_39
Cluster_38
Cluster_35
Cluster_7
Cluster_20
Cluster_9
Cluster_18
Cluster_42
Cluster_23
Cluster_34
Cluster_11
Cluster_40
Cluster_26
Cluster_1
Cluster_0
Cluster_36
Cluster_16
Cluster_4
Cluster_22
Cluster_2
Cluster_41
Cluster_32
Cluster_3
Cluster_8
Cluster_25
Cluster_31
Cluster_28
Cluster_27
Cluster_6

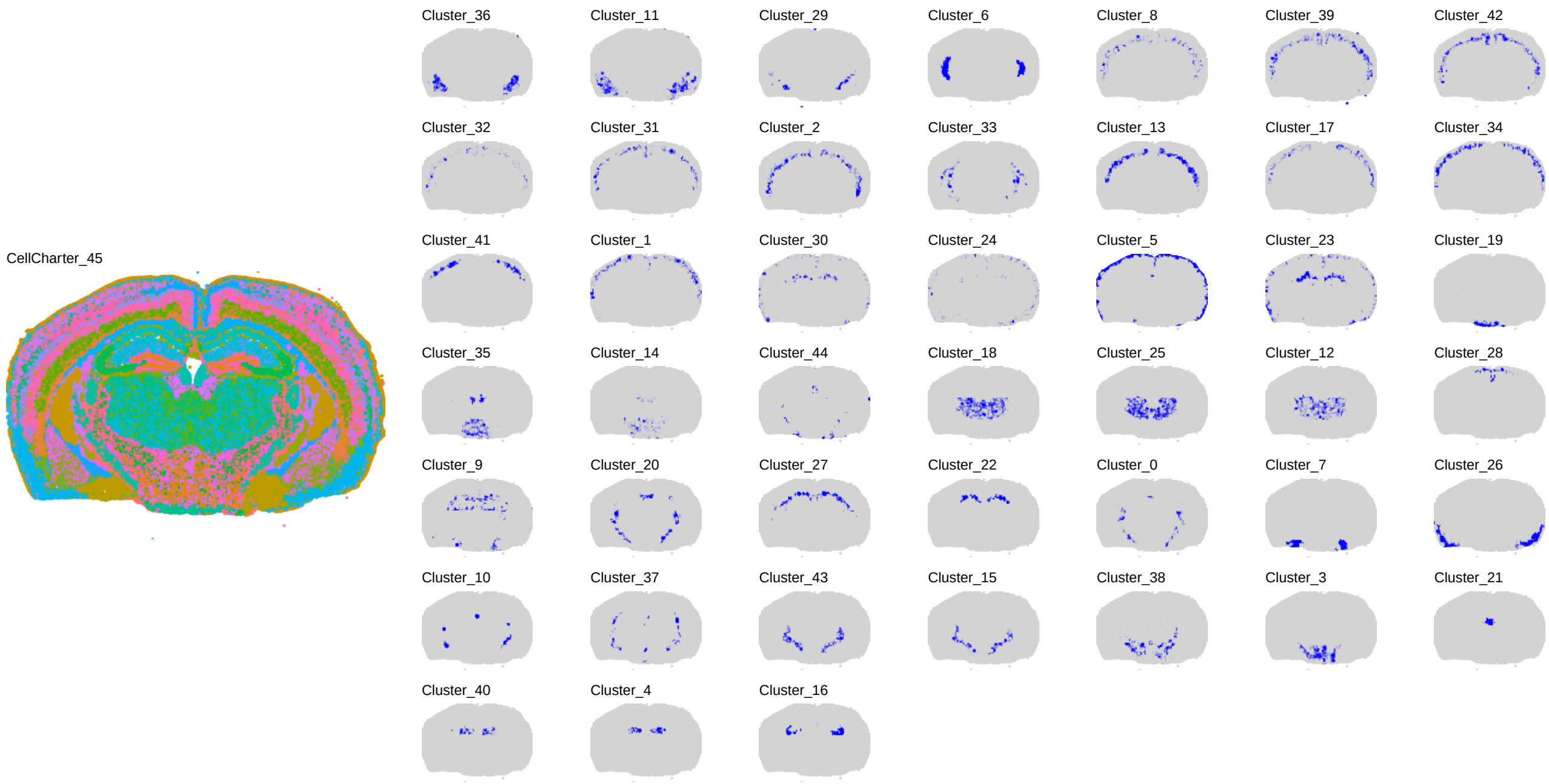
CellCharter_45
Cluster_36
Cluster_11
Cluster_29
Cluster_6
Cluster_8
Cluster_39
Cluster_42
Cluster_32
Cluster_31
Cluster_2
Cluster_33
Cluster_13
Cluster_17
Cluster_34
Cluster_41
Cluster_1
Cluster_30
Cluster_24
Cluster_5
Cluster_23
Cluster_19
Cluster_35
Cluster_14
Cluster_44
Cluster_18
Cluster_25
Cluster_12
Cluster_28
Cluster_9
Cluster_20
Cluster_27
Cluster_22
Cluster_0
Cluster_7
Cluster_26
Cluster_10
Cluster_37
Cluster_43
Cluster_15
Cluster_38
Cluster_3
Cluster_21
Cluster_40
Cluster_4
Cluster_16

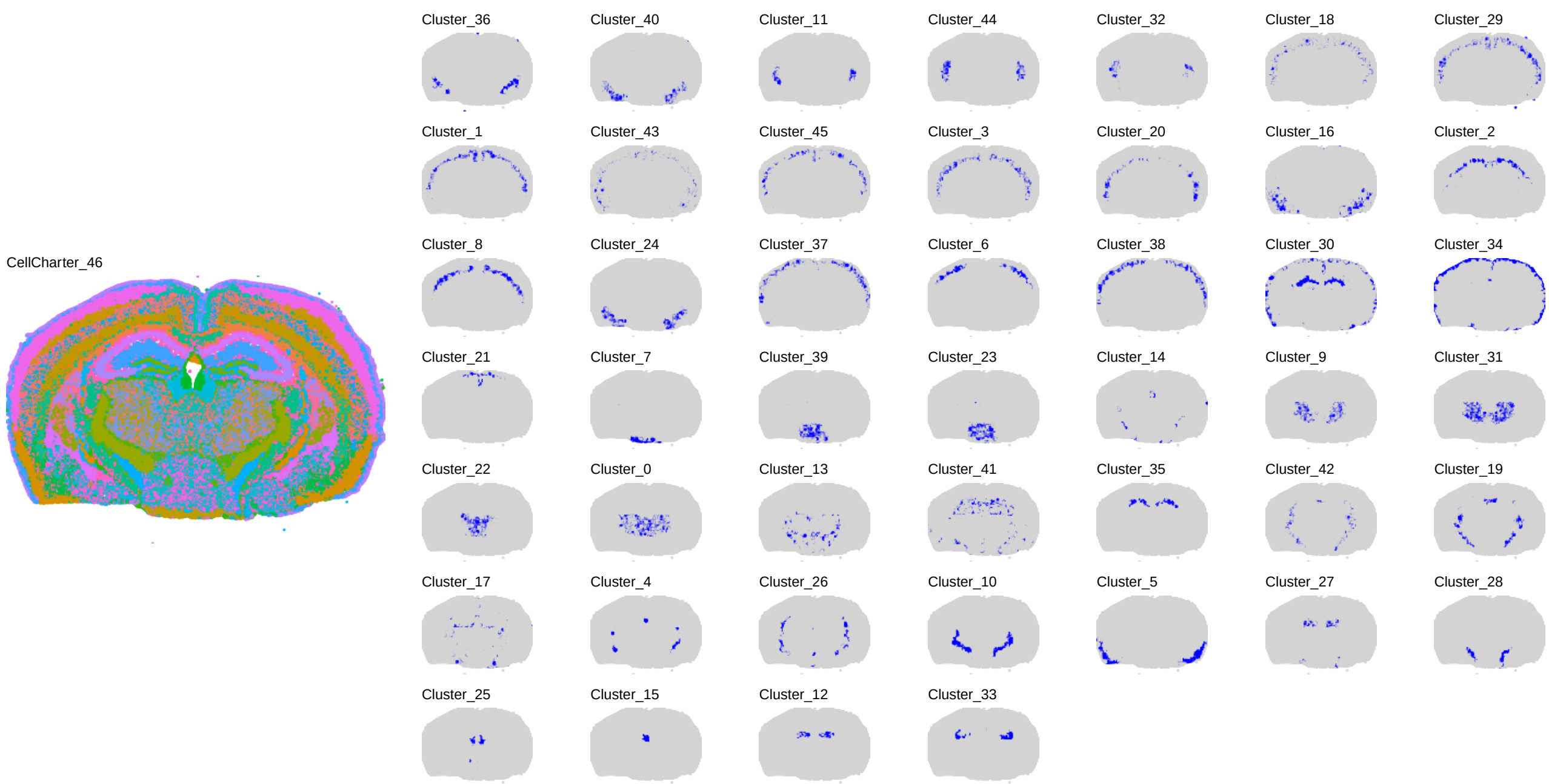
CellCharter_46
Cluster_36
Cluster_40
Cluster_11
Cluster_44
Cluster_32
Cluster_18
Cluster_29
Cluster_1
Cluster_43
Cluster_45
Cluster_3
Cluster_20
Cluster_16
Cluster_2
Cluster_8
Cluster_24
Cluster_37
Cluster_6
Cluster_38
Cluster_30
Cluster_34
Cluster_21
Cluster_7
Cluster_39
Cluster_23
Cluster_14
Cluster_9
Cluster_31
Cluster_22
Cluster_0
Cluster_13
Cluster_41
Cluster_35
Cluster_42
Cluster_19
Cluster_17
Cluster_4
Cluster_26
Cluster_10
Cluster_5
Cluster_27
Cluster_28
Cluster_25
Cluster_15
Cluster_12
Cluster_33

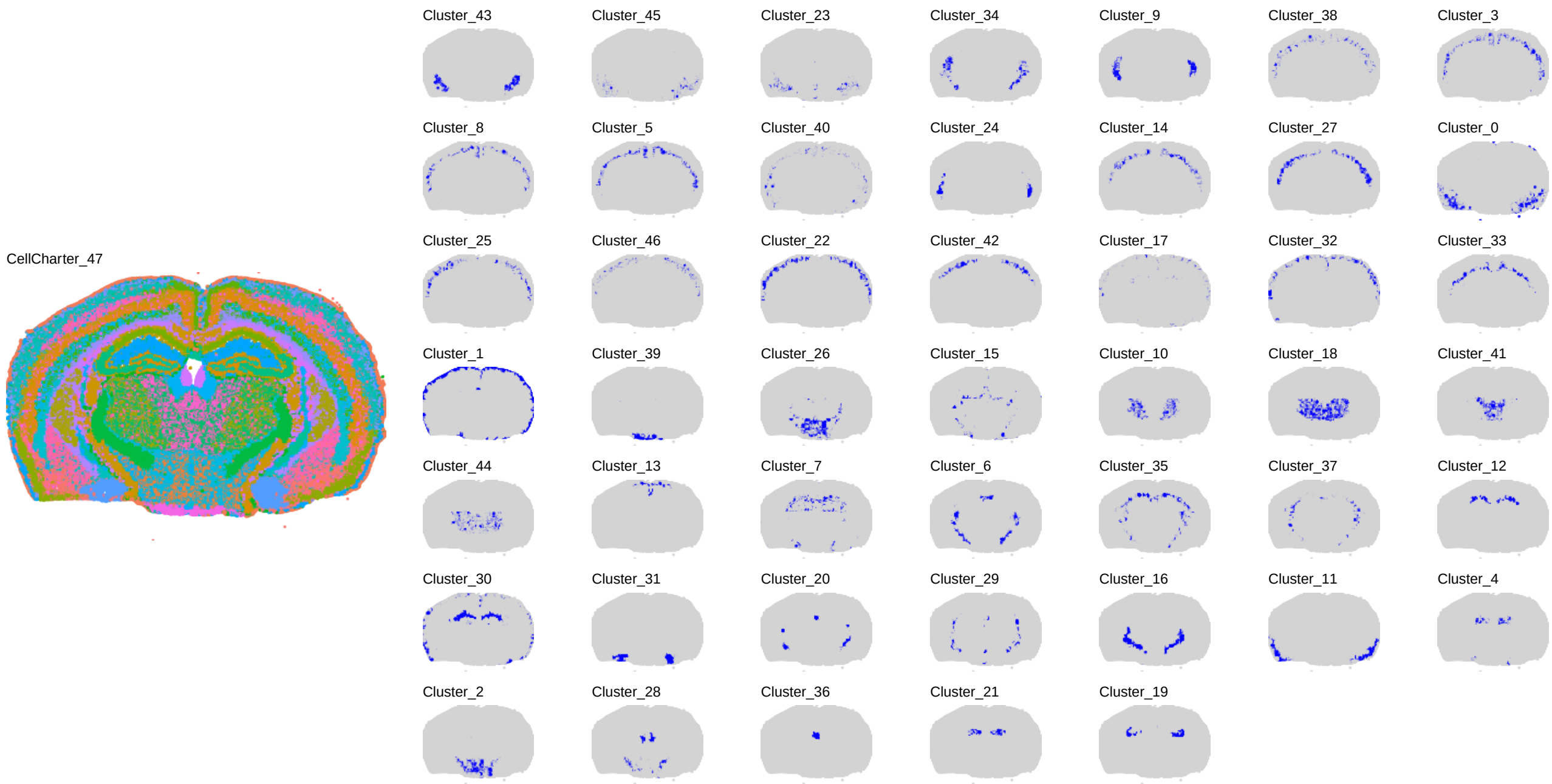
CellCharter_47
Cluster_43
Cluster_45
Cluster_23
Cluster_34
Cluster_9
Cluster_38
Cluster_3
Cluster_8
Cluster_5
Cluster_40
Cluster_24
Cluster_14
Cluster_27
Cluster_0
Cluster_25
Cluster_46
Cluster_22
Cluster_42
Cluster_17
Cluster_32
Cluster_33
Cluster_1
Cluster_39
Cluster_26
Cluster_15
Cluster_10
Cluster_18
Cluster_41
Cluster_44
Cluster_13
Cluster_7
Cluster_6
Cluster_35
Cluster_37
Cluster_12
Cluster_30
Cluster_31
Cluster_20
Cluster_29
Cluster_16
Cluster_11
Cluster_4
Cluster_2
Cluster_28
Cluster_36
Cluster_21
Cluster_19

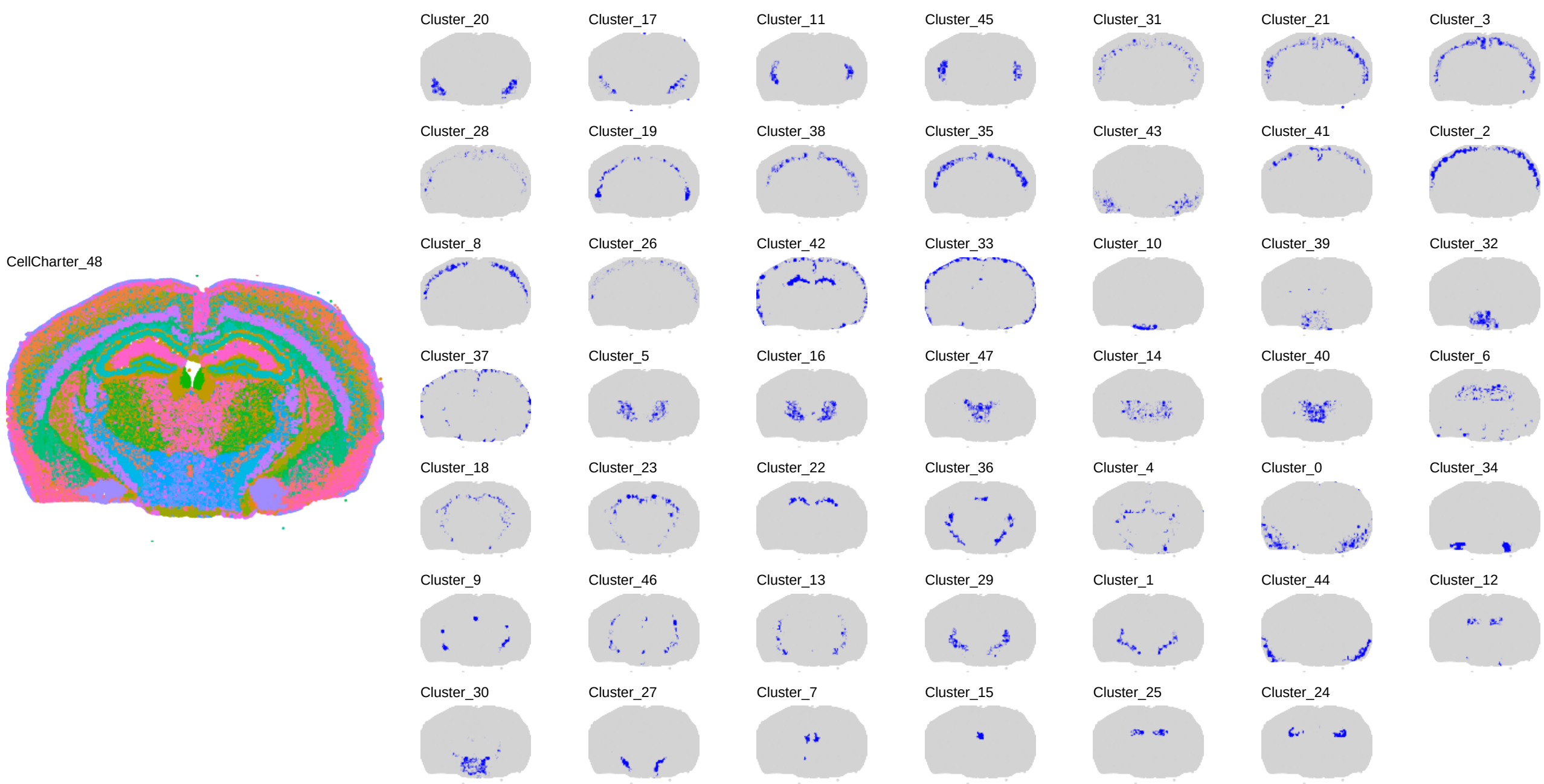
CellCharter_48
Cluster_20
Cluster_17
Cluster_11
Cluster_45
Cluster_31
Cluster_21
Cluster_3
Cluster_28
Cluster_19
Cluster_38
Cluster_35
Cluster_43
Cluster_41
Cluster_2
Cluster_8
Cluster_26
Cluster_42
Cluster_33
Cluster_10
Cluster_39
Cluster_32
Cluster_37
Cluster_5
Cluster_16
Cluster_47
Cluster_14
Cluster_40
Cluster_6
Cluster_18
Cluster_23
Cluster_22
Cluster_36
Cluster_4
Cluster_0
Cluster_34
Cluster_9
Cluster_46
Cluster_13
Cluster_29
Cluster_1
Cluster_44
Cluster_12
Cluster_30
Cluster_27
Cluster_7
Cluster_15
Cluster_25
Cluster_24

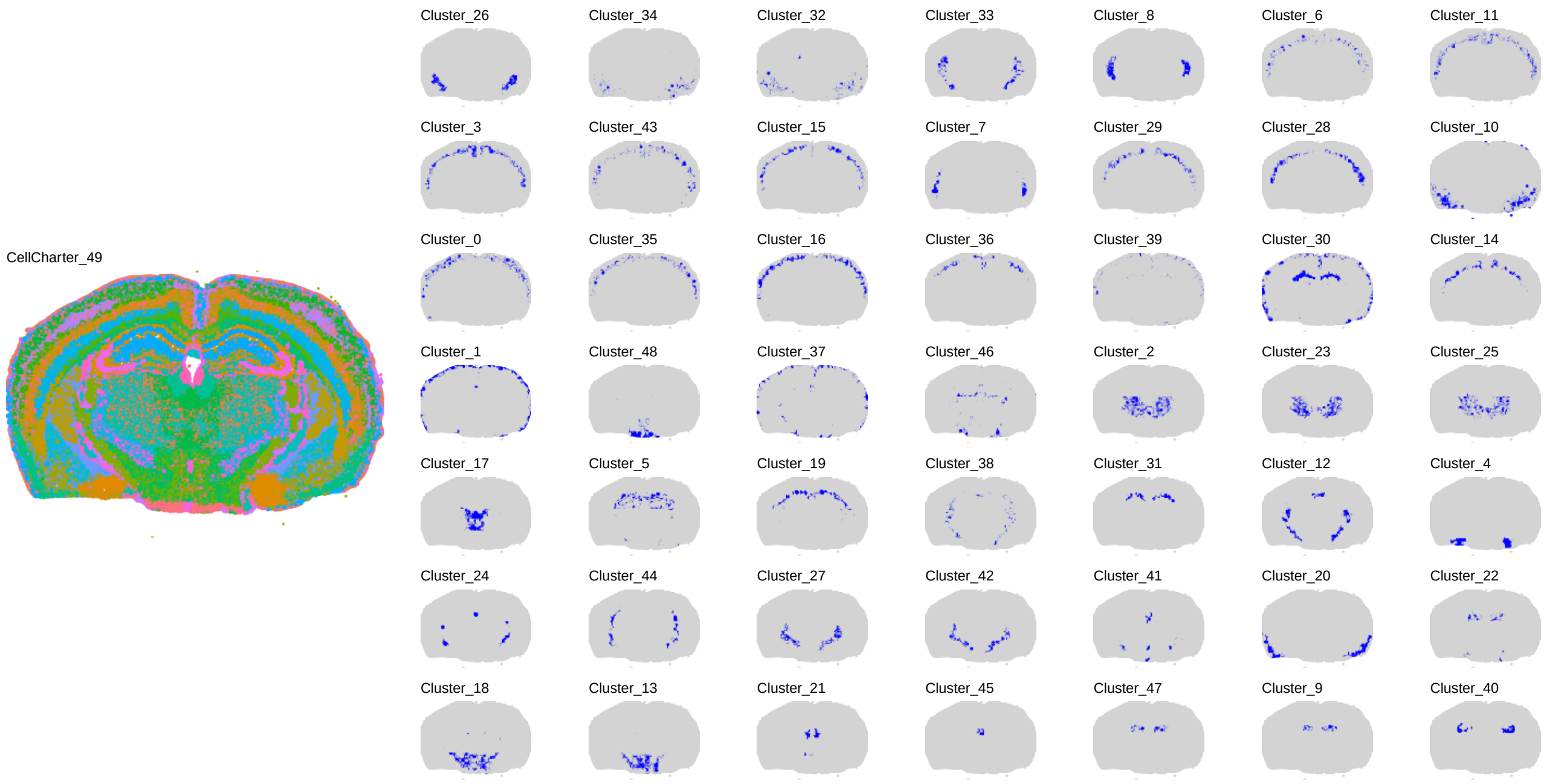
CellCharter_49
Cluster_26
Cluster_34
Cluster_32
Cluster_33
Cluster_8
Cluster_6
Cluster_11
Cluster_3
Cluster_43
Cluster_15
Cluster_7
Cluster_29
Cluster_28
Cluster_10
Cluster_0
Cluster_35
Cluster_16
Cluster_36
Cluster_39
Cluster_30
Cluster_14
Cluster_1
Cluster_48
Cluster_37
Cluster_46
Cluster_2
Cluster_23
Cluster_25
Cluster_17
Cluster_5
Cluster_19
Cluster_38
Cluster_31
Cluster_12
Cluster_4
Cluster_24
Cluster_44
Cluster_27
Cluster_42
Cluster_41
Cluster_20
Cluster_22
Cluster_18
Cluster_13
Cluster_21
Cluster_45
Cluster_47
Cluster_9
Cluster_40
