# Supplementary material for: Beyond benchmarking: an expert-guided consensus approach to spatially aware clustering
Source: Nat Methods. 2026 Aug 24;23(9):1814–26. doi: 10.1038/s41592-026-03194-8 (PMC13541621; doi:10.1038/s41592-026-03194-8)
Supplement: Supplementary file 2 [file 41592_2026_3194_MOESM2_ESM.pdf]

nature portfolio

Corresponding author(s): Naveed Ishaque

Last updated by author(s): Feb 21, 2026

Reporting Summary

Nature Portfolio wishes to improve the reproducibility of the work that we publish. This form provides structure for consistency and transparency in reporting. For further information on Nature Portfolio policies, see our Editorial Policies and the Editorial Policy Checklist.

Statistics

For all statistical analyses, confirm that the following items are present in the figure legend, table legend, main text, or Methods section.

n/a	Confirmed	
☐	☒	The exact sample size (*n*) for each experimental group/condition, given as a discrete number and unit of measurement
☐	☒	A statement on whether measurements were taken from distinct samples or whether the same sample was measured repeatedly
☐	☒	The statistical test(s) used AND whether they are one- or two-sided<br>*Only common tests should be described solely by name; describe more complex techniques in the Methods section.*
☒	☐	A description of all covariates tested
☒	☐	A description of any assumptions or corrections, such as tests of normality and adjustment for multiple comparisons
☐	☒	A full description of the statistical parameters including central tendency (e.g. means) or other basic estimates (e.g. regression coefficient) AND variation (e.g. standard deviation) or associated estimates of uncertainty (e.g. confidence intervals)
☐	☒	For null hypothesis testing, the test statistic (e.g. *F*, *t*, *r*) with confidence intervals, effect sizes, degrees of freedom and *P* value noted<br>*Give P values as exact values whenever suitable.*
☒	☐	For Bayesian analysis, information on the choice of priors and Markov chain Monte Carlo settings
☒	☐	For hierarchical and complex designs, identification of the appropriate level for tests and full reporting of outcomes
☒	☐	Estimates of effect sizes (e.g. Cohen's *d*, Pearson's *r*), indicating how they were calculated

Our web collection on statistics for biologists contains articles on many of the points above.

Software and code

Policy information about availability of computer code

Data collection

Exact scripts to downloaded data are deposited on https://github.com/SpatialHackathon/SACCELERATOR/tree/native/data.

Data analysis

The SACCELERATOR workflow and all relevant code to run the analysis are available as open-source software on GitHub https://github.com/SpatialHackathon/SACCELERATOR. All code for reproducing results presented in this study, including figures and collected ARIs for the meta benchmark are available via https://github.com/SpatialHackathon/SpaceHack2023_study. Links to raw data are provided in the Datasets section. All filtered data, intermediate results, and final results are deposited on Zenodo https://zenodo.org/records/15487520.

Installation scripts for all tools evaluated in this study are deposited on: https://github.com/SpatialHackathon/SACCELERATOR/tree/native/method
Scripts that perform all analysis presented in the study are deposited on: https://github.com/SpatialHackathon/SpaceHack2023_study

Software and libraries used in the study:
Snakemake (v8.14.0)
SLURM (v21.08.5)
scran (version 1.34.0)
scater(version 1.34.1)
dbscan(version 1.2.0)
clue}(version 0.3_6)

Spatially aware clustering tools used:
MAPLE 0.99.1 (b173e89) 2024 R Jeon et al. (2024)

BANKSY 0.99.7 (dbda6fd) 2023 R (+ Python) Singhal et al. (2023)
PRECAST 1.6.3 2023 R Liu et al. (2023)
GraphST 1.1.1 2023 Python Long et al. (2023)
SPICEMIX 0.0.71 2023 Python Chidester et al. (2023)
CellCharter 0.2.0 2023 Python Varrone et al. (2023)
STAGATE 1.0.1 (48ce7f8) 2022 Python Dong & Zhang (2022)
scMEB 1.1 2022 R Yang et al. (2022)
SpaceFlow 1.0.4 2022 Python Ren et al. (2022)
DR-SC 3.3 2022 R Liu et al. (2022)
SCAN-IT 0.1 (ebf3894) 2022 Python Cang et al. (2022)
SOTIP 1.0 (d3b762c) 2022 Python Yuan et al. (2022)
Stardust 2.0 (f1b5417) 2022 R Avesani et al. (2022)
BASS 1.1.0.016 (37980c9) 2022 R Li & Zhou (2022)
spatialGE 1.2.0.0000 2022 R Ospina et al. (2022)
SpaGCN 1.2.7 2021 Python Hu et al. (2021)
BayesSpace 1.10.1 2021 R Zhao et al. (2021)
SEDR 1.0.0 (8c273af) 2021 Python Fu et al. (2021)
MERINGUE 1.0 (ca9e2cc) 2021 R Miller et al. (2021)
Giotto 4.0.0 2021 R (+ Python) Dries et al. (2021)
Scanpy 1.10.0 2018 Python Wolf et al. (2018)
Seurat 5.0.1 2018 R Butler et al. (2018)

For manuscripts utilizing custom algorithms or software that are central to the research but not yet described in published literature, software must be made available to editors and reviewers. We strongly encourage code deposition in a community repository (e.g. GitHub). See the Nature Portfolio guidelines for submitting code & software for further information.

Data

Policy information about availability of data

All manuscripts must include a data availability statement. This statement should provide the following information, where applicable:

- Accession codes, unique identifiers, or web links for publicly available datasets
- A description of any restrictions on data availability
- For clinical datasets or third party data, please ensure that the statement adheres to our policy

Raw data download links (raw data and annotations were downloaded from the same source unless explicitly mentioned):

The STARmap_plus dataset was downloaded from the following sources: https://zenodo.org/records/8327576 (code from : https://github.com/wanglab-broad/mCNS-atlas).
"The abc_atlas_wmb_thalamus dataset was downloaded from the following sources: https://knowledge.brain-map.org/data/LVDBJAW8BI5YSS1QUBG/summary (data Access Jupyter Notebooks: https://alleninstitute.github.io/abc_atlas_access/intro.html)."
The libd_dlpfc dataset was downloaded from the following sources: https://research.libd.org/spatialLIBD/.
The osmfish_Ssp dataset was downloaded from the following sources: https://linnarssonlab.org/osmFISH/osmFISH_SScortex_mouse_all_cells.loom.
The spatialDLPFC dataset was downloaded from the following sources: https://research.libd.org/spatialLIBD/.
The stereoseq_developing_Drosophila_embryos_larvae dataset was downloaded from the following sources: https://db.cngb.org/stomics/datasets/STDS0000060.
The stereoseq_mouse_embryo dataset was downloaded from the following sources: https://ftp.cngb.org/pub/SciRAID/stomics/STDS0000058/stomics/; annotation downloaded from https://zenodo.org/records/10516814.
The visium_breast_cancer_SEDR dataset was downloaded from the following sources: https://www.10xgenomics.com/resources/datasets/human-breast-cancer-block-a-section-1-1-standard-1-1-0; processed data: https://github.com/JinmiaoChenLab/SEDR_analyses/tree/master/data/BRCA1.
The visium_chicken_heart dataset was downloaded from the following sources: https://www.ncbi.nlm.nih.gov/geo/query/acc.cgi?acc=GSE149457; https://github.com/madhavmantri/chicken_heart.
The xenium-breast-cancer dataset was downloaded from the following sources: https://www.10xgenomics.com/datasets/xenium-ffpe-human-breast-with-custom-add-on-panel-1-standard; annotation downloaded from: https://zenodo.org/records/10516814.
The xenium-mouse-brain-SergioSalas dataset was downloaded from the following sources: https://www.10xgenomics.com/datasets/fresh-frozen-mouse-brain-replicates-1-standard.
The slideseq2_olfactory_bulbdataset was downloaded from the following sources: https://db.cngb.org/stomics/datasets/STDS0000172/summary.
The merfish_aging_mouse_brain dataset was downloaded from the following sources: https://cellxgene.cziscience.com/collections/31937775-0602-4e52-a799-b6acdd2bac2e.
The visium_hd_cancer_colon dataset was downloaded from the following sources: https://www.10xgenomics.com/datasets/visium-hd-cytassist-gene-expression-libraries-of-human-crc.
The merfish_devheart dataset was downloaded from the following sources: https://datadryad.org/stash/dataset/doi:10.5061/dryad.w0vt4b8vp.
The stereoseq_liver dataset was downloaded from the following sources: https://db.cngb.org/stomics/lista/spatial/.
The cosmx_lung dataset was downloaded from the following sources: https://staging.nanostring.com/products/cosmx-spatial-molecular-imager/ffpe-dataset/nsclc-ffpe-dataset/; annotations downloaded from: https://zenodo.org/records/10516814.
The cosmx_liver dataset was downloaded from the following sources: https://nanostring.com/products/cosmx-spatial-molecular-imager/ffpe-dataset/human-liver-rna-ffpe-dataset/.

Research involving human participants, their data, or biological material

Policy information about studies with human participants or human data. See also policy information about sex, gender (identity/presentation), and sexual orientation and race, ethnicity and racism.

Reporting on sex and gender	N/A
Reporting on race, ethnicity, or other socially relevant groupings	N/A
Population characteristics	N/A
Recruitment	N/A
Ethics oversight	N/A

Note that full information on the approval of the study protocol must also be provided in the manuscript.

Field-specific reporting

Please select the one below that is the best fit for your research. If you are not sure, read the appropriate sections before making your selection.

☒ Life sciences ☐ Behavioural & social sciences ☐ Ecological, evolutionary & environmental sciences

For a reference copy of the document with all sections, see nature.com/documents/nr-reporting-summary-flat.pdf

Life sciences study design

All studies must disclose on these points even when the disclosure is negative.

Sample size	N/A
Data exclusions	N/A
Replication	N/A
Randomization	N/A
Blinding	N/A

Reporting for specific materials, systems and methods

We require information from authors about some types of materials, experimental systems and methods used in many studies. Here, indicate whether each material, system or method listed is relevant to your study. If you are not sure if a list item applies to your research, read the appropriate section before selecting a response.

Materials & experimental systems

n/a	Involved in the study	
☒	☐	Antibodies
☒	☐	Eukaryotic cell lines
☒	☐	Palaeontology and archaeology
☒	☐	Animals and other organisms
☒	☐	Clinical data
☒	☐	Dual use research of concern
☒	☐	Plants

Methods

n/a	Involved in the study	
☒	☐	ChIP-seq
☒	☐	Flow cytometry
☒	☐	MRI-based neuroimaging

Plants

Seed stocks	N/A
Novel plant genotypes	N/A
Authentication	N/A
