# Supplementary material for: Beyond benchmarking: an expert-guided consensus approach to spatially aware clustering
Source: Nat Methods. 2026 Aug 24;23(9):1814–26. doi: 10.1038/s41592-026-03194-8 (PMC13541621; doi:10.1038/s41592-026-03194-8)
Supplement: Supplementary file 3 [file 41592_2026_3194_MOESM3_ESM.pdf]

nature portfolio

Peer Review File

Beyond benchmarking: an expert-guided consensus approach to spatially aware clustering

Corresponding Author: Dr Naveed Ishaque

This file contains all editorial decision letters in order by version, followed by all author rebuttals in order by version.

Version 0:

Decision Letter:

22nd Apr 2025

Dear Dr Ishaque,

Thank you for your inquiry about submitting your manuscript, "Beyond Benchmarking: An Expert-Guided Consensus Approach to Spatially Aware Clustering" to Nature Methods. The paper sounds like it may be of interest, and should fit the scope of the journal. We would be willing to consider it for publication in Nature Methods.

Of course, it is very difficult to judge a paper based only on the limited information available in a presubmission inquiry. Therefore I am sure you understand that we cannot promise to send your paper out for peer review and must read it in its entirety before deciding if this would be suitable.

Please keep in mind that the journal is aimed at a large, interdisciplinary audience and places a strong emphasis on the practical value of the work presented for basic research in the life sciences. We strongly encourage you to include data to validate method performance and demonstrate its general applicability.

You will find our Guide to Authors at http://www.nature.com/naturemethods to assist you in preparing your manuscript. However, it is not necessary at this stage to spend major effort adhering to our detailed formatting instructions.

Thank you for your interest in Nature Methods.

Sincerely,
Madhura

Madhura Mukhopadhyay, PhD
Senior Editor
Nature Methods

Version 1:

Decision Letter:

21st Aug 2025

Dear Naveed,

Your Article, "Beyond benchmarking: an expert-guided consensus approach to spatially aware clustering", has now been seen by 3 reviewers. As you will see from their comments below, although the reviewers find your work of considerable potential

interest, they have raised a number of concerns. We continue to be interested in potentially publishing your manuscript in Nature Methods, but would like to evaluate your response to these concerns before we reach a final decision on publication.

We therefore invite you to revise your manuscript to address these concerns and add appropriate metrics, use cases and discussions.

We are committed to providing a fair and constructive peer-review process. Do not hesitate to contact us if there are specific requests from the reviewers that you believe are technically impossible or unlikely to yield a meaningful outcome.

When revising your paper:

* include a point-by-point response to the reviewers and to any editorial suggestions

* please underline/highlight any additions to the text or areas with other significant changes to facilitate review of the revised manuscript

* address the points listed described below to conform to our open science requirements

* ensure it complies with our general format requirements as set out in our guide to authors at www.nature.com/naturemethods

* resubmit all the necessary files by using the link below to access your home page

Link Redacted

Note: This URL links to your confidential account and associated information about manuscripts you may have submitted, or that you are reviewing for us. If you wish to forward this email to co-authors, please delete this link.

We hope to receive your revised paper within three months. If you are substantially delayed, please let us know. In this event, we will still be happy to reconsider your paper at a later date so long as nothing similar has been accepted for publication at Nature Methods or published elsewhere.

OPEN SCIENCE REQUIREMENTS

REPORTING SUMMARY
When revising your manuscript, please update your reporting summary.

Reporting summary: https://www.nature.com/documents/nr-reporting-summary.zip

If your paper includes custom software, we also ask you to complete a supplemental reporting summary.

Software supplement: https://www.nature.com/documents/nr-software-policy.pdf

Please submit these with your revised manuscript. They will be available to reviewers to aid in their evaluation if the paper is re-reviewed. If you have any questions about the checklist, please see http://www.nature.com/authors/policies/availability.html or contact me.

Please note that these forms are dynamic ‘smart pdfs’ and must therefore be downloaded and completed in Adobe Reader. We will then flatten them for ease of use by the reviewers. If you would like to reference the guidance text as you complete the template, please access these flattened versions at http://www.nature.com/authors/policies/availability.html.

For any revision that includes light microscopy data, we ask our authors to please include a completed light microscopy reporting table [https://www.nature.com/documents/Light_microscopy_reporting_table.xlsx] to ensure the methods are described thoroughly. The table will be available to reviewers and ultimately published should the manuscript be accepted at the journal.

EXTENDED DATA FIGURES
When re-submitting your manuscript, please ensure that any supplementary figures and tables that are crucial to the manuscript’s conclusions are converted into Extended Data figures and tables to increase visibility of these data. Extended Data figures and tables are online-only (present in the online PDF and full-text HTML versions of the paper), peer-reviewed display items that provide essential background to the article but are not included in the main article due to space constraints. A maximum of ten Extended Data display items (figures and tables) is permitted.

DATA AVAILABILITY
We strongly encourage you to deposit all new data associated with the paper in a persistent repository where they can be

freely and enduringly accessed. We recommend submitting the data to discipline-specific and community-recognized repositories; a list of repositories is provided here: http://www.nature.com/sdata/policies/repositories

All novel DNA and RNA sequencing data, protein sequences, genetic polymorphisms, linked genotype and phenotype data, gene expression data, macromolecular structures, and proteomics data must be deposited in a publicly accessible database, and accession codes and associated hyperlinks must be provided in the “Data Availability” section.

For papers containing bioimaging data, we strongly recommend depositing the data to Bioimage Archive (https://www.ebi.ac.uk/bioimage-archive/). Associated accession codes and hyperlinks should be provided in the "Data Availability" section.

Refer to our data policies here: https://www.nature.com/nature-research/editorial-policies/reporting-standards#availability-of-data

To further increase transparency, we encourage you to provide, in tabular form, the data underlying the graphical representations used in your figures. This is in addition to our data-deposition policy for specific types of experiments and large datasets. For readers, the source data will be made accessible directly from the figure legend. Spreadsheets can be submitted in .xls, .xlsx or .csv formats. Only one (1) file per figure is permitted: thus if there is a multi-paneled figure the source data for each panel should be clearly labeled in the csv/Excel file; alternately the data for a figure can be included in multiple, clearly labeled sheets in an Excel file. File sizes of up to 30 MB are permitted. When submitting source data files with your manuscript please select the Source Data file type and use the Title field in the File Description tab to indicate which figure the source data pertains to.

Please include a “Data availability” subsection in the Online Methods. This section should inform readers about the availability of the data used to support the conclusions of your study, including accession codes to public repositories, references to source data that may be published alongside the paper, unique identifiers such as URLs to data repository entries, or data set DOIs, and any other statement about data availability. At a minimum, you should include the following statement: “The data that support the findings of this study are available from the corresponding author upon request”, describing which data is available upon request and mentioning any restrictions on availability. If DOIs are provided, please include these in the Reference list (authors, title, publisher (repository name), identifier, year). For more guidance on how to write this section please see: http://www.nature.com/authors/policies/data/data-availability-statements-data-citations.pdf

CODE AVAILABILITY
Please include a “Code Availability” subsection in the Online Methods which details how your custom code is made available. Only in rare cases (where code is not central to the main conclusions of the paper) is the statement “available upon request” allowed (and reasons should be specified).

We request that you deposit code in a DOI-minting repository such as Zenodo, Gigantum or Code Ocean and cite the DOI in the Reference list. We also request that you use code versioning and provide a license.

For more information on our code sharing policy and requirements, please see: https://www.nature.com/nature-research/editorial-policies/reporting-standards#availability-of-computer-code

ORCID
Nature Methods is committed to improving transparency in authorship. As part of our efforts in this direction, we are now requesting that all authors identified as ‘corresponding author’ on published papers create and link their Open Researcher and Contributor Identifier (ORCID) with their account on the Manuscript Tracking System (MTS), prior to acceptance. This applies to primary research papers only. ORCID helps the scientific community achieve unambiguous attribution of all scholarly contributions. You can create and link your ORCID from the home page of the MTS by clicking on ‘Modify my Springer Nature account’. For more information please visit please visit www.springernature.com/orcid.

Please do not hesitate to contact me if you have any questions or would like to discuss these revisions further. We look forward to seeing your revised manuscript and thank you for the opportunity to consider your work.

Best regards,
Madhura

Madhura Mukhopadhyay, PhD
Senior Editor
Nature Methods

Reviewers' Comments:

Reviewer #1 (Remarks to the Author):

Sun et al. presents SACCELERATOR, a modular and extensible framework for evaluating spatially aware clustering methods in spatial transcriptomics. The authors argue that traditional benchmarks relying on fixed ground truth annotations are limited by subjectivity, granularity bias, and lack of scalability. To address these issues, they proposed a consensus-guided, expert-in-the-loop approach that integrates results from multiple spatial clustering methods to identify robust clustering patterns and highlight method disagreements through entropy-based spatial metrics. The framework is validated across many datasets, including case studies in brain and colorectal cancer tissues.

This is a timely and well-motivated contribution to the field. Benchmarking studies are common in spatial omics, but many rely on so-called ground truths that are often based on H&E images or prior biological assumptions. These annotations are prone to human error, granularity mismatch, and conceptual bias, and different benchmarking studies have led to contradictory conclusions.

This manuscript directly addresses those issues by showing, through extensive analyses across multiple datasets and platforms, that:

• Spatially aware clustering method performance is inconsistent across benchmarks;
• Ground truth annotations often penalize biologically meaningful patterns;
• A consensus approach provides a more robust, reproducible, and flexible foundation for evaluating spatially aware clustering results;
• An expert-in-the-loop workflow reflects real research practice more faithfully than static evaluations.
• The authors provide an open-source, modular platform that the community can build upon. This promotes transparency and reproducibility.

The consensus strategy is not algorithmically novel, but its application in this context is pragmatic and impactful. The authors also tackled scalability, modularity, and extensibility in a way that adds real value for the community.

Areas for improvement

1. Expert input is valuable but subjective; more structured documentation of expert feedback or annotation protocols would enhance reproducibility.
2. Consensus method scalability could be further quantified, especially as datasets reach millions of cells.
3. A more systematic exploration of clustering granularity and its biological interpretability would strengthen the guidance for users.
4. Some reliance on ARI remains; alternatives like biological validation or application-driven metrics could further support conclusions.

While the core innovation is not on the methods, this is a thoughtful and impactful study that moves the field beyond the limitations of rigid benchmarking toward a more practical and biologically grounded evaluation paradigm. The authors' analyses are rigorous and comprehensive, and the framework is well-engineered, and the case studies are compelling. I found this work much better than those pure benchmarking papers previously published in Nature Methods, and I believe it merits publication with minor revisions.

Reviewer #2 (Remarks to the Author):

Summary of key results

The authors introduce SACCELERATOR, an open-source consensus framework that integrates 22 spatially-aware clustering methods across 15 spatial-omics datasets. The main claims are: (i) current benchmarks are biased toward Visium/brain data and unreliable anatomical "ground-truth" labels; (ii) algorithm performance is highly context-dependent; and (iii) an expert-in-the-loop consensus strategy improves reproducibility and biological interpretability.

Originality & significance

Moving from static benchmarking to a dynamic, expert-guided consensus platform is highly original. No previous study has systematically examined method generalizability across healthy vs. cancer tissues while providing a practical feedback mechanism.

Data & methodology

1. Single-metric focus

I agree with the point of traditional evaluation metrics do not always reflect result quality and it is good to see the authors introduce both traditional and spatially aware metrics. However, all main figures use Adjusted Rand Index (ARI) only, despite claims of evaluating 17 metrics. Please add a supplementary heat-map or radar plot that includes NMI, SpatialARI, CHAOS and two stability scores to substantiate metric breadth. It would also be interesting to see how other metrics agree with the expert pathology ranking in figure 5. I encourage more detailed discussion regarding the 17 metrics and how they agree with the ground truth as well as clustering results.

In supplementary figure 2, SpatialARI has a correlation of 1 with ARI – is this expected? Could you extend the correlation matrix to all the datasets to see if the similarities and differences in metrics are consistent?

2. CME calculation and algorithmic similarity

The Consensus Entropy Metric (CME) is computed on “concordant” partitions pre-selected by high pairwise-ARI and low smoothness-entropy. This risks pooling methods with similar modelling assumptions, so low CME could reflect algorithmic redundancy rather than biological certainty. Analysis that might help answer this:

a) Algorithm-family stratification: group methods (graph-convolution, Bayesian, matrix-factorization, etc.), keep ≤2 representatives per family, and recompute CME.

b) External perturbation test: randomly erase 20 % of Layer 4 labels in slice 151673 and compare CME at perturbed vs. intact boundaries by permutation testing.

c) Intra- vs. inter-family ARI distribution to demonstrate that the concordant set is not dominated by a single family.

3. Consensus methods

The manuscript implements LCA, K-modes, and weight-based consensus without justification. Please add a Methods paragraph that states (i) why these three were selected, (ii) their underlying assumptions (e.g., local independence in LCA), and (iii) recommended usage scenarios.

4. Spatial-omics technology classification and comparison

The current wording (lines 152–53) conflates technology names with resolution tiers:
Consider revising to a resolution-based description:
“Technologies operating at bulk (original 55 µm Visium), cellular (~5–10 µm; Slide-seqV2, Xenium), and sub-cellular (<2 µm; Visium HD, MERFISH, STARmap PLUS, Stereo-seq, CosMx).”

The manuscript includes 10 different platforms across various datasets (including CosMX, MERSCOPE, MERFISH, STARmap, Xenium, Slideseq, Stereo-seq, Visium, osmFISH, and Visium HD). However only 2 case studies are discussed in detail for Visium HD and MERFISH. ‘MERSCOPE’ appears 3 times in the manuscript in the Methods section and I cannot see any discussion about the Visium chicken heart data. More results and discussion that brings out any insights gained across a wider range of platforms would be beneficial.

Page 18 ‘Slide-seqV2 mouse brain olfactory bulb dataset’ link appears to point to a Stomics dataset – please check and correct to the appropriate link.

5. Presentation of results

Figure 1E is hard to read (multi-colours within each violin plot representing different paramter configurations). A different display (separate violins per config?) might be easier to interpret.

Figure 2B. Consider extending this analysis other sample types (human cortex may be an edge case here, compared to mouse brain / breast cancer samples for example).

Supplementary Figure 3. Visium and Visium HD should use different colours to help the reader distinguish between them, as there are large differences in spatial resolution.

Reviewer #3 (Remarks to the Author):

I co-reviewed this manuscript with one of the reviewers who provided the listed reports. This is part of the Nature Methods initiative to facilitate training in peer review and to provide appropriate recognition for Early Career Researchers who co-review manuscripts.

Reviewer #3 (Remarks on code availability):

This codebase provides excellent value for researchers in spatial omics. While initial setup requires technical expertise, the modular design and comprehensive documentation make it a worthwhile investment for laboratories conducting spatial clustering analysis.

Version 2:

Decision Letter:

Our ref: NMETH-A60725B

13th Jan 2026

Dear Naveed,

Thank you for submitting your revised manuscript "Beyond benchmarking: an expert-guided consensus approach to spatially aware clustering" (NMETH-A60725B). It has now been seen by the original referees and their comments are below. The reviewers find that the paper has improved in revision, and therefore we'll be happy in principle to publish it in Nature Methods, pending minor revisions to satisfy the referees' final requests and to comply with our editorial and formatting guidelines.

We are now performing detailed checks on your paper and will send you a checklist detailing our editorial and formatting requirements within two weeks or so. Please do not upload the final materials and make any revisions until you receive this additional information from us.

TRANSPARENT PEER REVIEW
Nature Methods offers a transparent peer review option for new original research manuscripts submitted from 17th February 2021. We encourage increased transparency in peer review by publishing the reviewer comments, author rebuttal letters and editorial decision letters if the authors agree. Such peer review material is made available as a supplementary peer review file. **Please state in the cover letter 'I wish to participate in transparent peer review' if you want to opt in, or 'I do not wish to participate in transparent peer review' if you don't.** Failure to state your preference will result in delays in accepting your manuscript for publication.

Please note: we allow redactions to authors' rebuttal and reviewer comments in the interest of confidentiality. If you are concerned about the release of confidential data, please let us know specifically what information you would like to have removed. Please note that we cannot incorporate redactions for any other reasons. Reviewer names will be published in the peer review files if the reviewer signed the comments to authors, or if reviewers explicitly agree to release their name. For more information, please refer to our FAQ page.

ORCID
IMPORTANT: Non-corresponding authors do not have to link their ORCIDs but are encouraged to do so. Please note that it will not be possible to add/modify ORCIDs at proof. Thus, please let your co-authors know that if they wish to have their ORCID added to the paper they must follow the procedure described in the following link prior to acceptance: https://www.springernature.com/gp/researchers/orcid/orcid-for-nature-research

Author names using non-Roman characters
Nature Portfolio journals can support presentation of author names using non-Roman characters in the HTML version of the article. If you wish to, please include author names in parentheses after the Roman-character spelling; see example online here. Currently supported scripts are: Arabic, Chinese, Cyrillic, Devanagari, Greek, Hebrew, Hangul, Japanese and Persian. You will be asked to verify the rendering is correct at proof stage.

Thank you again for your interest in Nature Methods. Please do not hesitate to contact me if you have any questions. We will be in touch again soon.

Sincerely,
Madhura

Madhura Mukhopadhyay, PhD
Senior Editor
Nature Methods

Reviewer #1 (Remarks to the Author):

The authors have appropriately addressed my previous comments, and I recommend acceptance of the paper.

Reviewer #2 (Remarks to the Author):

We thank the authors for their thoughtful responses to our comments.

A final point that the authors might consider commenting on in the Discussion is that expert assessment itself will have some level of subjectivity which may affect results (e.g. Figure 5C-D). Assessing inter-expert variation is beyond the scope of this study, but something to be aware of given the human-in-the-loop aspect of the work.

Minor: Figures 5D and S19 A-D y-axis label "Rank (lower is better)". Is lower better in terms of all the metrics shown (ARI, NMI, Smoothness Entropy, CHAOS)?

Reviewer #3 (Remarks to the Author):

I co-reviewed this manuscript with one of the reviewers who provided the listed reports. This is part of the Nature Methods initiative to facilitate training in peer review and to provide appropriate recognition for Early Career Researchers who co-review manuscripts.

Version 3:

Decision Letter:

17th Jul 2026

Dear Naveed,

I am pleased to inform you that your Article, "Beyond benchmarking: an expert-guided consensus approach to spatially aware clustering", has now been accepted for publication in Nature Methods. The received and accepted dates will be 19th June 2025 and 17th July 2026. This note is intended to let you know what to expect from us over the next month or so, and to let you know where to address any further questions.

Over the next few weeks, your paper will be copyedited to ensure that it conforms to Nature Methods style. Once your paper is typeset, you will receive an email with a link to choose the appropriate publishing options for your paper and our Author Services team will be in touch regarding any additional information that may be required. It is extremely important that you let us know now whether you will be difficult to contact over the next month. If this is the case, we ask that you send us the contact information (email, phone and fax) of someone who will be able to check the proofs and deal with any last-minute problems.

After the grant of rights is completed, you will receive a link to your electronic proof via email with a request to make any corrections within 48 hours. If, when you receive your proof, you cannot meet this deadline, please inform us at rjsproduction@springernature.com immediately.

Authors may need to take specific actions to achieve compliance with funder and institutional open access mandates. If your research is supported by a funder that requires immediate open access (e.g. according to Plan S principles or the NIH public access policy) then you should select the gold OA route, and we will direct you to the compliant route where possible. Because authors warrant under our subscription licensing terms that they haven’t committed to licensing any version of their article under a licence inconsistent with the terms of our agreement – including the applicable embargo period – publication under the subscription model isn’t suitable for authors whose funders require no embargo.

If you have any questions about our publishing options, costs, Open Access requirements, or our legal forms, please contact ASJournals@springernature.com

If you have posted a preprint on any preprint server, please ensure that the preprint details are updated with a publication reference, including the DOI and a URL to the published version of the article on the journal website.

You may wish to make your media relations office aware of your accepted publication, in case they consider it appropriate to organize some internal or external publicity. Once your paper has been scheduled you will receive an email confirming the publication details. This is normally 3-4 working days in advance of publication. If you need additional notice of the date and time of publication, please let the production team know when you receive the proof of your article to ensure there is sufficient time to coordinate. Further information on our embargo policies can be found here:
https://www.nature.com/authors/policies/embargo.html

To assist our authors in disseminating their research to the broader community, our SharedIt initiative provides you with a unique shareable link that will allow anyone (with or without a subscription) to read the published article. Recipients of the link with a subscription will also be able to download and print the PDF.

As soon as your article is published, you will receive an automated email with your shareable link.

If you are active on Twitter/X or Bluesky, please e-mail me your and your coauthors’ handles so that we may tag you when the paper is published.

You can now use a single sign-on for all your accounts, view the status of all your manuscript submissions and reviews, access usage statistics for your published articles and download a record of your refereeing activity for the Nature journals.

Please note that you and any of your coauthors will be able to order reprints and single copies of the issue containing your article through Nature Portfolio's reprint website, which is located at http://www.nature.com/reprints/author-reprints.html. If there are any questions about reprints please send an email to author-reprints@nature.com and someone will assist you.

Please feel free to contact me if you have questions about any of these points.

Best regards,
Madhura

Madhura Mukhopadhyay, PhD
Senior Editor
Nature Methods

** Visit the Springer Nature Editorial and Publishing website at www.springernature.com/editorial-and-publishing-jobs for more information about our career opportunities. If you have any questions please click here.**

Open Access This Peer Review File is licensed under a Creative Commons Attribution 4.0 International License, which permits use, sharing, adaptation, distribution and reproduction in any medium or format, as long as you give appropriate credit to the original author(s) and the source, provide a link to the Creative Commons license, and indicate if changes were made. In cases where reviewers are anonymous, credit should be given to 'Anonymous Referee' and the source.
The images or other third party material in this Peer Review File are included in the article's Creative Commons license, unless indicated otherwise in a credit line to the material. If material is not included in the article's Creative Commons license and your intended use is not permitted by statutory regulation or exceeds the permitted use, you will need to obtain permission directly from the copyright holder.
To view a copy of this license, visit https://creativecommons.org/licenses/by/4.0/

General response

We sincerely thank the editor and reviewers for their rapid and positive evaluation of our manuscript.

In response to the reviewers' remarks, we have now: (1) expanded our analysis and discussion to include metrics beyond the ARI, while emphasizing our reliance on ARI not to rank methods according to performance as commonly done, (2) expanded our scalability analysis of the entire consensus clustering pipeline, and (3) added detailed guidelines and practical considerations to support users with new biological applications.

Below, we provide our point-by-point responses. To facilitate the review, we have modified the comment numbering for clarity. Our responses are presented in blue text, and new text or figures added to the manuscript are highlighted in red.

We believe these revisions, which incorporate additional guidelines, comparative analyses, and new supplementary figures as requested, have further strengthened the study. We are grateful for the reviewers' thoughtful feedback and the opportunity to refine and improve our work.

Reviewer #1 (Remarks to the Author):

Sun et al. presents SACCELERATOR, a modular and extensible framework for evaluating spatially aware clustering methods in spatial transcriptomics. The authors argue that traditional benchmarks relying on fixed ground truth annotations are limited by subjectivity, granularity bias, and lack of scalability. To address these issues, they proposed a consensus-guided, expert-in-the-loop approach that integrates results from multiple spatial clustering methods to identify robust clustering patterns and highlight method disagreements through entropy-based spatial metrics. The framework is validated across many datasets, including case studies in brain and colorectal cancer tissues.

This is a timely and well-motivated contribution to the field. Benchmarking studies are common in spatial omics, but many rely on so-called ground truths that are often based on H&E images or prior biological assumptions. These annotations are prone to human error, granularity mismatch, and conceptual bias, and different benchmarking studies have led to contradictory conclusions.

This manuscript directly addresses those issues by showing, through extensive analyses across multiple datasets and platforms, that:
• Spatially aware clustering method performance is inconsistent across benchmarks;
• Ground truth annotations often penalize biologically meaningful patterns;
• A consensus approach provides a more robust, reproducible, and flexible foundation for evaluating spatially aware clustering results;
• An expert-in-the-loop workflow reflects real research practice more faithfully than static evaluations.
• The authors provide an open-source, modular platform that the community can build upon. This promotes transparency and reproducibility.

The consensus strategy is not algorithmically novel, but its application in this context is pragmatic and impactful. The authors also tackled scalability, modularity, and extensibility in a way that adds real value for the community.

Areas for improvement
1. Expert input is valuable but subjective; more structured documentation of expert feedback or annotation protocols would enhance reproducibility.

We agree that documentation and transparency of expert feedback and annotation protocols are critical for reproducibility and to guide users in their own use cases. While the annotation process was in sufficient detail for case study 1 on the MERFISH mouse thalamus dataset (e.g. regions were annotated based on registration to CCF v3.0), some details were missing for case study 2 on the VisiumHD colon cancer dataset. We address this point by including additional material to provide more structured documentation, and also by adding further details of how reference annotation was performed for the VisiumHD colon cancer dataset.

*While the nature of biological inquiry remains different for individual case studies, we were able to generalize the steps for running the consensus clustering and engaging with experts into a flow chart (**Supplementary Figure S21**), which we included as part of the online documentation for SACCELERATOR. Additionally, we have also provided users with an expert-in-the-loop pipeline checklist **(Supplementary File 1)**, which lists the questions and checkpoints for each step of the pipeline. While the specific analysis using our pipeline will vary according to the biological questions of interest, tissue types, and spatial technologies, we distill the rationale and logic of the pipeline into the checklist*

questionnaire and try to give instructions as specific as possible for each step. Given the varied nature of computational biology analysis, we hope this general guideline, along with the annotation protocol, can serve as a structured strategy on how to involve experts in different stages of the analysis, incorporating their feedback and ensuring biological and computational validity. This is further detailed in a new section titled "Practical guidelines for an expert-in-the-loop workflow".

New section: Practical guidelines for an expert-in-the-loop workflow

Bioinformatics analyses are inherently iterative and question-driven, and spatially aware clustering analyses are no exception. Their strong dependence on biological context is what challenges the idea of a one-size-fits-all benchmarking framework and motivates the development of the expert-in-the-loop workflow. While our workflow has demonstrated its utility across several case studies, its broader application inevitably depends on case-specific conditions and expert interpretation. To summarize this complexity, we compiled a concise guideline outlining key considerations and checkpoints for the workflow, including approaches for expert involvement, parameter selection, and ensuring reproducibility (**Supplementary File 1**). Rather than prescribing a standardized best practice, this guideline serves as a reference manual to promote thoughtful and transparent use of the workflow across diverse analysis settings.

Whether studies are hypothesis-driven or exploratory, expert knowledge offers critical guidance that helps prevent misinterpretations and analytical pitfalls. The scope and nature of involvement of tissue experts inevitably vary across projects. In the case studies, we demonstrate how experts can guide clustering granularity (**Figure 4E**), assess base clustering performance (**Figure 5C**), and interpret consensus-level biological features (**Figure 4F-H**). Beyond these specific instances, the guideline outlines additional scenarios for expert participation and the reasoning behind them. We also illustrate how they translate into actionable steps by mapping analysis decisions to guideline checkpoints, exemplified by a detailed breakdown of analysis logic for Case study 1 (**Supplementary Figure S21)**, which offers practical insights into the workflow's implementation.

New methods text section: Visium HD colorectal cancer histology section annotation

At the time of analysis, no ground truth annotation was available for the Visium HD human colorectal cancer dataset. After data download, annotation of the Visium HD colorectal cancer histology sections was performed on hematoxylin and eosin H&E-stained whole-slide images by a trained histologist. Regions of smooth muscle, vessels, normal epithelium, and neoplastic epithelium were manually annotated, followed by review by a pathologist. Smooth muscle was identified by its characteristic pink eosinophilic staining in organized bundles, with elongated, centrally located nuclei. Blood vessel regions were annotated based on the presence of luminal structures, filled with erythrocytes and lined by flat cells (endothelium). Normal epithelium was identified by recognizing well-organized crypt architecture, composed of columnar epithelial cells with basally oriented nuclei, low nuclear-to-cytoplasmic ratios, and regular cell polarity. Neoplastic epithelium was annotated by identifying regions with loss of normal crypt architecture, increased nuclear pleomorphism, hyperchromasia, irregular nuclear contours, aberrant nuclear-to-cytoplasmic ratios, and loss of polarity. Stroma or connective tissue was identified by its bright eosinophilic staining, low cellular density, presence of spindle-shaped fibroblast-like cells, and visible connective tissue fibers (e.g., collagen). Rare interspersed immune cells, such as neutrophils, lymphocytes and others, could occasionally be observed. Stroma was typically located adjacent to epithelial or neoplastic areas. Dense accumulation of lymphoid cells, including lymphocytes and histiocytes would represent lymphoid structures. However, such structures were not observed in the

present CRC case. Dissociated cell material indicative of detritus or necrosis was identified by the presence of cellular debris, often accompanied by infiltration of neutrophilic granulocytes.

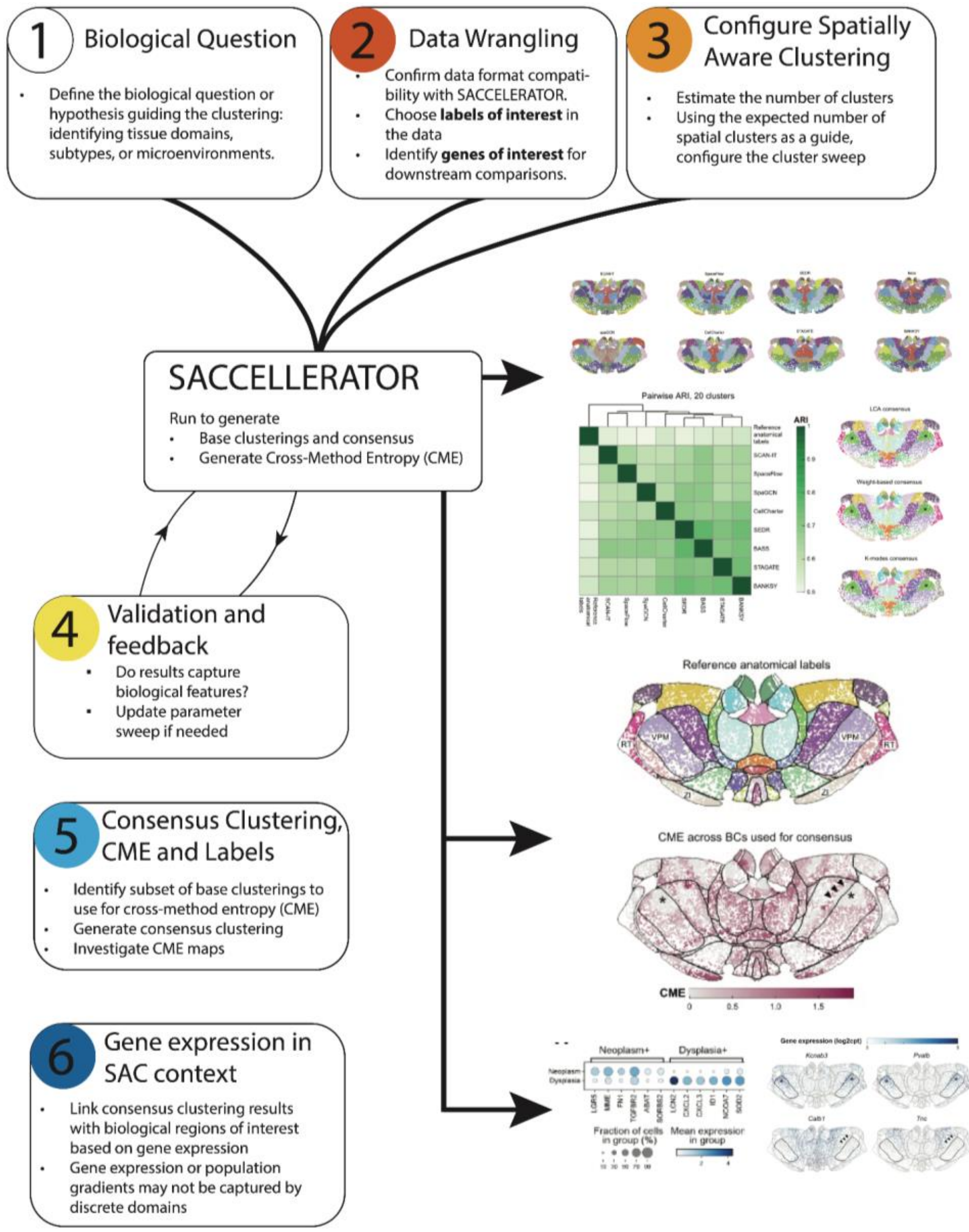

Supplementary Figure S21: *Schematic of the SACCELERATOR consensus guided expert in the loop framework as applied to analysis of the MERFISH mouse brain thalamus dataset (Case study 1). To address issues in identifying biologically relevant regions in spatial transcriptomics data, we adopt a flexible expert-in-the-loop consensus-driven approach. This goes beyond traditional ensemble/consensus methods, and allows researchers to interact with intermediate results to determine which tools should be used to generate a consensus (steps 1-3). Inclusion of an expert-in-the-loop is critical to ensure that the computational analysis matches the biological question at hand, and we believe that when the focus of the analysis is to uncover novel biological discoveries, tissue experts are accessible more often than not (steps 4-6). Figure uploaded to the GitHub landing page for SACCELERATOR:* *https://github.com/SpatialHackathon/SACCELERATOR/.*

**Biological Question**	• **Define the biological question** or hypothesis guiding the clustering: identifying tissue domains, subtypes, or microenvironments. For example:<br>○ Does spatial transcriptomics support subdividing anatomical regions of the thalamus?<br>○ Can spatial transcriptomics identify neoplastic progression stages in colorectal cancer?
**Data Wrangling**	• Confirm **data format compatibility** with SACCELERATOR.<br>• Identify **labels of interest** already in the data: manual annotations and cell type labels can be used for comparison and validation.<br>• Identify **genes of interest** for downstream comparisons.
**Configure Spatially Aware Clustering**	• **Estimate** how many spatial clusters are present in the tissue<br>• Using the **expected number of spatial clusters** as a guide, configure the cluster sweep to span at least ±5 numbers of clusters around the expected number.<br>• **Run** all, or selected methods suitable for the data. By default the downstream consensus workflow will select the 8 most concordant methods based on cross-method ARI. This will generate the base clusterings
**Validation and Feedback**	• **Review SACCELERATOR outputs**: and compare to biologically relevant structures.<br>○ Validate scale and number of clusters in the context of the biological topic.<br>○ Does one base clustering result address biological questions?<br>○ For example, in the brain: do some outputs include laminar organization of cortex? In cancer cases, do some outputs correctly identify tumor tissue?<br>• If biological features are not included, **review SACCELLERATOR configuration** and broaden the parameter sweep.
**Consensus Clustering, CME and Reference Labels**	• If needed, identify the **subset of base clusterings** to use for cross-method entropy (CME), e.g., filtering base clusterings based on spatial continuity measures (such as smoothness entropy).<br>• Generate **visual summaries** (spatial consensus plot, cross-method entropy map, weight distribution plot, pairwise ARI heatmaps with clustering method class) to accompany expert interpretation.<br>• Using CME heatmap and consensus, check if any single base **clustering method/class dominates the consensus**.<br>• Verify that spatial resolution and granularity of the **final consensus** includes the biological scales of interest.<br>• **Interpretation of CME maps:**<br>○ Identify low entropy areas, where labels are consistent across methods.<br>○ Areas of high entropy often correspond to boundaries between regions where multiple methods give slightly different results.
**Gene Expression in SAC context**	• **Review gene expression** in the context of consensus clustering.<br>○ Link **consensus clustering results with biological regions of interest** based on gene expression.<br>○ Gene expression or population **gradients may not be captured by discrete domains**.<br>○ Do genes of interest show differential expression between consensus clusters?
**Important Notes**	• SACCELLERATOR assumes cellular **quality control** has already been performed.<br>• Even within a single tissue sample, genes of interest, relevant length scales, and expected numbers of clusters can **vary based on biological questions**. Independent runs of SACCELERATOR may be required to address different biological questions.<br>• We recommend a discussion to **review interpretations** of the consensus clusterings, including, for example: choice of a single consensus output or application of CME to identify domain boundaries.

***Supplementary File 1**: SACCELERATOR checklist.*

2. Consensus method scalability could be further quantified, especially as datasets reach millions of cells.

*We appreciate the reviewer's emphasis on scalability as spatial transcriptomics datasets grow ever larger. To address this, we have now systematically benchmarked 3 consensus clustering strategies (Latent Class Analysis (LCA), K-modes, and weighted-based consensus) on multiple datasets spanning from 3,798 spots to 142,991 cells across different technologies and tissue sections, quantifying both runtime and memory usage in a new **Supplementary Figure S23**. We quantified how both sample size (in terms of the number of spatial observations, such as cells and spots) and the number of base-clustering inputs affect the runtime and memory usage of the consensus algorithms. These new results demonstrate that while consensus clustering generally remains tractable up to the medium-sized datasets tested, algorithmic choice has measurable effects, with K-modes offering particularly robust scaling for very large dataset inputs. Both K-modes and weighted consensus are robust in terms of memory use with respect to the number of input base-clusterings, whereas LCA has an increasing memory demand correlating to the number of input base-clusterings, albeit always with lower median memory usage than weighted consensus in the datasets tests. We summarize these findings in the Results and Discussion sections.*

*Importantly, as the reviewer points out (and as shown in our original **Supplementary Figure S3**), the consensus-building step should be considered in the context of total computational requirements, since all contributing SAC methods must be run first. We now explicitly clarify this cumulative computational burden in the revised manuscript in both text and figure legends.*

Added text to the new "Practical guidelines for an expert-in-the-loop workflow" section: The choice of consensus algorithm should be tailored to the specific research question, taking into account tissue type, spatial technology, and the underlying biological hypothesis. In this study, our recommendations are primarily guided by scalability considerations (**Supplementary Figure S23**). We assessed runtime and memory usage for the three implemented consensus algorithms (K-modes, LCA, and weighted consensus) across a range of dataset sizes (i.e., number of cells or bins) and the number of input base clusterings. Overall, resource requirements increase with dataset size for all three methods. K-modes scaled best to large datasets, followed by LCA and weighted consensus. When increasing the number of base clusterings, all methods showed a trend of marginally increased runtime; however, LCA showed a stronger tendency toward higher memory consumption with increasing numbers of base clusterings. Across both variables, K-modes was the most resource-efficient, Weighted consensus was the most resource-intensive, and LCA showed intermediate performance.

Added Discussion text: In addition to the scalability of the individual methods with the number of cells and genes, the scalability of our proposed consensus-based clustering is also affected by the number of included base clusterings. Our results indicated K-modes as the most resource-efficient approach followed by LCA and finally the weighted-consensus. In addition to the number of base clusterings included, users should also consider the diversity of the selected methods in terms of their underlying algorithms. Since we envision the use of consensus clustering within an exploratory analysis without clear prior knowledge on the expected tissue clusters, aiming for a sufficiently diverse set of base clustering is desirable.

Added text to "Consensus clustering algorithms" methods section: The cluster scalability results were generated using the benchmark feature of Snakemake. The consensus part of the pipeline was applied to all existing clustering results of datasets with the following names: xenium-mouse-brain-SergioSalas, abc_atlas_wmb_thalamus, libd_dlpfc, visium_hd_cancer_colon, cosmx_lung,

STARmap_plus, visium_breast_cancer_SEDR. Results of those consensus results can be found on Zenodo (Data and Code Availability).

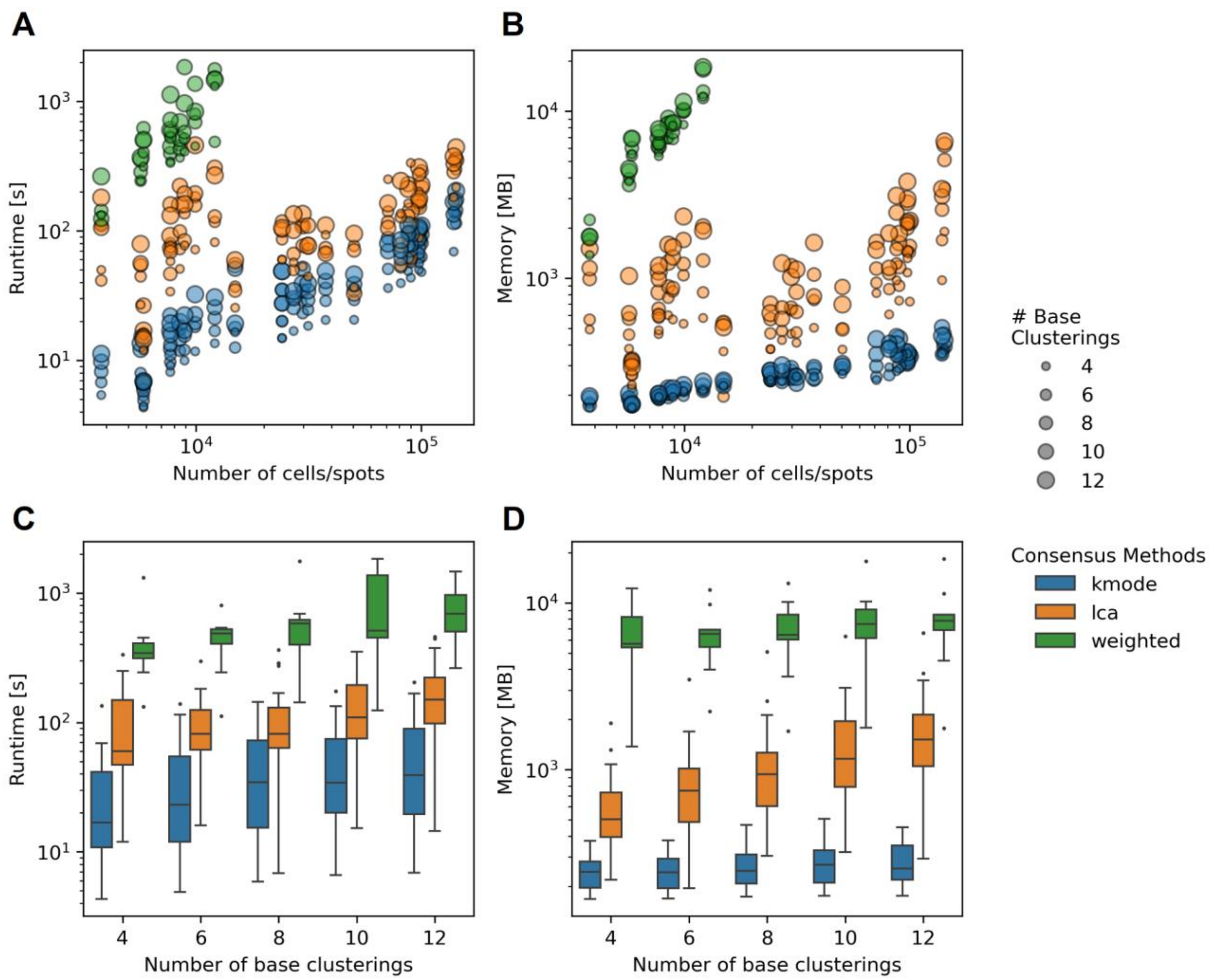

***New Supplementary Figure S23: Scalability analysis of consensus algorithms**. **a,b,** consensus algorithm runtime (a) and memory (b) w.r.t number of cells and spots in the dataset; **c,d,** consensus algorithm runtime (c) and memory (d) w.r.t number of base clustering input.*

3. A more systematic exploration of clustering granularity and its biological interpretability would strengthen the guidance for users.

We thank the reviewer for highlighting the need for a systematic exploration of clustering granularity and its biological interpretability. In most cases, we have run the algorithms across a sweep of cluster numbers. However, since it may require some input from tissue experts, we feel that it is difficult to give clear prescriptive guidance to users, other than to bring additional awareness to the problem. Nonetheless, given our access to neuroanatomy experts, we now include a detailed sweep of cluster numbers for the mouse brain. Previously, we explored how clustering granularity changed in the isocortex region as we reduced the number of target clusters from 11 (as defined in the reference annotation), where the isocortex was over-segmented compared to the reference annotation, down to 5 clusters. We

have revised this analysis to align with the reviewer request and now cover a range from 5 to 49 using CellCharter.

*At the low end of the parameter sweep (**Supplementary Figure S24A shows 5 and 9 clusters**), anatomically distinct regions are merged into single clusters, including grey matter and white matter and the thalamus and hypothalamus. Additionally, cortical layers are not separated cleanly. In this regime, clusters are of limited utility for downstream analysis or annotations. In the intermediate range targeted by our sweep (**Supplementary Figure S24B shows 11 and 18 clusters**), clusters reflect finer anatomical divisions, including distinct clusters for CA subfields and dentate gyrus in the hippocampus and, at 18 clusters, a distinct cluster for Layer 4 in the cortex and separate clusters for thalamus, zona incerta and hypothalamus. This regime is detailed, while still achieving spatially segregated clusters. At high numbers of clusters (**Supplementary Figure S24C shows 24 and 31 clusters**), clusters begin to become intermixed, reflecting cellular diversity within anatomical domains. At very high numbers of clusters, the clusters reflect a combination of discrete cell identities and their local neighborhoods. This high level of resolution is not useful in the context of annotating anatomical domains or associating clusters with discrete brain structures.*

*These findings offer practical guidance for users: examining how the derived cluster structure "tracks" the known anatomical hierarchy can be a valuable strategy for selecting biologically meaningful clustering resolutions. Furthermore, we have mentioned the issue of "scale" (clustering granularity) again in the flowchart Supplementary Note, we have amplified the message in the text around **Figure 2**; it is discussed again in the context of **Figure 3** (Visium DLPFC) where we search for a "sweet spot" of number of methods and number of clusters (5, 7, and 9 clusters were used), and again in **Figure 4** (MERFISH of adult mouse thalamus regions) where we explored 16, 20, and 24 clusters.*

Added text to the new "Practical guidelines for an expert-in-the-loop workflow" section: A critical, yet often underexplored, aspect of SAC is the investigation of the granularity of tissue features, analogous to the granularity considerations commonly addressed in cell typing for single-cell transcriptomics. We further examined the clustering granularity in the Xenium mouse brain dataset (**Supplementary Figure S24**). Although a broad range of cluster numbers corresponded well with established anatomical segmentations, clusterings with very low numbers were insufficient to resolve larger, coarse anatomical structures and very high cluster numbers tended to capture cellular heterogeneity rather than spatially contiguous anatomical domains. This finding highlights the importance of selecting an appropriate clustering resolution that balances anatomical interpretability with cellular detail when analyzing spatial transcriptomic data.

Changed text in the Discussion: ~~Although we have not exhaustively investigated all possible clustering granularities, our framework allows researchers to investigate spatial structures at multiple scales.~~ Using our framework, we investigated a wide range of granularity levels in the Xenium mouse brain dataset. Our results indicate that for a range of clusterings, there is good correspondence with the expected anatomical divisions, while low numbers were insufficient to resolve larger structures and high numbers capturing cellular heterogeneity instead of anatomical domains.

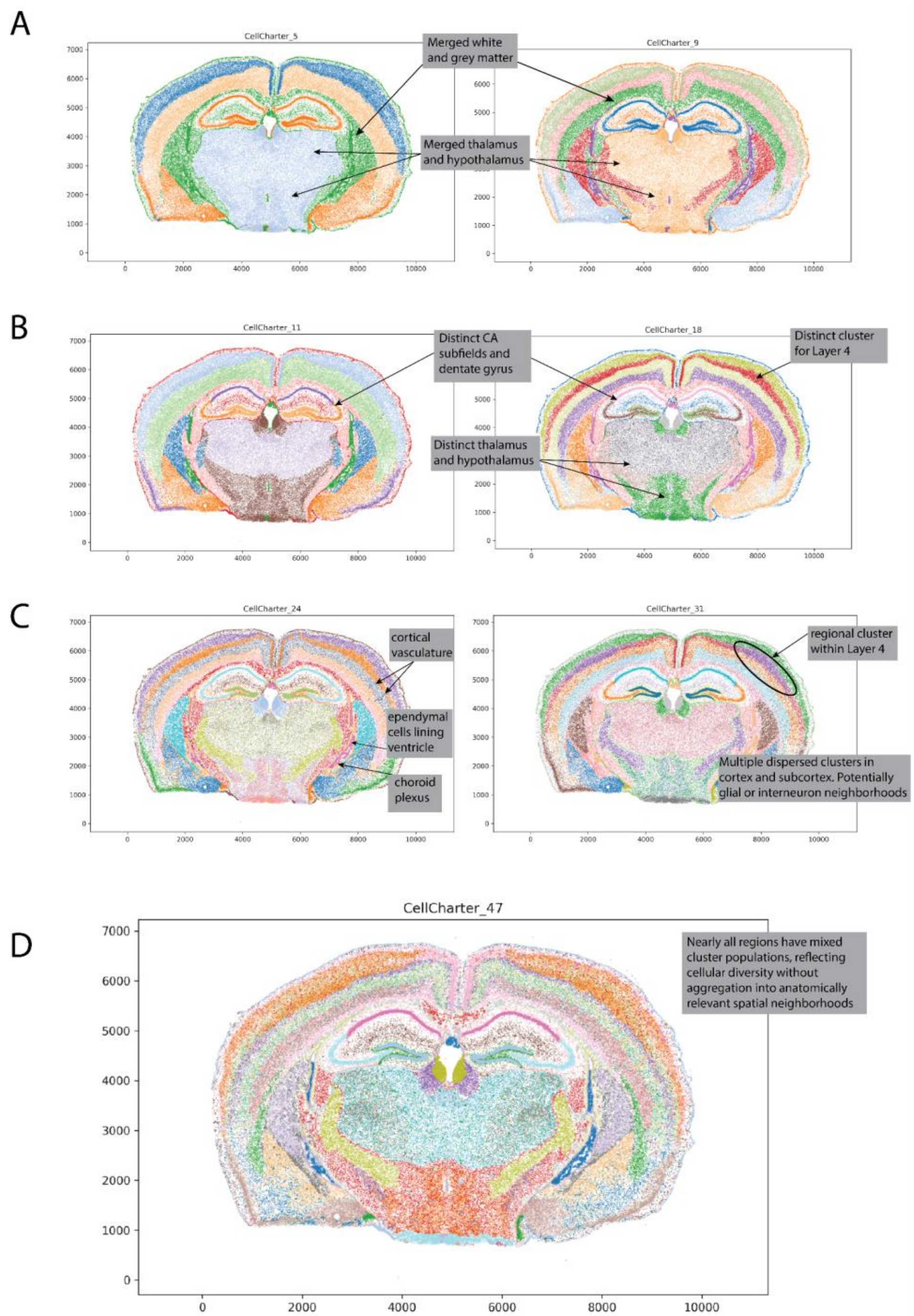

New Supplementary Figure S24: SAC cluster sweep encompasses high- and low-resolution clustering results. In this annotated example using CellCharter on the Xenium mouse brain data, we covered a wide range of cluster parameters to illustrate how spatial resolution is related to cluster number. Subplot titles indicate the number of clusters. A. Low numbers of clusters fail to resolve even coarse anatomical regions. B. Moderate cluster numbers include highly resolved anatomical structures, including cortical layers, structures within the hippocampus and distinct domains for thalamus and hypothalamus. C. High cluster numbers showing further resolution, which may be desirable for some studies, but some anatomical regions begin to include multiple clusters built on distinct cellular populations. D. Very high cluster number showing salt-and-pepper distributions of clusters across the brain, reflecting cellular diversity rather than contiguous spatial domains.

Added text to the "GT granularity analysis" (Method) **section:** The granularity scanning for the Xenium Mouse Brain Dataset in ***Supplementary Figure S24*** is conducted using the default configuration of CellCharter in the workflow while setting cluster numbers from 5 to 49. Results of the cluster sweeping have been uploaded to Zenodo (Data and code availability), and additional visualization of the sweeping results can be found in **Supplementary File 2**.

4. Some reliance on ARI remains; alternatives like biological validation or application-driven metrics could further support conclusions.

We thank the reviewer for raising the point regarding the use of the Adjusted Rand Index (ARI). Please also refer to the response to reviewer 2 comment 1, where we further investigate the relatedness of other general-purpose and application-specific metrics. We would like to clarify that while ARI is reported in our work, it is not intended as a critical or exclusive validation metric. Its use is explicitly described as a familiar reference for readers and to compare clustering assignments between computational approaches, not as the primary measure of performance or biological relevance. The main text and figure legends make clear that ARI is not used as a proxy for biological accuracy or discovery impact. Rather, the ARI is reported alongside other, more contextually relevant metrics. To ensure the clarity of our message and avoid misunderstanding of our usage of ARI, we have also added additional sentences in the Results and Discussion sessions to emphasize the role of metrics in our workflow.

Our primary focus is on metrics and validation strategies that are uniquely appropriate for spatial transcriptomics (thus application-driven metrics). We employ smoothness entropy (SE) as an application-driven metric that directly measures spatial continuity and coherence, quantifying how well predicted domains align with known or plausible tissue architecture. The SE is a major criterion for selecting the optimal set of results for consensus clustering in case study 1 ***(Supplementary Figure S22*** *shows the ranking of SE for different base clustering instances****)****. Additionally, the cross-method entropy (CME), which is central to the SACCELERATOR framework, quantifies biologically meaningful consistency across model-specific outputs and requires domain knowledge and adaptation to the biological context at hand.*

Beyond these computational metrics, expert and biological validation form a cornerstone of our study. For both presented case studies, clustering assignments were carefully annotated and interpreted in collaboration with tissue experts. The mouse brain thalamus analysis was led by Dr Meghan Turner and Dr Brian Long from the Allen Institute for Brain Science, who identified a biologically supported split in the ventral posteromedial nucleus (VPM) through differential gene expression of Pvalb, Tnc, Calb1, and Kcnab3. These findings were consistent with independent work by Rubio-Teves et al. (https://www.jneurosci.org/content/44/43/e1096242024), where these distinct regions within the VPM were found to correspond to different axonal projections. Similarly, the VisiumHD colorectal cancer analysis was led by histologists and pathologist Dr Amin El-Heliebi and Dr Martin Zacharias from the Medical University of Graz. In this case study, we explicitly demonstrate that ARI does not associate with performance in recognizing clinically relevant regions. Instead, we proceed with generating consensus clustering based on the preferred output of SEDR (based on expert evaluation rather than ARI), where our approach successfully separated neoplastic and dysplastic areas and linked these to genes implicating a transition from inflammation (CXCL2, CXCL3) and stress (SOD2, NCOA7) to aggressive and pro-invasive states (e.g., LGR5, TGFB2).

While we report ARI for completeness and comparability with other studies in the field, our study emphasizes and is rigorously supported by biologically and application-motivated evaluation criteria. Our conclusions rest on the convergence of spatial coherence metrics, cross-method consistency measures, and expert-guided biological validation. We believe this multi-faceted approach provides robust support for the plausibility and biological relevance of our results, and we hope this clarifies the rationale for our metric selection strategy.

Added text to the new "Practical guidelines for an expert-in-the-loop workflow" section: In our case studies, base clusterings were chosen based on concordance blocks identified within the cross-method ARI heatmap (**Figure 3B**), as well as rankings of application-specific metrics that evaluate the spatial smoothness of clustering results, such as the smoothness entropy (SE, **Supplementary Figure S22**). Given the diversity of metrics included in the workflow, we encourage users to adapt these selection strategies to whichever metrics most effectively capture the biological phenomena of interest.

Added text to the Discussion: In our work, we rely on several metrics to compare clustering results between different methods. And while we rely on similar metrics to those reported in other studies (e.g. ARI), these are not intended as a critical or exclusive validation metric. Their use is explicitly described as a familiar reference for readers and to compare clustering assignments between computational approaches, not as the primary measure of performance or biological relevance. Our analysis of metric-metric correlation across various datasets revealed that inter-metric relationships are generally similar, with general-purpose metrics (ARI, NMI) clustering separately from application-specific spatial metrics (CHAOS, PAS), and internal validation metrics (DBI, Calinski-Harabasz) also forming distinct clusters based on their focus on cluster compactness and separation. Overall, ARI and SE were found to be generally representative of the other metrics in most scenarios.

5. While the core innovation is not on the methods, this is a thoughtful and impactful study that moves the field beyond the limitations of rigid benchmarking toward a more practical and biologically grounded evaluation paradigm. The authors' analyses are rigorous and comprehensive, and the framework is well-engineered, and the case studies are compelling. I found this work much better than those pure benchmarking papers previously published in Nature Methods, and I believe it merits publication with minor revisions.

We thank the reviewer for their positive remark. Indeed, we believe that it is important to highlight the limitations of current benchmarking practices where sufficient work has not been done in establishing reference datasets and evaluation strategies that are suitable for the task.

Reviewer #2 (Remarks to the Author):

Summary of key results

The authors introduce SACCELERATOR, an open-source consensus framework that integrates 22 spatially-aware clustering methods across 15 spatial-omics datasets. The main claims are: (i) current benchmarks are biased toward Visium/brain data and unreliable anatomical "ground-truth" labels; (ii) algorithm performance is highly context-dependent; and (iii) an expert-in-the-loop consensus strategy improves reproducibility and biological interpretability.

Originality & significance

Moving from static benchmarking to a dynamic, expert-guided consensus platform is highly original. No previous study has systematically examined method generalizability across healthy vs. cancer tissues while providing a practical feedback mechanism.

Data & methodology

1. Single-metric focus

1a. I agree with the point of traditional evaluation metrics do not always reflect result quality and it is good to see the authors introduce both traditional and spatially aware metrics. However, all main figures use Adjusted Rand Index (ARI) only, despite claims of evaluating 17 metrics.

We recognize that we indeed focused a bit too heavily on the Adjusted Rand Index (ARI). This point was also raised by reviewer 1 in comment 4, where we further detail how the use of ARI is not used to rank methods' performance but rather as a familiar reference to readers for comparing clustering assignment, and that the majority of results also utilise application-driven metrics and biological validation in the literature for support.

After careful reflection, we believe that the implementation of a large number of metrics is no longer central to our story, but rather a remnant of when our work endeavored to be a benchmark study. The core of SACCELERATOR is built around expert-guided consensus-driven analysis, to which the spatial clustering methods are core. As such, we believe an extensive evaluation of what these evaluation metrics mean, while interesting, is out of the scope of this study. We believe that toning down the emphasis may prevent misinterpretation that the large number of evaluation metrics is central to our study.

***Revised text**: The method outputs were evaluated using ~~17 different~~ **multiple** metrics that used the GT labels and spatial information to varying extents*

However, we appreciate that some level of systematic evaluation of the metrics would be useful, so we have now endeavored to include further evaluations for other metrics as suggested.

1b. Please add a supplementary heat-map or radar plot that includes NMI, SpatialARI, CHAOS and two stability scores to substantiate metric breadth.

We have expanded on the reporting of metric-metric correlation to additional datasets (new ***Supplementary Figure S1****). We observe that in most cases, the inter-metric relationships are highly similar. In particular, we notice that general-purpose metrics (e.g., ARI, NMI) cluster distinctly from application-specific metrics that evaluate spatial contiguity (e.g., CHAOS and PAS), with the only exception being the Jaccard Index. We also noticed the Davies-Bouldin Index (DBI) to cluster separately, which may reflect that this metric evaluates clustering performance based on cluster compactness (intra-cluster similarity) and separation (inter-cluster similarity). Similarly, the Calinski–Harabasz index clusters separately, possibly due to its nature as an internal validation metric based on cluster compactness and separation without reference to ground truth labels (used by e.g. ARI) or spatial relationships (e.g. as considered by CHAOS).*

Furthermore, we supplement the cross-metric similarity heatmaps with radar plots of the NMI, ARI, CHAOS and Smoothness Entropy (new ***Supplementary Figure S2****). Across nearly all datasets, we saw that methods scoring highly for any one given metric also scored highly for the others. In very few cases we saw methods with better ARI or NMI corresponding to worse CHAOS and smoothness entropy, particularly prominent in the Stereo-seq liver dataset (****Supplementary Figure 2, S28****). Tissue organization strongly varies between organs. The liver is an example of an organ with low contiguity and high periodicity. Methods achieving high ARI for this dataset like DR-SC better capture the repeating zonation pattern. A high CHAOS represents intermixed clusters, where cells of the same clusters are not segregated but spread out throughout the tissue. This is the natural state of the liver. CHAOS rewards methods like SCAN-IT, which merges separate biological zones into larger, spatially continuous segments. Classifying the entire central-to-portal tract as one cluster increases spatial smoothness and yields better CHAOS scores despite not matching the boundaries of the reference annotation.*

Together, this suggests that the ARI and smoothness entropy are generally representative of the other metrics in most situations. Despite this observation, it would be important to guide users to investigate these metrics.

Revised text (Results section)*:* In the workflow, we have implemented both accuracy-based metrics, such as ARI and NMI, and spatial continuity-based metrics, such as SE and CHAOS. Across different datasets, the two groups of metrics show general concordance among themselves in terms of correlation and ranking (**Supplementary Figure S1** to **S2**).

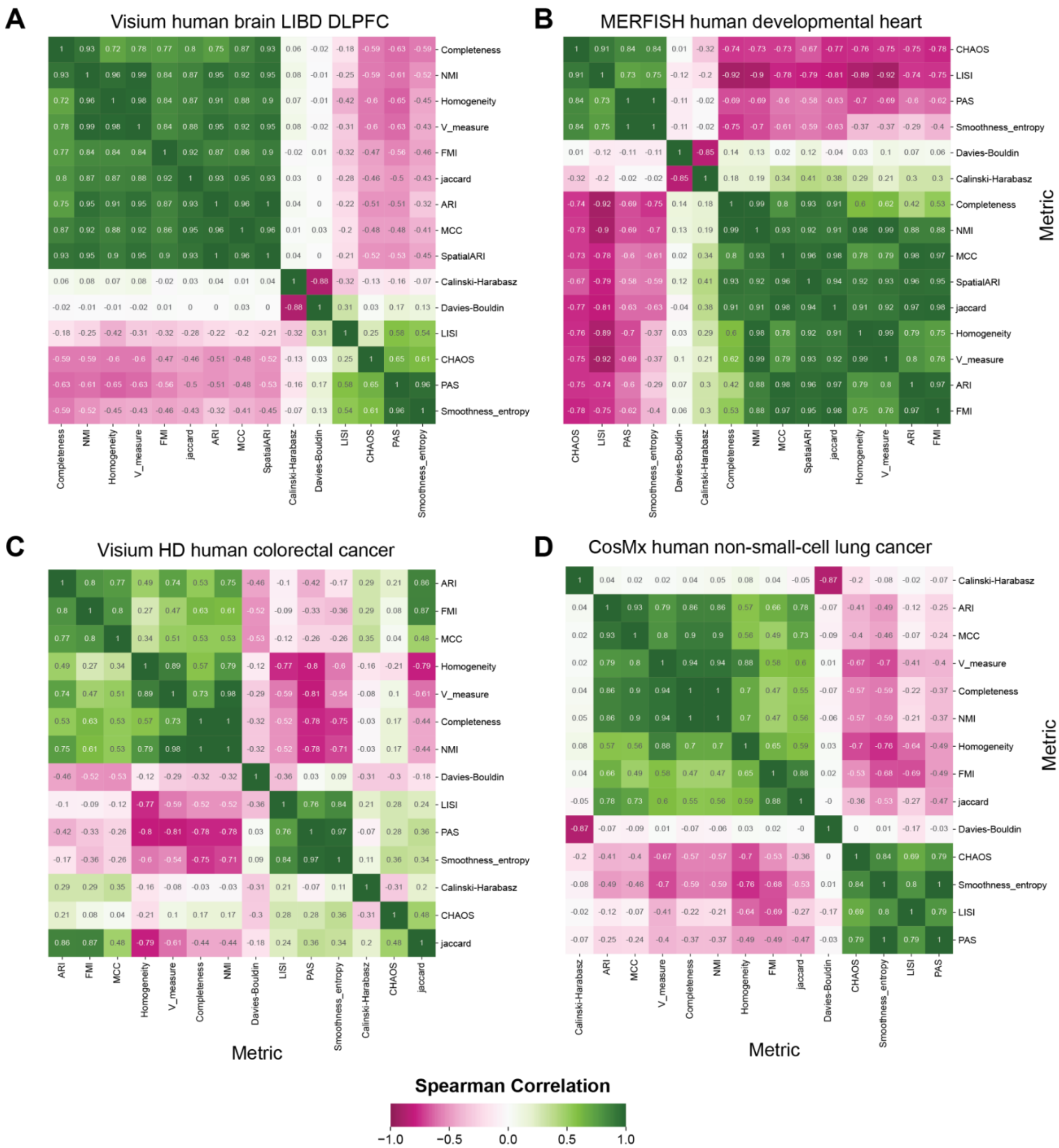

Revised Supplementary Figure S1: Spearman correlation matrix of different metrics across all methods and samples from **A**. LIBD DLPFC, **B**. MERFISH human developmental heart, **C**. Visium HD human colorectal cancer, **D**. CosMx lung.

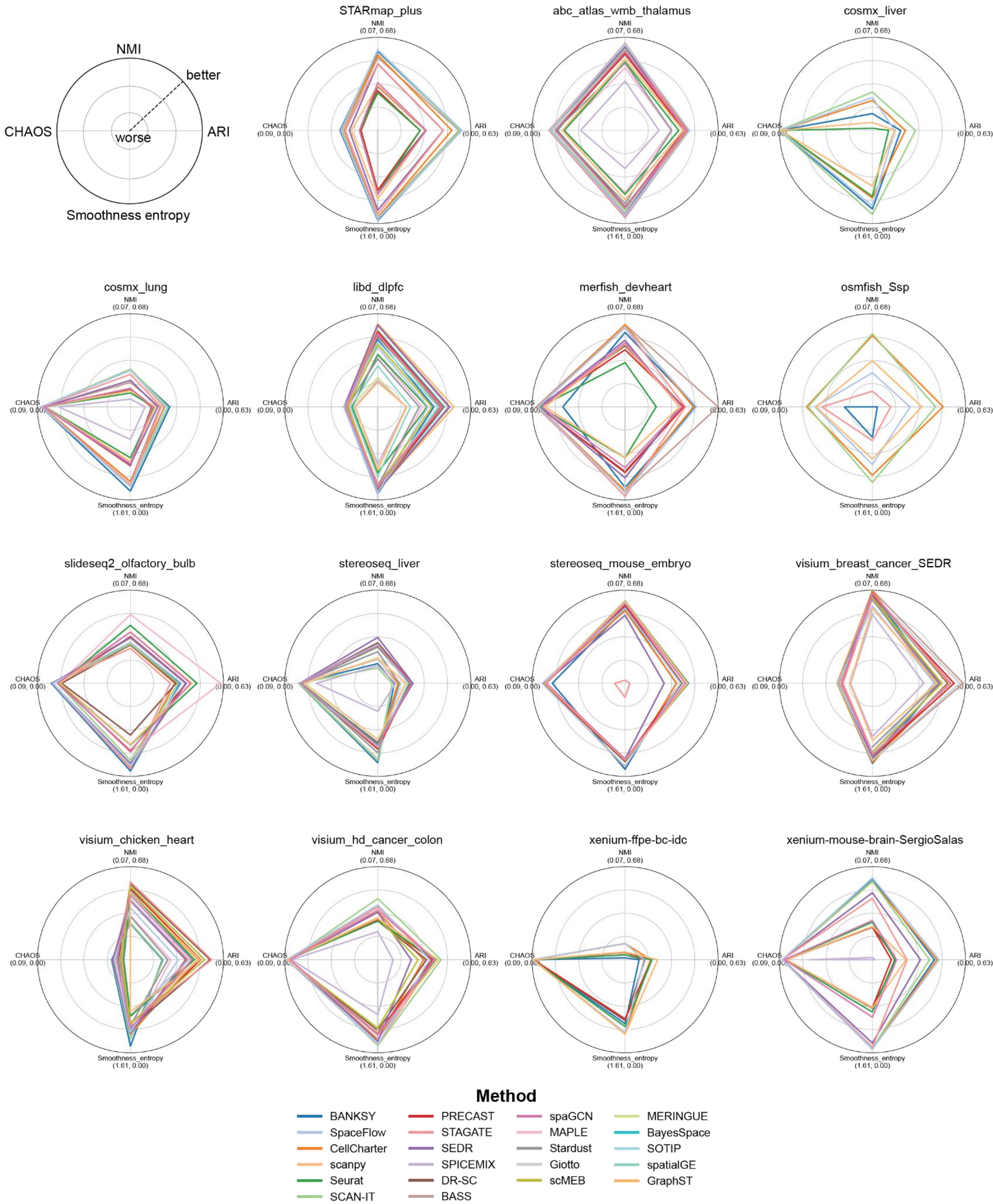

New Supplementary Figure S2: Radar plot of metrics scores over all datasets. Metrics shown are ARI, NMI, Smoothness Entropy, and CHAOS. Smoothness Entropy and Chaos scores are inverted so that the outer range of the radar plot corresponds to better scores.

1c. It would also be interesting to see how other metrics agree with the expert pathology ranking in **Figure 5**. I encourage more detailed discussion regarding the 17 metrics and how they agree with the ground truth as well as clustering results.

We extend the evaluated metrics versus the expert ranking for the colorectal cancer VisiumHD dataset presented in case study 2. Here, we expanded the comparison to also include the NMI, Smoothness Entropy, and CHAOS scores, which were shown to be representative of the other metrics. All metrics showed no clear association with the expert ranking, similar to what we observed with the ARI. As such, we have updated the description of this lack of concordance of expert vs metric evaluation.

Revised text*: This lack of concordance between expert versus metric evaluation was also evident when looking into the NMI, Smoothness Entropy, and CHAOS scores (****Supplementary Figure S19****).*

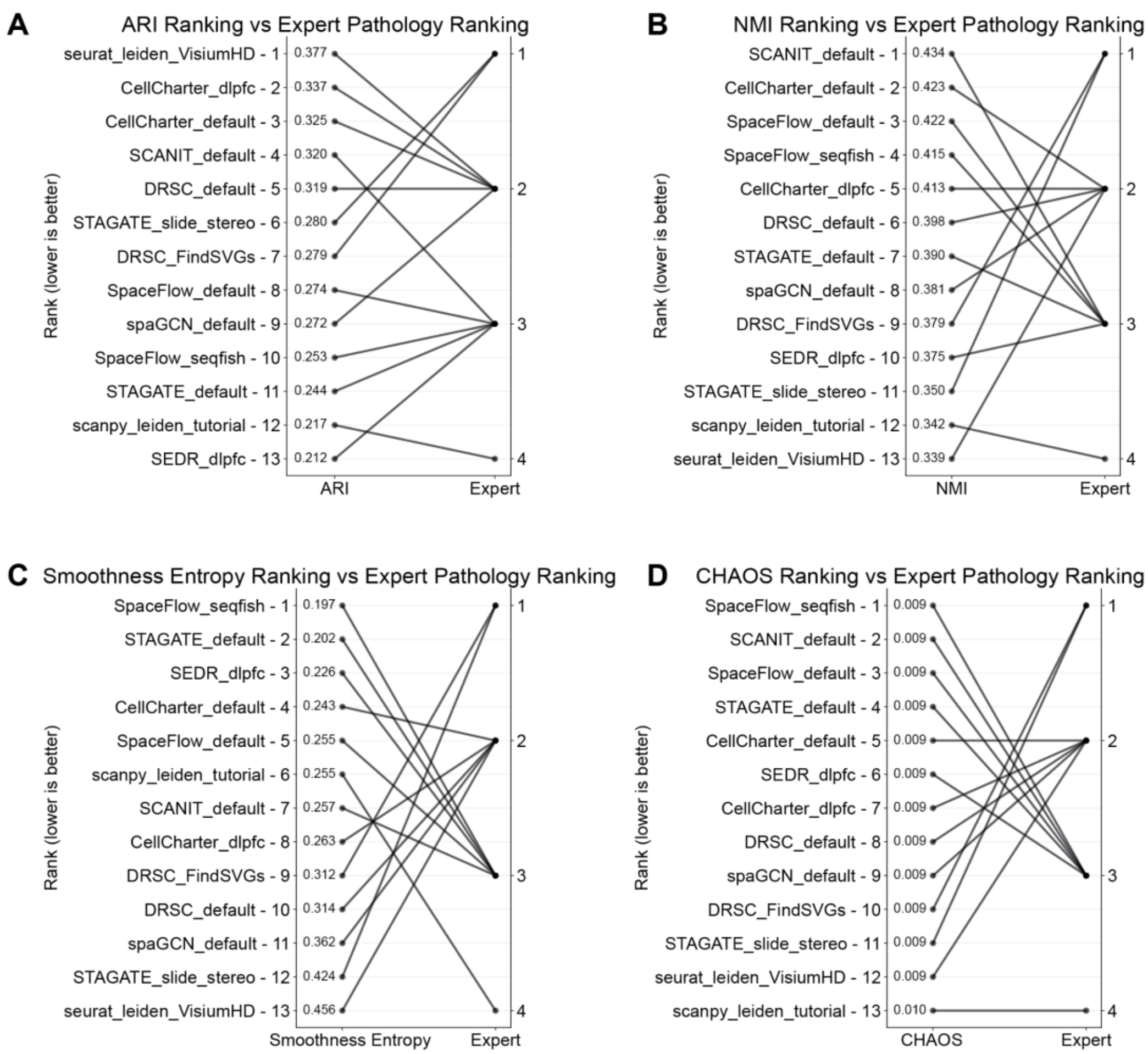

New Supplementary Figure S19: Comparison of expert rankings against evaluation metrics for the VisiumHD colorectal cancer sample. Rankings according to (A) the ARI, (B) NMI, (C) Smoothness entropy, (D) CHAOS vs the expert rankings.

1d. In supplementary figure 2, SpatialARI has a correlation of 1 with ARI – is this expected? Could you extend the correlation matrix to all the datasets to see if the similarities and differences in metrics are consistent?

Here, it is important to note that the ARI exhibited high levels of correlation to other metrics as well, including the MCC (0.96). In this case, the Spearman correlation of 1 was correctly reported. However, the Pearson correlation was 0.999458. We believe the convergence of these metrics for the DLPFC dataset is due to the reduced spatial complexity of the Visium dataset.

*We further investigated the correlation of ARI with spatialARI in other datasets (**Revised Supplementary Figure S1, previously S2**). We did not observe a Spearman correlation of 1.0 for other datasets, e.g., in the MERFISH developmental heart dataset, the Spearman correlation was 0.96. We were unable to calculate the spatialARI for all datasets due to high computational requirements. However, we believe that we have addressed the concern that the ARI and spatialARI are usually correlated, but not always 1.0. As reported in an earlier response, we observe that in most cases, the inter-metric relationships are similar between datasets.*

2. CME calculation and algorithmic similarity

The Consensus Entropy Metric (CME) is computed on "concordant" partitions pre-selected by high pairwise-ARI and low smoothness-entropy. This risks pooling methods with similar modelling assumptions, so low CME could reflect algorithmic redundancy rather than biological certainty. Analysis that might help answer this:
a) Algorithm-family stratification: group methods (graph-convolution, Bayesian, matrix-factorization, etc.), keep ≤2 representatives per family, and recompute CME.
b) External perturbation test: randomly erase 20 % of Layer 4 labels in slice 151673 and compare CME at perturbed vs. intact boundaries by permutation testing.
c) Intra- vs. inter-family ARI distribution to demonstrate that the concordant set is not dominated by a single family.

We thank the reviewer for this comment and agree that it may be possible for certain algorithm classes to identify similar patterns, thus there is an added risk of locking in those common patterns. However, if we look at the mix of algorithms from our results, we often observe multiple algorithms and multiple classes of clustering methods included in the base clusterings used for consensus.

*At the beginning of the project we grouped methods, classifying them as embedding+clustering, deep learning, and Bayesian/HMRF approaches, (**Reviewer-only Table 1**). However, since the classification is not always straightforward and sometimes ambiguous (e.g. GraphST is a deep learning method but also fits the embedding+clustering category), we decided not to include it in the manuscript.*

Reviewer-only Table 1: categorization of methods to algorithmic classes.

Embedding + clustering	Deep learning	Bayesian / HMRF
Banksy	SpaGCN	BayesSpace
Stardust	SEDR	PRECAST
spatialGE	SpaceFlow	scMEB
Meringue	SOTIP	DR-SC
SCAN-IT	GraphST	SPICEMIX
CellCharter	STAGATE	maple
		BASS
		Giotto

*If we recreate **Figure 3** (consensus clustering of results of the Slice 151673 of the DLFPC dataset) with row annotation that shows algorithms and classes (see **Reviewer-only Figure 1** below), we notice that there are methods that were exclusively in the concordant block used for the consensus that belonged to the three classes: embedding and clustering (SCAN-IT, CellCharter), deep learning (GraphST, SpaGCN), and Bayesian/HMRF (bass, precast, BayesSpace). Furthermore, we found that some methods were completely excluded from the consensus clustering that belonged to embedding and clustering (Stardust, Meringue, spatialGE), deep learning (SpaceFlow), and Bayesian/HMRF (maple, spicemix), as well as non-spatial methods (scanpy, seurat). Some methods only had some runs contributing to the consensus that belonged to embedding and clustering (Banksy), deep learning (SEDR, SOTIP, STAGATE), and Bayesian/HMRF (DR-SC) classes of algorithms. Altogether, there seems to be a spread of all method classes that contribute entirely, partially, or were completely excluded from the census calling of the DLPFC Visium dataset.*

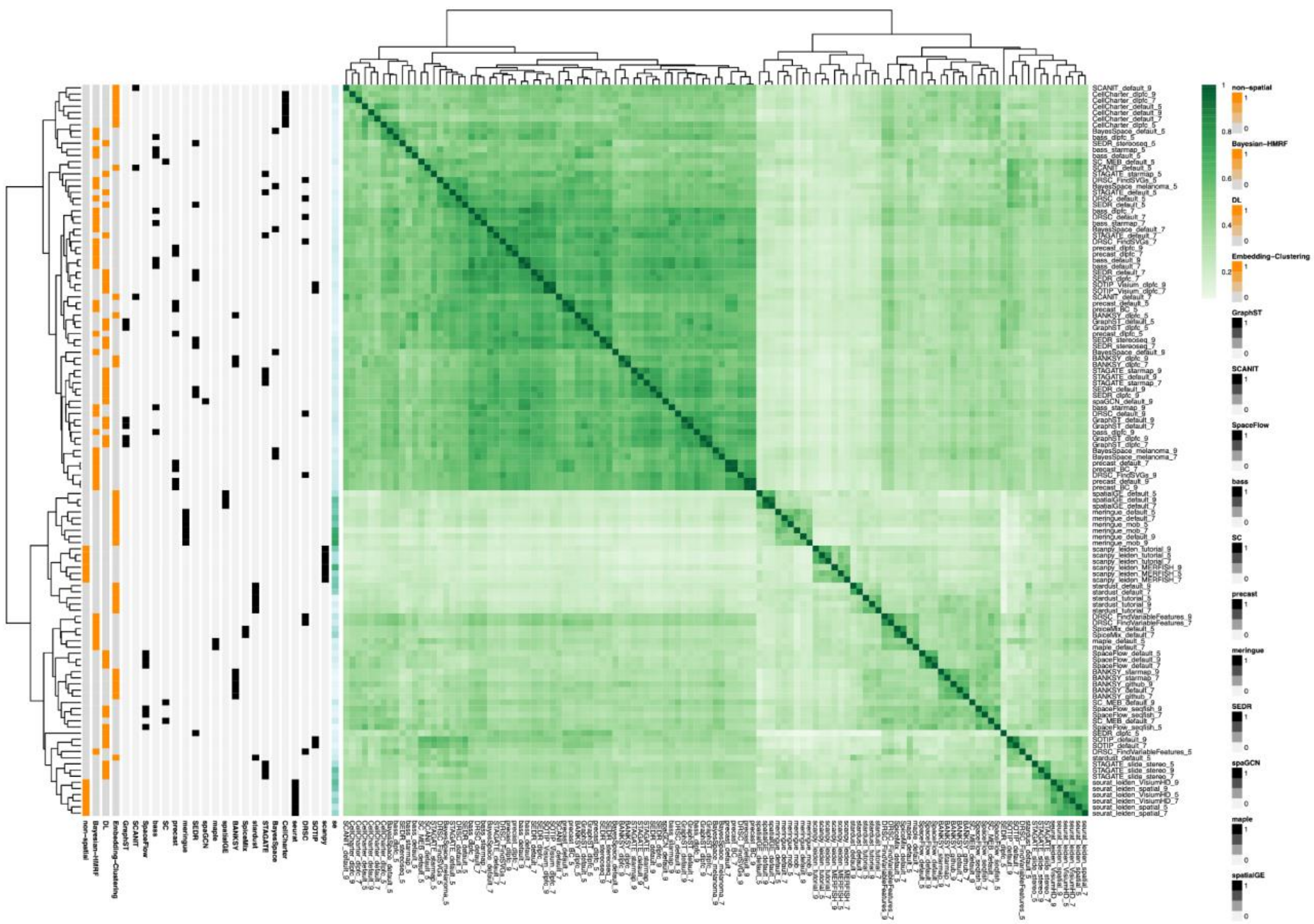

Reviewer-only Figure1: DLPFC method class. Heatmap shows the set of methods run on the DLPFC Slice 151673, and the blocks of similarity (similar to **Figure 3**). Row annotations were added with a column for each algorithm (light grey for absent, black for present) and for each algorithm class (medium grey for absent and orange for present).

*Furthermore, in the analysis that searches for a "sweet spot" of the number of clusters and number of base clusterings (**Figure 3E**), and the fact that we often run multiple configurations of the same algorithm, we actually directly impose that a single algorithm is not used more than once in the consensus for this analysis. **This is now explicitly written in the Methods section**:* In some cases, we have multiple clusterings from the same algorithm (different parameter sets) with the same number of clusters; to build the consensus (as described above), however, we enforce algorithm diversity by only keeping one base clustering (the smoothest) per algorithm (***Supplementary Figure S22)***.

*Following on from the point above about mixing of algorithms and algorithm classes, we computed pairwise ARI heatmaps (across a set of base clusterings) for 3 other datasets and also observe in these datasets considerable mixing of algorithms (black row annotation) and algorithm classes (orange row annotations, **Reviewer-only Figures 2-4**).*

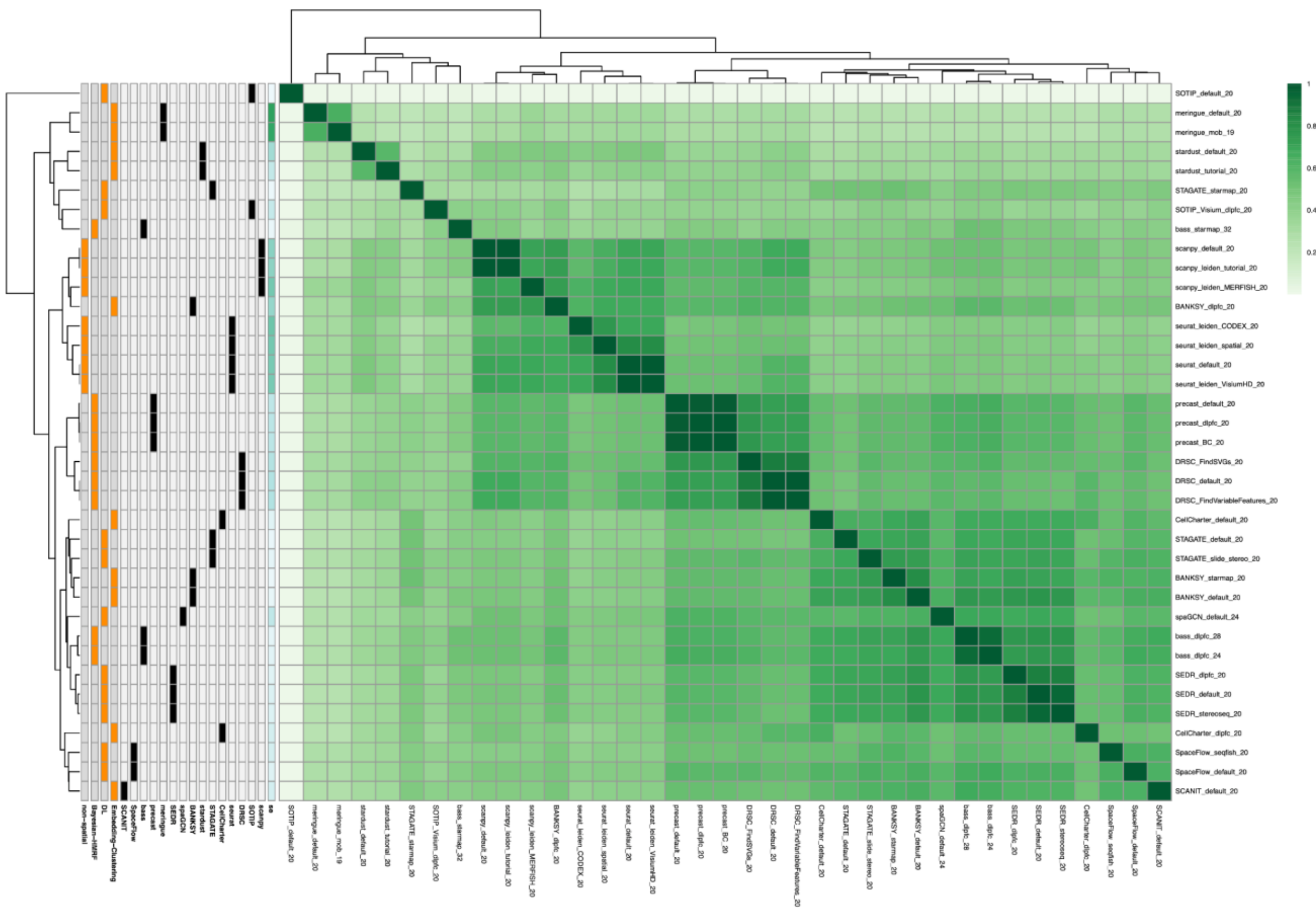

Reviewer-only Figure 2: Thalamus MERFISH dataset at 20 clusters. Heatmap shows the pairwise ARI across the set of methods run on the mouse_thalamus dataset (slice 6800), and the blocks of similarity (an extension of methods shown in **Figure 4**). Row annotations were added with a column for each algorithm (light grey for absent, black for present) and for each algorithm class (medium grey for absent and orange for present).

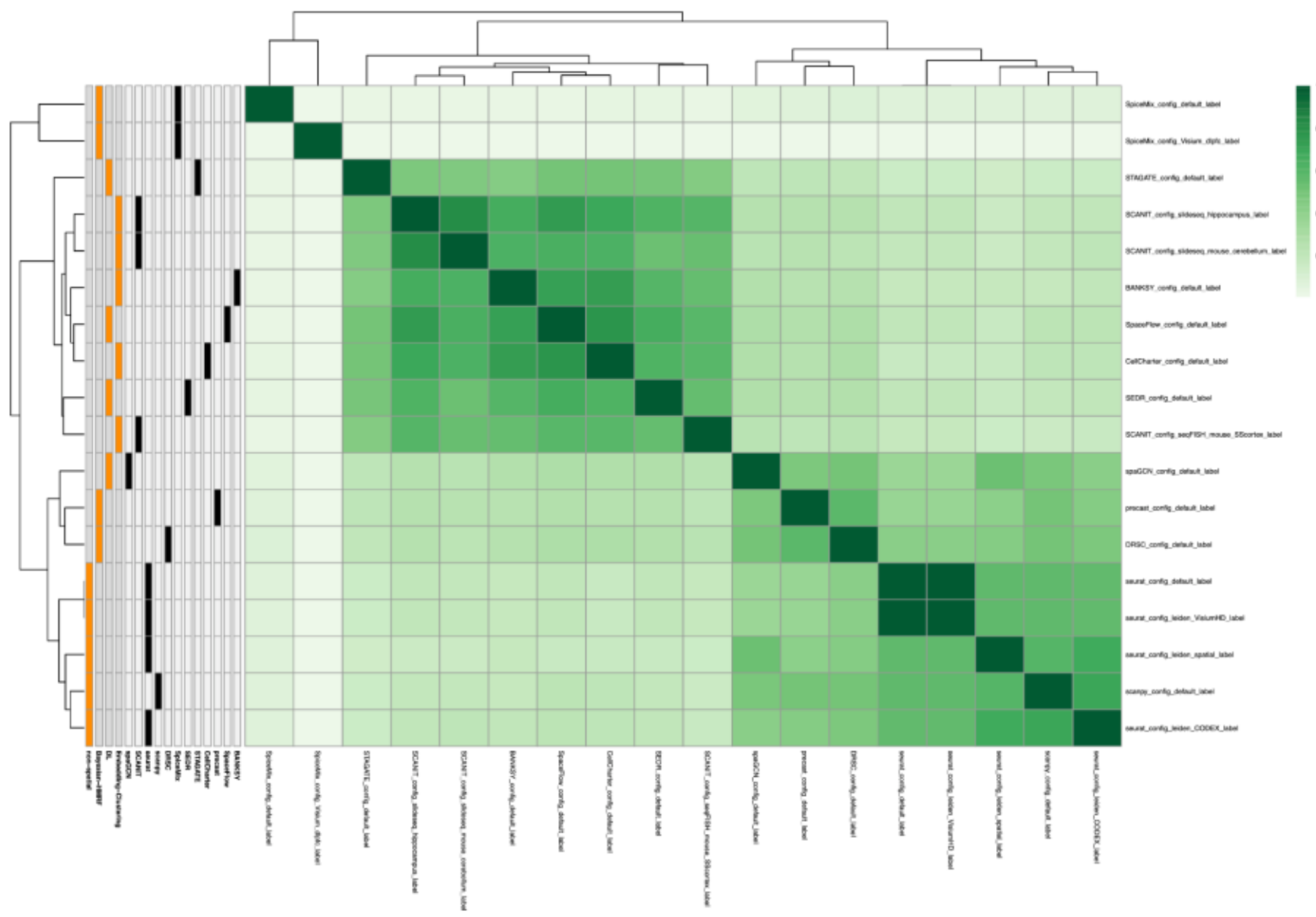

Reviewer-only Figure 3: mouse brain Xenium dataset. Heatmap shows the pairwise ARI across the set of methods run on the mouse brain Xenium dataset, and the blocks of similarity (similar to **Figure 3**). Row annotations were added with a column for each algorithm (light grey for absent, black for present) and for each algorithm class (medium grey for absent and orange for present).

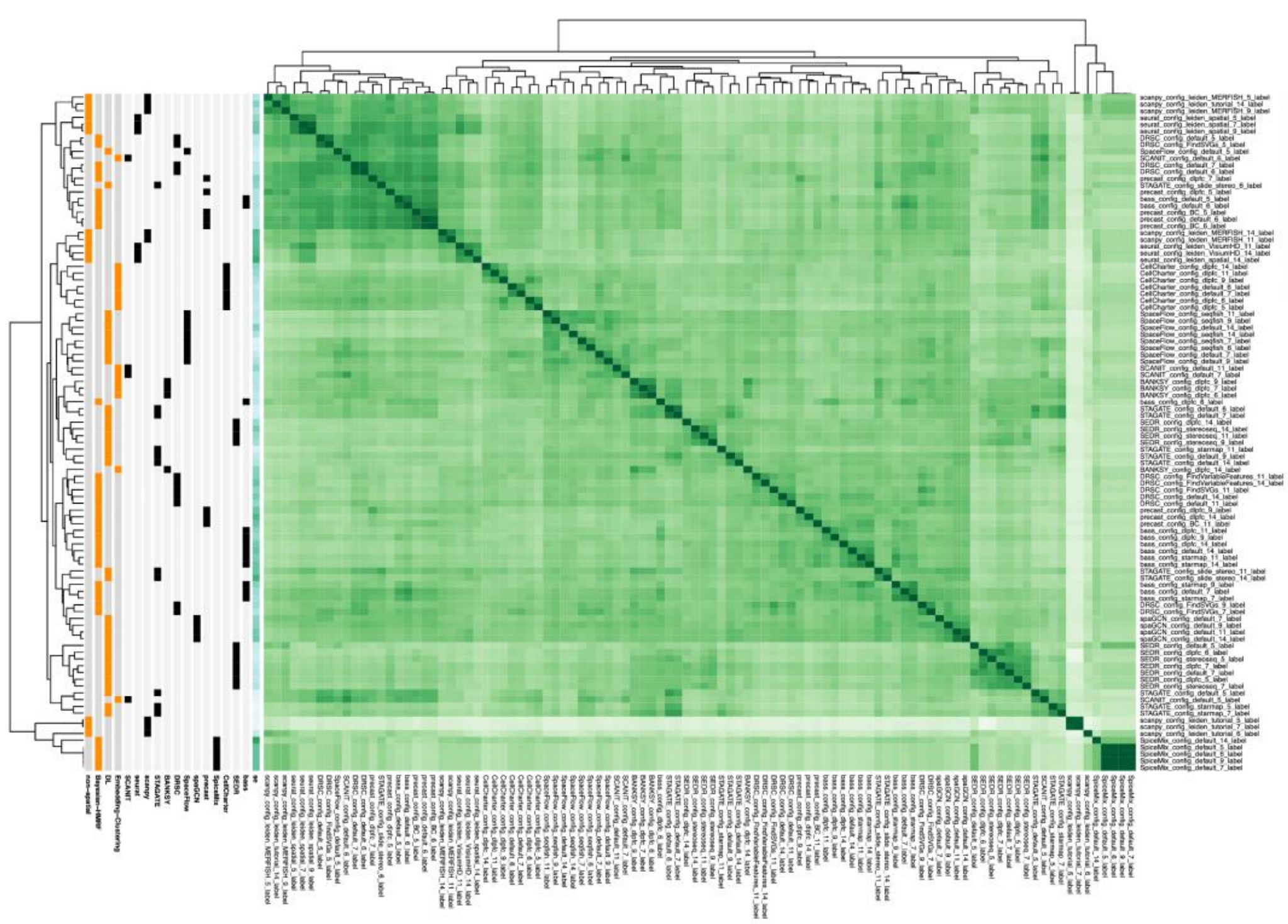

Reviewer-only Figure 4: Visium HD colon cancer. Heatmap shows the pairwise ARI across the set of methods run on the Visium HD colon cancer dataset, and the blocks of similarity. Row annotations were added with a column for each algorithm (light grey for absent, black for present) and for each algorithm class (medium grey for absent and orange for present).

In summary, we did not observe overrepresentation of single algorithmic classes in our analyses. Despite this, as with any consensus-building approach, there remains a risk that the consensus is dominated by algorithms of a similar class, and as such, we have added a comment in a new section called "Practical guidelines for an expert-in-the-loop workflow" that it is important to consider a diversity of clustering methods. And as we show above, the extent of algorithm mixing can be easily visualized across multiple base clusterings.

Added text to the new "Practical guidelines for an expert-in-the-loop workflow" section: Base clustering (BC) and the selection of consensus algorithms are pivotal steps in the workflow. Strategies for BC selection can generally be classified into three categories: concordance-based, ranking-based, and manual selection. Manual selection relies on expert feedback or contextual biological criteria, while the other two approaches can be flexibly tailored to the research question at hand. In our case studies, base clusterings were chosen based on concordance blocks identified within the cross-method ARI heatmap (***Figure 3B***), as well as rankings of application-specific metrics that evaluate the spatial smoothness of clusters, such as SE ***(Supplementary Figure S22***). Given the diversity of metrics included in the workflow, we encourage users to adapt these selection strategies to whichever metrics most effectively capture the biological phenomena of interest. Since consensus clustering aims to synthesize outputs from methods based on different modeling frameworks, a valuable sanity check during BC selection is to ensure a mix of clustering methods.

Revised text (Discussion section)*:* In addition to the number of base clusterings included, users should also consider the diversity of the selected methods in terms of their underlying algorithms. Since we envision the use of consensus clustering within an exploratory analysis without clear prior knowledge on the expected tissue clusters, aiming for a sufficiently diverse set of base clustering is desirable.

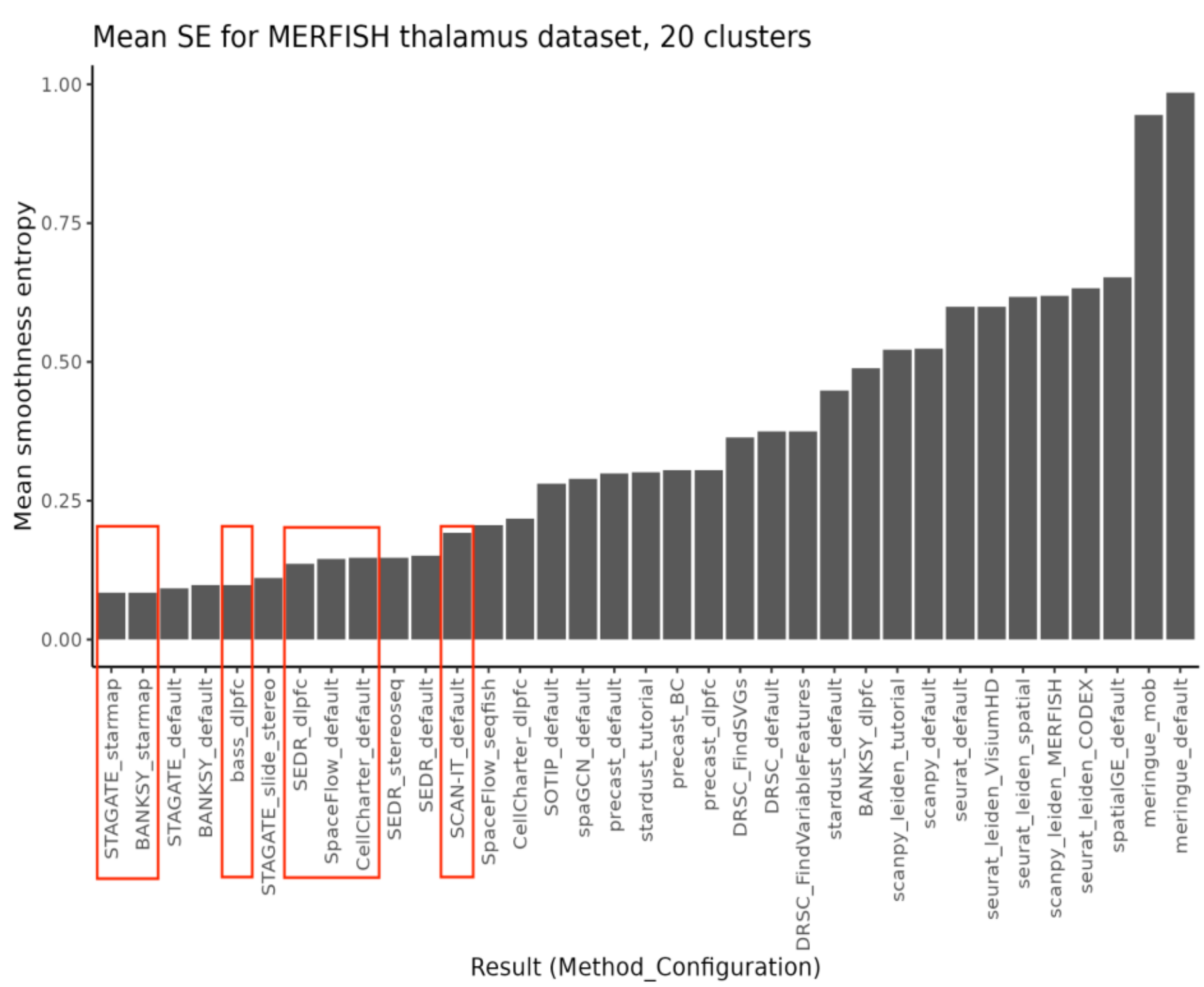

Supplementary Figure S22. Smoothness entropy of the MERFISH thalamus dataset with 20 clusters. Selected instances of base clusterings are marked by red boxes. Only the smoothness configuration of each method is selected.

3. Consensus methods

The manuscript implements LCA, K-modes, and weight-based consensus without justification. Please add a Methods paragraph that states (i) why these three were selected, (ii) their underlying assumptions (e.g., local independence in LCA), and (iii) recommended usage scenarios.

*We thank the reviewer for the suggestions. The three methods were selected as representative examples of different consensus algorithm categories, and we have added more detailed descriptions, justification, and assumptions of each method. We also encourage users to incorporate and explore other reasonable choices beyond the three demonstrative ones. Additionally, we also addressed both the computational and biological concerns for usage scenario recommendation. For computational concerns, we conducted a scalability analysis for the consensus methods (**Supplementary Figure S23**). Please also refer to the response to reviewer 1 comment 2, where we describe the scalability of the consensus algorithms in detail. To guide users with their biological question-specific analysis, we incorporated an expert-guided workflow instruction and workflow checklist for consensus algorithm selection (**Supplementary Figure S21, Supplemental File 1**). We have also added a paragraph in the results section aiming at providing more guidance and insights for consensus algorithm selection.*

Added text to the new "Practical guidelines for an expert-in-the-loop workflow" section: The choice of consensus algorithm should be tailored to the specific research question, taking into account tissue type, spatial technology, and the underlying biological hypothesis. In this study, our recommendations are primarily guided by scalability considerations (***Supplementary Figure S23,*** *see above*). We assessed runtime and memory usage for the three implemented consensus algorithms (K-modes, LCA, and weighted consensus) across a range of dataset sizes (i.e. number of cells or bins) and the number of input base clusterings. Overall, resource requirements increase with dataset size for all three methods. K-modes scaled best to large datasets, followed by LCA and weighted consensus. When varying the number of base clusterings, no consistent trend in runtime was observed; however, LCA showed a tendency toward higher memory consumption with increasing numbers of base clusterings. Across both variables, K-modes was the most resource-efficient, Weighted consensus was the most resource-intensive, and LCA showed intermediate performance.

Revised "Consensus clustering algorithms (Method)" section (revision in red): Three consensus clustering approaches were implemented in the framework and applied in the paper: K-modes, Latent Class Analysis (LCA), and weight-based consensus. While many consensus algorithms exist, these three were selected as representative examples to demonstrate the flexibility of the consensus workflow.

K-modes was selected as the categorical analogue of K-means. In the context of consensus clustering, each base clustering contributes to the consensus clustering equally, and their labels serve merely as nominal categories without ordering or magnitude, which aligns with the assumption of the K-modes method. As an algorithmic approach to consensus clustering, K-modes aims to find clustering partitions that minimize the overall hamming distance to cluster-specific mode vector. This approach returns interpretable results in a scalable manner, especially for big datasets (***Supplementary Figure S23***). In the SACCELERATOR framework, K-modes is implemented via R package diceR.

LCA was chosen as a representative model-based consensus clustering approach. LCA assumes that there exists an unobserved categorical latent class (the consensus clustering) that explains the observed base clusterings, and that individual base clustering results are conditionally independent given the latent class. Unlike the distance-based K-modes, LCA is a probabilistic approach that allows for flexible

handling of base clustering disagreement by method-specific conditional probabilities. In the SACCELERATOR framework, LCA is implemented via R package diceR.

Additionally, we also implemented a weighted-based consensus based on a Bregmannian divergence framework utilizing the weighted Jensen-Shannon Divergence (JSD) as the loss function:

$$\min_{S,\{\omega_m\}} \sum_{m=1}^{M} \omega_m \,\mathrm{JSD}(S \parallel S^{(m)}) + \lambda \sum_{m=1}^{M} \omega_m \log \omega_m$$

subject to

$$\sum_{m=1}^{M} \omega_m = 1, \quad \omega_m \geq 0.$$

where M is the number of base clusterings, S is the binary similarity matrix for consensus results, $S^{(m)}$ is the binary similarity matrix of individual base clusterings, and ω_m are the respective weights of each base clustering. The second term is an entropy regularization of the weights to prevent the inclination of the binary similarity matrix to any single base clusterings. The resulting binary similarity matrix S was clustered using the Leiden algorithm to generate the final consensus clusters. Unlike LCA and K-modes, the weighted consensus also returns the weight terms assigned to each base clustering, providing an interpretable understanding of the relative contributions of different methods. However, the incorporation of large binary matrices limits the scalability of this method (Supplementary Figure S23).

Although the three approaches (K-modes, LCA, and weighted consensus) are already integrated into the workflow, we encourage users to explore additional consensus algorithms as needed, such as majority voting or linkage clustering ensemble. The choice of method should be guided by both computational considerations (e.g., scalability, ***Supplementary Figure S23***) and the specific biological questions of interest, ideally in consultation with domain experts (see ***Supplementary Figure S21*** for an overview of the expert-guided workflow instruction).

4. Spatial-omics technology classification and comparison

4a. The current wording (lines 152–53) conflates technology names with resolution tiers:
Consider revising to a resolution-based description:
“Technologies operating at bulk (original 55 µm Visium), cellular (~5–10 µm; Slide-seqV2, Xenium), and sub-cellular (<2 µm; ,Visium HD MERFISH, STARmap PLUS, Stereo-seq, CosMx).”

*We thank the reviewer for this clarification. The sentence has now been removed as it is not central to the main part of the manuscript anymore due to the modification of **Figure 1D** (see response to comment 5 below).*

4b. The manuscript includes 10 different platforms across various datasets (including CosMX, MERSCOPE, MERFISH, STARmap, Xenium, Slideseq, Stereo-seq, Visium, osmFISH, and Visium HD). However only 2 case studies are discussed in detail for Visium HD and MERFISH.
‘MERSCOPE’ appears 3 times in the manuscript in the Methods section and I cannot see any discussion about the Visium chicken heart data. More results and discussion that brings out any insights gained across a wider range of platforms would be beneficial.

We included a wide breadth of datasets with the aim to (1) cover different tissue types beyond the brain, which has been heavily used in the context of evaluating spatial clustering methods, and (2) to cover more recent technologies. As such, we choose to focus on our use cases on the thalamus dataset measured using MERFISH as a representative of high-resolution, panel-based technologies (such as Xenium and CosMx) and the colorectal cancer dataset measured using VisiumHD as a representative for sequence-based methods (such as Stereo-seq and Slide-seq v2). We believe these two case studies cover the most up-to-date technologies in use by most research labs.

*We agree with the reviewer that expanding our use cases to more datasets can lead to additional biological insights. But this would require access to different tissue experts to ensure the validity of our reported findings, similar to the two use cases presented. To address the reviewer's remark, we have now included the primary results of the consensus clustering of six additional datasets covering a wider range of tissues and technologies. This includes breast cancer (Visium), liver (stereo-seq), lung (CosMx), mouse brain (Xenium), mouse brain (STARmap-plus), and ABC Atlas (MERFISH) (**New Supplementary Figures S25-S29**). We would like to note that these results are merely meant to illustrate the generalizability of our approach to different datasets and technologies and should not be used directly for biological interpretations. As they have not been checked by tissue experts, they are considered preliminary and are intended for those who are interested in exploring further. We noted this explicitly in our Zenodo repository.*

*In addition, we believe it is important to explicitly mention all datasets that were processed to also show the community which of those datasets have human vs computer-generated reference annotations (last column of **Table 1**) to emphasize the limitations of previously reported benchmarks. As a community-initiated project, we have implemented scripts to automatically download and format those datasets. Even though extensive case-wise analyses requires more computational resources and biological expertise beyond the scope of our project, we intend to include those datasets to encourage community adaptation and further exploration of those datasets.*

Finally, we have included a schematic that illustrates the SACCELERATOR *approach and a checklist for hands-on practice using our workflow. Please see also the response to Reviewer 1 Comment 1 for a detailed explanation of the added workflow description. We have also incorporated and reflected those concerns in the new results section.*

Added text to the new "Practical guidelines for an expert-in-the-loop workflow" section: Although only two case studies were presented, the selected datasets represent two major classes of spatial transcriptomic technologies: imaging-based (MERFISH) and sequencing-based (Visium HD). They also cover tissue types that particularly benefit from spatial context, including brain and solid tumors. We expect that the practical guideline, comprehensive documentation, and aforementioned considerations will encourage the community to further evaluate and adapt the workflow across a wide range of settings. As a first step to facilitate those adaptations, we have included unsupervised (i.e., no expert involvement) consensus results from a selection of representative datasets covering different recent technologies (***Supplementary Figure S25-29***, Data Availability) as an initial resource to spur further investigation.

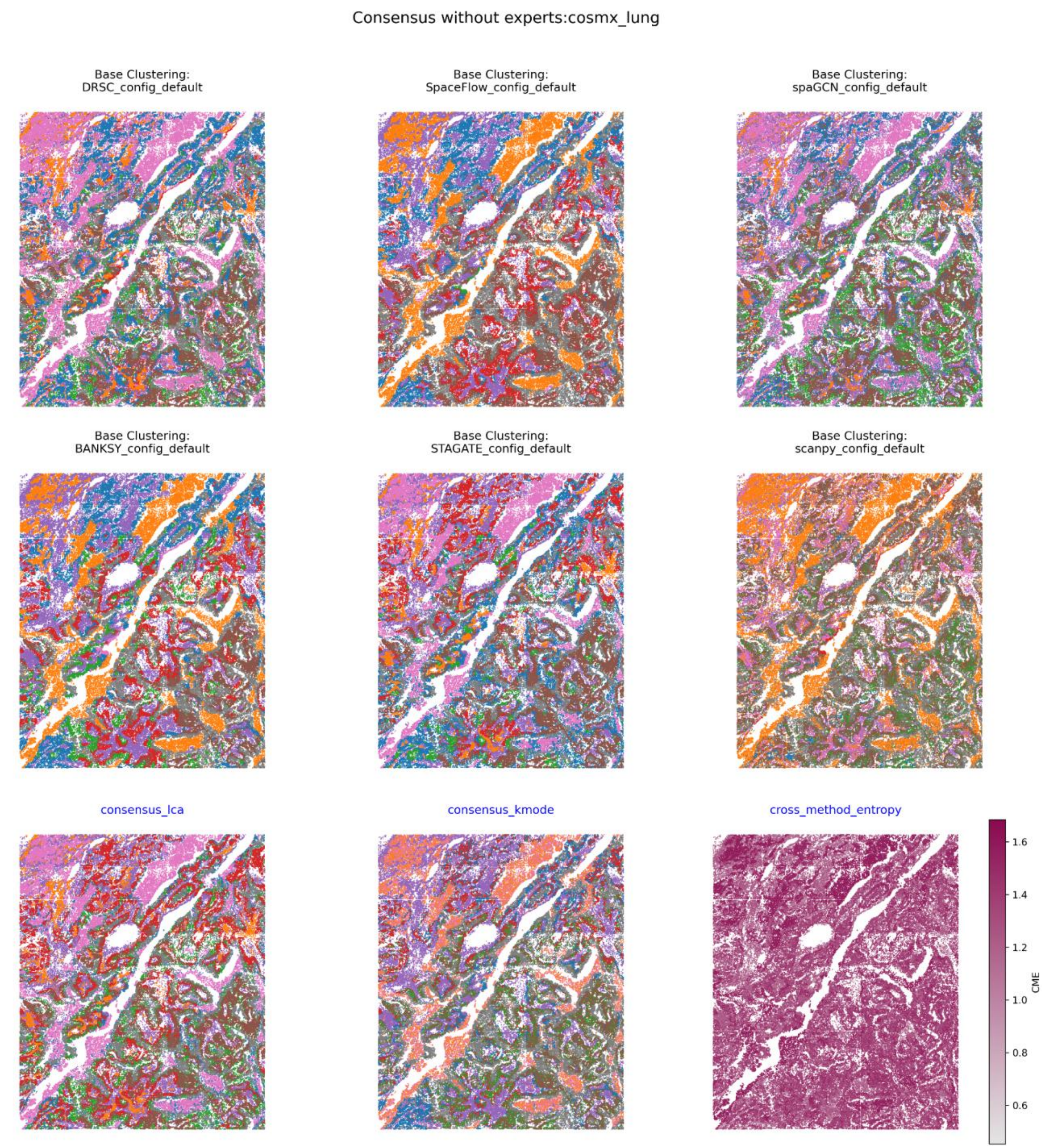

Supplementary Figure S25: Base and consensus clusterings for the CosMX lung dataset. (A-F) Base clustering from methods using default configurations set to 8 clusters. (G-H) Consensus clustering using LCA and kmodes consensus building. Results for weighted are not shown as the computation did not complete. (I) Cross method entropy heatmap.

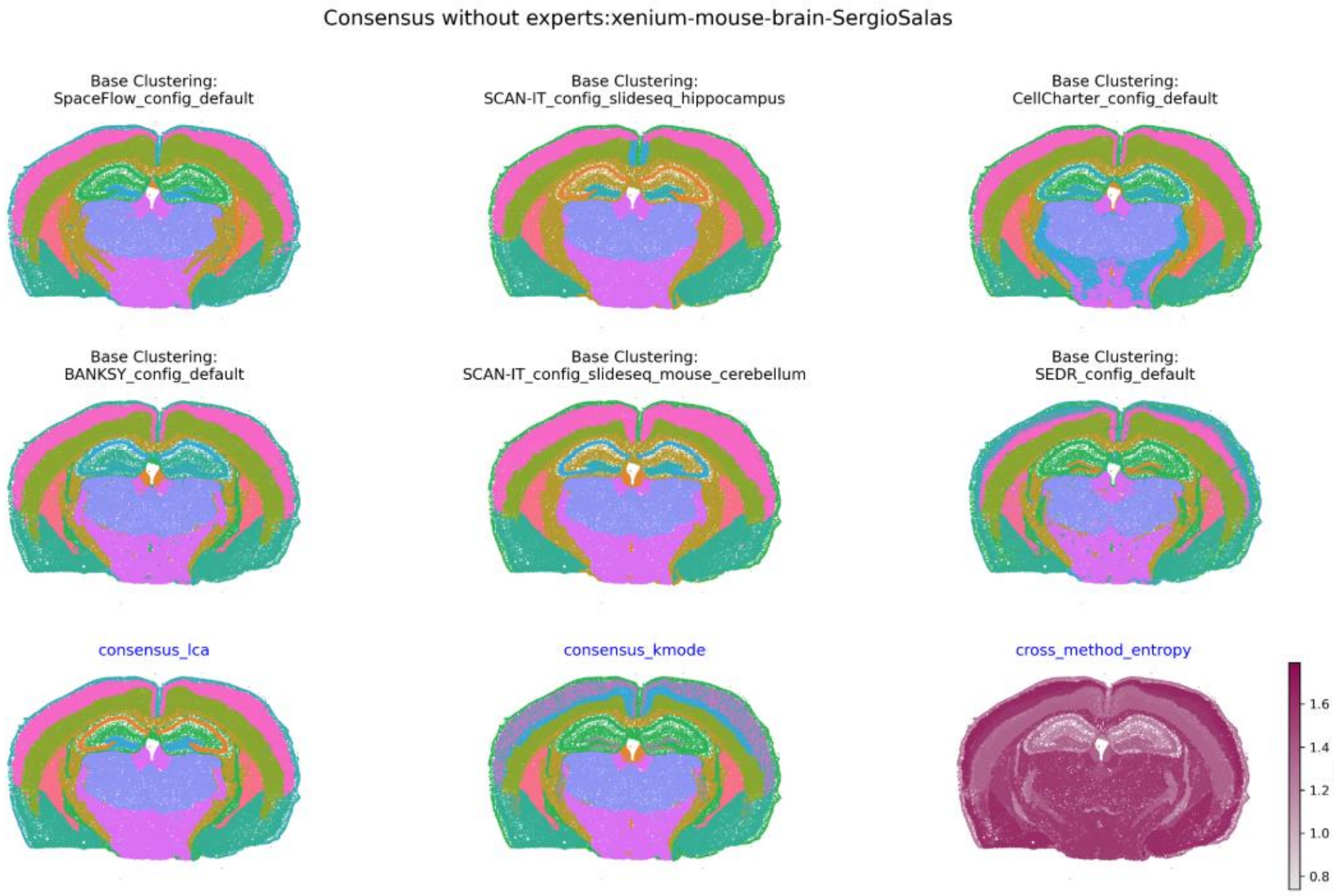

Supplementary Figure S26: Base and consensus clusterings for the Xenium mouse brain dataset. (A-F) Base clustering from methods using default configurations set to 11 clusters. (G-H) Consensus clustering using LCA and kmodes consensus building. Results for weighted are not shown as the computation did not complete. (I) Cross method entropy heatmap.

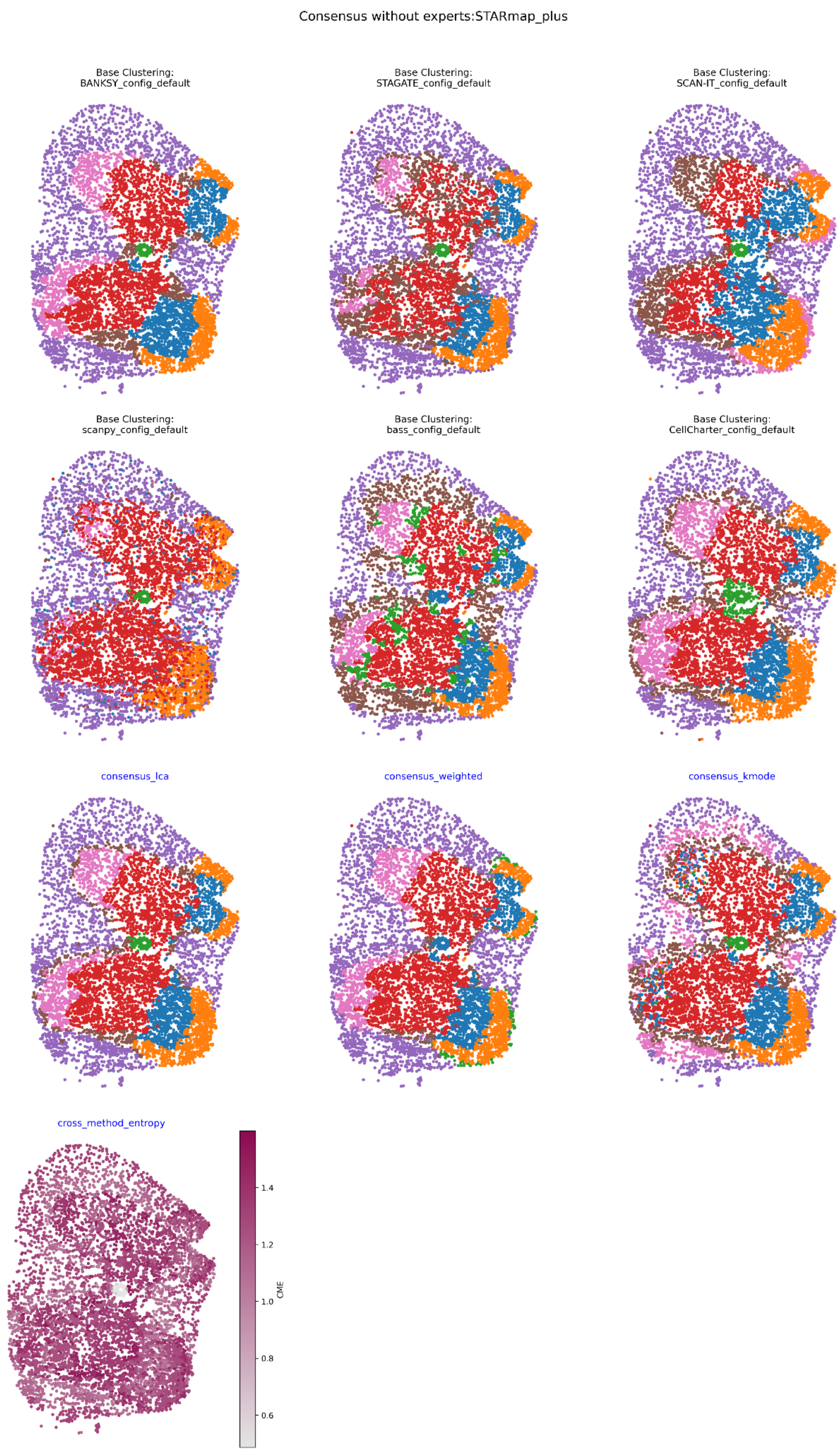

Supplementary Figure S27: Base and consensus clusterings for the STARmap plus mouse brain dataset. (A-F) Base clustering from methods using default configurations set to 7 clusters. (G-I) Consensus clustering using LCA, kmodes and weighted consensus building. (J) Cross method entropy heatmap.

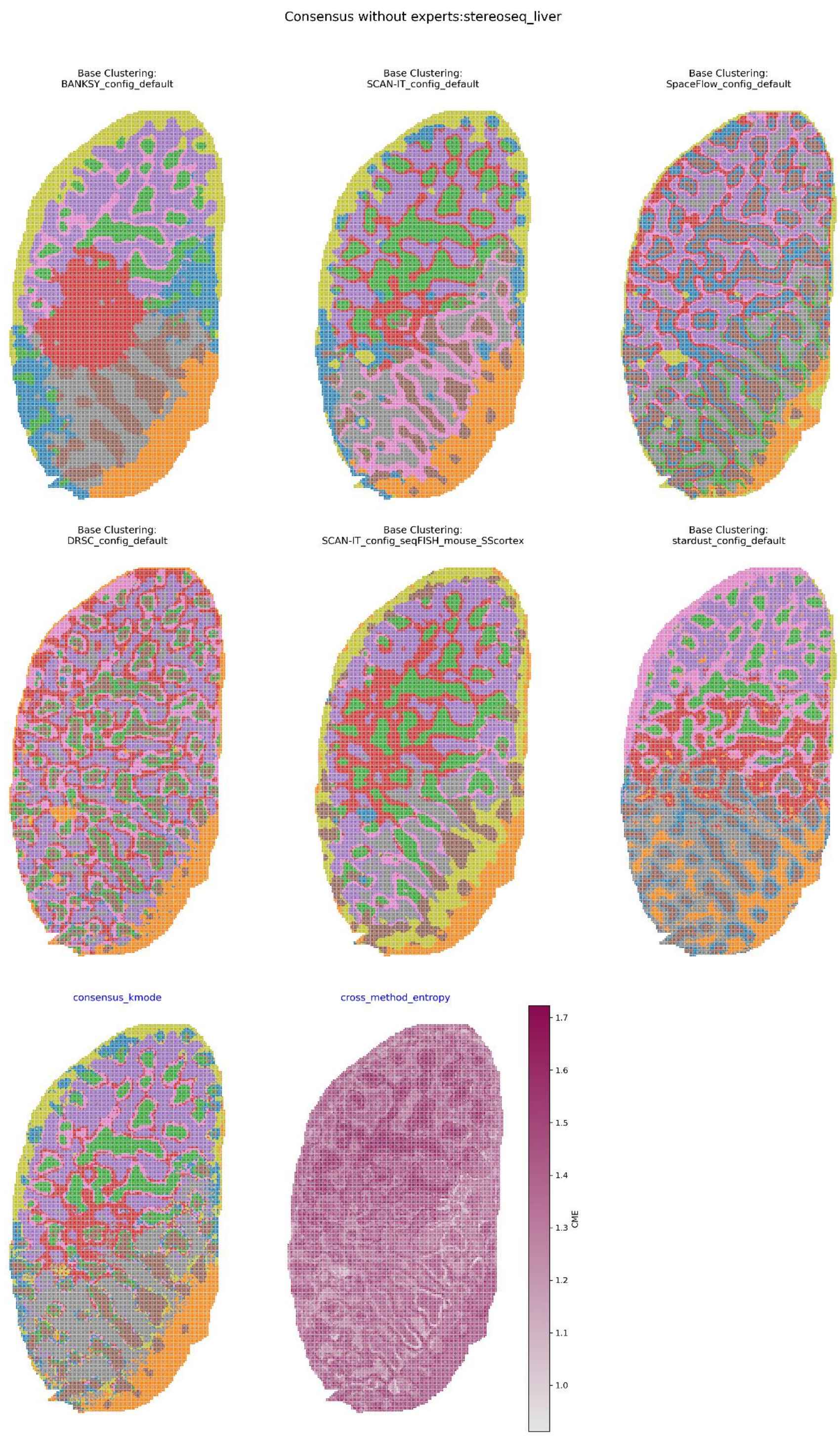

Supplementary Figure S28: Base and consensus clusterings for the Stero-seq liver dataset. (A-F) Base clustering from methods using default configurations set to 9 clusters. (G) Consensus clustering using kmodes. Results for LCA and weighted are not shown as the computation did not complete. (H) Cross method entropy heatmap.

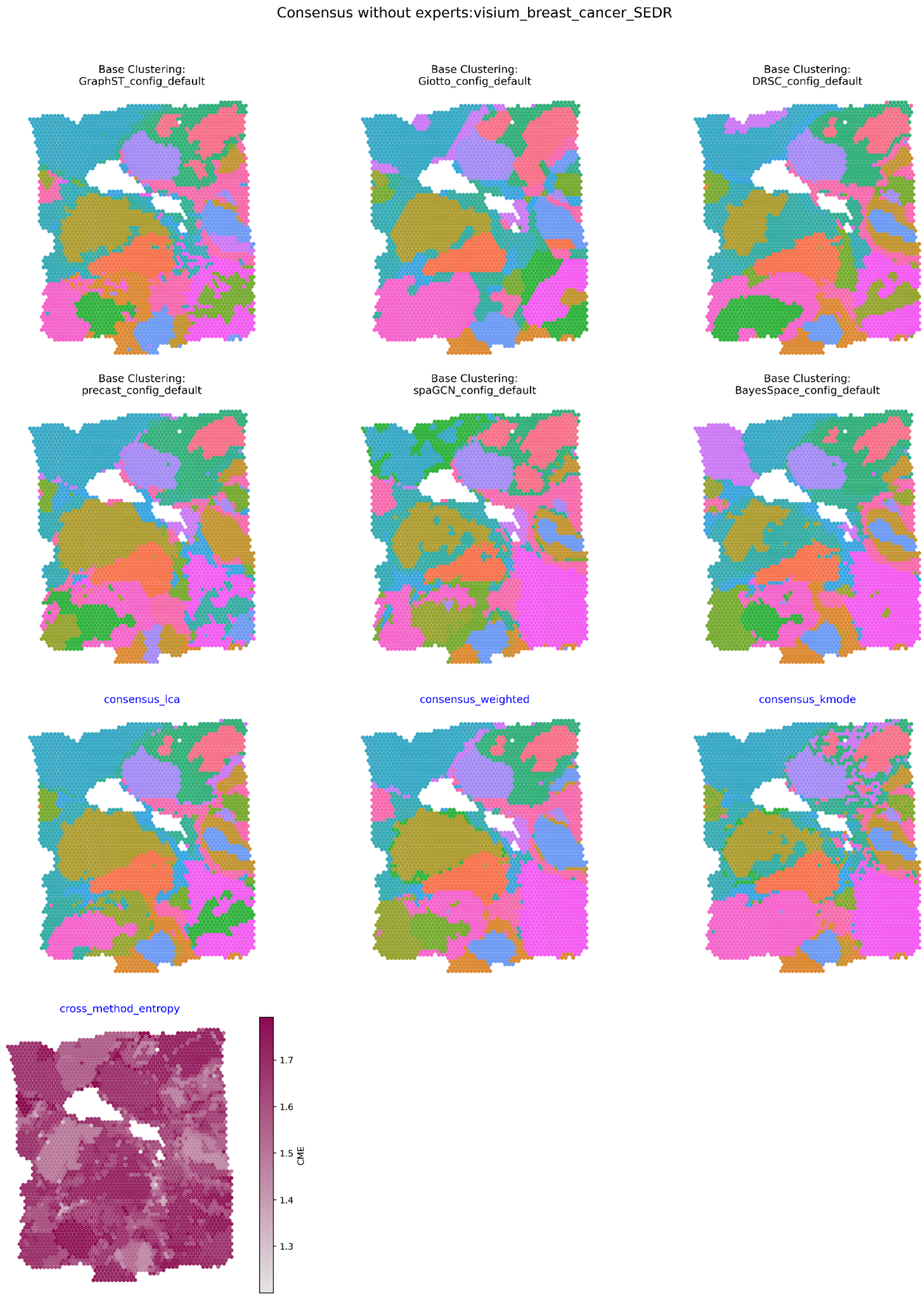

Supplementary Figure S29: Base and consensus clusterings for the Visum breast cancer dataset. (A-F) Base clustering from methods using default configurations set to 20 clusters. (G-I) Consensus clustering using LCA, kmodes and weighted consensus building. (J) Cross method entropy heatmap.

4c. Page 18 ‘Slide-seqV2 mouse brain olfactory bulb dataset’ link appears to point to a Stomics dataset – please check and correct to the appropriate link.

The table on Page 18 only linked to the paper reference. We have verified that the link on Page 15 describing the Slide-seq v2 dataset is correct - indeed, the StomicsDB link is for the Slide-SeqV2 data. Furthermore, we have increased consistency of URLs pointing to resources on the same domain.

5. Presentation of results

5a. Figure 1E is hard to read (multi-colours within each violin plot representing different paramter configurations). A different display (separate violins per config?) might be easier to interpret.

*We acknowledge that the multi-colored violin plots may be hard to interpret and have adjusted the corresponding visualizations of **Figure panels 1D** and **1E** (for convenience, included below).*

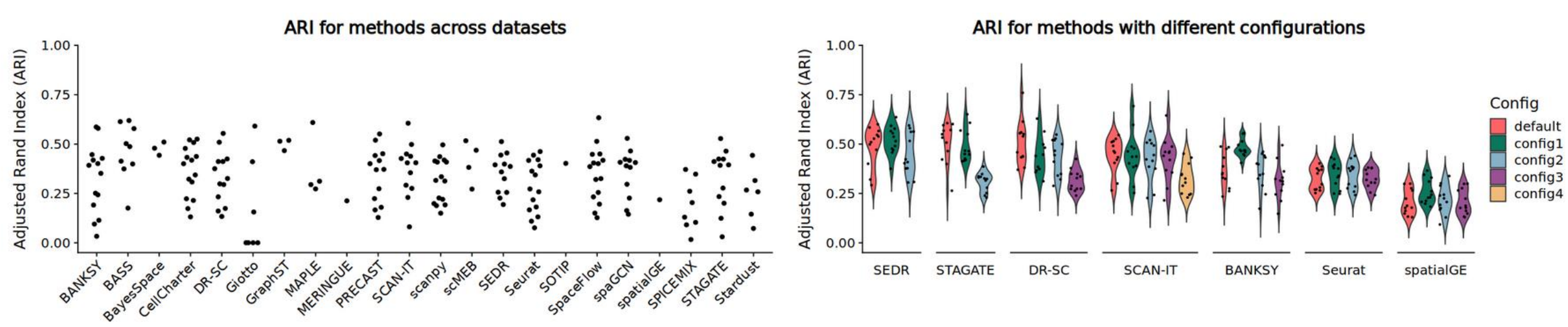

Revised Figure Panels 1D, 1E: (D, left) ARI for methods across different spatial resolutions of SRT technologies; (**E, right**) ARI for methods using different configurations applied to the LIBD DLPFC dataset.

5b. Figure 2B. Consider extending this analysis [to] other sample types (human cortex may be an edge case here, compared to mouse brain / breast cancer samples for example).

*We thank the reviewer for the suggestions and have extended the analysis to both Visium Human Breast Cancer and MERFISH Mouse Brain Thalamus datasets (**Supplementary Figure S7**). Similar patterns have been observed in both cases. We have added the analysis results in the Supplementary Figures and added necessary descriptions in the main text.*

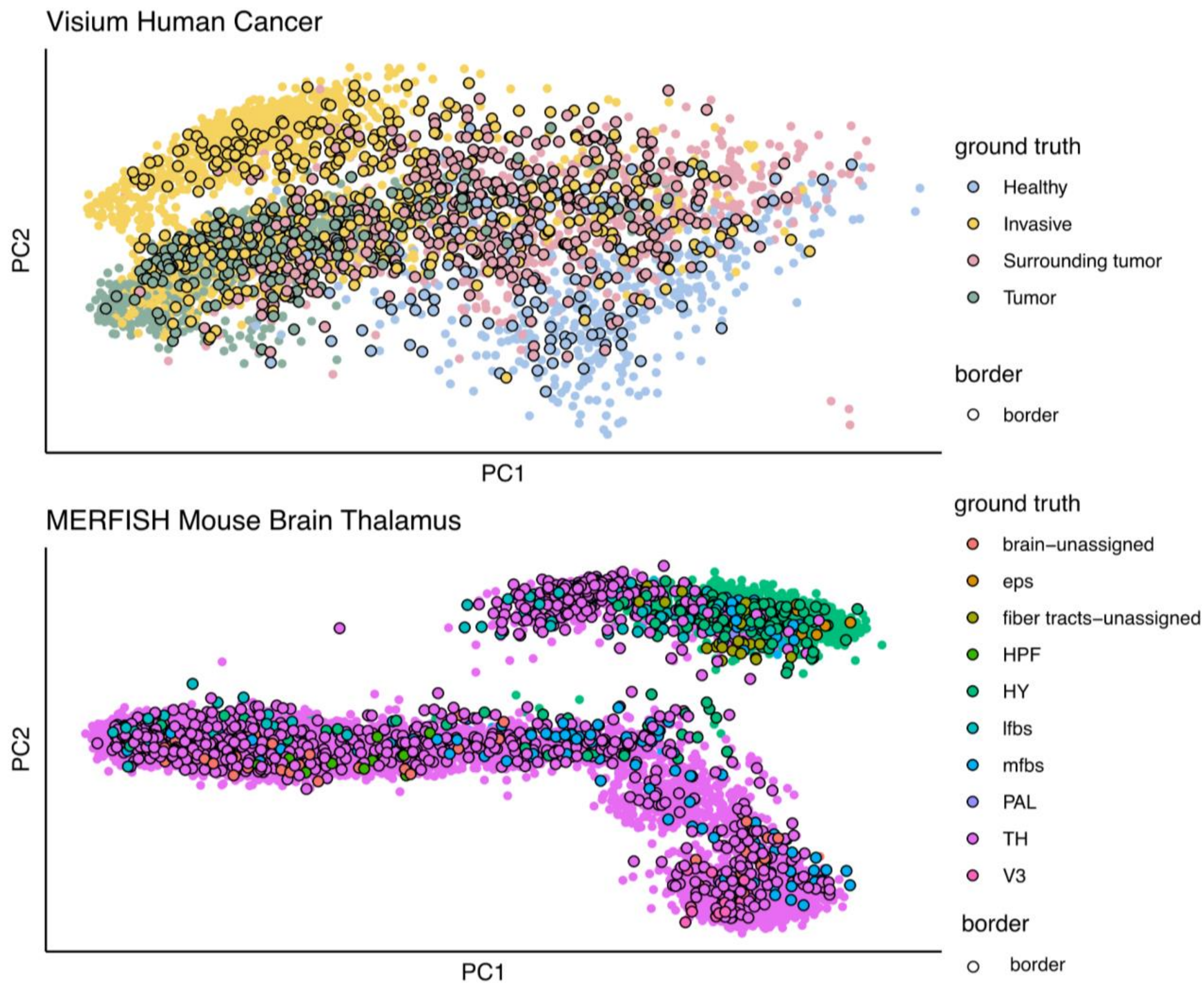

Supplementary Figure S7. Visium human cancer, and MERFISH mouse brain thalamus datasets, projected on the 1st and 2nd principal component (PC), colored by GT labels. Spots that are spatially located at the border between GT clusters are marked with black outlines.

Added text: Similar intermixed patterns can also be observed in other datasets (Supplementary Figure S7).

5c. Supplementary Figure 3. Visium and Visium HD should use different colours to help the reader distinguish between them, as there are large differences in spatial resolution.

We thank the reviewer for the attention to detail and have corrected this oversight on our part.

Reviewer #3 (Remarks to the Author):

I co-reviewed this manuscript with one of the reviewers who provided the listed reports. This is part of the Nature Methods initiative to facilitate training in peer review and to provide appropriate recognition for Early Career Researchers who co-review manuscripts.

Reviewer #3 (Remarks on code availability):

This codebase provides excellent value for researchers in spatial omics. While initial setup requires technical expertise, the modular design and comprehensive documentation make it a worthwhile investment for laboratories conducting spatial clustering analysis.

We thank the reviewer for their acknowledgment of the value of our codebase.

General response

We sincerely thank the editor and reviewers for their rapid and positive evaluation of our manuscript.

Below, we provide our point-by-point responses. To facilitate the review, we have modified the comment numbering for clarity. Our responses are presented in blue text, and new text or figures added to the manuscript are highlighted in red.

Reviewer #1 (Remarks to the Author):

The authors have appropriately addressed my previous comments, and I recommend acceptance of the paper.

Reviewer #2 (Remarks to the Author):

We thank the authors for their thoughtful responses to our comments.

2.1) A final point that the authors might consider commenting on in the Discussion is that expert assessment itself will have some level of subjectivity which may affect results (e.g. Figure 5C-D). Assessing inter-expert variation is beyond the scope of this study, but something to be aware of given the human-in-the-loop aspect of the work.

Response: We agree with the reviewer and have added sentences to the discussion to address this point.

Added text: “Furthermore, expert evaluation remains essential, though subjective, for biological interpretation, and is best used alongside quantitative metrics (Supplementary Discussion).”

2.2) Minor: Figures 5D and S19 A-D y-axis label "Rank (lower is better)". Is lower better in terms of all the metrics shown (ARI, NMI, Smoothness Entropy, CHAOS)?

Response: We understand the potential for confusion. Although some metrics use lower scores to indicate better performance, in Figures 5D and S19A-D a lower rank is indeed better. To prevent confusion between ranks and metric scores, we have added a clarification to the legend of Figure S19.

Added text: “The lowest rank is assigned to the best score per metric i.e., highest value for ARI and NMI, and lowest value for Smoothness entropy and CHAOS.”

Reviewer #3 (Remarks to the Author):

I co-reviewed this manuscript with one of the reviewers who provided the listed reports.
